# Natural Products Inspired Scaffold Diversification Leads to Unnatural Molecular Warhead and Covalent Strategy to Modulating Protein Function through Electrophilic Bromine Transfer

**DOI:** 10.1002/advs.202512849

**Published:** 2026-04-30

**Authors:** Beau R. Brummel, Sofia Kokkaliari, Qiwen Gao, Jinjing Chen, Larissa Costa de Almeida, Srinivasarao Tenneti, Derek A. Leas, Eli Levit, Lewis S. Alexander, Khalil A. Abboud, Pingrong Wei, Michael D. Cameron, James R. Rocca, Ranjala Ratnayake, Donna D. Zhang, Hendrik Luesch, Robert W. Huigens

**Affiliations:** ^1^ Department of Medicinal Chemistry and Center for Natural Products, Drug Discovery and Development (CNPD3) College of Pharmacy University of Florida Gainesville Florida USA; ^2^ Department of Pharmaceutical & Biomedical Sciences College of Pharmacy University of Georgia Athens Georgia USA; ^3^ Department of Molecular Medicine Center for Inflammation Science and Systems Medicine The Herbert Wertheim UF Scripps Institute for Biomedical Innovation and Technology Jupiter Florida USA; ^4^ Department of Pharmacology and Therapeutics College of Medicine University of Florida Gainesville Florida USA; ^5^ Department of Chemistry University of Florida Gainesville Florida USA; ^6^ Department of Chemistry Franklin College of Arts and Sciences University of Georgia Athens Georgia USA; ^7^ Department of Molecular Medicine The Herbert Wertheim UF Scripps Institute for Biomedical Innovation and Technology Jupiter Florida USA; ^8^ McKnight Brain Institute University of Florida Gainesville Florida USA; ^9^ University of Florida Health Cancer Center University of Florida Gainesville Florida USA; ^10^ Program in Cancer and Stem Cell Biology Duke‐NUS Medical School Singapore Singapore; ^11^ Department of Infectious Diseases College of Veterinary Medicine University of Georgia Athens Georgia USA

**Keywords:** antioxidant response element, antioxidant response element activating agents, complexity‐to‐diversity, indole alkaloids, molecular warhead

## Abstract

We discovered a thiolate‐reactive α,α‐*gem*‐dibromo lactam warhead that activates transcription factor Nrf2 and demonstrates anti‐inflammatory activities, which have implications in cancer, neurodegeneration, and cardiovascular diseases. Our findings originated from new compounds accessed through aza‐oxyallyl cation‐mediated [3+2]‐cycloadditions of ajmalicine and inspired indoles. RNA‐seq and Ingenuity Pathway Analysis illuminated detailed transcriptional profiles of α,α‐*gem*‐dibromo lactams to show activation of the antioxidant Keap1/Nrf2 pathway and suppressed NF‐κB‐mediated inflammatory signaling. We demonstrated the importance of the α,α‐*gem*‐dibromo lactam, as the corresponding α,α‐*gem*‐dichloro lactam is unable to activate the Nrf2 pathway. Chemical reactions with cysteine‐containing compounds suggested α,α‐*gem*‐dibromo lactams react with cysteine residues to transfer electrophilic bromine to target molecules, linking unique chemistry to our biological findings. In contrast to electrophiles that activate the canonical Nrf2 pathway by covalently targeting C151 of Keap1, the α,α‐*gem*‐dibromo lactams function in a C151‐independent manner, indicating a distinct selectivity and consequently mode of action. Pharmacological profiling of several α,α‐*gem*‐dibromo lactams compared with ajmalicine against adrenergic receptors revealed substantial differences, indicating tunability and novel pharmacology. Future investigations aim to explore the fundamental activities of the dibromo lactam warhead related to human disease. Installation of this warhead into various molecular scaffolds may be a generalizable method to create cystein‐reactive electrophiles.

## Graphical abstract

We report a new thiolate‐reactive α,α‐*gem*‐dibromo lactam warhead that activates transcription factor Nrf2 and demonstrates anti‐inflammatory activities, which have implications in cancer, neurodegeneration, and cardiovascular diseases. RNA‐seq illuminated detailed transcriptional profiles, and chemical reactions with cysteine‐containing compounds suggested α,α‐*gem*‐dibromo lactams react with cysteine residues to transfer electrophilic bromine to target molecules, linking unique chemistry to our biological findings.

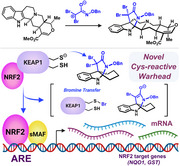

## 1 Introduction

Natural products and small molecules that covalently modify and modulate key cellular targets have played significant roles in drug discovery, medicine, and chemical biology [1, 2]. Nature has provided a diversity of molecules bearing electrophilic warheads that react with the nucleophilic groups on select molecular targets (i.e., amino acid residues, nitrogenous bases) to elicit biological activities desirable to the treatment of disease [3]. Electrophilic warhead chemotypes include (1) ring‐strained functional groups to promote reactivity (e.g., epoxides, cyclopropanes, aziridines, beta lactams, beta lactones), (2) non‐strained electrophiles (e.g., α,β‐unsaturated carbonyls, nitriles), and (3) latent functionality that requires modification to liberate an “armed” warhead (e.g., heminaminals, enamides, quinones; prodrug action) [4].

A multitude of natural products covalently modify their corresponding biological targets through mechanisms involving strained electrophilic warheads, such as (1) beta lactam antibiotics that selectively react with a nucleophilic hydroxyl group of a critical serine residue in transpeptidase enzymes in bacteria [5], (2) epoxomicin which bears an epoxide warhead that demonstrates potent inhibition of the 20S proteasome [6], (3) lipstatin covalently modifies gastric and pancreatic lipases through the action of its beta lactone warhead [7] and its derivative orlistat has found use in the treatment of obesity, and (4) duocarmycin natural products (e.g., CC‐1065) possess an electrophilic cyclopropyl warhead that reacts with AT‐rich sites to alkylate the nitrogenous base of DNA [8]. In addition, several natural products possess latent electrophilic warheads that require activation through an initial chemical transformation (similar to prodrug action), examples include: (1) mitomycin C which contains a quinone moiety that undergoes an intracellular bioreduction and subsequent aziridine ring opening to generate multiple electrophilic sites that undergo nucleophilic attack by guanine to crosslink DNA [9], (2) the enediyne natural product calicheamicin undergoes a series of transformations including intramolecular conjugate addition of a liberated thiol nucleophile to its electrophilic enone moiety, which facilitates a Bergman cyclization to form a diradical species that results in DNA strand scission [10] and is used to treat acute lymphoblastic leukemia, (3) ecteinascidin 743 bears a latent hemiaminal group that undergoes an acid‐promoted dehydration to an electrophilic iminium that reacts with guanine's N2 to form an alkylated DNA adduct and is approved for clinical uses as a cancer therapy [11], (4) select V‐ATPase inhibitors have a latent enamide warhead (e.g., salicylihalamide A, palmerolide A) [12, 13] that is postulated to undergo protonation in the acidic tumor microenvironment to give an armed *N*‐acyl iminium electrophile that subsequently reacts with a critical lysine residue to covalently modify V‐ATPase resulting in potent anticancer activities, and (5) lactacystin is a natural product that is transformed into the more reactive clasto‐lactacystin beta lactone warhead which targets and irreversibly inhibits the 20S proteasome [14, 15].

There is a growing number of compounds bearing a diversity of electrophilic warheads that have found clinical use as therapeutic agents and as probe moieties in chemical biology. Over the last decade, there have been multiple approvals for therapies adorned with α,β‐unsaturated amide warheads that covalently modify and inhibit various targets for cancer therapy, including: ibrutinib (targets Bruton's tyrosine kinase, or BTK), afatinib (inhibits epidermal growth factor receptor, or EGFR), osimertinib (mutant‐selective EGFR inhibitor), and sotorasib (targeting the KRAS^G12C^ mutant). Nirmatrelir (PF‐07321332) reversibly binds SARS‐CoV‐2's main protease 3CL through the action of a nitrile warhead that covalently modifies a key cysteine residue and has been approved for emergency use for the treatment of COVID‐19. In addition to clinical agents, small molecules that can modify select biological targets through the action of various electrophilic groups have played an important role in chemoproteomic profiling and the design of molecular glue degraders (e.g., discovery of covalent RNF114‐based degraders for PROTAC applications, fumarate‐based degraders of RING‐family E3 ligases) [16, 17]. Functionally, electrophilic warheads have been developed for covalent modification of various cysteine, tyrosine, aspartic acid/glutamic acid, serine/threonine, and lysine residues of protein targets [1, 2, 16, 17, 18, 19].

Here, we discovered an α,α‐*gem*‐dibromo lactam warhead that modulates biological targets through bromine transfer to cysteine residues shown through in vitro chemical and proof‐of‐concept cellular studies, focusing on the Keap1/Nrf2 pathway [20, 21, 22, 23] known to be regulated through Keap1 cysteine modifications, including measurement of functional transcriptome‐wide consequences. These findings originated from new chemistry related to our established ring distortion program that aims to rapidly generate diverse and complex small molecules from readily available indole alkaloids [24, 25, 26]. More specifically, these focused efforts aimed to synthesize novel ring fusion scaffolds from indole alkaloids through dearomative [3+2]‐cycloadditions with aza‐oxyallyl cations, followed by biological screens. Small molecule‐mediated activation of the Keap1/Nrf2 pathway has garnered significant interest due to its cytoprotective potential, as it enhances redox balance, metabolic homeostasis, and proteostasis, thereby improving cellular resilience to damage. Additionally, Nrf2 activation suppresses inflammation and collectively contributes to cancer prevention as well as the mitigation of various neurological and cardiovascular diseases [20, 22, 23, 27, 28, 29].

**FIGURE 1:**
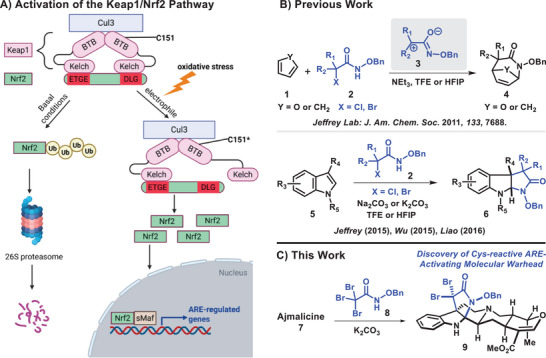
(A) Molecular details regarding the canonical activation of the Keap1/Nrf2 pathway. Nrf2 is bound to the Kelch domain of Keap1 through ETGE and DLG motifs. Under basal conditions, Nrf2 is ubiquitinated by the Keap1‐Cul3 complex, transferred to the 26S proteasome, and degraded (left). Under oxidative stress, Keap1 undergoes modification at one or more of its cysteines, which suppresses the ubiquitination of Nrf2. C151 is the most critical cysteine with respect to functional consequences. C151^*^ indicates the modified cysteine upon reaction with electrophiles. Nrf2 translocates to the nucleus where it dimerizes with sMAF proteins, resulting in activation of ARE‐regulated gene transcription (right). (B) Previous work on aza‐oxyallyl cation‐mediated cycloaddition reactions. (C) Generation of new ring fusion scaffolds from cycloadditions of aza‐oxyallyl cations with ajmalicine, a complex indole alkaloid.

Nrf2 is a transcription factor that binds to the antioxidant response element (ARE) in the promoter region of antioxidant and detoxification genes [20, 23]. Activation of Nrf2 has been linked to cancer prevention and anti‐inflammatory activity through inhibition of the proinflammatory transcription factor NF‐κB, which prompted, in part, our motivation to screen for activators of the Nrf2/ARE pathway. The activity of Nrf2 is regulated by the cytoplasmic repressor Keap1, which functions as a substrate adaptor for a Cullin 3‐based E3 ubiquitin ligase complex (Figure 1A) [30, 31]. In normal cells, Nrf2 activation facilitates cell survival and prevents carcinogenic effects of reactive oxygen species (ROS). However, in cancer cells, somatic mutations in NFE2L2 or KEAP1 can lead to constitutive Nrf2 activation, driving chemoresistance by enhancing redox balance, metabolic adaptation, and drug efflux. While Nrf2 activation is protective in normal cells and plays a role in cancer prevention, its persistent activation in tumors can promote therapy resistance. Therefore, Nrf2 modulation must be context‐dependent, with activation beneficial for cancer prevention and inhibition potentially useful for sensitizing certain Nrf2‐addicted cancers to treatment [23]. Under basal conditions, Keap1 forms a homodimer through its BTB domain that binds to Nrf2 in a 2:1 ratio, with one Kelch domain binding to the ETGE motif and the other to the DLG motif, allowing for the ubiquitination by the Cullin3 complex and subsequent degradation of Nrf2 by the 26S proteasome [21, 23, 28, 29, 30, 31, 32]. During oxidative stress, the cysteine‐rich Keap1 reacts with ROS or undergoes alkylation by electrophilic compounds, preventing the ubiquitination of Nrf2 and leading to Nrf2 stabilization. Then Nrf2 undergoes nuclear translocation and activates Nrf2 target genes, which represents the canonical (Keap1 cysteine‐dependent) pathway. Michael acceptors, the most common pharmacophore for Keap1 alkylation, commonly act as soft electrophiles on multiple cysteines with different selectivity but predominantly at C151, located in the BTB domain, that is thought to be critical for activity [22, 23]. The Nrf2/ARE activity in a functional screen served as a model system for reaction with cellular nucleophiles and indirectly probes for cysteine reactivity. Keap1 is the most cysteine‐rich protein in the proteome and a redox sensor, which represented an ideal model system to identify novel and potentially tunable reactivity. Here, we probed chemistry and cellular consequences of a unique series of ring fusion compounds, identifying a new α,α‐*gem*‐dibromo lactam warhead that reacts with cellular thiols. The semisynthetic triterpenoid bardoxoline methyl is a covalent modifier of Keap1, and therefore a potent activator of transcription factor Nrf2, has advanced to Phase III clinical trials for chronic inflammatory conditions, demonstrating the therapeutic utility of Nrf2 activators [33, 34]. The immunomodulator and Nrf2 activator dimethyl fumarate has been approved to treat multiple sclerosis and attenuates psoriasis [35].

The aza‐oxyallyl cation was first reported by Sheehan in 1968 during work related to the synthesis of α‐lactams [36]. In 2011, Jeffrey and co‐workers reported aza‐oxyallyl cations to engage in aza‐[4+3] cycloaddition reactions with furan and cyclopentadiene (Figure 1B, formation of product **4**) [37]. In 2015 and 2016, the Jeffrey, Wu, and Liao groups independently reported regioselective aza‐oxyallyl‐mediated dearomative [3+2]‐cycloaddition reactions with simple indoles (Figure 1B, formation of product **6**) [38, 39, 40]. Wu and colleagues reported computational studies using density functional theory supporting a stepwise cycloaddition mechanism with the key C─C bond initially forming between the nucleophilic C3 position of the indole heterocycle and the aza‐oxyallyl cation before the final C─N bond forming step to product [39]. These collective efforts inspired our work to probe the potential of aza‐oxyallyl cations to react with the complex indole alkaloid ajmalicine **7** (Figure 1C) to access novel ring‐fused scaffolds to explore new chemical space targeting new pharmacological space.

## 2 Results and Discussion

### 2.1 Synthesis

**SCHEME 1:**
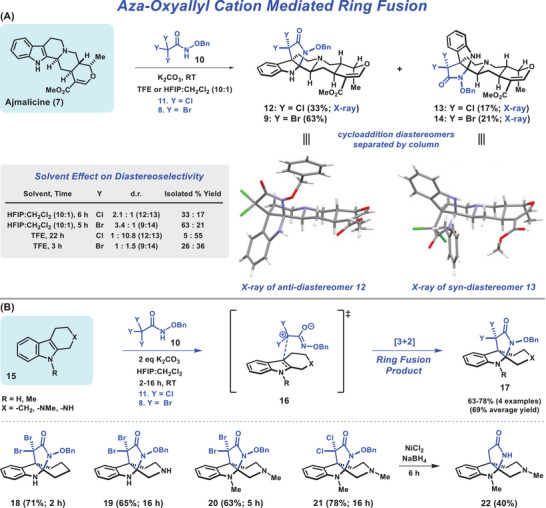
Aza‐oxyallyl cation mediated [3+2]‐cycloaddition reactions to new ring‐fused products from (A) the indole alkaloid ajmalicine **7**, and (B) tetrahydrocarbazole/tryptoline substrates **15**.

Our initial synthesis efforts were focused on ajmalicine **7**, a complex indole alkaloid that is commercially available. The first wave of experiments aimed to probe the chemoselectivity of the [3+2]‐cycloaddition reaction between an aza‐oxyallyl cation generated from **8** or **11** and ajmalicine **7**, which contains an indole heterocycle, a tertiary amine, and an electronically rich α,β‐unsaturated ester (Scheme 1A). In addition, we were curious to learn if ajmalicine's rigid, polycyclic molecular architecture bearing four stereocenters could induce any level of diastereoselectivity in the formation of desired ring fusion products (i.e., **9** and **14**).

The dearomative [3+2]‐cycloaddition between ajmalicine **7** and *N*‐(benzyloxy)‐2,2,2‐trichloroacetamide **11** proceeded smoothly using potassium carbonate as a base at room temperature in 1,1,1,3,3,3‐hexafluoroisipropanol (HFIP) and dichloromethane (10:1 HFIP:CH_2_Cl_2_) to yield ring fusion diastereomer products **12** (33% yield; isolated pure using column chromatography) and **13** (17% yield; isolated pure) after 6 h (Scheme 1A). In addition, Ajmalicine **7** and *N*‐(benzyloxy)‐2,2,2‐tribromoacetamide **8** were subjected to the same reaction conditions over 5 h to generate the desired ring fusion diastereomers **9** (63% yield; isolated pure) and **14** (21%; isolated pure) in a 84% combined yield. We found these reactions to be completely regioselective based on NMR data regarding the diagnostic ^13^C signal of the indoline carbon bearing two nitrogen atoms observed at ∼85 – 88 ppm (CDCl_3_).

X‐ray analysis of **12**, **13**, and **14** further supported our NMR findings regarding the regioselectivity of the cycloaddition reaction while enabling us to unambiguously assign the absolute stereochemistry of all four ring fusion products synthesized from ajmalicine **7** (Scheme 1A). Interestingly, we found the reaction solvent to dramatically impact the diastereoselectivity of this [3+2]‐cycloaddition reaction as HFIP:CH_2_Cl_2_ (10:1) gave 2.1:1 and 3.4:1 diastereomeric ratios (d.r.; based on crude NMR analysis; see Supporting Information) when **7** was reacted with *N*‐(benzyloxy)‐2,2,2‐trihaloacetamides **11** and **8**, respectively; however, in analogous reactions performed in trifluoroethanol (TFE) the diastereomeric ratios for this reaction were reversed to give 1:10.8 and 1:1.5 d.r. upon reacting ajmalicine with **11** and **8**. We are currently working to better understand the diastereoselectivity profile of this cycloaddition reaction with ajmalicine and will report our findings in due course. Overall, we were very encouraged that ajmalicine **7** undergoes the regioselective [3+2]‐cycloaddition with *N*‐(benzyloxy)‐2,2,2‐trihaloacetamides **8** and **11** to give new ring fusion products in 50%–84% combined yields of diastereomeric pairs (**12**/**13** and **9**/**14**).

We also performed a series of dearomative [3+2]‐cycloaddition reactions on more simplified indoles, including tetrahydrocarbazole and tryptoline derivatives (Scheme 1B; reaction of **15** to **17**). For these experiments, we used 10:1 HFIP:CH_2_Cl_2_ to synthesize ring fused analogs **18** – **21** in 69% average yield (range: 63%–78% isolated yield; reaction times: 2 – 16 h). In addition, we reduced **21** with nickel(II) chloride and sodium borohydride to give lactam **22** in 40% yield to evaluate and probe the biological activity of a fully reduced ring fusion molecule.

### 2.2 Biological Studies

**FIGURE 2:**
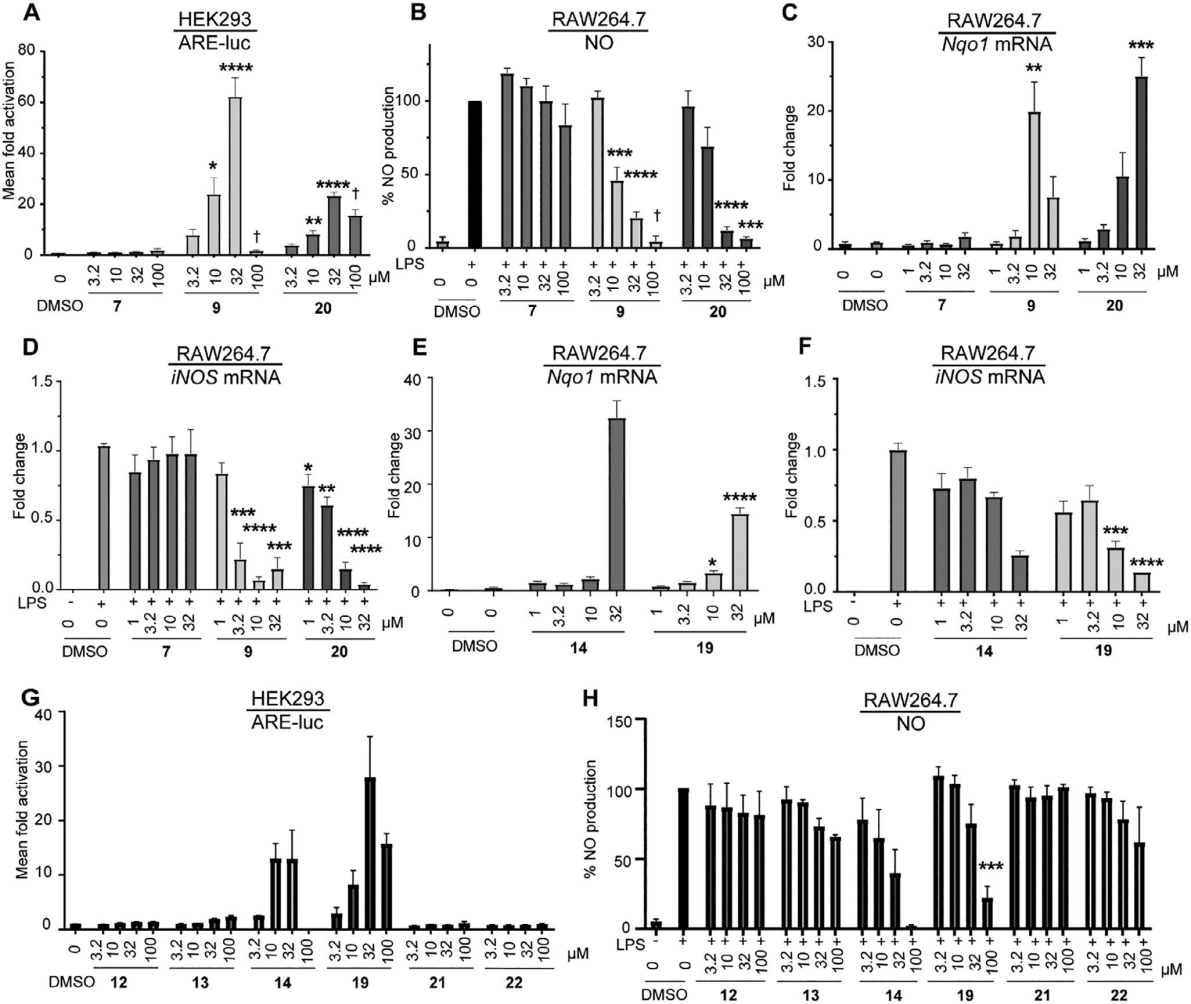
Ajmalicine **7** and ring fusion analogs **9**, **12–14**, and **19–22** were tested for their Nrf2/ARE activation and NO inhibition in HEK293‐ARE‐luc and RAW264.7 cells, respectively. (A, G) Mean fold ARE‐luc activation in HEK293 cells (24 h). (B, H) Percent NO inhibition in RAW264.7 cells (24 h). Cells were pretreated with compounds for 1 h prior to stimulation with LPS (1 µg/mL) for 24 h. (C, E) *Nqo1* mRNA expression in RAW264.7 cells (12 h). (D, F) *iNOS* mRNA expression in RAW264.7 cells (12 h). ^†^ Indicates cell death based on the viability assay performed in parallel (Figure S3). Data are presented as mean ± SEM (*n* = 3). NRF2 activation (ARE‐luciferase) and NO production (RAW264.7 assays) were analyzed by one‐way ANOVA with Dunnett's post hoc test; ^*^ *p* < 0.05, ^**^ *p* < 0.01, ^***^ *p* < 0.001, ^****^ *p* < 0.0001 versus DMSO (0.5%) or DMSO (0.5%) + LPS controls, respectively.

We performed an ARE‐luciferase reporter gene assay using stably transfected HEK293 cells for activators of the Nrf2 pathway. Through the screening of our ring distortion library from indole alkaloids (∼350 compounds at the time of this screen), we identified two ARE‐activators, compounds **9** and **20** (Figure 2), which contained the α,α‐*gem*‐dibromo lactam moiety. We validated the ARE activity in a dose‐response analysis in HEK293 cells (Figure 2A). Both compounds strongly activated the reporter at 10 µm, with maximal response at 32 µm (∼71‐fold for **9** and ∼25‐fold for **20**), without apparent induction of cytotoxicity except at 100 µm (Figure S3). In contrast, ajmalicine **7** was inactive in this assay, suggesting that the α,β‐unsaturated ester, potentially acting as a Michael acceptor, was not the pharmacophore responsible for ARE activation. This finding is further supported by the activity of **20**, which lacks an α,β‐unsaturated ester moiety. Both **9** and **20** possess the α,α‐*gem*‐dibromo lactam moiety, which was consequently hypothesized to be the pharmacophore responsible for the observed ARE activation.

There is significant cross‐talk between oxidative stress and inflammation pathways. We then tested compounds **9** and **20**, along with ajmalicine **7**, in a cell type more relevant to inflammation. RAW264.7 mouse macrophage cells were pretreated with the compounds for 1 h and then stimulated with LPS for 24 h to activate proinflammatory pathways through the TLR4 receptor. Compounds **9** and **20** each inhibited nitric oxide (NO) production as a proxy for anti‐inflammatory activity, starting at 10 µm and with over 85% efficacy at 32 µm (Figure 2B), while ajmalicine **7** was inactive, suggesting that the ARE‐luciferase activation and NO inhibition may be linked. We then assessed the gene expression of Nrf2 target gene *Nqo1* (12 h treatment), which was 9‐fold upregulated at 10 µm and 18.6‐fold at 32 µm for **20** and 29‐ and 11.5‐fold for **9** (Figure 2C). The induction of *Nqo1* expression correlated with the suppression of *iNOS* gene expression (Figure 2D), which is an NF‐κB target gene encoding the enzyme for NO synthesis, further suggesting that the two activities are coupled. This is consistent with previous findings that the induction of iNOS and NO production of Keap1‐targeting agents are lost in Nrf2^−/−^ and Keap1^−/−^ primary macrophages or embryonic fibroblasts, indicating that the anti‐inflammatory activity functions through an Nrf2/Keap1 mechanism of action [41, 42].

We then performed focused SAR studies with analogs **12–14**, **19**, **21,** and **22** to conclusively identify the pharmacophore responsible for the ARE activation observed with compounds **9** and **20**. Three analogs were evaluated using the same assay platform for each of the two hit compounds (e.g., **12–14** are analogs of **9**; **19**, **21**, **22** are analogs of **20**), distinguished by the presence of dibromo, dichloro, or methylene unit alpha to the lactam's carbonyl carbon (Figure 2G,H). The data across these assays support the α,α‐*gem*‐dibromo lactam moiety being the pharmacophore, as compounds **9**, **14**, **19**, and **20** all demonstrated similar activities. Compounds **19** and **20** activate the ARE pathway and do not possess the α,β‐conjugated system found in ajmalicine derivatives **9** and **14**. We note this structural distinction as select ARE‐activating agents operate through a thiol‐reactive α,β‐conjugated system [23, 29]. In addition, analogs **21** (α,α‐*gem*‐dichloro lactam) and **22** (methylene group alpha to the lactam) completely lost activity, demonstrating the α,α‐*gem*‐dibromo lactam moiety to be responsible for ARE activation. From the focused studies with related analogs, only compounds **14** (an analog of **9**) and **19** (an analog of **20**) were active compounds in the reporter gene assay (Figure 2G), modified endogenous transcript levels based on RT‐qPCR (Figure 2E,F), and decreased NO production (Figure 2H).

**FIGURE 3:**
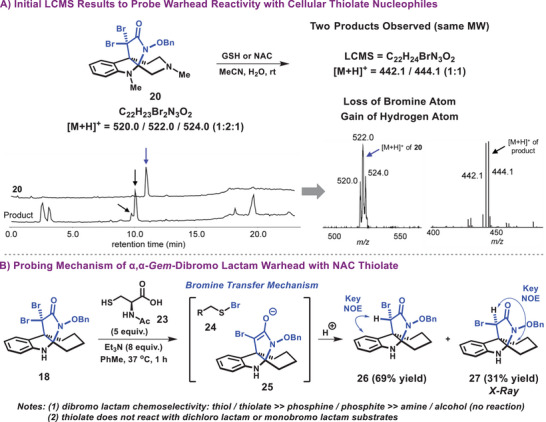
Chemical reactivity of the α,α‐*gem*‐dibromo lactam warhead. (A) Observed reaction of **20** with GSH or NAC in an aqueous environment (small scale reaction using MS for product detection). (B) Scaled up chemical reaction of **18** with NAC to characterize products **26** and **27** by NMR.

We then embarked on probing the mechanism at the molecular level. The LCMS profile of the chemical reaction of α,α‐*gem*‐dibromo lactam **20** with glutathione (GSH) and *N*‐acetyl cysteine (NAC) as model reagents to represent thiol‐containing cellular nucleophiles, indicated that **20** reacted with both thiols over the course of 2 h at room temperature. Interestingly, we observed the formation of two mono‐brominated species during this reaction (Figure 3A, LCMS findings). Next, we performed a series of scaled‐up reactions between α,α‐*gem*‐dibromo lactam **18** and select thiol nucleophiles (e.g., NAC, 1,3‐propanedithiol) to characterize products using NMR and gain mechanistic insights. During these studies, **18** was reacted with NAC using triethylamine as a base in toluene at 37°C to afford monobrominated diastereomers **26** and **27** in quantitative yield after 1 h (Figure 3B; see Table S1 for full details related to our efforts probing reaction conditions). We found this reaction to require triethylamine as a base and conclude that this transformation proceeds through a thiolate nucleophile, as the pKa of cysteine's thiol is reported to be ∼ 8.5 [43]. In addition, we note this interesting transformation can be completed in only 15 min at 37°C (Table S1, entry 2). Collectively, we propose that triethylamine deprotonates NAC's thiol to liberate a thiolate nucleophile which rapidly attacks an electrophilic bromine atom of the α,α‐*gem*‐dibromo lactam moiety to displace enolate **25** in a “bromine transfer” step followed by a final protonation step to yield diastereomers **26** and **27**. The stereochemistry of diastereomers **26** and **27** was assigned using NOE experiments in addition to the X‐ray obtained for compound **27** (see Figure 3B; see additional details in Table S1). We were interested to know the fate of the thiol nucleophile as we believed brominated thiol **24** to be highly unstable, so we performed a reaction between α,α‐*gem*‐dibromo lactam **18** and 1,3‐propanedithiol in deuterated chloroform to observe diastereomers **26** and **27** in addition to the clean formation of 1,2‐dithiolane via NMR (disulfide structure shown in Table S1, entry 3; see NMR section).

We further probed the chemoselectivity related to the bromine transfer reaction from the α,α‐*gem*‐dibromo lactam moiety of **18** using a focused panel of nucleophiles (e.g., amine, alcohol, phosphorus‐based) while also investigating α,α‐*gem*‐dichloro lactam **21** and monobrominated lactams **26** and **27** as possible substrates (Table S1). From these experiments, we found that butylamine and butanol were unable to react with α,α‐*gem*‐dibromo lactam **18** at 37°C after 24 h; however, phosphorus‐based nucleophiles (e.g., P(OEt)_3_ & triphenylphosphine) were able to transform **18** into **26** and **27,** albeit in significantly lower yields compared to thiols (Table S1). Although phosphorus‐based nucleophiles allowed for additional insights related to the bromine transfer reaction, we do not believe these particular experiments mimic cellular events but provide information on scope, generalizability, and tunability. Finally, we found thiol/thiolate nucleophiles not to react with α,α‐*gem*‐dichloro lactam **21** or monobrominated lactams **26** and **27**, demonstrating the requirement of the α,α‐*gem*‐dibromo lactam moiety for bromine transfer.

From our collective findings, we believe the α,α‐*gem*‐dibromo lactam moiety is acting as an electrophilic warhead, reacting with select cellular thiolates. It is well established that a protein's microenvironment can dramatically increase the acidity of thiols on select cysteine residues (pK_a_ values as low as 2.88) where they exist in thiolate form [44, 45]. Specifically, we believe thiolate nucleophiles attack an electrophilic bromine on the α,α‐*gem*‐dibromo lactam moiety to form a brominated‐thiol intermediate and an enolate intermediate. Under cellular assay conditions, an enolate resulting from the postulated bromine transfer would rapidly protonate in an aqueous environment to form a monobrominated lactam product. The bromine transfer process we show in Figure 3 is reminiscent of a few chemical reactions, including: (1) the initial step of the Appel reaction where triphenylphosphine attacks a bromine atom on bromoform to displace a stabilized carbanion, (2) the utilization of a titanium‐activated dibromomalonate to facilitate electrophilic bromine transfer to the alkene nucleophile of an allylic alcohol displacing a monobrominated malonate enolate [46], and (3) triphenylphosphine, hydroxide, or ionic liquid promoted debromination reactions of α‐bromoketones [47, 48, 49, 50]. Alternative reactions involving bromine transfer processes between molecular bromine and thiol‐ or thioether‐containing molecules include (1) transformation of 1,3‐propanedithiol to 1,2‐dithiolane [51] (a protocol used to confirm formation of 1,2‐dithiolane by NMR during these studies; see Table S1), and (2) formation of bis‐(3‐bromopropyl) disulfide from trimethylene sulfide [52].

**FIGURE 4:**
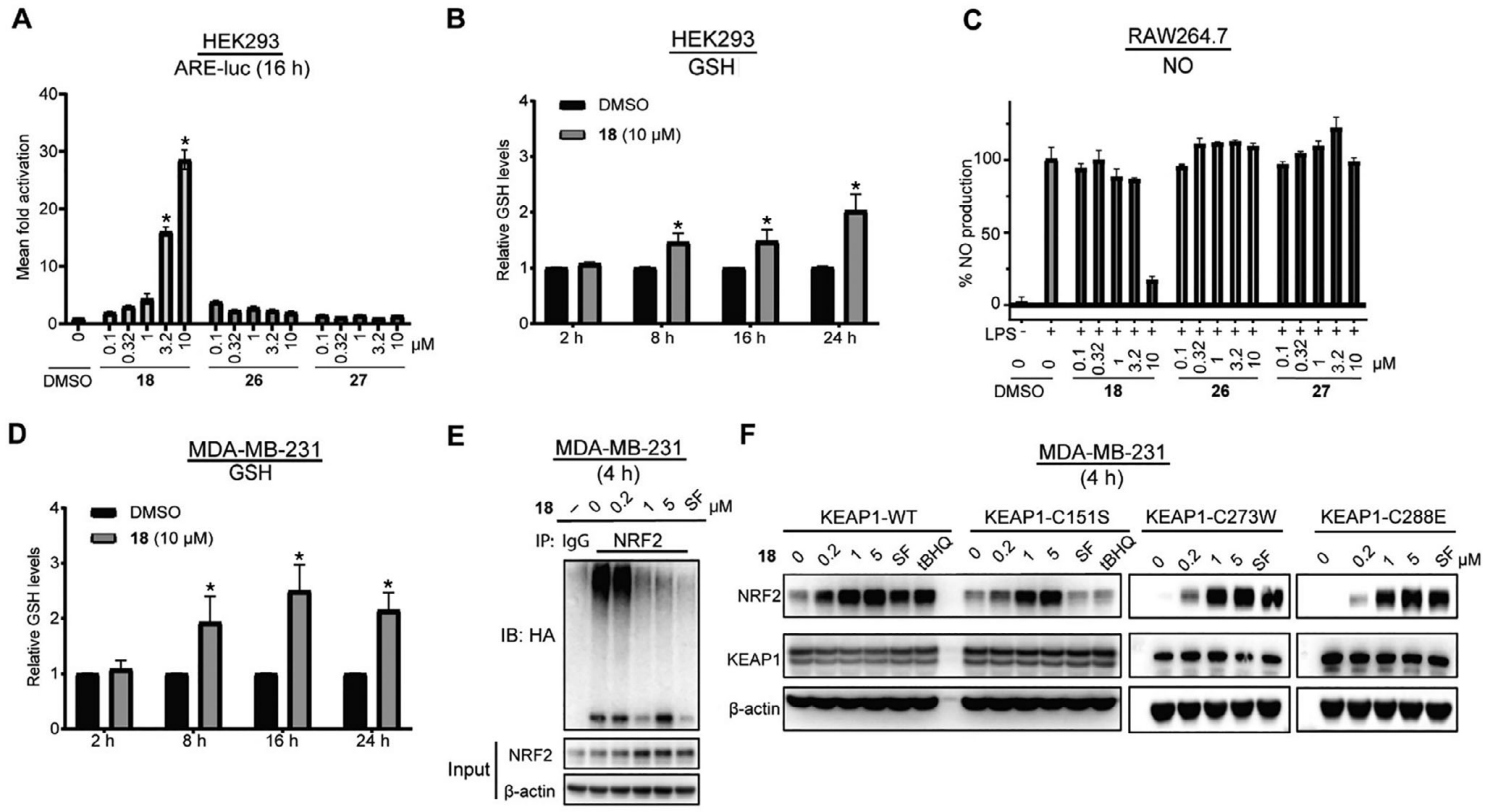
α,α‐Gem‐dibromo lactam **18** selectively activates NRF2 and increases cellular GSH. (A) Mean fold activation in HEK293‐ARE‐luc cells after 16 h treatment demonstrates that the simplified and tetrahydrocarbazole analogue of **19** and **20**, α,α‐gem‐dibromo lactam **18** is active, whereas monobrominated compounds **26** and **27** are inactive. NRF2 activation was assessed by measuring luciferase activity using the BriteLite detection reagent. Data are presented as mean fold activation ± SEM (*n* = 3). Statistical significance was determined by one‐way ANOVA followed by Dunnett's post hoc test; ^*^ *p* < 0.05 versus DMSO (0.5%) control. (B) Relative GSH levels in HEK293‐ARE‐luc cells were unchanged at 2 h but significantly increased at 8, 16, and 24 h (peak). DMSO (0.5%) served as the control. Data are presented as mean ± SEM (*n* = 3). Statistical significance was determined using a two‐tailed unpaired Student's *t*‐test (^*^ *p* < 0.05). (C) Percent NO inhibition in RAW264.7 cells indicates the inhibition in the NO production of LPS‐stimulated cells. Compound **18** showed cytotoxicity at concentrations higher than 10 µm in RAW264.7 cells (Figure S2). (D) Relative GSH levels in MDA‐MB‐231 cells were unchanged at 2 h but significantly increased at 8, 16 h (peak), and 24 h. DMSO (0.5%) served as the control. Data are presented as mean ± SEM (*n* = 3). Statistical significance was determined using a two‐tailed unpaired Student's *t*‐test (^*^p < 0.05). (E) MDA‐MB‐231 cells were transfected with expression vectors for NRF2, KEAP1, and hemagglutinin (HA)‐ubiquitin for 40 h. Transfected cells were then treated for 4 h with the indicated concentration of compound **18**, SF (1 µm), along with 10 µm MG132. NRF2 ubiquitination was assessed by immunoprecipitation of NRF2 with anti‐NRF2 antibodies, followed by immunoblot analysis with an anti‐HA antibody. (F) Increased stabilization of NRF2 upon treatment with compound **18** is independent of C151, C273, and C288 in KEAP1. MDA‐MB‐231 cells were transfected with NRF2 and either KEAP1‐WT, KEAP1‐C151S, KEAP1‐C273W, or KEAP1‐C288E plasmids (1:1 ratio). Forty hours post‐transfection, cells were treated with compounds for 4 h, and protein lysates collected for Western blot analysis. Sulforaphane (SF; 1 µm) and *tert*‐butylhydroquinone (tBHQ; 50 µm) are established ARE‐activating agents and served as positive C151‐dependent controls.

With respect to the α,α‐*gem*‐dibromo lactam warhead, we show this electrophile reacts chemoselectively with thiolate nucleophiles, and Keap1 is rich in cysteine residues, making it an ideal model system to discover and study this new molecular warhead. To further probe the potential mechanism, we evaluated the carbocyclic prototype version of **19** and **20**, α,α‐*gem*‐dibromo lactam **18,** and the corresponding monobromo lactams **26** and **27** in the ARE‐luc assay and found that α,α‐*gem*‐dibromo lactam **18** was able to activate the ARE pathway in HEK293 cells, while monobromo lactams **26** and **27** were found to be inactive (Figure 4A). These results further support our findings that the α,α‐gem‐dibromo lactam warhead selectively reacts with thiol‐based nucleophiles, linking its unique bromine transfer reactivity to its biological activity. We then assessed if the activation may be a result of GSH depletion. GSH levels were measured at non‐cytotoxic concentrations of **18** (10 µm) in a time‐dependent manner and found to not to appreciably change after 2 h but rose over time and peaked at 24 h (Figure 4B), consistent with increased GSH biosynthesis as a result of Nrf2/ARE activation and subsequent action of translated GSH biosynthetic enzymes. The ARE activation was time‐dependent and peaked after 16 h, consistent with a transcriptional mechanism (Figure S10). We then tested compounds **18**, **26,** and **27** for NO production in RAW264.7 cells (Figure 4C), which correlated with the Nrf2/ARE activity data in HEK293 cells. To further probe the mechanism and support our findings, we verified increased GSH production in another commonly used cell line for Nrf2 studies (MDA‐MB‐231, Figure 4D) and assessed the ubiquitination state of NRF2 in cells following compound treatment using an immunoprecipitation assay. We evaluated **18** alongside established ARE‐activating agents sulforaphane (SF) and *tert*‐butylhydroquinone (tBHQ; positive controls). NRF2 was immunoprecipitated with an anti‐NRF2 antibody, and ubiquitinated species were detected by immunoblotting with an anti‐HA antibody against HA‐tagged ubiquitin. Treatment of MDA‐MB‐231 breast cancer cells with compound **18** or positive control, SF, prevents ubiquitination of NRF2 (Figure 4E). In MDA‐MB‐231 breast cancer cells transfected with NRF2 and either KEAP1‐WT or one of the KEAP1 mutants (C151S, C273W, or C288E) at a 1:1 ratio, compound **18** stabilized and increased NRF2 protein levels in a dose‐dependent manner. This effect was more potent than sulforaphane and tert‐butylhydroquinone (Figure 4F, left). Unlike conventional NRF2 activators such as SF or tBHQ, which are KEAP1‐C151 dependent, compound **18** increased NRF2 protein levels independent of KEAP1 C151, C273, and C288, as activation was not compromised when these cysteine residues were mutated (Figure 4F, right).

To determine cellular downstream consequences at the global level and to potentially identify additional pathways as a proxy of the extent of other proteins targeted in the cysteome, compounds **9**, **19**, **20,** and ajmalicine **7** were subjected to RNA‐seq at 10 µm using RAW264.7 cells. At this concentration, compounds **9** and **20** had strongly induced *Nqo1* and inhibited *iNOS*, while **19** only had marginal activity (Figure 2C–F). Therefore, we expected to validate the Nrf2 pathway as one of the primary pathways for **9** and **20** but not necessarily for **19**.

**FIGURE 5:**
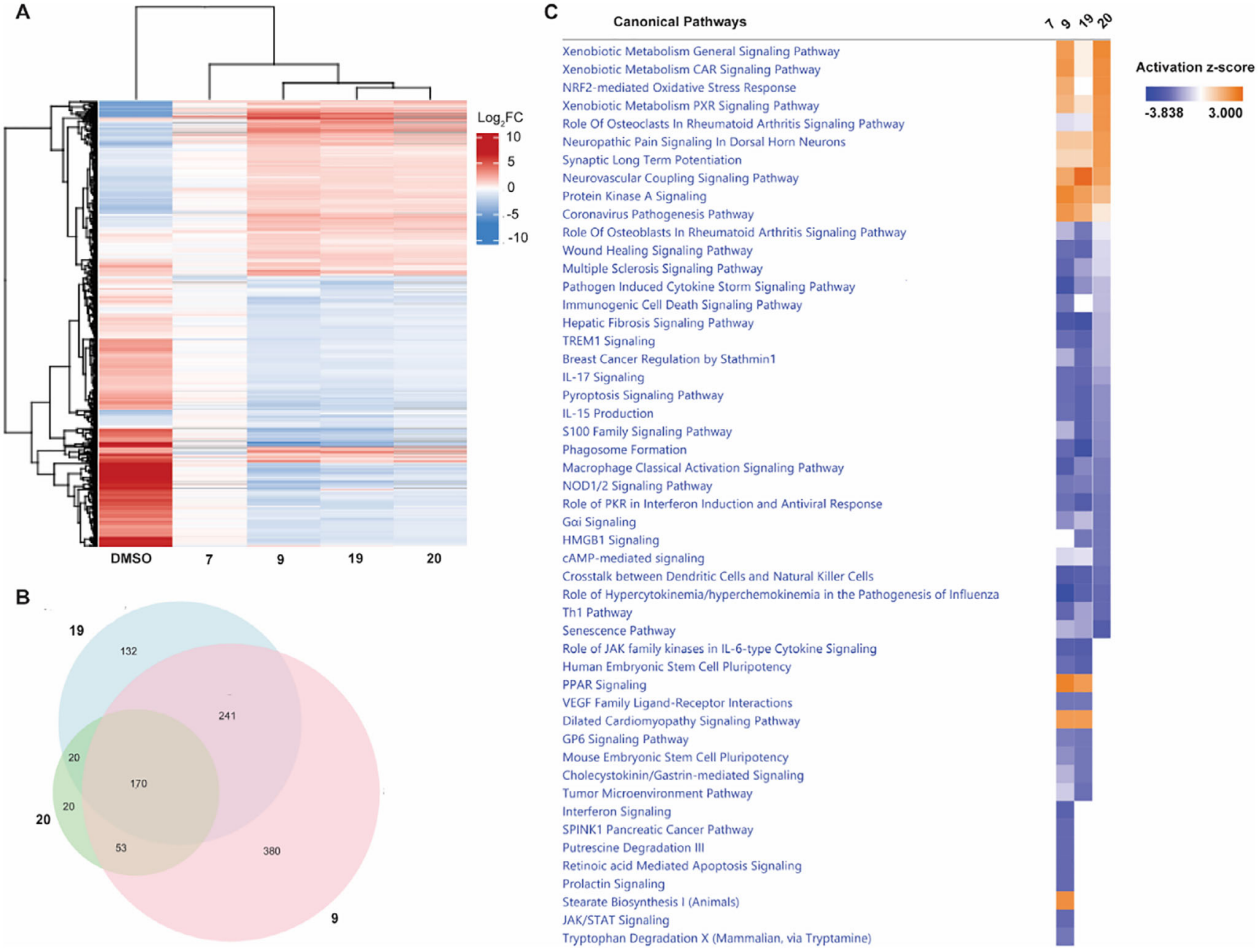
RNA‐seq data analysis. (A) Heatmap showing the effect of LPS on RAW264.7 cells treated with vehicle control in comparison to the effects of the compounds in LPS‐induced cells. (B) Venn diagram of affected genes for compounds **9**, **19**, and **20**. (C) Pathway comparison for the three prioritized active compounds. The pathways shown have a *p*‐value < 0.05. The pathways are ranked based on the z‐score for **20**.

RAW264.7 cells were exposed to the compounds for 1 h before the addition of LPS. RNA isolated 12 h later was subjected to sequencing (in triplicate). Differentially expressed genes upon RNA‐seq were evaluated based on their fold modulation, with genes showing more than 2‐fold change and a *p*‐value of < 0.05 being selected (Figure 5; Figure S4). Ajmalicine **7** only regulated 10 genes (up or down), while α,α‐*gem*‐dibromo lactam **9** regulated the most genes with 844 (378 up and 466 down), **19** regulated 563 (208 up and 355 down), and **20** regulated 263 (166 up and 97 down), as shown in Figure 5A and Figure S4. Expectedly, there was a large overlap of genes, but also sets of uniquely regulated genes (Figure 5B).

The RNA‐seq data were analyzed using the Ingenuity Pathway Analysis (IPA), generating a series of well‐established pathways, labeled as canonical pathways. Consistent with previously performed experiments, canonical pathways related to antioxidant and anti‐inflammatory pathways were identified for analogs **9** and **20,** and to a lesser extent for **19** at the concentration tested. A comparison of the canonical pathways of the compounds showed that analogs **9**, **19**, and **20** affected similar pathways. The data for analogs **9** and **20** show activation of the Nrf2 pathway, with Nrf2‐mediated oxidative stress response being one of the top activated canonical pathways, as well as the xenobiotic metabolism general signaling pathway (Figure 5C; Figures S5 and S6). Genes in the Nrf2 pathway that are up‐ and down‐ regulated by the three compounds are shown in Table S2. Analog **9** has a lower activation z score (1.6) and a higher *p*‐value for the Nrf2‐mediated oxidative stress compared to **20** (2.2). Some of the altered genes belong to the GST family, which is divided into seven subtypes and catalyze the nucleophilic attack of glutathione (GSH) on electrophilic substrates, facilitating their elimination; several of these GST genes are well‐established Nrf2 target genes. The higher effect of analog **9** on the GST family is indicative of its upregulation of the xenobiotic metabolism general signaling pathway. Analog **19** regulates some of the genes belonging to the two canonical pathways mentioned above, its overall effect has a lower fold change than that of compounds **9** and **18**, consistent with our RT‐qPCR experiments (Figure 2G,H). These findings could explain why the main canonical pathways activated by **19** are not the same as those by the other two compounds. Ajmalicine **7** has been excluded from Table S2 as it shows little to no effect on the genes listed.

The second general pathway is inflammation, which in all three datasets is shown to be inhibited by various canonical pathways. For analog **9**, the top canonical pathway is the pathogen‐induced cytokine storm signaling pathway, which is inhibited by this compound. Table S3 contains the genes mostly affected in the NF‐κB pathway, with IL6, CXCL9, and various TNF superfamily members, such as Tnfsf9, Tnfsf10, and Tnfsf14, which are downregulated, and Tnfsf13, which is upregulated. Most of the inhibited pathways by all three compounds are related to the regulation of certain interleukins (IL6, IL18, IL15, and members of the TNF superfamily), all of which are directly related to immune response and also cell proliferation.

As seen in Figure 5C, analog **9** affects more pathways compared to the other two. Analog **19** does not have Nrf2 as one of its pathways, indicating that other pathways and proteins are preferentially targeted. This is supported by the RT‐qPCR data, in which the mRNA expression change of *Nqo1* was lower in the cells treated with it compared to those treated with analog **20**. Analog **19** possesses a secondary aniline and amine while **20** contains tertiary (methylated) aniline and amine as their only differences, indicating there could be potential interactions between these molecules and their targets and that the activity is tunable. It is important to note that the RNA‐seq profile of analog **19** is more similar to that of **9**. The top diseases and functions predicted to be affected by the compounds (Tables S4–S6) also support the similarities in the profiles of analogs **9** and **19** compared to analog **20** (Figure 5C). The different profile of the three analogs indicates that the molecules can be further tuned to increase their activity and also their selectivity.

**FIGURE 6:**
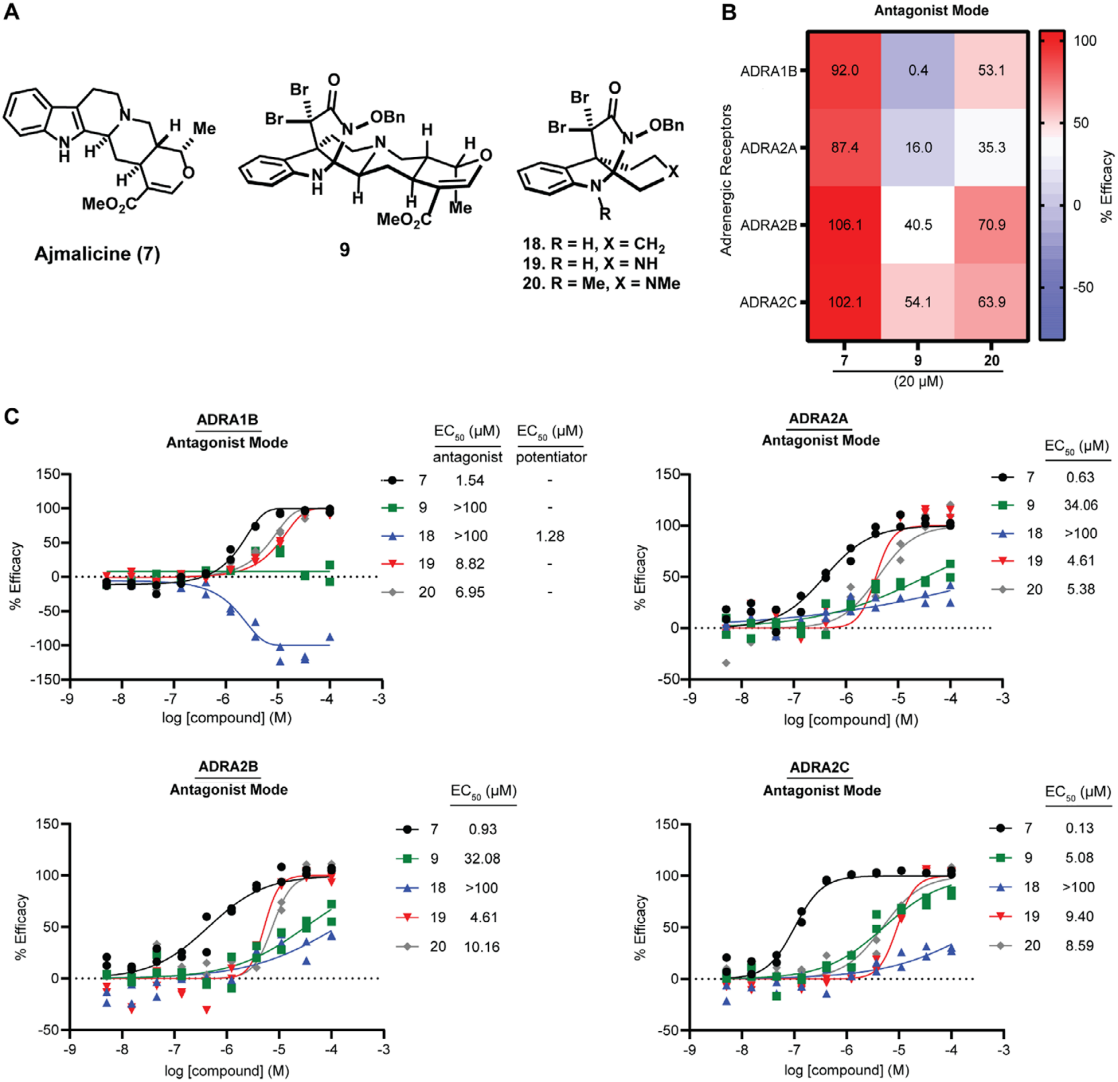
Antagonist profiling of compounds **7**, **9**, and **18**–**20** against adrenergic GPCRs. (A) Chemical structures of compounds **7**, **9**, and **18**–**20**. (B) Heatmap showing antagonist activity of compounds **7**, **9**, and **20** against ADRA1B, ADRA2A, ADRA2B, and ADRA2C in HEK293 PathHunter cells using GPCR PathHunter β‐arrestin assays. (C) Dose‐response curves (0.005–100 µm) for compounds **7**, **9**, and **18**–**20** across the four adrenergic GPCRs. EC_50_ values (µm) were calculated using nonlinear regression analysis.

Therefore, we tested a subset of compounds (Figure 6A) against a panel of GPCRs targeted by the parent compound, ajmalicine (**7**), which blocks alpha‐adrenergic receptors (ADRA), using GPCR PathHunter β‐arrestin assays. Specifically, we initially compared the profile of **7** with that of **9** and **20** at 20 µm against different receptor subtypes, including ADR1B, ADRA2A, ADRA2B, and ADRA2C, demonstrating substantial differences for all three compounds (Figure 6B). Dose‐response analyses were extended to include compounds **18** and **19**, consistently revealing that ajmalicine (**7**) was the most potent and broad‐spectrum antagonist with EC_50_ values ranging from 0.13 to 1.54 µm (Figure 6C). Compound **9** showed reduced efficacy and retained significant activity below 10 µm against ADRA2C (EC_50_ = 5.1 µm vs EC_50_ for >30 µm for the other receptors), while compounds **19** and **20** were roughly equipotent across the four receptors (EC_50_ = 4.6 to 10 µm); attenuation of activity for those two compounds compared with ajmalicine (**7**) ranged from 4.5‐fold (ADRA1B) to 72‐fold (ADRA2C) under the experimental conditions (Figure 6C). Most strikingly, compound **18** was unable to antagonize any of the four receptors. However, in antagonist mode (in the presence of agonist at EC_80_), the compound acted as an agonist potentiator and presumable positive allosteric modulator (PAM) with total selectivity for ADRA1B (EC_50_ of 1.28 µm, Figure 6C), without displaying activity in the absence of agonist (Figure S11), which warrants further investigation. Overall, this pilot data on adrenergic receptors indicates the tunability of the scaffold and that reengineering of the parent compound can lead to novel pharmacology.

At this stage, the compounds represent proof of concept for the activity and selectivity of the warhead and associated novel pharmacological profile. Microsomal stability of the new compounds is low compared with ajmalicine, indicating that the modifications negatively impacted stability (Table S7). Future endeavors would seek to improve metabolic stability accordingly.

## 3 Conclusions

In conclusion, we have discovered an α,α‐*gem*‐dibromo lactam warhead that activates the Keap1/Nrf2 pathway in a distinct way and demonstrates anti‐inflammatory activities. These findings resulted from the chemical synthesis of new ring fusion scaffolds, which were accessed through aza‐oxyallyl cation‐mediated [3+2]‐cycloaddition reactions of ajmalicine **7** and polycyclic indoles. RNA‐seq and Ingenuity Pathway Analysis illuminated detailed activity profiles for α,α‐*gem*‐dibromo lactams **9**, **19**, and **20** to show these small molecules activated the Nrf2 pathway, including several downstream target genes (e.g., NQO1, GST). In addition, these compounds suppressed NF‐kB‐mediated inflammatory signaling linked to cancer. We show NAC and other thiolates selectively react with the α,α‐*gem*‐dibromo lactam warhead to transfer bromine, linking unique chemistry to these biological discoveries. Structure‐activity relationship (SAR) studies demonstrated the requirement of the α,α‐*gem*‐dibromo lactam moiety as the α,α‐*gem*‐dichloro lactam is unable to activate the Keap1/Nrf2 pathway or react with thiol nucleophiles. Mechanistic studies at the target level suggested a distinct cysteine dependency‐profile and mode of Nrf2 activation, although additional studies are needed. Further investigations are underway and aim to leverage this new α,α‐*gem*‐dibromo warhead to explore and probe its fundamental activities related to other areas of biology and disease treatment. Overall, we have discovered a biologically interesting chemotype and reactivity profile that could lead to the creation of new electrophiles for transient covalent protein modification with distinct selectivity patterns, adding to the arsenal of existing targeting moieties and probes. The cycloaddition products modulated the pharmacological profile of the parent indole, ajmalicine, at the level of GPCRs, attenuating and modulating the selectivity fingerprint against adrenergic receptors.

## 4 Experimental Section

Regarding biological studies, cell origin and cell culture conditions include: RAW264.7 cells (American Type Culture Collection, ATCC) and HEK293‐ARE‐luc cells (Signosis) were cultured in Dulbecco's modified Eagle's medium (DMEM) supplemented with 10% fetal bovine serum at 37°C humidified air, and 5% CO_2_. There were no animals used in this study.

### 4.1 NO Inhibition and Cell Viability Assays (RAW264.7 cells)

RAW264.7 cells (ATCC) were cultured in Dulbecco's modified Eagle's medium (DMEM) supplemented with 10% fetal bovine serum at 37°C humidified air, and 5% CO_2_. Cells were seeded (20 000/well) in 96‐well clear‐bottom plates, allowed to attach for 24 h before being treated with the compounds or the solvent control (0.5% DMSO) for 1 h, followed by the addition of LPS at 1 µg/mL. Non‐stimulated cells were tested at the same time. The production of nitric oxide (NO) was calculated after 24 h by measuring the nitrite concentration, an oxidative product of NO, in the cell supernatant. 50 µL of the supernatant were then mixed with the Griess reagent using the manufacturer's protocol (Promega), and the absorbance was measured at 540 nm. A calibration curve was generated using a fresh nitrite solution standard, to calculate the concentration of NO. The cell viability was measured under the same seeding density and time points, using MTT dye following the manufacturer's protocol (Promega).

### 4.2 ARE‐Luciferase and Cell Viability Assays (HEK293‐ARE‐luc cells)

HEK293‐ARE‐luc cells were cultured in Dulbecco's modified Eagle's medium (DMEM) supplemented with 10% fetal bovine serum at 37°C humidified air, and 5% CO_2_. Cells were seeded (10 000/well) in 96‐well plates, allowed to attach overnight before being treated with the compounds or the solvent control (0.5% DMSO) for 24 h. For time course experiments, treatment times were 2, 8, 16, or 24 h. The cell viability was measured under the same seeding density and time points, using MTT dye following the manufacturer's protocol (Promega). The ARE activation was measured by seeding HEK293‐ARE‐luc cells (10 000/well) in white 96‐well plates and allowed to attach overnight before treatment with compounds or the solvent control (0.5% DMSO). 100 µL of Britelite was added, and the plates were incubated for 5 min in the dark and luminescence measured using the Envision XCITE (PerkinElmer). Relative fold activation was calculated by normalizing the luminescence of treated samples to that of the solvent control.

### 4.3 LC‐Mass Spectrometry Analysis Protocol

Initially, 100 µg of analog **20** was dissolved in 500 µL of acetonitrile (ACN). Then, 50 equivalents of glutathione (GSH) or *N*‐acetyl cysteine (NAC) were dissolved in a 2:1 water:ACN solution and added to the compound solution. The samples were then stirred at room temperature and subjected to LCMS (Kinetex C18, 0.5 mL/min) at various time points with a water:ACN gradient (0–2 min 10%, 2–16 min 10%–95%, 16–21 min 95%, 21–25 min 10%). This analysis was performed in 20 min intervals for the duration of 2 h to monitor the reaction.

### 4.4 Glutathione Assays

Intracellular GSH levels were quantified using the Sigma–Aldrich Glutathione GSH/GSSG Assay Kit (MAK440) following the manufacturer's instructions. Briefly, HEK293 ARE‐luc cells or MDA‐MB‐231 cells (1 000 000 cells/well) were seeded into six‐well plates and incubated overnight at 37°C. Cells were harvested at 2, 8, 16, and 24 h after treatment with 10 µm of compound **18**. Cells were then washed with ice‐cold PBS and lysed in 50 mm phosphate buffer (pH 7.0) containing 1 mm EDTA. Lysates were centrifuged at 14 000 × g at 4°C for 5 min for GSSG measurement, and 15 min for GSH measurement, and the supernatant was collected. For GSSG determination, reduced GSH was derivatized using the kit's scavenger reagent, whereas total GSH was measured without derivatization. Samples and GSH standards were incubated with the kit's working reagent containing DTNB and glutathione reductase in a 96‐well plate at room temperature for 10 min, and absorbance was read at 412 nm using a microplate reader. Glutathione concentrations were calculated from standard curves, and GSSG levels were not detected at this assay format.

### 4.5 RNA Isolation and RT‐qPCR

RAW264.7 cells were seeded (2.5 × 10^5/well) in 6‐well plates and treated with different concentrations of the compounds or vehicle control for 1 h prior to treatment with LPS at 1 µg/mL and incubated for 12 h. Total RNA was extracted using the RNAeasy Mini Kit, and RT‐PCR analysis was performed to detect the expression of *iNOS*, nqo1, and β‐actin (internal standard) on aliquots of cDNA prepared from it. Starting with 500 ng of total RNA, it was reverse‐transcribed to cDNA using SuperScript II Reverse Transcriptase (Invitrogen, Carlsbad, CA, USA) and oligo (dT) (Invitrogen). The cDNA samples were used as templates for TaqMan gene expression assays (Applied Biosystems, Waltham, MA, USA). The qPCR analysis was performed in triplicate at 25 µL total volume (12.5 µL of TaqMan 2 × universal master mix, 1.25 µL of a 20 × TaqMan gene expression assay probe, 1 µL of cDNA, and 10.25 µL of RNase‐free sterile water) to detect the expression of *iNOS* (Mm00440502_m1, Applied Biosystems), nqo1 (Mm01253561_m1, Applied Biosystems) and β‐actin (internal standard, 4352663, Applied Biosystems). The qPCR method was as follows: 50°C for 2 min, 95°C for 10 min, and 40 cycles of 95°C for 15 s and 60°C for 1 min.

### 4.6 RNA Extraction and Illumina Sequencing Library Construction

RNA extraction is performed with 18 RNA samples were measured by the QUBIT fluorescent method (Invitrogen) and Agilent Bioanalyzer. An amount of 250 ng of high quality of total RNA with RIN of 7 or higher was used for library construction using the reagents provided in the NEBNext Poly(A) mRNA Magnetic Isolation Module (New England Biolabs, catalog # E7490) and the NEBNext Ultra II Directional RNA Library Prep Kit (New England Biolabs, catalog #E7760) according to the manufacturer's user guide. Briefly, 200 ng of total RNA was used for mRNA isolated using the NEBNext Poly(A) mRNA Magnetic Isolation Module (New England Biolabs, catalog # E7490). Then, the poly A‐enriched RNA was fragmented in NEBNext First Strand Synthesis Buffer via incubation at 94°C for the desired time. This step was followed by first‐strand cDNA synthesis using reverse transcriptase and random hexamer primer. Synthesis of ds cDNA is done using the second‐strand master mix provided in the kit, followed by end‐repair and dA‐tailing. At this point, Illumina adaptors are ligated to the sample. Finally, the library was amplified, and followed by purification with AMPure beads (Beckman Coulter, catalog # A63881). The library size and mass were assessed by analysis in the Agilent TapeStation using a High Sensitivity DNA1000 Screen Tape. Typically, a 250–900 library peak is observed with the highest peak at ∼420 bp. Barcoded libraries were pooled equimolarly for sequencing simultaneously for NavaSeq 6000 S4 2 × 150 cycles run as described below. RNA‐seq library performed at UF ICBR Gene Expression Core (https://biotech.ufl.edu/gene‐expression‐genotyping/, RRID:SCR_019145).

### 4.7 Illumina NovaSeq6000 Sequencing

Normalized libraries were submitted to the “Free Adapter Blocking Reagent” protocol (FAB, Cat# 20024145) in order to minimize the presence of adaptor‐dimers and index hopping rates. The library pool was diluted to 0.8 nm and sequenced on one S4 flow cell lane (2 × 150 cycles) of the Illumina NovaSeq6000. The instrument's computer utilized the NovaSeq Control Software v1.6. Cluster and SBS consumables were v1.5. The final loading concentration of the library was 120 pm with 1% PhiX spike‐in control. One lane generated 2.5 – 3 billion paired‐end reads (∼950 Gb) with an average Q30% ≥ 92.5% and Cluster PF = 85.4%. FastQ files were generated using the BCL2fastQ function in the Illumina BaseSpace portal. The Illumina NovaSeq 6000 was used to sequence the libraries for 2 × 150 cycles. Sequencing was performed at the ICBR NextGen Sequencing (https://biotech.ufl.edu/next‐gen‐dna/, RRID:SCR_019152).

### 4.8 RNA Sequencing Analysis

The RNA seq data was analyzed using Ingenuity Pathway Analysis (IPA). The compounds were added in triplicate at 10 µm. For the RNA seq analysis, the samples were compared to the DMSO (+) LPS control as well as the DMSO (‐) LPS control. Using the IPA software, the analysis was done using a cutoff of 2‐fold change in the gene expression and *p*‐value < 0.05.

### 4.9 KEAP1 Cysteine Dependency Analysis

MDA‐MB‐231 cells were seeded in 6‐well plates and transfected with 1 µg of NRF2 expression plasmid along with either WT or mutant KEAP1 plasmid at a 1:1 ratio (relative to NRF2). 40 h post‐transfection, the cells were treated with the indicated compounds. Compound **18** was applied at concentrations of 0.2, 1, and 5 µm, while SF and tBHQ were applied at 1 and 50 µm, respectively. Cell lysates were harvested 4 h post‐treatment and analyzed by immunoblotting.

### 4.10 Ubiquitination Assay

MDA‐MB‐231 cells were seeded in 35‐mm dishes and co‐transfected with expression vectors for HA‐Ub, KEAP1, and NRF2 at a 1:1:1 ratio. After 24 h, cells were treated with the indicated compounds along with 10 µm MG132 for 4 h to inhibit proteasomal degradation. Cells were then harvested in lysis buffer containing 1% SDS, 150 mm NaCl, 10 mm Tris‐HCl (pH 8.0), and 1 mm DTT. Immediately after harvesting, lysates were boiled in preheated water to denature proteins. The boiled lysates were then diluted fivefold in the same buffer without SDS and incubated with anti‐NRF2 antibody (Santa Cruz, H300) for immunoprecipitation. Immunoprecipitated proteins were analyzed by immunoblotting with an anti‐HA antibody (rat monoclonal antibody, clone 3F10, Roche, Cat# 11867423001), followed by an HRP‐conjugated goat anti‐rat IgG secondary antibody (Cell Signaling Technology, Cat# 7077) to detect ubiquitinated NRF2. A small aliquot of the total cell lysates was used for the input control.

### 4.11 GPCR PathHunter β‐Arrestin Assays

Global GPCR profiling experiments and dose response studies for selected targets (hits) against active compounds and their analogs were carried out by Eurofins/Discovery employing the assay developed by DiscoverX (Eurofins, DiscoverX, Fremont, CA). Compounds **7**, **9,** and **20** were profiled at 20 µm against the gpcrMAX GPCR assay panel using the PathHunter β‐arrestin assay. The assay uses enzyme fragment complementation with β‐galactosidase as a functional reporter, in agonist and antagonist modes. Briefly, the PathHunter cell lines are expanded, seeded at a total volume of 20 µL in 384‐well white microplates, and incubated at 37°C for the appropriate time prior to testing. For agonist mode, cells are incubated for at least 120 min with the compounds to induce a response with a final assay vehicle concentration at 1%. For antagonist mode, cells are preincubated with the antagonist and challenged by the agonist (EC_80_ concentration). Plates are read with a PerkinElmer Envision TM instrument for chemiluminescence detection. The adrenergic receptor targets ADRA1B, ADRA2A, ADRA2B, and ADRA2C were used to further evaluate the compounds **7**, **9**, **20,** alongside compounds **18** and **19** in dose response experiments in antagonist mode. For agonist determination of compound **18,** cells were incubated with the sample at different concentrations to induce a response. Data was calculated as % inhibition or activation for each target using the following formulae: % activity = 100% x (mean RLU of test sample – mean RLU of vehicle control) / (mean MAX control ligand – mean RLU of vehicle control); % inhibition = 100% x (1‐(mean RLU of test sample – mean RLU of vehicle control) / (mean RLU of EC_80_ control – mean RLU of vehicle control)). All data was plotted using GraphPad Prism software.

### 4.12 Hepatic Microsomal Stability

Microsome stability was evaluated by incubating 1 µm test compound with 0.5 mg/mL hepatic microsomes in 100 mm KPi, pH 7.4. The reaction was initiated by adding NADPH (1 mm final concentration). Aliquots were removed at 0, 5, 10, 20, 40, and 60 min and added to acetonitrile (5X, v:v) to stop the reaction and precipitate the protein. At the end of the assay, the samples were centrifuged through a Millipore Multiscreen Solvinter 0.45 micron low‐binding PTFE hydrophilic filter plate and analyzed by LC‐MS/MS. Data were log‐transformed and represented as half‐life.

### 4.13 Statistical Analysis

Data are presented as mean ± SEM of three independent experiments (*n* = 3). NRF2 activation (ARE‐luciferase) and NO production (RAW264.7 assays) were analyzed by one‐way ANOVA with Dunnett's post hoc test, comparing treated groups to DMSO or DMSO + LPS controls, respectively (^*^ *p* < 0.05, ^**^ *p* < 0.01, ^***^ *p* < 0.001, ^****^ *p* < 0.0001). GSH measurements in compound **18**‐treated cells were assessed by using a two‐tailed unpaired Student's *t*‐test, with ^*^p < 0.05 considered statistically significant. All analyses were performed using GraphPad Prism 10.6.1. EC_50_ values and their 95% confidence intervals for the antagonist profiling against adrenergic GPCRs were calculated using nonlinear regression analysis performed with GraphPad Prism 10.6.1.

## Conflicts of Interest

The authors declare no conflicts of interest.

## Supporting information




**Supporting File**: advs75277‐sup‐0001‐SuppMat.pdf.

## Data Availability

The data that support the findings of this study are available in the Supporting Information document associated with this article. The RNA‐seq data has been submitted to the NCBI, and the GEO number is GSE297104 (https://www.ncbi.nlm.nih.gov/geo/). Crystallographic data has been deposited with the Cambridge Crystallographic Data Center (CCDC) and can be obtained at http://www.ccdc.cam.ac.uk/.
