# Supplementary material for: Natural Products Inspired Scaffold Diversification Leads to Unnatural Molecular Warhead and Covalent Strategy to Modulating Protein Function through Electrophilic Bromine Transfer
Source: Adv Sci (Weinh). 2026 Apr 30;13(42):e12849. doi: 10.1002/advs.202512849 (PMC13334971; doi:10.1002/advs.202512849)

Natural Products Inspired Scaffold Diversification Leads to Unnatural Molecular Warhead and Covalent Strategy to Modulating Protein Function Through Electrophilic Bromine Transfer

Beau R. Brummel[a†], Sofia Kokkaliari[a†], Qiwen Gao[b], Jinjing Chen[c], Larissa Costa de Almeida[a], Srinivasarao Tenneti[a], Derek A. Leas[a], Eli Levit[a,b], Lewis S. Alexander[c,d], Khalil A. Abboud[e], Pingrong Wei[f], Michael D. Cameron[g], James R. Rocca[a,h], Ranjala Ratnayake[a,i], Donna D. Zhang[c,i], Hendrik Luesch[a,i,j]*, Robert W. Huigens III[a,b,f,k]*

[a]Department of Medicinal Chemistry and Center for Natural Products Drug Discovery and Development (CNPD3), College of Pharmacy, University of Florida, Gainesville, Florida 32610, United States. [b]Department of Pharmaceutical & Biomedical Sciences, College of Pharmacy, University of Georgia, Athens, Georgia 30602, United States. [c]Department of Molecular Medicine, Center for Inflammation Science and Systems Medicine, UF Scripps Institute for Biomedical Innovation and Technology, Jupiter, Florida 33458, United States. [d]Department of Pharmacology and Therapeutics, College of Medicine, University of Florida, Gainesville, Florida 32610, United States. [e]Department of Chemistry, University of Florida, Gainesville, Florida 32610, United States. [f]Department of Chemistry, Franklin College of Arts and Sciences, University of Georgia, Athens, Georgia 30602, United States. [g]The Herbert Wertheim UF Scripps Institute for Biomedical Innovation and Technology, Jupiter, FL 33458, United States. [h]McKnight Brain Institute, University of Florida, Gainesville, Florida 32610, United States. [i]University of Florida Health Cancer Center, University of Florida, Gainesville, Florida 32610, United States. [j]Program in Cancer and Stem Cell Biology, Duke-NUS Medical School, Singapore, 169857, Singapore. [k]Department of Infectious Diseases, College of Veterinary Medicine, University of Georgia, Athens, Georgia 30602, United States.

[†]authors contributed equally to this work

*Corresponding Authors: Robert.Huigens@uga.edu, luesch@cop.ufl.edu

Supporting Information

Table of Contents

1.) General Information. S3

2.) Figure S1. Mechanism for Aza-Oxallyl-Mediated [3+2]-Cycloaddition of Indoles. S4

3.) Figure S2. Chemical Structures of New Ring Fusion Compounds. S5

4.) Figure S3. Cell Viability in HEK293-ARE Cells and RAW264.7 Cells. S6

5.) Figure S4. Volcano Plots of Up- and Down-Regulated Genes. S7

6.) Figure S5. Nrf2 Mediated Oxidative Stress Pathway for 20. S9

7.) Figure S6. Xenobiotic Metabolism General Signaling Pathway for 20. S10

8.) Figure S7. Structure-Activity Relationship Insights for New ARE-Activating Agents. S11

9.) Figure S8. Mechanistic Pathway for Bromine Transfer Reaction. S12

10.) Figure S9. Reacting 1,3-Propanedithiol with Molecular Bromine, NBS, or Compound 18. S13

11.) Figure S10. ARE-luc Activation Time Course Study with Compound 18. S14

12.) Figure S11. Pharmacological Characterization of Compound 18 at the ADRA1B Receptor. S15

13.) Table S1. Investigating the Scope of the Bromine Transfer Reaction. S16

14.) Table S2. Fold Change of Genes in the Nrf2-Keap1 Canonical Pathways. S17

15.) Table S3. Log2 Fold Change of Genes in the NF-κB Pathway by 9, 19, and 20. S18

16.) Table S4. Top Diseases and Functions for 9. S20

17.) Table S5. Top Diseases and Functions for 19. S21

18.) Table S6. Top Diseases and Functions for 20. S22

19.) Table S7. Hepatic Microsomal Stability Results. S23

20.) Procedures for Synthesis and Characterization Data. S24

21.) X-Ray Protocols & Data. S36

22.) References. S49

23.) NMR Spectra. S50

1.) General Information.

All chemical reactions were carried out under an atmosphere of argon unless otherwise specified. Chemical reagents were purchased from commercial sources and used without further purification. Anhydrous solvents were transferred via syringe to flame-dried glassware, which was cooled under a stream of dry argon. Analytical thin layer chromatography (TLC) was performed using 250 µm Silica Gel 60 F254 pre-coated plates (EMD Chemicals Inc.). Flash column chromatography was performed using 230-400 Mesh 60Å Silica Gel (Sorbent Technologies). Ajmalicine was purchased from Chemodex LTD (≥ 98% purity).

NMR experiments were recorded on the following instruments: Varian Unity spectrometer (400 MHz for ^{1}H NMR), Bruker Avance II spectrometer (600 MHz for ^{1}H NMR; 151 MHz for ^{13}C NMR), or Agilent Systems VNMRS spectrometer (600 MHz for ^{1}H NMR; 151 MHz for ^{13}C NMR). All spectra are presented using MestReNova 11.0 (Mnova) software and are displayed without the use of the signal suppression function. Spectra were obtained in $CDCl_3$ as an NMR solvent (reference peak information: ^{1}H NMR: 7.26 ppm; ^{13}C NMR: 77.16 ppm). NMR samples where the respective solvent peaks were buried in the sample signals referenced TMS at 0.00 ppm for ^{1}H NMR experiments. NMR experiments were performed at room temperature unless otherwise indicated. Chemical shift values (δ) are reported in parts per million (ppm) for all ^{1}H NMR and ^{13}C NMR spectra. ^{1}H NMR multiplicities are reported as: s = singlet, d = doublet, t = triplet, q = quartet, m = multiplet, br = broad, appt. = apparent.

Melting points (MP) were obtained on a Mel-Temp II capillary melting point apparatus and were uncorrected. High-resolution mass spectra were obtained from the Mass Spectrometry Facility in the Chemistry Department at the University of Florida. X-Ray crystallographic data were obtained from the Chemistry Department at the University of Florida and the Chemistry Department at the University of Georgia.

2.) Figure S1. Mechanism for Aza-Oxallyl-Mediated [3+2]-Cycloaddition of Indoles.

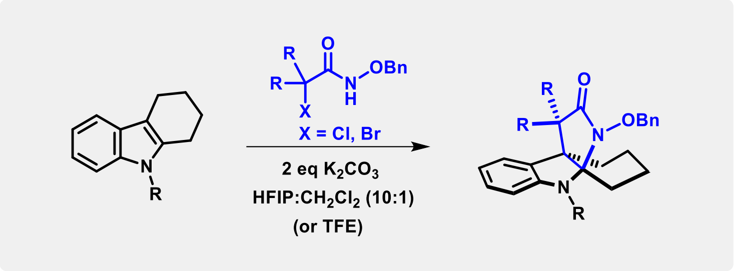

Mechanistic Pathway.

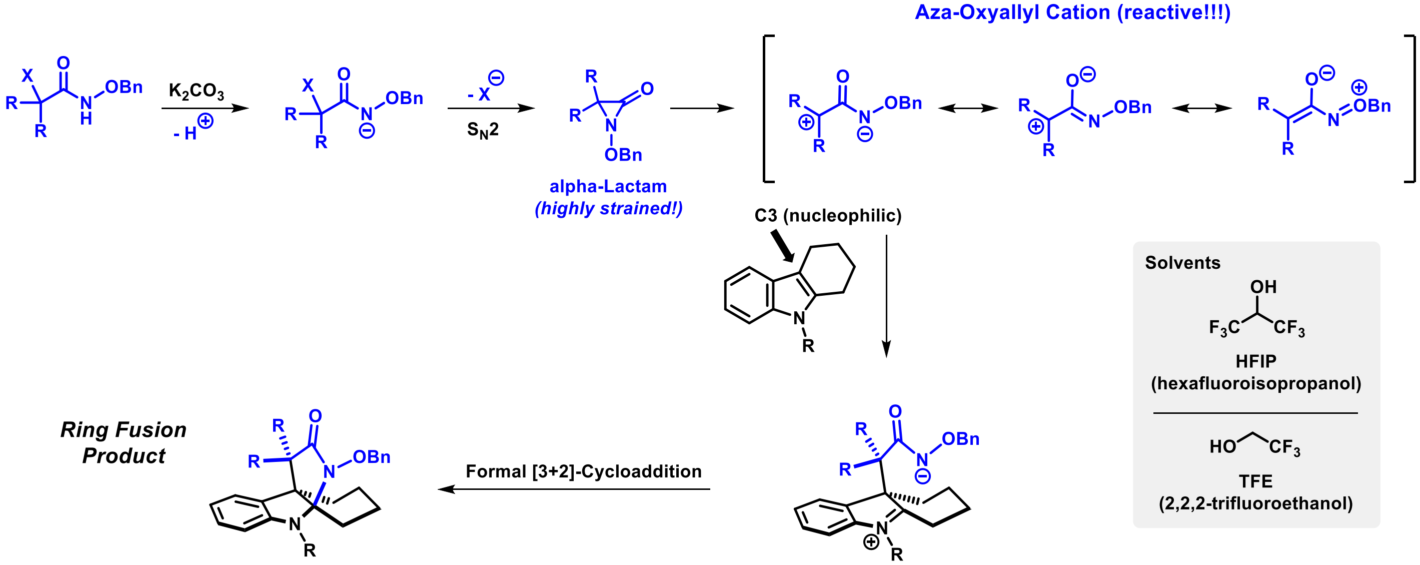

3.) Figure S2. Chemical Structures of New Ring Fusion Compounds.

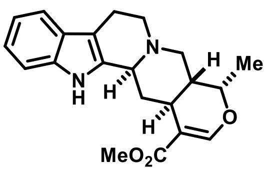

Ajmalicine (7)

- No ARE Activation
- No Nitric Oxide Inhibition
- RNA-seq (acquired)
- Angatonist profiling against adrenergic GPCRs

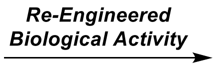

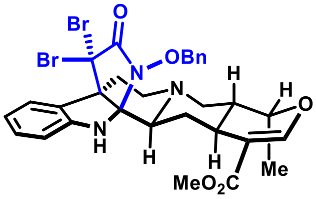

9

- ARE Activation
- Nitric Oxide Inhibition
- RNA-seq (explored activities)
- Angatonist profiling against adrenergic GPCRs

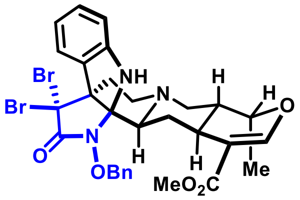

14

- ARE Activation
- Nitric Oxide Inhibition

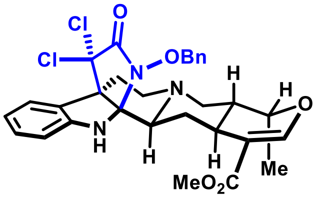

12

- No ARE Activation
- No Nitric Oxide Inhibition

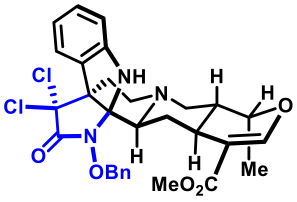

13

- No ARE Activation
- No Nitric Oxide Inhibition

Simplified Dibromolactam Probes

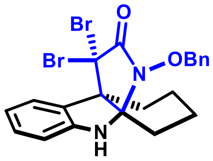

18

- ARE Activation
- Nitric Oxide Inhibition
- Bromine Transfer with Thiols (scaled up reaction, NMR studies)
- Prevents ubiquitination of NRF2
- Stabilized NRF2 independent of C151 in KEAP1 (MDA-MB-213 cells)
- Activates NRF2 while increasing cellular GSH levels
- Angatonist profiling against adrenergic GPCRs
- Pharmacological characterization at the ADRA1B receptor

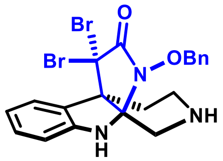

19

- ARE Activation
- Nitric Oxide Inhibition
- RNA-seq (explored activities)
- Angatonist profiling against adrenergic GPCRs

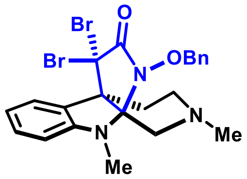

20

- ARE Activation
- Nitric Oxide Inhibition
- RNA-seq (explored activities)
- GSH or NAC Loss of Bromine (initial LCMS experiments)
- Angatonist profiling against adrenergic GPCRs

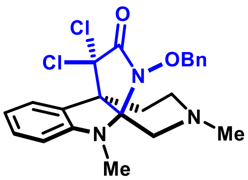

21

- No ARE Activation
- No Nitric Oxide Inhibition
- Does Not React with NAC

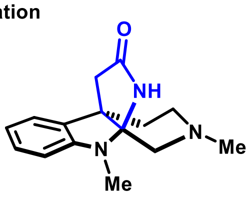

22

- No ARE Activation
- No Nitric Oxide Inhibition

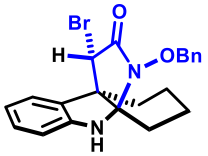

26

- No ARE Activation
- No Nitric Oxide Inhibition
- Does Not React with NAC

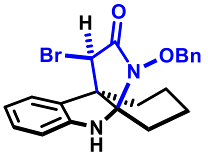

27

- No ARE Activation
- No Nitric Oxide Inhibition
- Does Not React with NAC

4.) Figure S3. Cell Viability in HEK293-ARE Cells and RAW264.7 Cells.

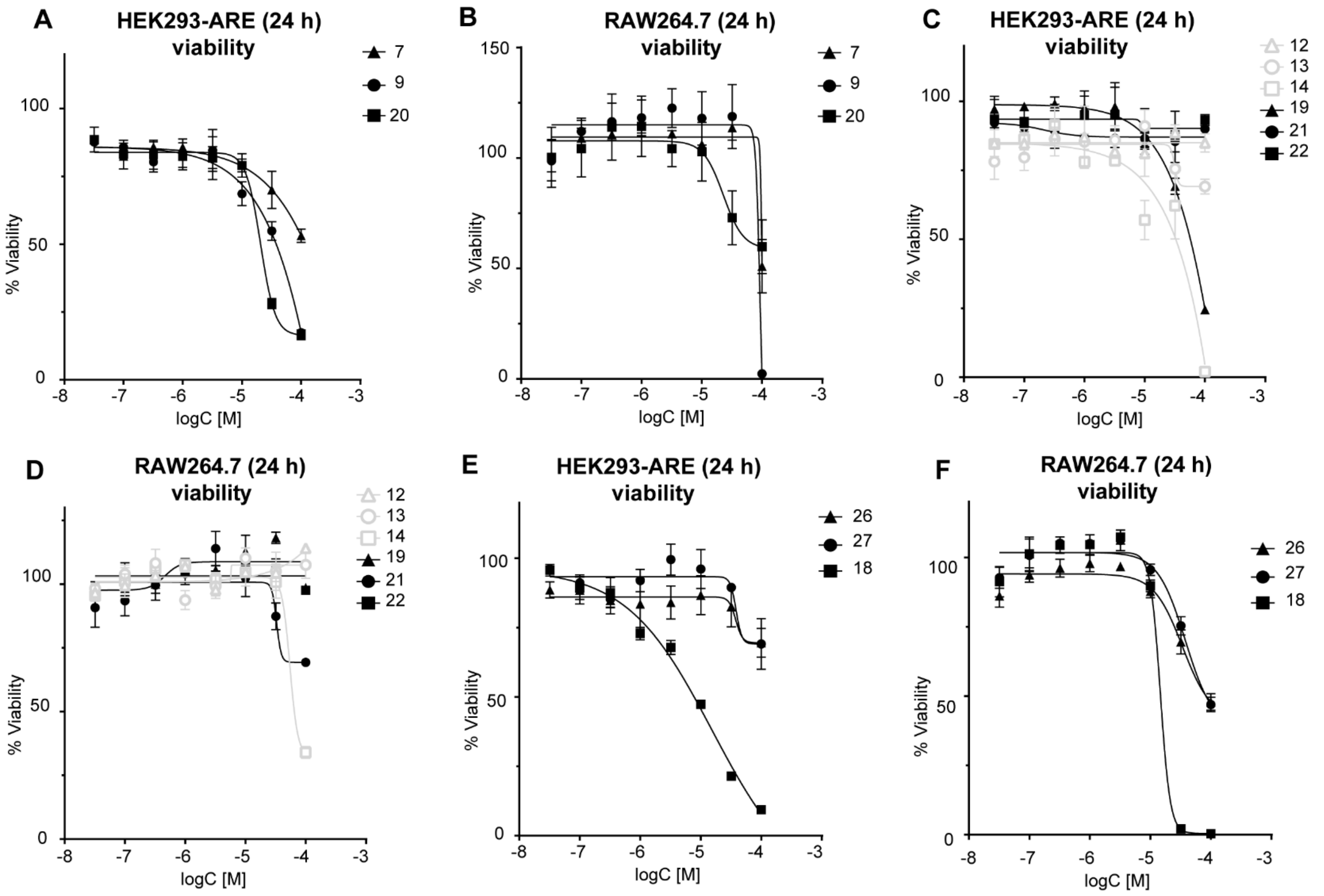

Cell viability in A, C, E) HEK293-ARE cells, and B, D, F) RAW264.7 cells.

5.) Figure S4. Volcano Plots of Up- and Down-Regulated Genes.

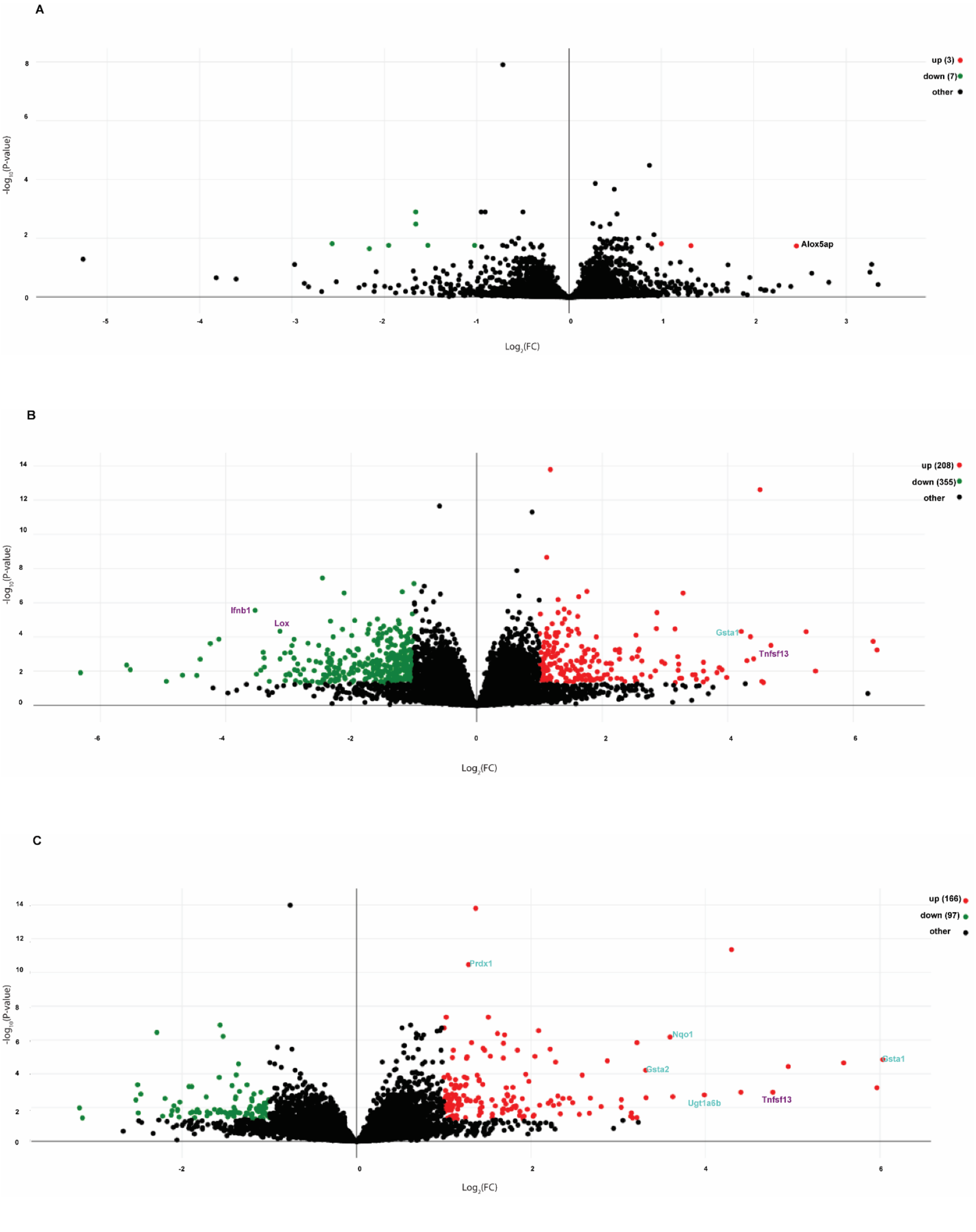

5.) Figure S4. Volcano Plots of Up- and Down-Regulated Genes. (continued)

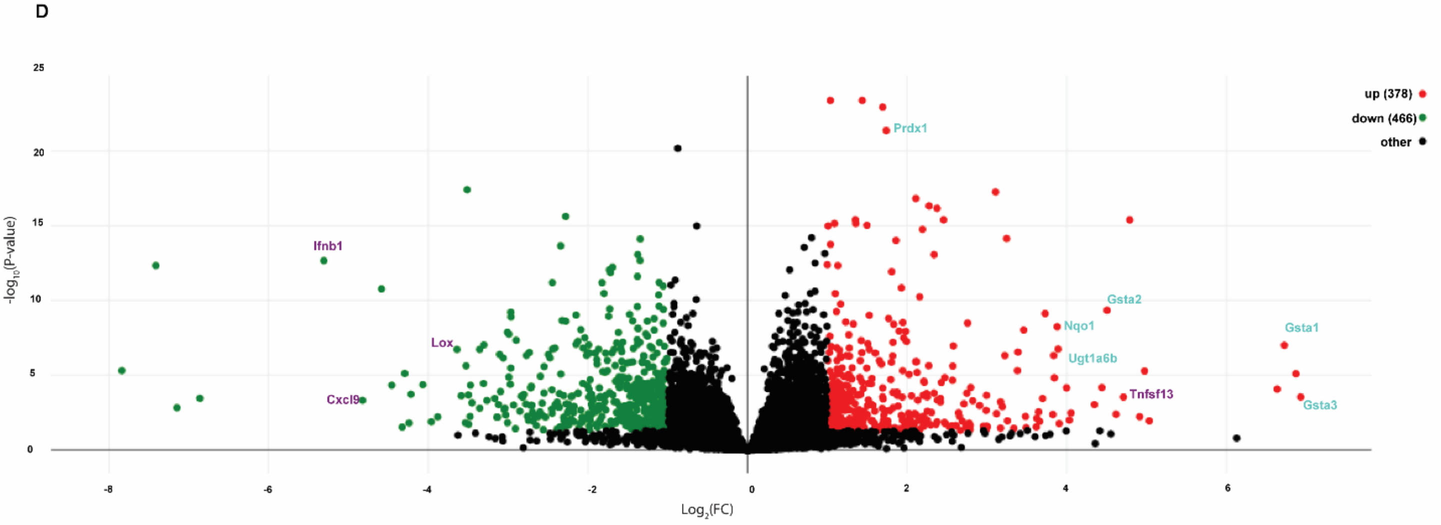

Volcano plots showing up- and down- regulated genes compared to the vehicle control for A) ajmalicine (10 genes up- or downregulated), B) compound **19** (208 up- and 355 downregulated), C) compound **20** (166 up- and 97 downregulated) and D) compound **9** (378 up- and 466 downregulated) (threshold > 2-fold, *p*-value < 0.05). In purple are the affected genes found in the NF-κB pathway, while in blue the Nrf2 pathway genes.

6.) Figure S5. Nrf2 Mediated Oxidative Stress Pathway for 20.

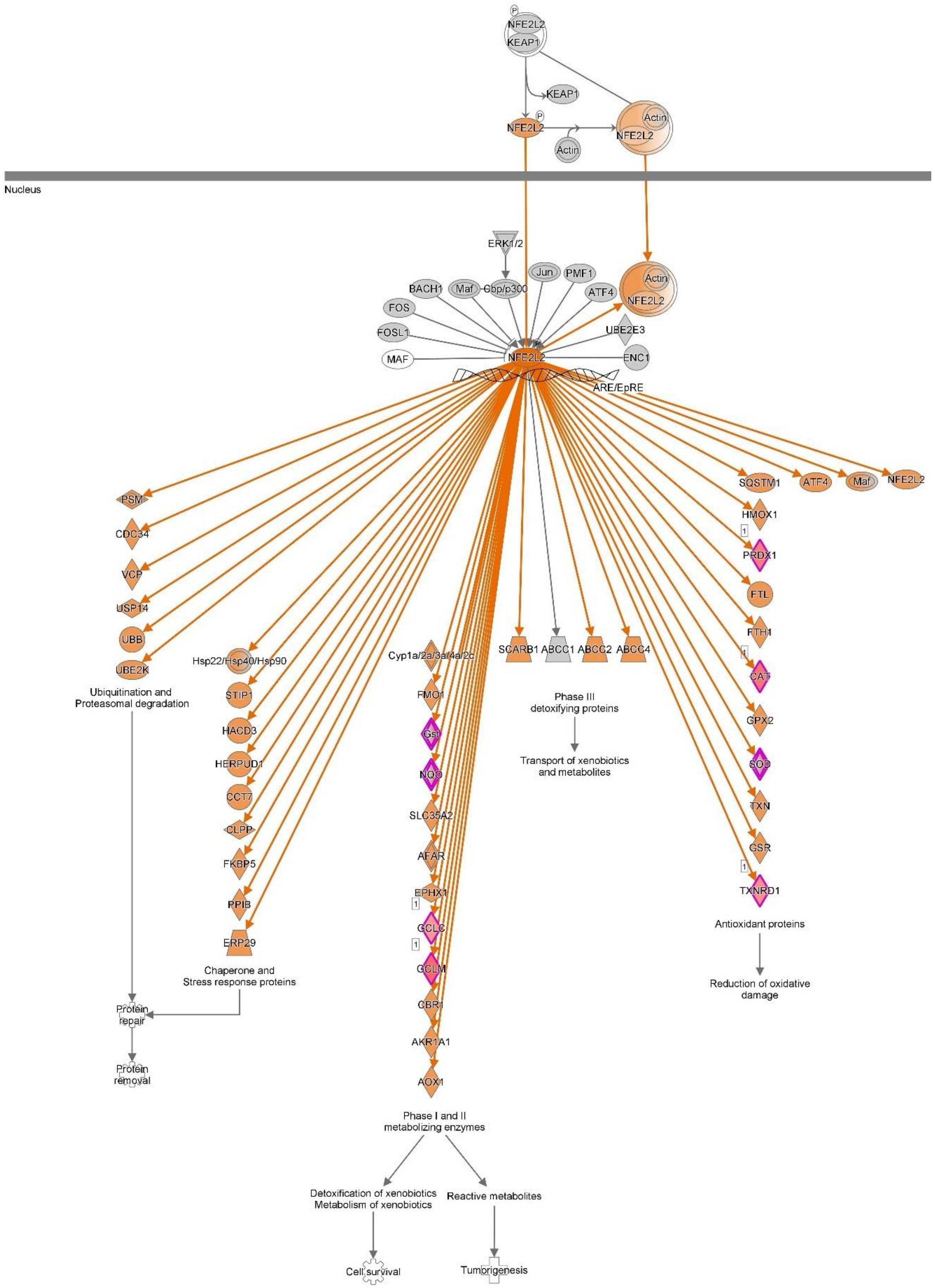

Nrf2 mediated oxidative stress pathway for **20** with activated genes in orange.

7.) Figure S6. Xenobiotic Metabolism General Signaling Pathway for 20.

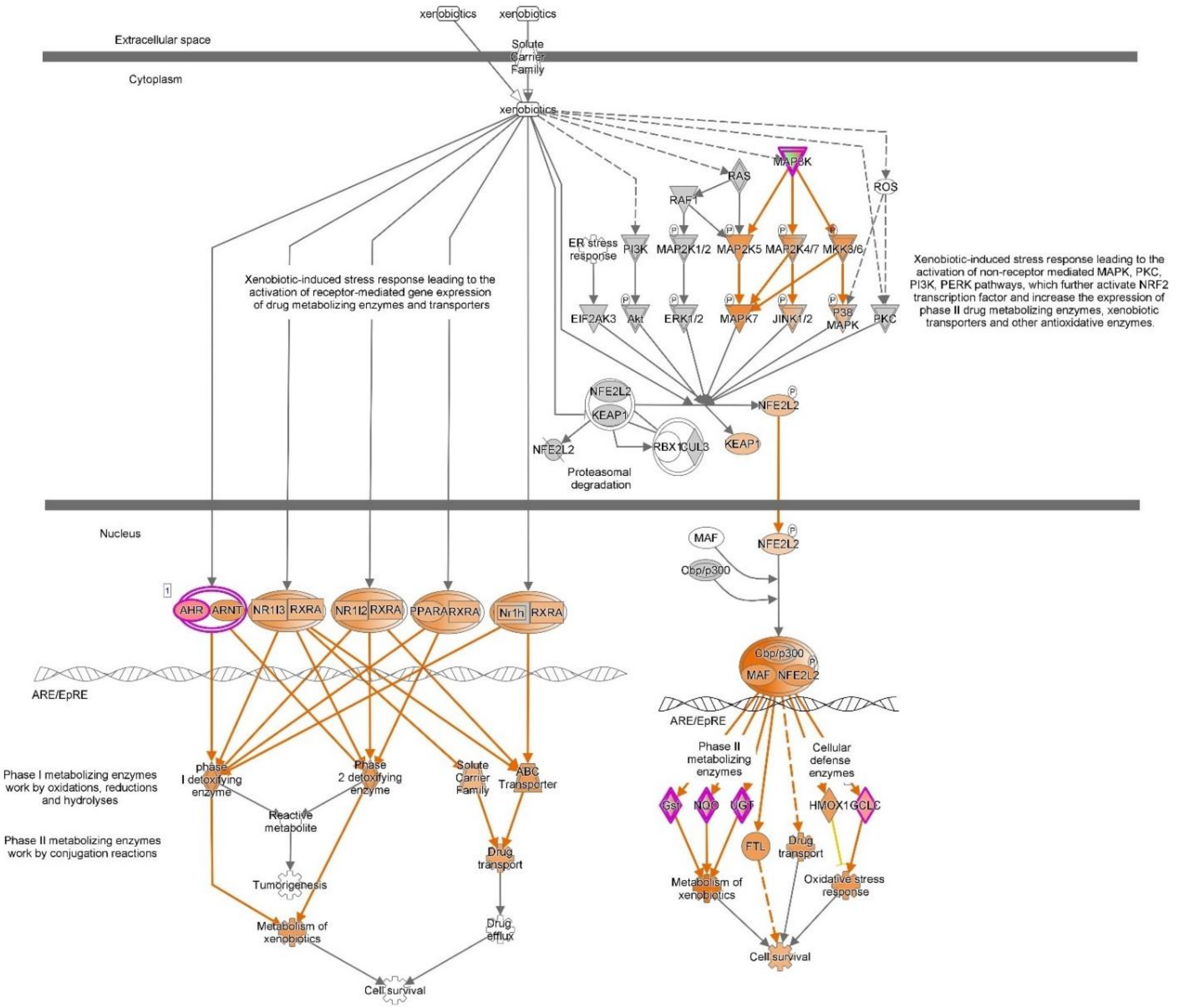

Xenobiotic metabolism general signaling pathway for **20** with activated genes in orange.

8.) **Figure S7. Structure-Activity Relationship Insights for New ARE-Activating Agents.**

ARE Activating Agents with SAR Insights

Active | **Inactive**

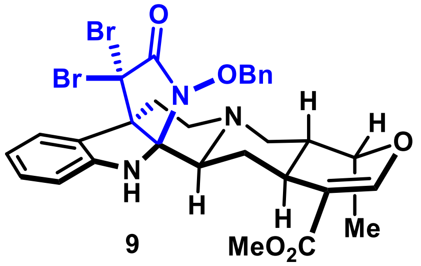

9

>>>

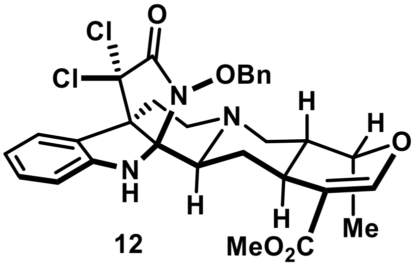

12

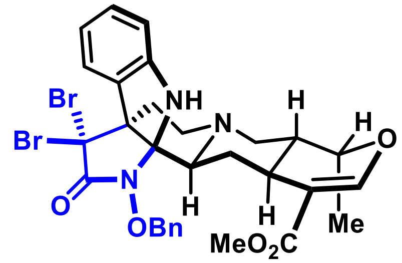

14

>>>

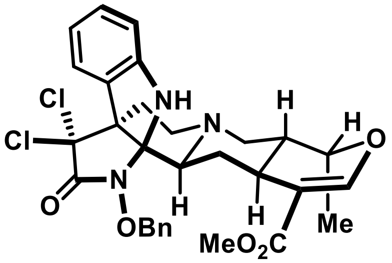

13

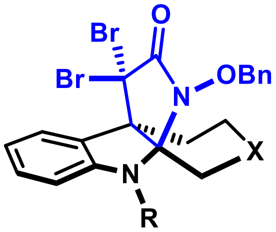

18. R = H, X = CH_2
19. R = H, X = NH
20. R = Me, X = NMe

>>>

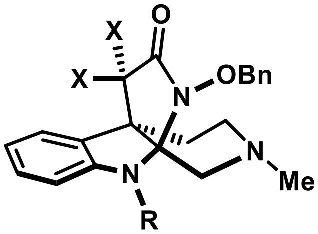

21. X = Cl
22. X = H

26. X = H, Y = Br
27. X = Br, Y = H

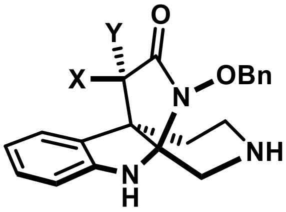

9.) **Figure S8. Mechanistic Pathway for Bromine Transfer Reaction.**

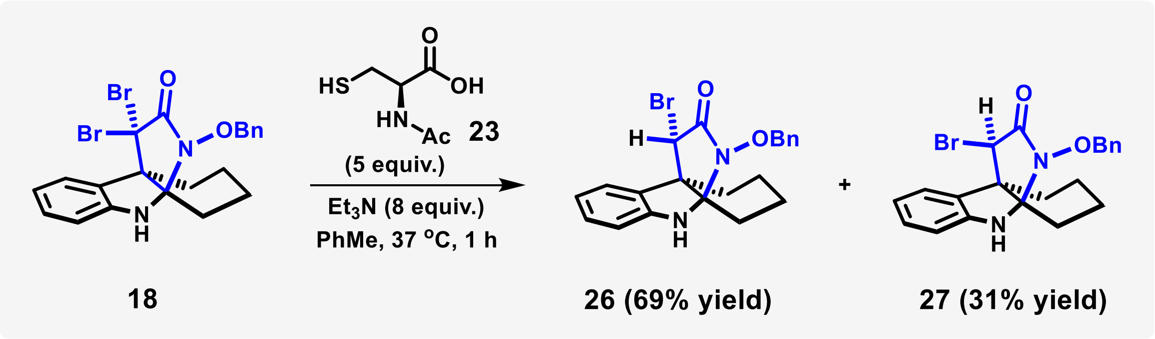

Mechanistic Pathway for Thiolate-Mediated Bromine Transfer

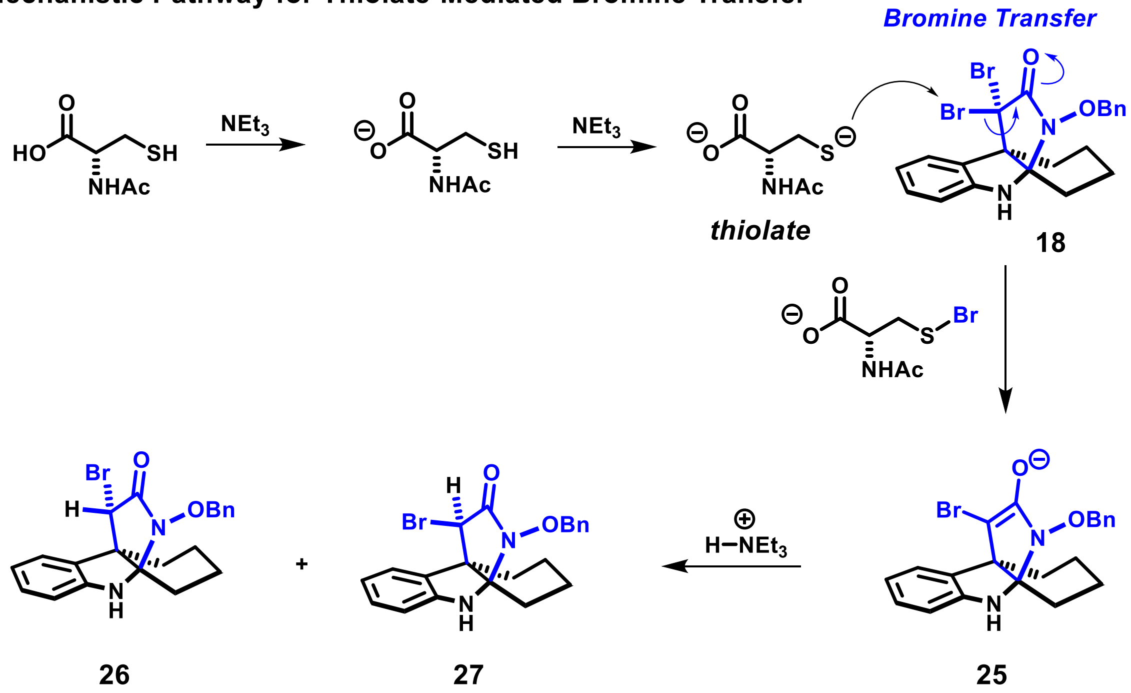

Possible Activation of Keap1 via Thiolate-Mediated Bromine Transfer

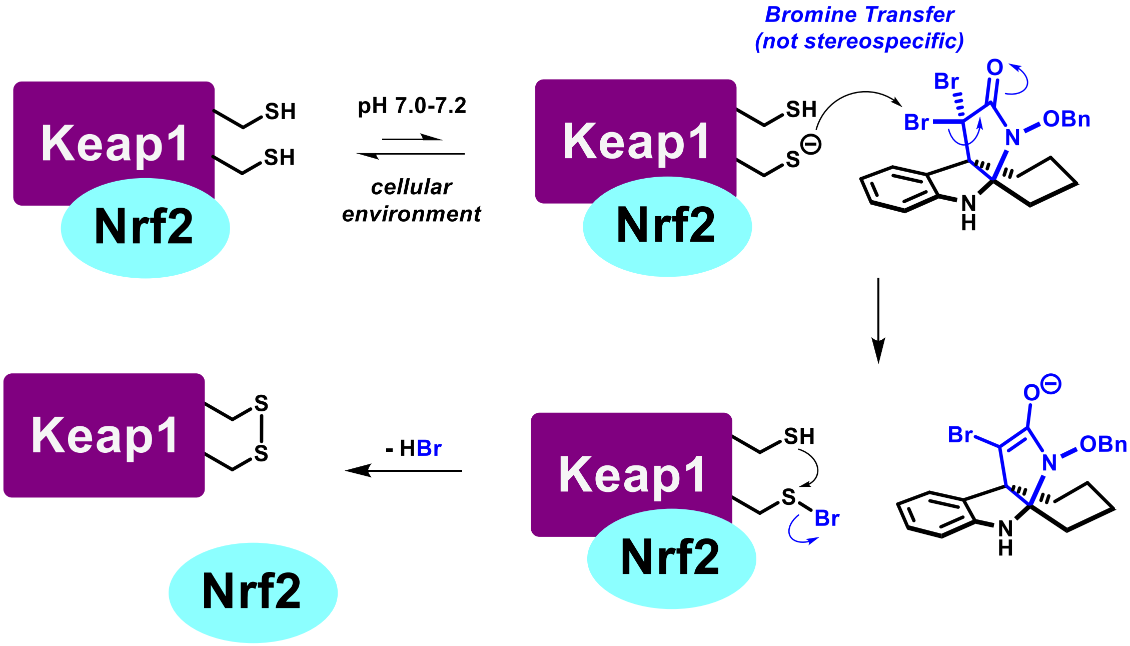

10.) Figure S9. Reacting 1,3-Propanedithiol with Molecular Bromine, NBS, or Compound 18.

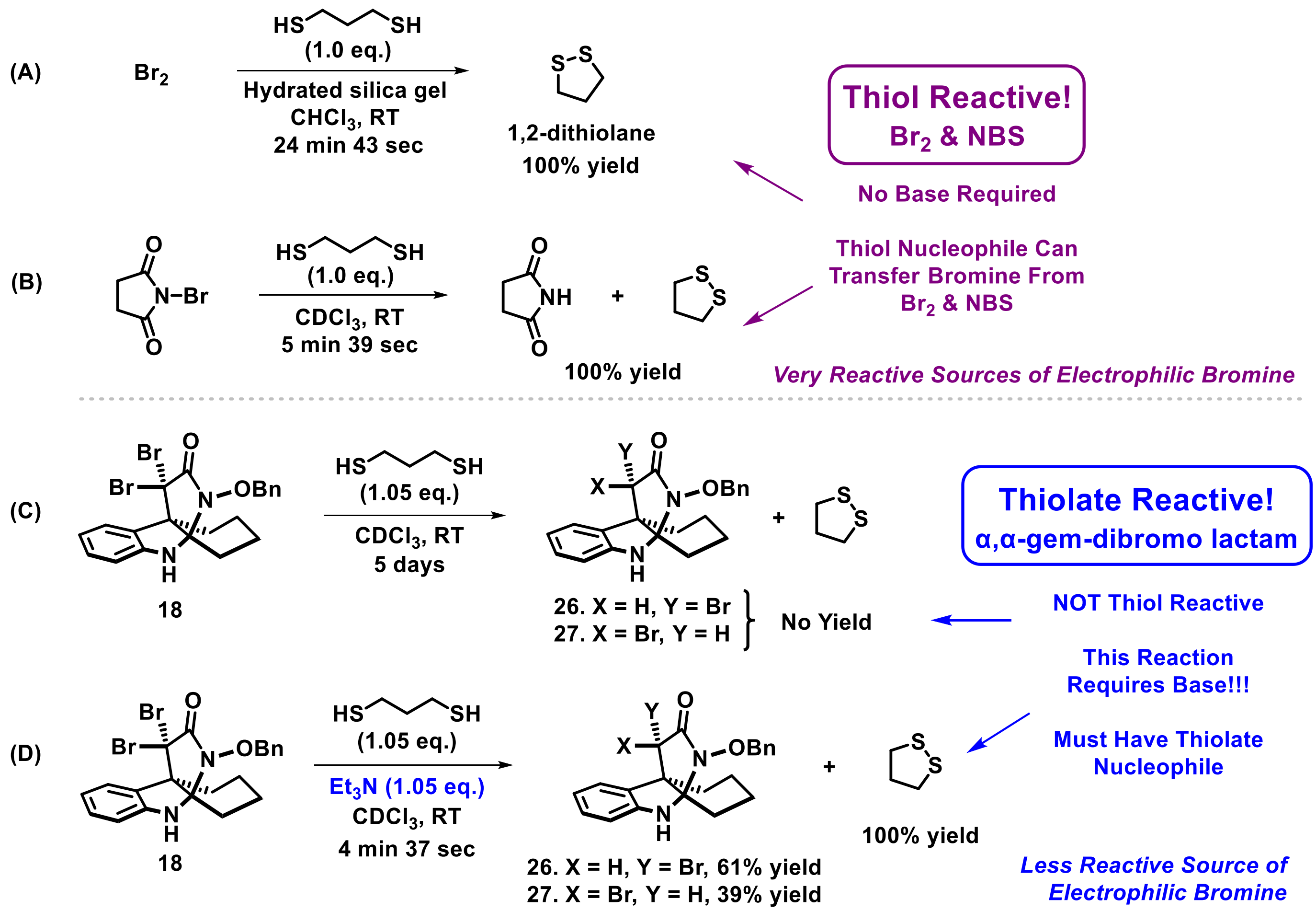

We probed the reactivity of various electrophilic bromine sources with 1,3-propanedithiol in NMR experiments to observe 1,2-dithiolane (a stable downstream product of bromine transfer in this reaction). Our goals were to determine (1) if a thiol was nucleophilic enough for bromine transfer, or see if a base required to generate an more reactive thiolate nucleophile, and (2) how reactive the α,α-*gem*-dibromo lactam warhead (**18**) is compared to established electrophilic bromine sources (Br_2 & NBS). All yields reported were not isolated, but determined by NMR as % conversion (from starting material to product based on NMR integrations). Experimental details include: **(A)** Deuterated chloroform (1.3 mL), 1,3-propanedithiol (0.1 mL, 1.0 mmol), and a mixture of molecular bromine (51 µL, 1.0 mmol) in 1.3 mL deuterated chloroform was sequentially added to pre-hydrated silica gel. After filtration, A 0.5 mL aliquot was transferred to an NMR tube for analysis. **(B)** *N*-bromosuccinimide (81.9 mg, 0.46 mmol) dissolved in 3.4 mL deuterated chloroform was mixed with 1,3-propanedithiol (46 µL, 0.46 mmol) in 3.4 mL deuterated chloroform. A 0.5 mL aliquot of the resulting mixture was transferred to an NMR tube for analysis. **(C)** 1,3-Propanedithiol (10.7 µL, 0.107 mmol) was added to a solution of **18** (50 mg, 0.102 mmol) in 1.5 mL deuterated chloroform. A 0.5 mL aliquot of the resulting mixture was transferred to an NMR tube. **(D)** 1,3-Propanedithiol (10.7 µL, 0.107 mmol) was added to a solution of **18** (50 mg, 0.102 mmol) in 0.75 mL deuterated chloroform. Separately, triethylamine (14.9 µL, 0.107 mmol) was dissolved in 0.75 mL chloroform. Then, 0.25 mL of each solution was combined in an NMR tube. Note: Reaction times were recorded from the addition of the final reagent to the start of the first NMR scan.

11.) Figure S10. ARE-luc Activation Time Course Study with Compound 18.

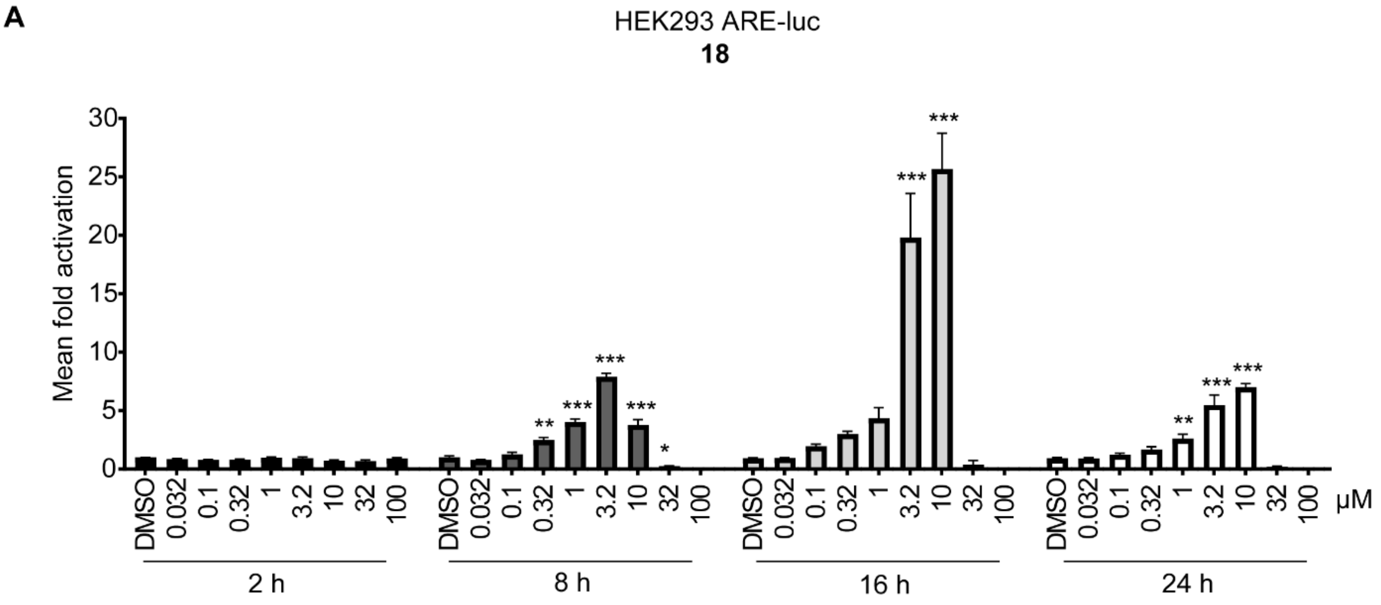

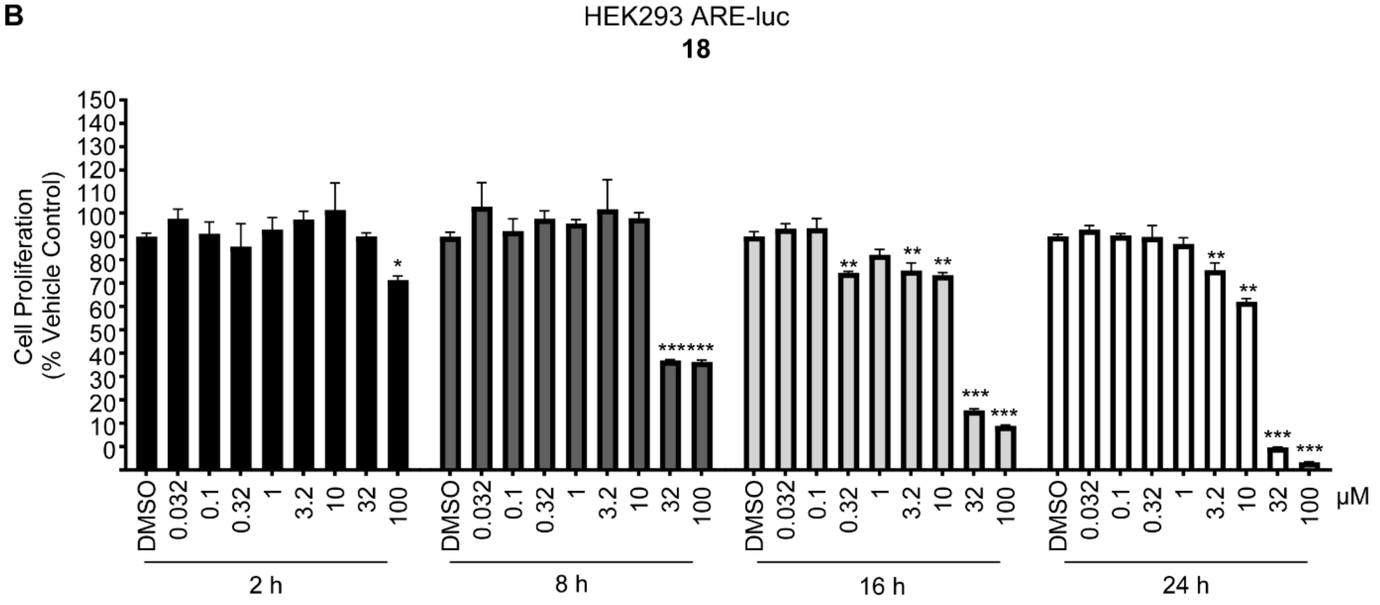

A time course study to correlate ARE-luc activity of compound **18** with GSH production in HEK293 cells. A) The dose response experiments showed compound **18** activated the reporter at later time points (8 h and especially 16 h), consistent with a transcriptional response. Treatment with **18** at non-toxic concentrations showed elevated luciferase activity, consistent with Nrf2/ARE activation and subsequent action of GSH biosynthesis enzymes. B) Cell viability studies of HEK293 treated with compound **18**.

12.) Figure S11. Pharmacological Characterization of Compound 18 at the ADRA1B Receptor.

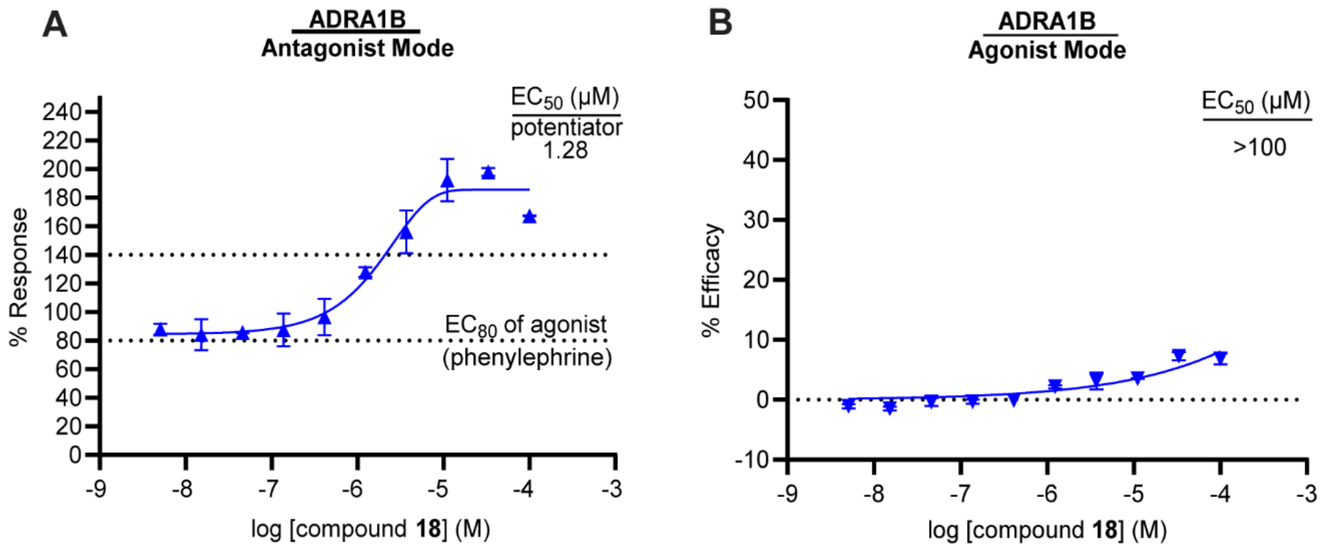

Pharmacological characterization of compound **18** at the ADRA1B receptor. A) Dose-response curve for compound **18** at ADRA1B generated using the PathHunter β-arrestin assay (DiscoverX/Eurofins) in antagonism mode. Cells were preincubated with increasing concentrations of compound **18** and subsequently stimulated with an EC_{80} concentration of phenylephrine (agonist). Receptor activity was quantified by chemiluminescence and expressed as percent (%) response relative to assay controls. Data corresponding to compound **18** at ADRA1B from **Fig. 6C** were normalized to a positive response range and baseline-corrected by adding 80% of the agonist response. Data points represent mean ± SD. B) Agonist activity of compound **18** at ADRA1B assessed in HEK293 PathHunter cells using the GPCR PathHunter β-arrestin assay. A concentration–response curve (0.005 - 100 μM) was generated, and EC_{50} values (μM) were determined by nonlinear regression analysis.

13.) Table S1. Investigating the Scope of the Bromine Transfer Reaction.

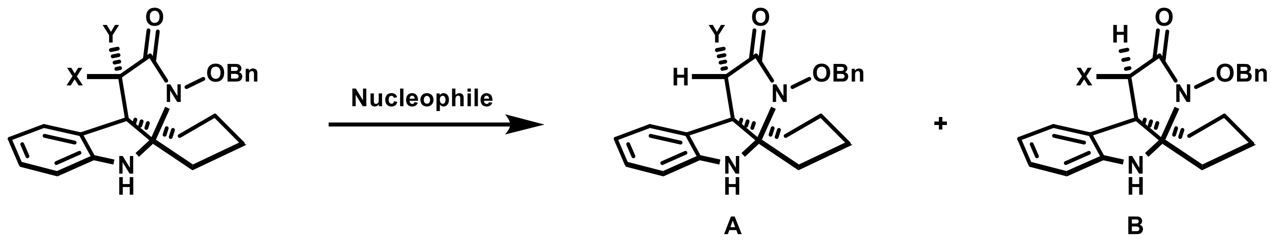

Entry	X	Y	Nucleophiles	Conditions	% Yield (A : B)
1	Br	Br	1.1 eq. $HSCH_2CH_2CH_2SH$	2 eq. Et_3N, PhMe, RT, 4 h	43% : 51%
2	Br	Br	5 eq. $HSCH_2CH_2CH_2SH$	8 eq. Et_3N, PhMe, 37 °C, 15 min	32% : 59%
3[a]	Br	Br	1.05 eq. $HSCH_2CH_2CH_2SH$	1.05 eq. Et_3N, $CDCl_3$, 37 °C, 1 h	55% : 45%
4[a]	Br	Br	1.05 eq. $HSCH_2CH_2CH_2SH$	1.05 eq. Et_3N, $CDCl_3$, RT, 4 min 37 sec	61% : 39%
5	Br	Br	1.05 eq. $HSCH_2CH_2CH_2SH$	$CDCl_3$, RT, 5 days	No Reaction
6	Br	Br	1.05 eq. $HSCH_2CH_2CH_2SH$	$CHCl_3$, 37 °C, 4 h	No Reaction
7	Br	Br	5 eq. $HSCH_2CH_2CH_2SH$	PhMe, 37 °C, 22 h	No Reaction
8	Br	Br	5 eq. NAC	8 eq. Et_3N, PhMe, 37 °C, 1 h	69% : 31%
9	Br	Br	5 eq. NAC	PhMe, 37 °C, 17 h	No Reaction
10	Br	Br	5 eq. NAC	8 eq. Et_3N, H_2O, MeCN, 37 °C, 1 h	18% : 80%
11	Br	Br	50 eq. NAC	H_2O, MeCN, 37 °C, 24 h	No Reaction
12	Br	Br	50 eq. NAC	H_2O, MeCN, 100 °C, 1 h	29% : 13%
13	Br	Br	No Nucleophile	H_2O, MeCN, 100 °C, 1 h	No reaction[b]
14	Br	Br	5 eq. $P(OEt)_3$	8 eq. Et_3N, PhMe, 37 °C, 1 h	23% : 8%
15	Br	Br	5 eq. $P(OEt)_3$	PhMe, 37 °C, 1 h	12% : 13%
16	Br	Br	5 eq. PPh_3	8 eq. Et_3N, PhMe, 37 °C, 1 h	39% : 11%
17	Br	Br	5 eq. PPh_3	PhMe, 37 °C, 1 h	15% : 68%
18	Br	Br	5 eq. $CH_3(CH_2)_7OH$	8 eq. Et_3N, PhMe, 37 °C, 24 h	No Reaction
19	Br	Br	5 eq. $CH_3(CH_2)_5NH_2$	8 eq. Et_3N, PhMe, 37 °C, 24 h	No Reaction
20	Cl	Cl	5 eq. $HSCH_2CH_2CH_2SH$	8 eq. Et_3N, PhMe, 37 °C, 24 h	No Reaction
21	Br	H	1.1 eq. NAC	2 eq. Et_3N, PhMe, RT, 22 h	No Reaction
22	H	Br	1.1 eq. NAC	2 eq. Et_3N, PhMe, RT, 21 h	No Reaction

Note: Yields were isolated in all cases with the exception of entry 3. The reaction in entry 12 was performed in an attempt to mimic conditions of the LC/MS experiment, where the heating probe operates at elevated temperatures to aid in dissolving the eluent and improve ionization efficiency. NAC: *N*-acetyl cysteine. [a]Reaction performed in $CDCl_3$ to characterize the formation of 1,2-dithiolane via ^1H NMR; % yields reported were determined by ^1H NMR analysis (as % conversion, no starting material remained in these NMR experiments). [b]Significant decomposition was observed, with 41% of the starting material being recovered.

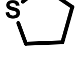

1,2-dithiolane (observed by NMR; Entry 3 and 4)

14.) Table S2. Fold Change of Genes in the Nrf2-Keap1 Canonical Pathways.

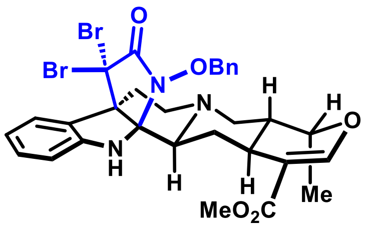

9

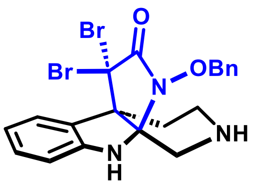

19

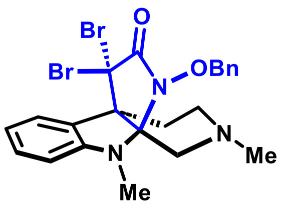

20

Symbol	9	19	20
Cat	3.2	2.3	2.5
Ephx1	2.1	-	-
Gclc	2.1	-	2.0
Gclm	4.3	2.5	2.8
Gsr	2.0	-	-
Gsta1	104.0	19.7	64.0
Gsta2	22.6	-	9.8
Gsta3	119.4	-	-
Gsta5	-	-	64.0
Gstm1	2.1	-	-
Gstm5	-	0.1	-
Gsto1	2.0	-	-
Gstt1	0.3	0.3	-
Mgst2	3.7	-	2.1
Nqo1	14.9	5.7	12.1
Prdx1	3.2	-	2.5
Prkce	0.4	0.5	-
Rasd1	0.1	-	-
Txnrd1	2.3	-	2.1

Fold change of genes in the Nrf2-Keap1 canonical pathways, up- (green) or down- (red) regulated by compounds **9**, **19**, and **20** (threshold >2-fold, p-value < 0.05).

15.) Table S3. Fold Change of Genes in the NF-κB Pathway by 9, 19, and 20.

Symbol	9	19	20
Abcb1	2.3	-	-
Acvrl1	-2.4	-2.9	-
Aebp1	10.6	-5.6	-5.6
Aldh1b1	-	-2.0	-2.3
Atf3	-	-2.0	-
Batf	-6.7	-5.3	-
Bcl3	-2.0	-2.4	-
Bst2	-2.0	-2.0	-
C1Ra	-	-2.4	-
C1Rb	-3.2	-2.9	-
C4b	5.7	-	-
Casp4	-2.3	-	-
Cbr3	3.7	2.5	2.8
Ccl22	-5.0	-	-2.9
Ccl7	-2.3	-	-
Cd40	-2.3	-	-
Cd82	2.3	-	2.0
Cdkn2d	-2.3	-2.6	-
Clec7a	-	3.2	-
Crip2	2.8	-	-
Cxcl9	-25.0	-	-
Daxx	-3.2	-2.3	-
Dcstamp	2.6	-	2.6
Dgka	-2.6	-2.1	-
Dhx58	-2.0	-	-
Egln1	-2.1	-3.0	-
Epsti1	-2.9	-2.1	-
Gadd45b	-2.9	-2.3	-2.4
Gas6	6.5	5.7	4.9
Gbp2	-2.4	-	-
Gclc	2.1	-	2.0
Gfpt2	-2.3	-	-
Ghr	-	5.7	-
Glrx	-2.9	-2.5	-
Gsr	2.0	-	-
Hcar2	-2.6	-	-2.0
Hivep2	-2.1	-	-
Id1	-	-4.0	-
Ifi202b	-2.4	-	-
Ifnb1	-33.3	-11.1	-4.3
Il15	-5.6	-3.2	-2.4
Il18	-4.5	-3.7	-2.9
Il18bp	-	-2.6	-
Il1r1	-3.2	-	-
Il1rl1	-	-	2.0
Il6	-4.3	-2.6	-2.9
Irf1	-2.1	-2.1	-
Irf7	-2.6	-	-
Irgm1	-2.0	-	-

Symbol	9	19	20
Lca5	-2.1	-	-
Lox	-12.5	-8.3	-3.7
Lsp1	-4.3	-5.0	-
Lta	-	-3.4	-
Ltc4s	3.5	-	-
Mmp9	2.8	3.7	3.7
Mt2	-2.1	-2.3	-
Mttp	2.5	2.1	2.3
Nfatc	-	2.0	-
Nlrc5	-2.3	-	-
Nmi	-2.0	-	-
Nod1	-5.6	-4.0	-2.6
Nox1	-4.3	-	-2.6
Nxn	-2.3	-3.2	-
Odc1	-2.1	-	-
Olr1	-2.6	-	-
Pim2	-2.6	-2.1	-
Pip5k1c	2.0	-	-
Pou2f2	-2.3	-2.3	-
Pou3f1	-7.1	-8.3	-
Psmb9	-2.6	-2.4	-
Rab31	-2.0	-	-
Rcan1	-5.0	-2.9	-2.6
Rel/Rela/Relb	-2.1	-	-
Rgs16	-5.3	-	-
Rsad2	-7.7	-4.5	-
Saa3	-3.0	-2.3	-2.6
Sbno2	-	-2.0	-
Slc16a3	-	-2.4	-
Slc1a2	8.0	13.9	-
Slc22a4	3.2	2.5	2.3
Slpi	10.6	-	4.0
Stat3-Stat3	-2.1	-	-
Tert	-2.4	-	-
Tfec	3.2	3.2	3.0
Tifa	-	-2.3	-
Timp2	4.9	-	6.1
Tlr8	-8.3	-3.2	-3.2
Tnfrsf14	-3.7	-	-
Tnfrsf9	-5.3	-	-
Tnfsf10	-4.5	-3.7	-
Tnfsf13	26.0	21.1	27.9
Tnfsf14	-3.7	-2.9	-
Tnip3	-2.6	-2.3	-
Trem2	4.9	-	3.2
Trim30a/Trim30d	-10.0	-4.5	-
Trpc4	-5.0	-	-
Txnrd1	2.3	-	2.1
Uba7	-2.1	-	-

Itgax	3.7	-	-	Usp18	-2.6	-	-
Itpkb	-2.1	-2.0	-	Vegf	-	-2.0	-
Itpr	-2.6	-2.0	-	Vopp1	4.0	-	-
Jag1	2.5	2.0	2.1	Zbp1	-2.6	-	-
Kdm6b	-2.0	-	-				

Fold change of genes in the NF-κB pathway with up- (green) or down-regulated (red) by compounds **9**, **19**, and **20** (p-value < 0.05).

16.) Table S4. Top Diseases and Functions for 9.

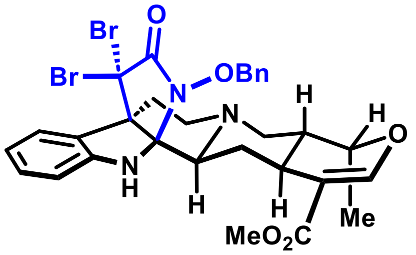

9

Data for Compound 9

Diseases or Functions	p-value	Activation z-score	No. of Molecules
Organismal death	1.40 E-14	3.898	230
Infection of mammalia	2.34 E-23	3.805	64
Infection by coronavirus	1.16 E-16	3.075	73
Viral Infection	9.78 E-20	2.974	196
Infection by RNA virus	9.54 E-12	2.734	119
Leukopoiesis	2.63 E-17	-3.628	108
Immune response of cells	1.64 E-09	-3.668	72
Proliferation of lymphocytes	2.77 E-17	-3.787	100
Proliferation of lymphatic system cells	8.69 E-19	-4.016	108
Proliferation of blood cells	1.91 E-20	-4.017	119

In green are the positive (activated) and red (decreased) z-scores. Furthermore, in blue are the overlapping functions of **9** and **20**, orange indicates overlapping functions of **9** and **19**, and purple represents overlap between **9**, **19**, and **20**.

17.) Table S5. Top Diseases and Functions for 19.

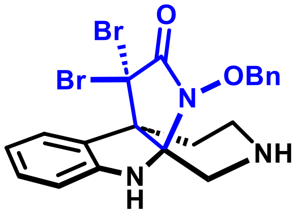

19

Data for Compound 19

**Diseases or Functions**	**p-value**	**Activation z-score**	**No. of Molecules**
Organismal death	4.59 E-12	2.877	161
Transport of molecule	8.00 E-08	2.307	102
Infection by coronavirus	1.70 E-09	2.219	45
Cell death of lymphatic system cells	1.31 E-07	2.112	37
Infection of mammalia	3.43 E-13	2.019	39
Maturation of blood cells	9.65 E-09	-3.358	28
Endothelial cell development	3.27 E-11	-3.188	41
Maturation of leukocytes	9.76 E-08	-3.103	24
Proliferation of lymphatic system cells	1.35 E-17	-3.077	82
Maturation of cells	1.67 E-11	-3.072	47

In green are the positive (activated) and red (decreased) z-scores. Furthermore, orange indicates overlapping functions of **9** and **19**, and purple represents overlap between **9**, **19**, and **20**.

18.) Table S6. Top Diseases and Functions for 20.

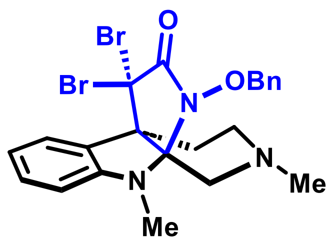

20

Data for Compound 20

**Diseases or Functions**	**p-value**	**Activation z-score**	**No. of Molecules**
Differentiation of bone cells	4.16 E-06	2.185	90
Apoptosis of neurons	1.79 E-05	-2.693	87
Proliferation of blood cells	9.35 E-11	-2.769	42
Proliferation of lymphatic system cells	1.51 E-10	-2.876	45
Apoptosis	3.95 E-08	-2.953	21
Necrosis	3.87 E-08	-3.203	20

In green are the positive (activated) and red (decreased) z-scores. Furthermore, in blue are the overlapping functions of **9** and **20**, and purple indicates overlap between **9**, **19** and **20**.

19.) Table S7. Hepatic Microsomal Stability Results.

	$t_{1/2}$ in Hepatic Microsomes (0.5 mg/mL)		Intrinsic Clearance	
**Compound ID**	**HLM ($t_{1/2}$ in min)**	**MLM ($t_{1/2}$ in min)**	**Human Cl_{int} (μL/min/mg)**	**Mouse Cl_{int} (μL/min/mg)**
9	1.1 ± 0.1	1.8 ± 0.1	1244 ± 57	758 ± 47
18	1.4 ± 0.1	1.5 ± 0.1	975 ± 32	942 ± 32
19	2.9 ± 0.0	3.3 ± 1.2	477 ± 4.3	474 ± 143
20	1.1 ± 0.0	1.3 ± 0.0	1268 ± 5.4	1077 ± 14
Ajmalicine	23.2 ± 1.2	4.1 ± 0.2	60 ± 3.1	339 ± 18
Sunitinib	48.6 ± 10	37.9 ± 0.7	30 ± 5.5	37 ± 0.7

Notes: $t_{1/2}$ = Half life; HLM = Human liver microsomes; MLM = Mouse liver microsomes; Cl_{int} = Microsomal intrinsic clearance rate.

20.) Procedures for Synthesis and Characterization Data.

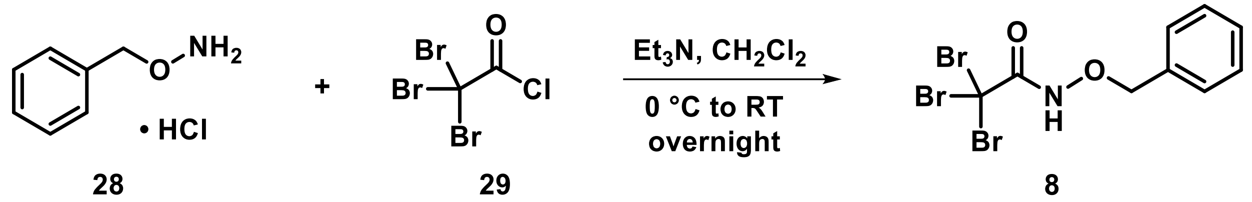

Procedure. Triethylamine (2.3 mL, 16.5 mmol) was added to a stirring solution of *O*-benzylhydroxylamine hydrochloride **28** (2.0 g, 12.7 mmol) in dichloromethane (52 mL). The reaction mixture was then cooled to 0 ºC before tribromoacetyl chloride **29** (2.5 mL, 12.7 mmol) was added dropwise. The resulting reaction mixture was then allowed to stir for five hours at 0 ºC before warming to room temperature and continuing for an additional 11 hours. Upon completion of the reaction, the mixture was then quenched with water and dichloromethane was added, and the contents were then transferred to a separatory funnel for extraction. Following extraction with dichloromethane, the resulting organic layers were collected, dried with sodium sulfate, filtered and concentrated via rotovap. The resulting crude product was purified via column chromatography using a gradient of 100% hexanes to 5:1 hexanes:ethyl acetate to afford 2.8 grams of desired amide **8** as a white solid (55% yield). Note: This procedure was used to make related amide **11**.

^{1}H NMR (600 MHz, $CDCl_3$): δ 9.36 (br m, 1H), 7.48 - 7.43 (m, 2H), 7.43 - 7.34 (m, 3H), 5.00 (s, 2H).

^{13}C NMR (151 MHz, $CDCl_3$): δ 159.9, 134.1, 129.8, 129.4, 128.9, 78.5, 31.1.

HRMS (DART): calc. for $C_9H_9Br_3NO_2$ $[M+H]^+$: 401.8158, found: 401.8159.

MP: 74 - 76 °C.

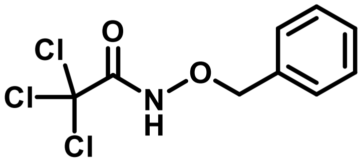

11

Yield: 81% yield; 3.6 grams of **11** was isolated as a white solid.

Note: This is a known compound (CAS No.: 22426-88-6). Our spectral data for this compound matched the reported data in the literature[1] and we have included ^{1}H and ^{13}C NMR in the spectra section of this document as an internal reference for this study.

^{1}H NMR (600 MHz, $CDCl_3$): δ 9.11 (s, 1H), 7.47 - 7.37 (m, 5H), 5.01 (s, 2H).

^{13}C NMR (151 MHz, $CDCl_3$): δ 159.7, 134.0, 129.8, 129.5, 128.9, 90.4, 78.7.

MP: 72 - 74 °C.

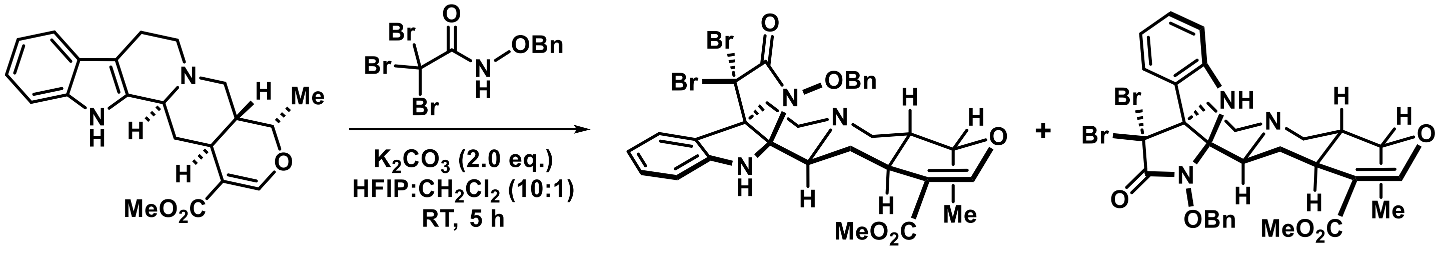

Ajmalicine 7 **9 (63% yield)** **14 (21% yield)**

Procedure. Ajmalicine **7** (300 mg, 0.851 mmol) was added to a flame-dried round-bottom flask and dissolved in a mixture of hexafluoroisopropanol (2.8 mL) and dichloromethane (0.28 mL). *N*-(benzyloxy)-*2,2,2*-tribromoacetamide (410 mg, 1.02 mmol) was then added to the reaction flask, followed by potassium carbonate (235 mg, 1.70 mmol). The resulting reaction was then stirred for five hours at room temperature until complete as determined by Thin Layer Chromatography (TLC) analysis. After this time, the reaction mixture was then filtered through a short pad of celite and the filtrate was concentrated under reduced pressure. The crude material was then transferred to a separatory funnel (partitioned between water and dichloromethane), extracted with dichloromethane, and dried over sodium sulfate. The resulting residue was then purified via column chromatography using 100% dichloromethane before adding very small volumes of methanol (99.9:0.1 to 99.7:0.3 to 99.5:0.5 to 99.3:0.7 dichloromethane:methanol) to afford each diastereomer pure (diastereomer **14**, 121 mg, 21% yield, white solid, eluted off the column first; diastereomer **9**, 361 mg, 63% yield, white solid, eluted off the column second). Note: The diastereomeric ratio for this reaction was determined to be **3.4:1** using crude NMR analysis.

TLC in 99:1 Dichloromethane:Methanol

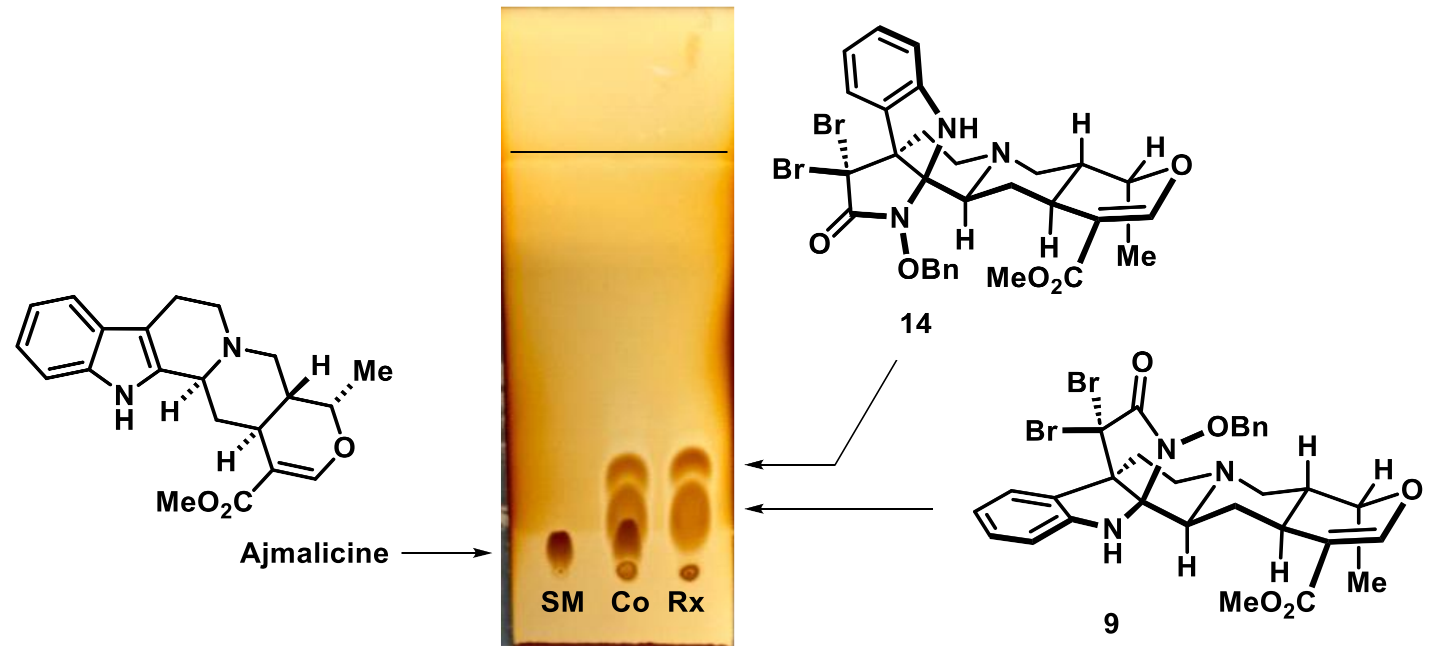

Note: TLC image was taken by Dr. Srinivasarao Tenneti.

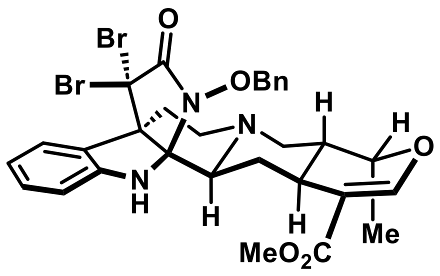

9

Yield: 63%; 361 mg of **9** was isolated as a white solid.

^{1}H NMR (600 MHz, CDCl$_3$): δ 7.47 (d, *J* = 1.3 Hz, 1H), 7.46 - 7.42 (m, 2H), 7.37 - 7.31 (m, 3H), 7.25 (ddd, *J* = 7.8, 7.8, 1.0 Hz, 1H, partially buried), 7.19 (d, *J* = 7.4 Hz, 1H), 6.94 (dd, *J* = 7.4, 7.4 Hz, 1H), 6.70 (d, *J* = 7.8 Hz, 1H), 5.23 (d, *J* = 9.1 Hz, 1H), 5.11 (d, *J* = 9.1 Hz, 1H), 4.95 (s, 1H), 4.24 (qd, *J* = 6.6, 3.9 Hz, 1H), 3.71 (s, 3H), 3.05 (m, 1H), 2.85 (ddd, *J* = 11.7, 3.8, 3.8 Hz, 1H), 2.78 (dd, *J* = 10.8, 2.7 Hz, 1H), 2.48 (ddd, *J* = 14.3, 2.5, 2.5 Hz, 1H), 2.19 (m, 1H), 2.12 (ddd, *J* = 14.1, 12.4, 4.1 Hz, 1H), 2.04 - 1.90 (m, 3H), 1.87 (dddd, *J* = 11.3, 11.3, 3.2, 3.2 Hz, 1H, "appt. tt"), 1.70 (dd, *J* = 11.1, 11.1 Hz, 1H), 1.00 (d, *J* = 6.6 Hz, 3H).

^{13}C NMR (151 MHz, CDCl$_3$): δ 167.3, 164.7, 154.9, 151.5, 134.0, 130.5, 130.2, 129.0, 128.5, 127.1, 123.7, 120.5, 111.0, 106.3, 86.5, 76.5, 73.3, 68.8, 66.7, 58.6, 57.3, 53.5, 51.0, 40.1, 32.5, 30.8, 29.4, 14.8.

HRMS (ESI): calc. for $C_{30}H_{32}Br_2N_3O_5$ $[M+H]^+$: 674.0685, found: 674.0689.

MP: 168-170 °C.

$[\alpha]_D^{20}$: -23° (*c* 0.98 g/100 mL, CHCl$_3$).

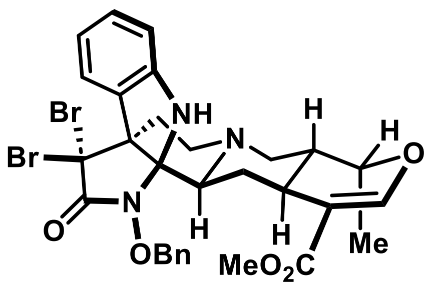

14

Yield: 21%; 121 mg of **14** was isolated as a white solid.

^{1}H NMR (600 MHz, CDCl$_3$): δ 7.67 (d, *J* = 7.8 Hz, 1H), 7.60 - 7.55 (m, 2H), 7.51 (d, *J* = 1.7 Hz, 1H), 7.37 - 7.33 (m, 3H), 7.08 (ddd, *J* = 7.9, 7.3, 1.1 Hz, 1H), 6.82 (ddd, *J* = 7.9, 7.3, 0.8 Hz, 1H), 6.57 (d, *J* = 7.8 Hz, 1H), 5.33 (d, *J* = 9.6 Hz, 1H), 5.00 (d, *J* = 9.6 Hz, 1H), 4.74 (s, 1H), 4.34 (qd, *J* = 6.6, 3.9 Hz, 1H), 3.56 (s, 3H), 3.39 (ddd, *J* = 12.7, 2.9, 2.9 Hz, 1H), 3.16 (ddd, *J* = 12.5, 12.5, 4.2 Hz, 1H), 3.03 (dd, *J* = 11.2, 2.4 Hz, 1H), 2.94 (dd, *J* = 10.8, 2.5 Hz, 1H), 2.79 (ddd, *J* = 15.2, 4.1, 1.3 Hz, 1H), 2.66 (ddd, *J* = 12.2, 6.9, 0.9 Hz, 1H), 2.25 (dddd, *J* = 11.4, 11.4, 2.6, 1.8 Hz, 1H), 2.12 - 2.02 (m, 2H), 1.98 (dddd, *J* = 11.0, 11.0, 3.0, 3.0 Hz, 1H, "appt. tt"), 1.30 (m, 1H, partially buried), 1.14 (d, *J* = 6.6 Hz, 3H).

^{13}C NMR (151 MHz, CDCl$_3$): δ 167.3, 162.4, 154.5, 145.4, 134.6, 133.0, 129.8, 129.6, 129.2, 128.7, 128.6, 120.7, 112.3, 106.9, 85.5, 78.5, 73.4, 63.9, 61.9, 58.9, 57.4, 52.5, 50.9, 40.5, 34.3, 30.7, 29.9, 14.8.

HRMS (ESI): calc. for $C_{30}H_{32}Br_2N_3O_5$ $[M+H]^+$: 674.0685, found: 674.0690.

MP: 156 - 158 °C, decomposed.

$[\alpha]_D^{20}$: +5° (*c* 0.56 g/100 mL, CHCl$_3$).

Note: The X-ray structure of **14** is reported in the next section.

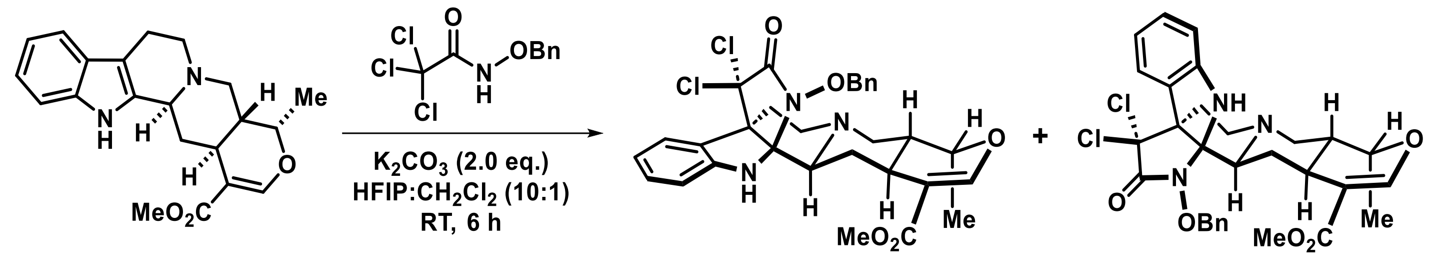

Ajmalicine **7** | **12 (33% yield)** | **13 (17% yield)**

Procedure. Ajmalicine **7** (200 mg, 0.567 mmol) was added to a flame-dried round-bottom flask and dissolved in a mixture of hexafluoroisopropanol (2.8 mL) and dichloromethane (0.28 mL). *N*-(benzyloxy)-*2,2,2*-trichloroacetamide (182 mg, 0.680 mmol) was then added to the mixture followed by the addition of potassium carbonate (157 mg, 1.13 mmol). The resulting reaction was then stirred for six hours at room temperature until complete by Thin Layer Chromatography (TLC) analysis, before being filtered through a short pad of celite. The resulting filtrate was then concentrated under reduced pressure. The resulting crude material was diluted with ethyl acetate and extracted, dried over sodium sulfate, filtered and concentrated via rotovap. The residue was then purified by column chromatography using a gradient of 100% hexanes to 8:2 to 1:1 to 1:2 hexanes:ethyl acetate to afford inseparable mixture of diastereomeric products **12** and **13** (240 mg, 72% combined yield of the diastereomeric mixture after the first column). With dichloromethane and methanol as solvent system for TLC, we found the two diastereomers (**12** & **13**) could be separated. Separation of the 240 mg sample of diastereomers was afforded using a second column (chromatography) with a gradient of 100% dichloromethane to 99.9:0.1 to 99.7:0.3 to 99.5:0.5 to 99.3:0.7 dichloromethane:methanol to afford each diastereomer pure (55 mg, 17% isolated yield of **13**, white solid, eluted off the column first; 110 mg, 33% isolated yield of **12**, white solid, eluted second). Note: The diastereomeric ratio for this reaction was determined to be **2.1:1** following the NMR acquired after the first column detailed (we were not initially aware this was a mixture of diastereomers **12** & **13**).

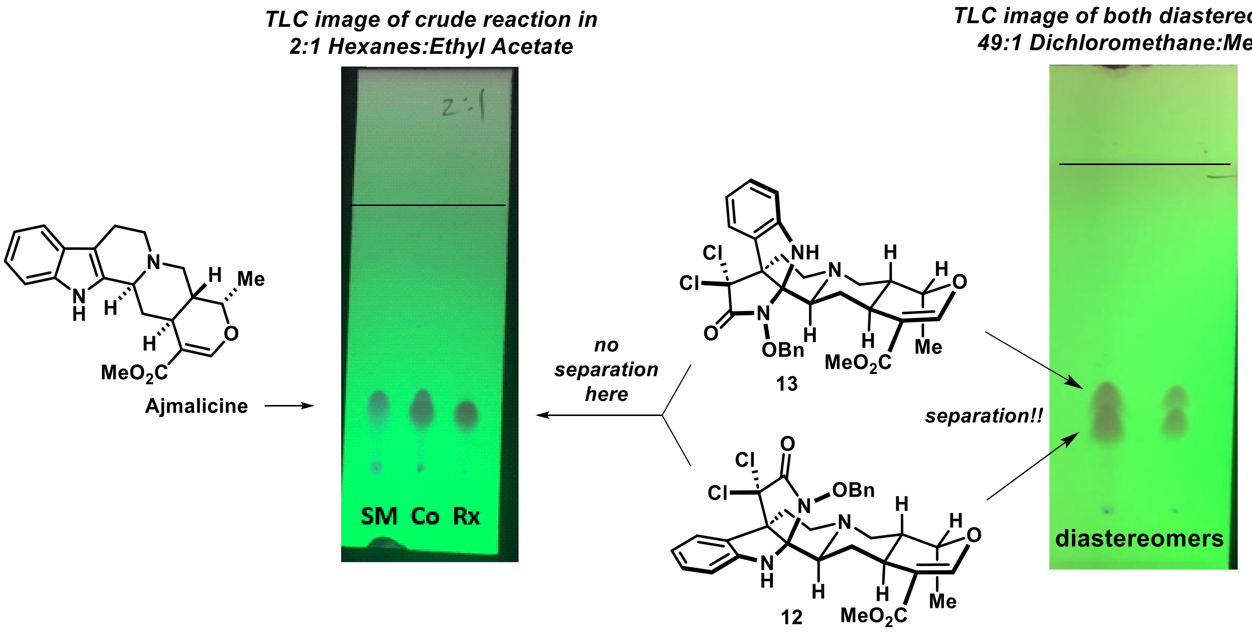

Note: TLC images were taken by Dr. Srinivasarao Tenneti.

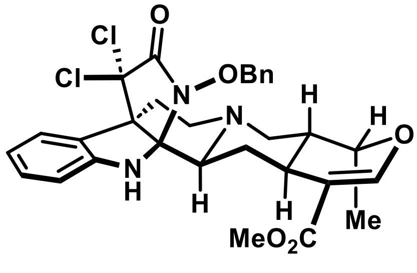

12

Yield: 33%; 110 mg of **12** was isolated as a white solid.

^{1}H NMR (600 MHz, $CDCl_3$): δ 7.48 (d, *J* = 1.0 Hz, 1H), 7.46 - 7.40 (m, 2H), 7.37 - 7.31 (m, 3H), 7.25 (ddd, *J* = 7.7, 7.7, 0.7 Hz, 1H, partially buried), 7.19 (d, *J* = 7.5 Hz, 1H), 6.92 (dd, *J* = 7.5, 7.5 Hz 1H), 6.69 (d, *J* = 7.8 Hz, 1H), 5.23 (d, *J* = 9.3 Hz, 1H), 5.12 (d, *J* = 9.3 Hz, 1H), 4.90 (s, 1H), 4.28 (qd, *J* = 6.5, 3.3 Hz, 1H), 3.72 (s, 3H), 3.05 (ddd, *J* = 12.6, 2.3, 2.3 Hz, 1H), 2.86 (ddd, *J* = 11.4, 3.7, 3.7 Hz, 1H), 2.81 (ddd, *J* = 10.7, 1.8, 1.8 Hz, 1H), 2.44 (ddd, *J* = 14.4, 2.5, 2.5 Hz, 1H), 2.24 (d, *J* = 10.8 Hz, 1H), 2.15 (ddd, *J* = 13.8, 13.8, 2.9 Hz, 1H), 2.07 (dd, *J* = 11.7, 11.7 Hz, 1H), 2.03 - 1.85 (m, 3H), 1.77 (dd, *J* = 10.8, 10.8 Hz, 1H), 1.02 (d, *J* = 6.5 Hz, 3H).

^{13}C NMR (151 MHz, $CDCl_3$): δ 167.3, 163.7, 154.9, 151.1, 134.0, 130.6, 130.2, 129.1, 128.6, 125.2, 124.2, 120.5, 111.0, 106.4, 87.4, 86.1, 76.9, 73.3, 68.1, 59.5, 57.4, 52.9, 51.1, 39.9, 30.8, 29.8, 29.2, 14.8.

HRMS (ESI): calc. for $C_{30}H_{32}Cl_2N_3O_5$ $[M + H]^+$: 584.1714, found: 584.1708.

MP: 154 - 156 °C.

$[\alpha]_D^{20}$: +5° (*c* 0.98 g/100 mL, $CHCl_3$).

Note: The X-ray structure of **12** is reported in the next section.

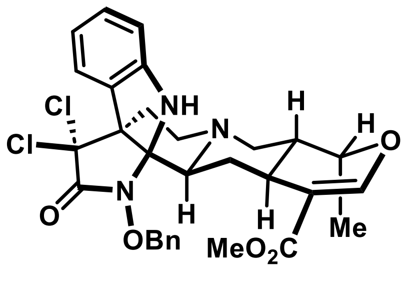

13

Yield: 17%; 55 mg of **13** was isolated as a white solid.

^{1}H NMR (600 MHz, $CDCl_3$): δ 7.60 - 7.46 (m, 4H), 7.40 - 7.32 (m, 3H), 7.09 (dd, *J* = 7.5, 7.5 Hz, 1H), 6.83 (dd, *J* = 7.5, 7.5 Hz, 1H), 6.59 (d, *J* = 7.8 Hz, 1H), 5.32 (d, *J* = 9.7 Hz, 1H), 4.96 (d, *J* = 9.7 Hz, 1H), 4.77 (s, 1H), 4.33 (qd, *J* = 6.5, 4.2 Hz, 1H), 3.55 (s, 3H), 3.40 (ddd, *J* = 12.5, 2.6, 2.6 Hz, 1H), 2.98 - 2.86 (m, 3H), 2.77 (dd, *J* = 15.2, 2.1 Hz, 1H), 2.72 (dd, *J* = 11.5, 6.5 Hz, 1H), 2.25 (dd, *J* = 10.9, 10.9 Hz, 1H), 2.06 (dd, *J* = 11.3, 10.9 Hz, 1H), 2.02 - 1.90 (m, 2H), 1.28 (m, 1H, partially buried), 1.14 (d, *J* = 6.5 Hz, 3H).

^{13}C NMR (151 MHz, $CDCl_3$): δ 167.3, 161.3, 154.5, 145.2, 134.5, 131.8, 129.6, 129.6, 129.1, 128.6, 128.2, 121.0, 112.3, 106.8, 86.1, 84.8, 78.6, 73.4, 64.4, 59.1, 57.3, 52.8, 50.9, 40.4, 32.4, 30.7, 29.9, 14.8.

HRMS (ESI): calc. for $C_{30}H_{32}Cl_2N_3O_5$ $[M + H]^+$: 584.1714, found: 584.1711.

MP: 160 - 162 °C, decomposed.

$[\alpha]_D^{20}$: +1° (*c* 0.29 g/100 mL, $CHCl_3$).

Note: The X-ray structure of **13** is reported in the next section.

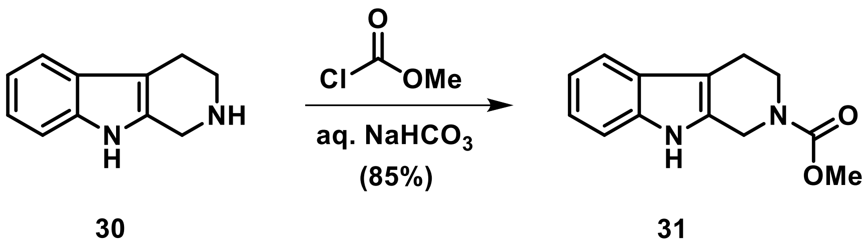

Procedure. A saturated solution of sodium bicarbonate (aqueous; 14 mL) was added to a stirring solution of tryptoline **30** (1.50 g, 8.71 mmol) in dichloromethane (14 mL) and allowed to stir for five minutes. Then, methyl chloroformate (808 µL, 10.5 mmol) was added to the mixture and the resulting reaction continued to stir for seven and a half hours at room temperature. Upon completion, the reaction was diluted with dichloromethane and transferred to a separatory funnel for extraction. Following extraction with dichloromethane, the resulting organic layers were then collected, dried with sodium sulfate, filtered and concentrated via rotovap to afford 1.81 grams of target compound **31** as a white solid (90% yield). Note: Compound **31** is a known (CAS: 89759-47-7) and our spectral data for this molecule matched with reported values in the literature.[2]

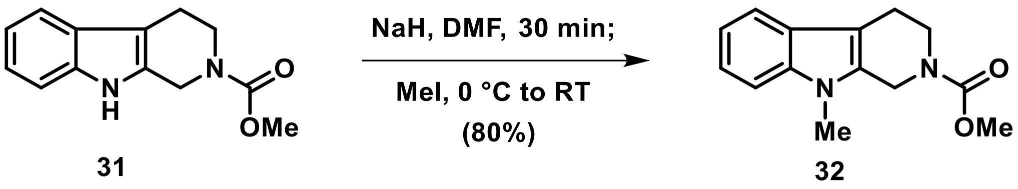

Procedure. *N,N*-Dimethylformamide (20 mL) was added to a round-bottom flask followed by sodium hydride (860 mg, 21.5 mmol, 60% dispersion in mineral oil) and the resulting mixture was then cooled to 0 °C. Next, a solution of compound **31** (3.3 g, 14.3 mmol; dissolved in 10 mL of *N,N*-dimethylformamide) was added to the reaction mixture, which was then allowed to stir for 30 minutes at room temperature. Following this time, the reaction mixture was then cooled to 0 °C and methyl iodide (2.85 g, 20.1 mmol) was added to the reaction and the temperature was slowly warmed to room temperature and continued stirring for 16 hours. Upon completion, the reaction was quenched with brine, transferred to a separatory funnel and extracted with ethyl acetate. The organic layers were then collected, dried with sodium sulfate, filtered and concentrated via rotovap. The crude product was then purified via column chromatography using 100% hexanes and ramping to 4:1 hexanes:ethyl acetate to give 2.9 grams of **32** (80% yield) as a semisolid.

^{1}H NMR (600 MHz, $CDCl_3$): δ 7.51 (d, *J* = 7.1 Hz, 1H), 7.29 (d, *J* = 8.1 Hz, 1H), 7.22 (dd, *J* = 8.1, 7.4 Hz, 1H), 7.13 (dd, *J* = 7.4, 7.1 Hz, 1H), 4.75 - 4.63 (m, 2H), 3.90 - 3.75 (m, 2H), 3.81 (s, 3H), 3.63 (s, 3H), 2.88 - 2.80 (m, 2H).

^{13}C NMR (151 MHz, $CDCl_3$): δ 156.6, 156.4*, 137.2, 131.8, 131.3*, 126.5, 121.3, 119.2, 118.1*, 118.0, 108.8, 107.9*, 107.4, 52.9, 42.3, 42.1*, 41.6, 41.4*, 29.4, 21.5, 21.1*. Note: Based on our ^{13}C NMR spectra, it appears that this compound exists as a mixture of rotamers. Regarding the tabulated ^{13}C NMR data, we have designated

* as a symbol to characterize what we believe to be the minor rotamer and signals of interest can be viewed in the spectra section of this document.

HRMS (ESI): calc. for $C_{14}H_{17}N_2O_2$ [M+H]$^+$: 245.1285, found: 245.1287.

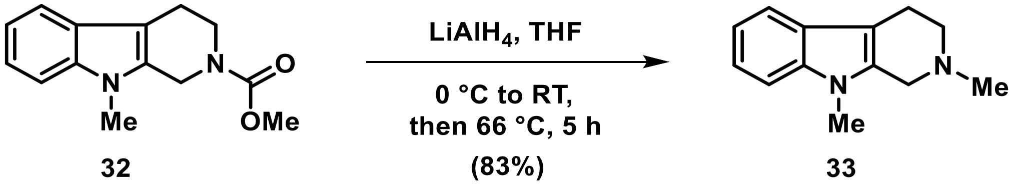

Procedure. Tetrahydrofuran (2 mL) was added to a round-bottom flask followed by lithium aluminium hydride (0.78 g, 20.5 mmol) before the mixture was cooled to 0 °C. Next, a solution of **32** (1.0 g, 4.09 mmol; dissolved in 0.5 mL of tetrahydrofuran) was added to the reaction mixture which was then allowed to warm to room temperature over 30 minutes before being heated to 66 °C and allowed to react for five hours. Upon completion of the reaction, the mixture was cooled and then quenched with 1 M sodium hydroxide (2 mL, aqueous solution), followed by the addition of distilled water (2 mL). The resulting mixture was then filtered through a frit funnel containing celite. The filtrate was then rinsed with warm ethyl acetate. The resulting organic solution collected contained the product was dried with sodium sulfate and concentrated via rotovap to afford 682 mg of **33** (83% yield) as a tan solid. Note: This is a known compound (CAS No.: 18144-39-3).

^{1}H NMR (600 MHz, $CDCl_3$): δ 7.53 (d, *J* = 7.6 Hz, 1H), 7.29 (m, 1H), 7.21 (m, 1H), 7.13 (m, 1H), 3.69 - 3.65 (m, 2H), 3.60 (s, 3H), 2.92 - 2.87 (m, 2H), 2.85 - 2.80 (m, 2H), 2.62 - 2.57 (m, 3H).

^{13}C NMR (151 MHz, $CDCl_3$): δ 137.2, 133.4, 126.8, 120.8, 118.8, 118.0, 108.7, 107.0, 53.0, 51.6, 46.0, 29.2, 21.6.

HRMS (DART): calc. for $C_{13}H_{17}N_2$ [M+H]$^+$: 201.1386, found: 201.1392.

MP: 84 - 86 °C (free base).

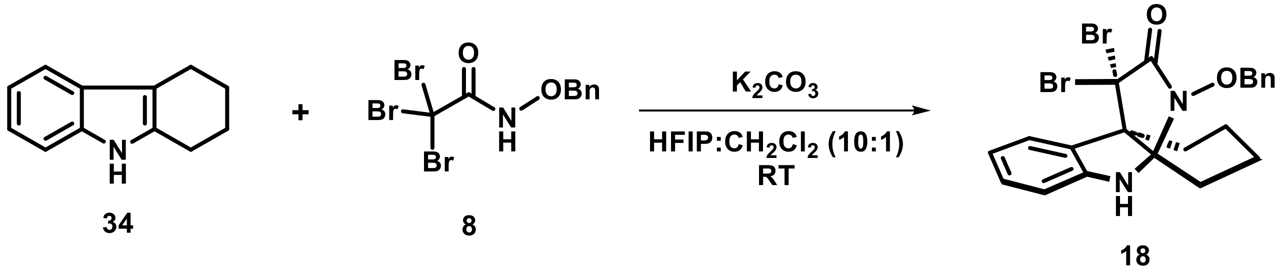

Procedure. 1,2,3,4-Tetrahydrocarbazole **34** (200 mg, 1.17 mmol) was added to a flame-dried round-bottom flask and dissolved in hexafluoroisopropanol (10 mL) and dichloromethane (1 mL). Then, *N*-(benzyloxy)-2,2,2-tribromoacetamide **8** (560 mg, 1.40 mmol) was added to the reaction in one portion before potassium carbonate (323 mg, 2.24 mmol) was added. The resulting reaction mixture was then stirred for two hours at room temperature. Upon completion (by TLC analysis), the reaction was quenched with brine and diluted with ethyl

acetate. The aqueous layer was then extracted with ethyl acetate and the organic layers were combined, dried over sodium sulfate, and concentrated under reduced pressure. Finally, the crude residue was purified via column chromatography using a gradient of 100% hexanes to 9:1 hexanes:ethyl acetate to afford **18** (389 mg, 71%) as a colorless foam. Note: This procedure was used to make related ring fusion products during the course of these studies.

^{1}H NMR (600 MHz, $CDCl_3$): δ 7.50 - 7.43 (m, 3H), 7.42 - 7.37 (m, 3H), 7.11 (ddd, *J* = 7.8, 7.5, 1.1 Hz, 1H), 6.82 (ddd, *J* = 7.6, 7.5, 0.9 Hz, 1H), 6.49 (d, *J* = 7.8 Hz, 1H), 5.15 (d, *J* = 10.5 Hz, 1H), 5.13 (d, *J* = 10.5 Hz, 1H), 4.18 (s, 1H), 2.43 (ddd, *J* = 14.7, 7.3, 4.9 Hz, 1H), 2.04 (ddd, *J* = 14.6, 10.3, 4.6 Hz, 1H), 2.00 - 1.90 (m, 2H), 1.85 (dddd, *J* = 13.9, 10.4, 6.9, 4.4 Hz, 1H), 1.57 (m, 1H), 1.45 (m, 1H), 1.33 (m, 1H).

^{13}C NMR (151 MHz, $CDCl_3$): δ 162.5, 147.8, 134.9, 131.0, 129.8, 129.6, 129.3, 128.8, 127.0, 119.7, 110.4, 84.9, 78.3, 65.4, 58.5, 34.2, 30.5, 20.6, 18.9.

HRMS (ESI): calc. for $C_{21}H_{21}Br_2N_2O_2$ $[M + H]^+$: 492.9945, found: 492.9961.

MP: 128 - 130 °C.

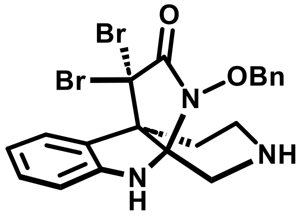

19

Yield: 65% yield; 370 mg of **19** was isolated as a tan foam.

^{1}H NMR (600 MHz, $CDCl_3$): δ 7.48 - 7.35 (m, 6H), 7.13 (dd, *J* = 7.6, 7.6 Hz, 1H), 6.85 (dd, *J* = 7.6, 7.6 Hz, 1H), 6.53 (d, *J* = 7.9 Hz, 1H), 5.16 (d, *J* = 10.7 Hz, 1H), 5.10 (d, *J* = 10.7 Hz, 1H), 4.33 (s, 1H), 3.21 (d, *J* = 14.0 Hz, 1H), 3.09 (ddd, *J* = 13.0, 7.6, 4.8 Hz, 1H), 2.89 (d, *J* = 14.0 Hz, 1H), 2.60 (ddd, *J* = 12.1, 5.8, 5.8 Hz, 1H), 2.32 (ddd, *J* = 14.8, 6.0, 5.2 Hz, 1H), 1.98 (ddd, *J* = 14.4, 7.6, 5.3 Hz, 1H).

^{13}C NMR (151 MHz, $CDCl_3$): δ 163.1, 148.1, 134.7, 130.4, 130.0, 129.9, 129.5, 128.9, 126.4, 120.2, 110.9, 82.0, 78.3, 64.9, 57.2, 49.3, 42.2, 34.8.

HRMS (ESI): calc. for $C_{20}H_{20}Br_2N_3O_2$ $[M+H]^+$: 493.9898, found: 493.9911.

MP: 87 - 89 °C.

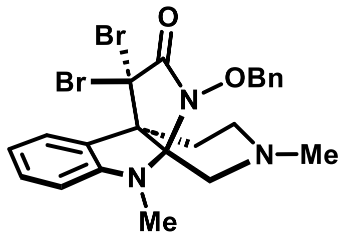

20

Yield: 63% yield; 82 mg of **20** was isolated as a tan solid.

^{1}H NMR (600 MHz, $CDCl_3$): δ 7.52 - 7.49 (m, 2H), 7.44 - 7.38 (m, 3H), 7.27 (ddd, *J* = 7.8, 7.8, 1.2 Hz, 1H), 7.19 (dd, *J* = 7.4, 0.7 Hz, 1H), 6.86 (ddd, *J* = 7.5, 7.5, 0.7 Hz, 1H), 6.41 (d, *J* = 7.8 Hz, 1H), 5.44 (d, *J* = 9.6 Hz, 1H), 5.06 (d, *J* = 9.6 Hz, 1H), 3.47 (dd, *J* = 12.8, 1.1 Hz, 1H), 2.80 (s, 3H), 2.79 (m, 1H), 2.43 (ddd, *J* = 14.2, 3.0, 3.0 Hz, 1H), 2.27 (s, 3H), 2.22 (ddd, *J* = 14.2, 11.6, 4.6 Hz, 1H), 2.09 (d, *J* = 12.8 Hz, 1H), 1.96 (ddd, *J* = 11.5, 11.5, 2.3 Hz, 1H).

^{13}C NMR (151 MHz, CDCl$_3$): δ 163.5, 152.4, 134.5, 130.6, 129.5, 129.2, 128.8, 126.8, 123.8, 118.9, 106.9, 87.5, 79.4, 66.8, 56.5, 55.4, 52.9, 45.7, 33.5, 29.5.

HRMS (ESI): calc. for $C_{22}H_{24}Br_2N_3O_2$ $[M+H]^+$: 522.0211, found: 522.0204.

MP: 120 - 122 °C, decomposed.

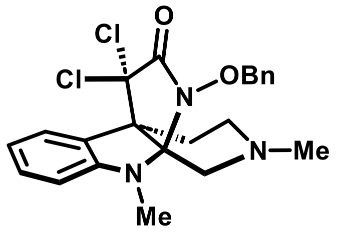

21

Yield: 78% yield; 254 mg of **21** was isolated as a white solid.

^{1}H NMR (600 MHz, CDCl$_3$): δ 7.50 - 7.46 (m, 2H), 7.44 - 7.38 (m, 3H), 7.26 - 7.23 (m, 2H), 6.83 (ddd, *J* = 7.5, 7.5, 0.8 Hz, 1H), 6.43 (d, *J* = 8.1 Hz, 1H), 5.33 (d, *J* = 9.7 Hz, 1H), 5.10 (d, *J* = 9.7 Hz, 1H), 3.23 (dd, *J* = 12.7, 1.0 Hz, 1H), 2.86 (s, 3H), 2.73 (m, 1H), 2.37 - 2.26 (m, 2H), 2.35 (d, *J* = 12.7 Hz, 1H), 2.29 (s, 3H), 2.15 (ddd, *J* = 11.6, 9.3, 3.9 Hz, 1H).

^{13}C NMR (151 MHz, CDCl$_3$): δ 162.1, 151.1, 134.3, 130.4, 129.5, 129.2, 128.7, 126.0, 124.7, 118.9, 107.1, 87.8, 86.6, 79.0, 57.1, 54.8, 52.4, 45.7, 31.4, 29.5.

HRMS (ESI): calc. for $C_{22}H_{24}Cl_2N_3O_2$ $[M+H]^+$: 432.1240, found: 432.1240.

MP: 76 - 78 °C.

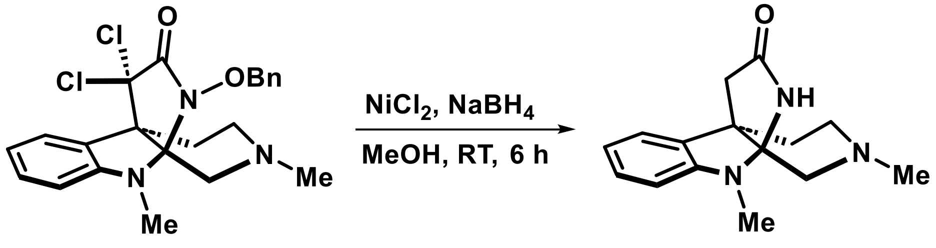

21 **22**

98:1:1 EtOAc:MeOH:TEA

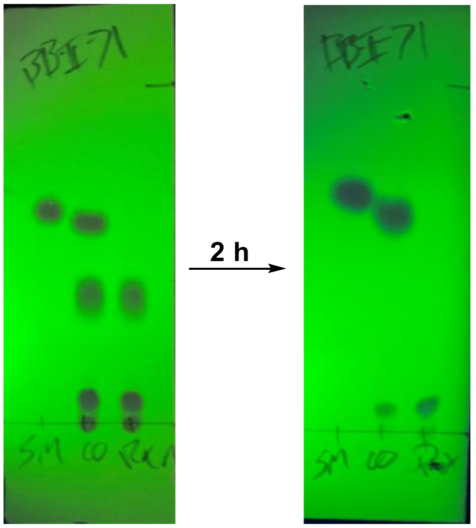

2nd Portion of Reagent Pool

Note: TLC images for the reaction above were taken by Dr. Beau Brummel.

Procedure. Compound **21** (150 mg, 0.35 mmol) and nickel(II) chloride (63 mg, 0.48 mmol) were added to a flame-dried round-bottom flask and dissolved in methanol (5 mL). Sodium borohydride (159 mg, 5.76 mmol) was then added slowly over five minutes to the resulting solution. The resulting reaction mixture was then stirred for four hours at room temperature. After consumption of the starting material (confirmed by TLC), a second portion of nickel(II) chloride (63 mg, 0.48 mmol) and sodium borohydride (159 mg, 5.76 mmol) was added to the reaction

mixture, which was then stirred for an additional two hours before the solvent was removed under reduced pressure. The crude mixture was then treated with 2N hydrochloric acid (aqueous) and washed with ethyl acetate. The aqueous layer was then basified with saturated sodium bicarbonate and extracted with ethyl acetate. The extract layers were then combined, dried over sodium sulfate, and the solvent was removed under reduced pressure to afford the fully reduced lactam **22** (36 mg, 40%) as a colorless foam.

^{1}H NMR (600 MHz, $CDCl_3$): δ 8.13 (s, 1H), 7.13 (dd, *J* = 7.6, 7.6 Hz, 1H), 7.02 (d, *J* = 7.2 Hz, 1H), 6.76 (dd, *J* = 7.3, 7.3 Hz, 1H), 6.49 (d, *J* = 7.8 Hz, 1H), 3.05 (d, *J* = 12.5 Hz, 1H), 2.78 (s, 3H), 2.65 (d, *J* = 16.6 Hz, 1H), 2.59 (d, *J* = 16.6 Hz, 1H), 2.48 (m, 1H), 2.30 (s, 3H), 2.30 (m, 1H, buried), 2.23 (m, 1H), 1.94 (ddd, *J* = 14.2, 3.4, 3.4 Hz, 1H), 1.81 (ddd, *J* = 14.5, 9.9, 4.5 Hz, 1H).

^{13}C NMR (151 MHz, $CDCl_3$): δ 176.8, 149.3, 134.4, 128.7, 122.2, 119.1, 108.7, 85.8, 58.9, 51.2, 47.4, 46.1, 41.1, 32.6, 29.0.

HRMS (ESI): calc. for $C_{15}H_{20}N_3O$ [M+H]+: 258.1601, found: 258.1614.

MP: 193 - 195 °C.

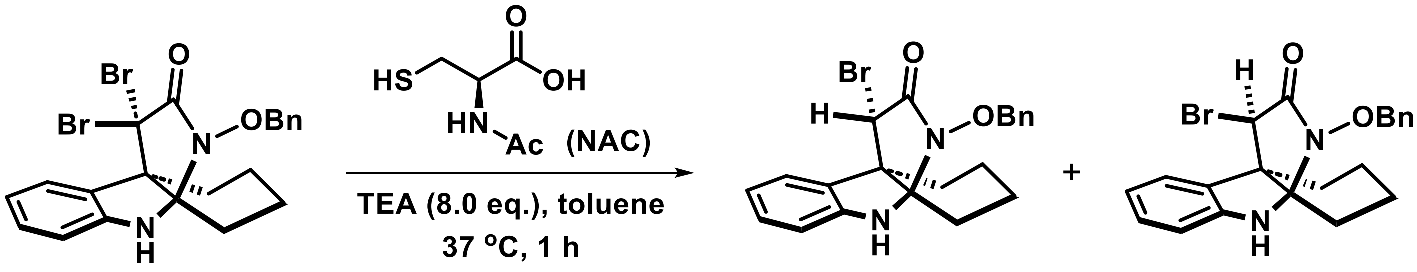

18 **26 (69% yield)** **27 (31% yield)**

Procedure. Compound **18** (50.8 mg, 0.10 mmol) was added to a flame-dried round-bottom flask and dissolved in toluene (3 mL). *N*-acetyl-L-cysteine (NAC; 81.6 mg, 0.50 mmol) was then added to the reaction in one portion before triethylamine (112 μL, 0.80 mmol) was added to the mixture drop-wise. The resulting mixture was then warmed to 37 °C and allowed to stir for 1 hour. Upon completion, the reaction was quenched with water and extracted with ethyl acetate. The resulting organic layers were then combined, dried with sodium sulfate, filtered, and concentrated under reduced pressure. Finally, the crude diastereomeric products were purified via column chromatography using hexanes:ethyl acetate (ramping hexanes:ethyl acetate mixtures from 1:0 to 9:1 to 4:1 hexanes:ethyl acetate) to afford each diastereomer pure (29.4 mg of compound **26** was isolated as a white solid, 69% yield; 13.1 mg of compound **27** was isolated as a white solid, 31% yield). Note: The stereochemical configuration of each diastereomer was determined by NOE experiments of **26** and **27** (see spectra for details).

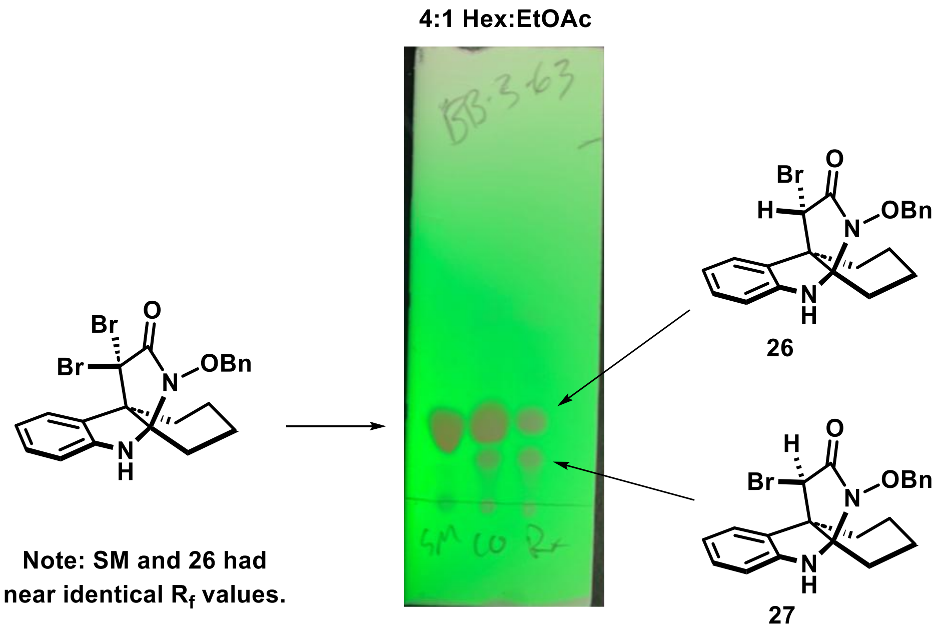

Note: The TLC image was taken by Dr. Beau Brummel.

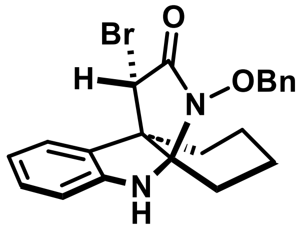

26

^{1}H NMR (400 MHz, CDCl$_3$): δ 7.45 - 7.35 (m, 5H), 7.08 (ddd, *J* = 7.6, 7.6, 1.0 Hz, 1H), 7.06 (d, *J* = 7.6 Hz, 1H), 6.80 (ddd, *J* = 7.6, 7.6, 0.7 Hz, 1H), 6.49 (d, *J* = 7.6 Hz, 1H), 5.09 (d, *J* = 10.6 Hz, 1H), 5.05 (d, *J* = 10.6 Hz, 1H), 4.73 (s, 1H), 4.22 (s, 1H), 2.24 (ddd, *J* = 14.5, 4.8, 4.8 Hz, 1H), 2.09 (ddd, *J* = 13.7, 4.3, 4.3 Hz, 1H), 1.87 - 1.73 (m, 2H), 1.69 - 1.54 (m, 2H), 1.51 - 1.32 (m, 2H).

^{13}C NMR (151 MHz, CDCl$_3$): δ 165.1, 146.0, 135.3, 133.8, 129.7, 129.4, 129.3, 128.9, 123.1, 120.5, 111.1, 85.3, 78.5, 51.3, 49.9, 33.5, 31.2, 20.9, 20.0.

HRMS (ESI): calc. for $C_{21}H_{22}BrN_2O_2$ $[M + H]^+$: 413.0859 / 415.0841, found: 413.0854 / 415.0837 (1:1 isotope cluster).

MP: 63 - 65 °C.

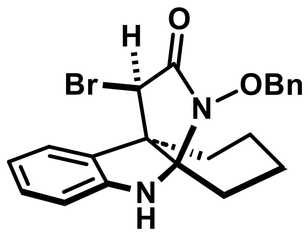

27

^{1}H NMR (600 MHz, CDCl$_3$): δ 7.57 (d, *J* = 7.6 Hz, 1H), 7.42 - 7.36 (m, 3H), 7.36 - 7.32 (m, 2H), 7.06 (ddd, *J* = 7.6, 7.6, 1.1 Hz, 1H), 6.80 (ddd, *J* = 7.6, 7.6, 0.8 Hz, 1H), 6.34 (d, *J* = 7.8 Hz, 1H), 5.14 (d, *J* = 11.5 Hz, 1H), 4.82 (d, *J* = 11.5 Hz, 1H), 4.77 (s, 1H), 3.91 (s, 1H), 2.23 (dddd, *J* = 14.4, 3.8, 3.8, 1.0 Hz, 1H), 2.17 (dddd, *J* = 14.6, 4.3, 4.3, 1.0 Hz, 1H), 1.69 - 1.51 (m, 3H), 1.43 - 1.23 (m, 3H).

^{13}C NMR (151 MHz, CDCl$_3$): δ 165.1, 146.6, 136.0, 133.0, 129.3, 129.1, 129.0, 128.6, 126.6, 119.7, 111.0, 84.7, 78.7, 51.2, 48.9, 32.9, 31.7, 20.6, 20.2.

HRMS (DART): calc. for $C_{21}H_{22}BrN_2O_2$ $[M + H]^+$: 413.0859 / 415.0841, found: 413.0867 / 415.0848 (1:1 isotope cluster).

MP: 74 - 76 °C.

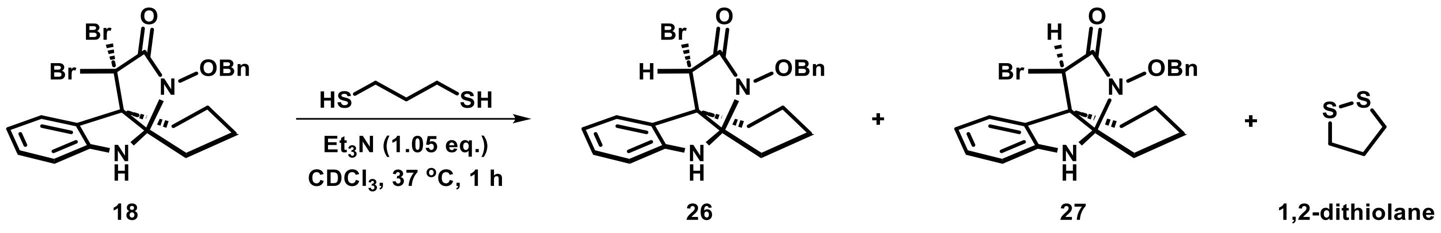

Procedure. Compound **18** (50 mg, 0.10 mmol) was added to a flame-dried round-bottom flask and dissolved in deuterated chloroform (1.5 mL). Then, 1,3-propanedithiol (10.5 µL, 0.105 mmol) was added to the reaction followed by the addition of triethylamine (14.6 µL, 0.105 mmol). The resulting mixture was warmed to 37 °C and allowed to stir for 1 hour. After this time, the reaction mixture was directly subjected to ^{1}H NMR analysis to confirm the formation of 1,2-dithiolane and percent yields for **26** (55%) and **27** (45%) were determined by ^{1}H NMR analysis (as no starting material remained).

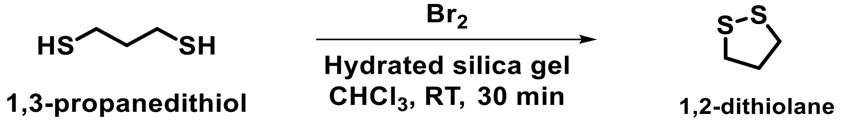

Procedure. Deionized water (0.6 mL) was added dropwise through a septum into a round bottom flask containing silica gel (1.0 g, chromatography grade) vigorously stirring. The resulting mixture was stirred for ~ 10 minutes until a free-floating powder was observed. Chloroform (12 mL) was then added to the resulting hydrated silica gel powder in the round bottom flask, which was then followed by the dropwise addition of 1,3-propanedithiol (0.1 mL, 1.0 mmol; dissolved in 1.3 mL chloroform). Next, a solution of bromine (50 µL, 1.0 mmol) dissolved in 1.3 mL chloroform was added to the reaction mixture dropwise. The characteristic brown color of molecular bromine disappeared as soon as it came in contact with the reaction mixture in the flask. The reaction was determined to be complete when a faint yellow color persisted (~ 20 minutes). Upon completion of the reaction, the solids were filtered off through a sintered glass funnel. Following this, 0.2 mL of the filtrate was mixed with 0.2 mL deuterated chloroform to obtain ^{1}H and ^{13}C NMR spectra for analysis. Notes: The reaction mixture was concentrated via rotovap to afford the crude 1,2-dithiolane as clear oil (73 mg, 69% yield; ≥ 93% pure). This procedure was used for the synthesis of 1,2-dithiolane, which was used as a reference compound during the course of these studies.[3] 1,2-Dithiolane is a known compound (CAS No. 557-22-2) and our spectral data for this molecule aligns with those previously reported (NMR solvents to previously report 1,2-dithiolane include: $CDCl_3$ and CD_2Cl_2).[4,5]

^{1}H NMR (600 MHz, $CDCl_3$): δ 3.13 (t, *J* = 6.6 Hz, 4H), 2.29 (p, *J* = 6.6 Hz, 2H).

^{13}C NMR (151 MHz, $CDCl_3$): δ 39.1, 33.7.

21.) X-Ray Protocols & Data.

X-Ray Analysis of 12. X-Ray Intensity data were collected at 100 K on a Bruker Dual micro source D8 Venture diffractometer and PHOTON III detector running APEX3 software package of programs and using MoKα radiation (λ = 0.71073 Å). The data frames were integrated and multi-scan scaling was applied in APEX3. Intrinsic phasing structure solution provided all of the non-H atoms. The structure was refined using full-matrix least-squares refinement.[6] The non-H atoms were refined with anisotropic displacement parameters and all of the H atoms were calculated in idealized positions and refined riding on their parent atoms. The absolute structure is established by the Flack x parameter of -0.001(16). In addition to the molecule, there is a water solvent molecule in the asymmetric unit. Its protons were obtained from a Difference Fourier map and along with the oxygen were refined as a rigid group. In the final cycle of refinement, 9522 reflections (of which 8960 are observed with $I > 2\sigma(I)$) were used to refine 384 parameters and the resulting R_1, wR_2 and S (goodness of fit) were 3.37%, 8.44% and 1.029, respectively. The refinement was carried out by minimizing the wR_2 function using F^2 rather than F values. R_1 is calculated to provide a reference to the conventional R value but its function is not minimized.

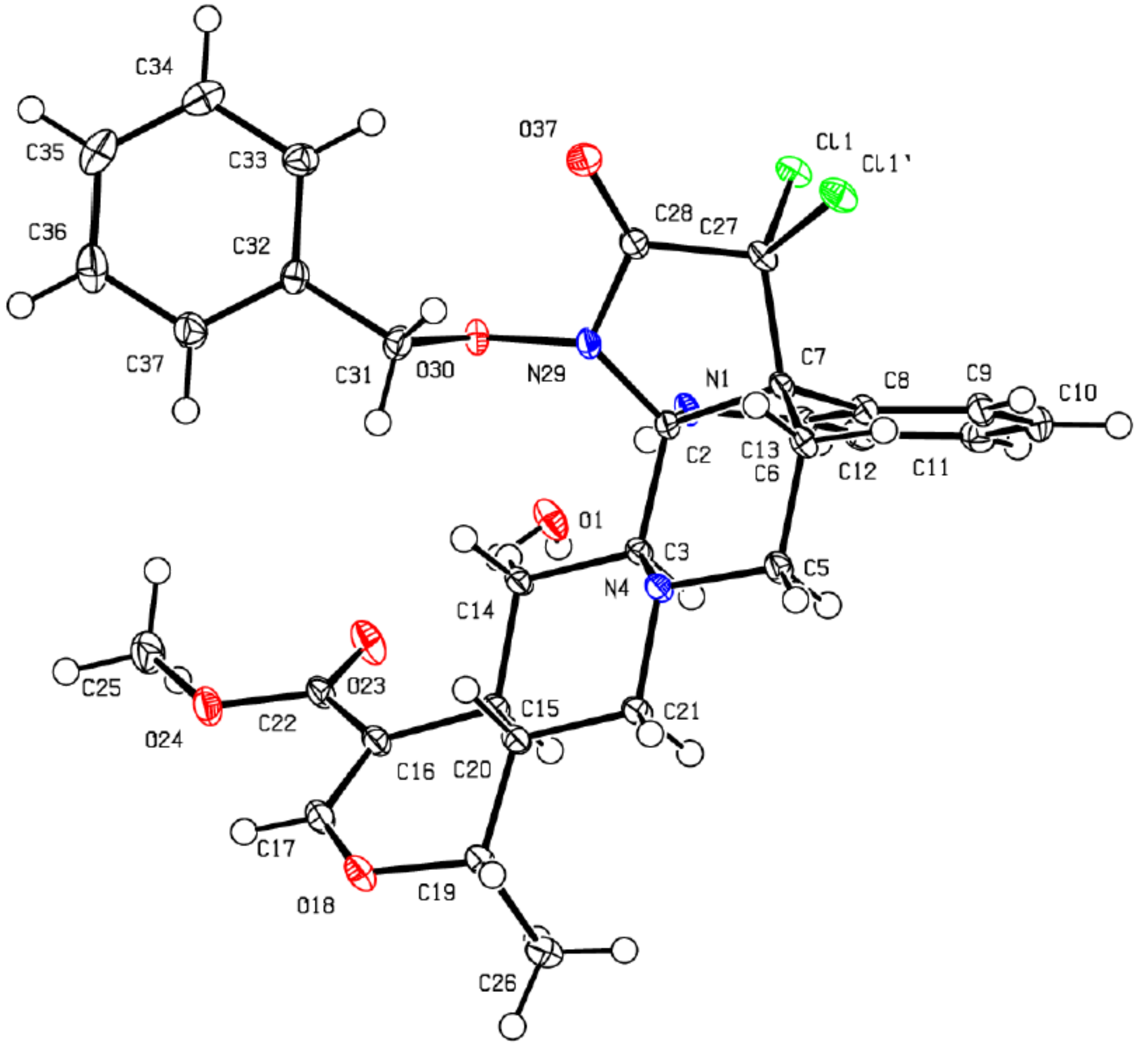

Raw crystal data and atomic numbering for **12**.

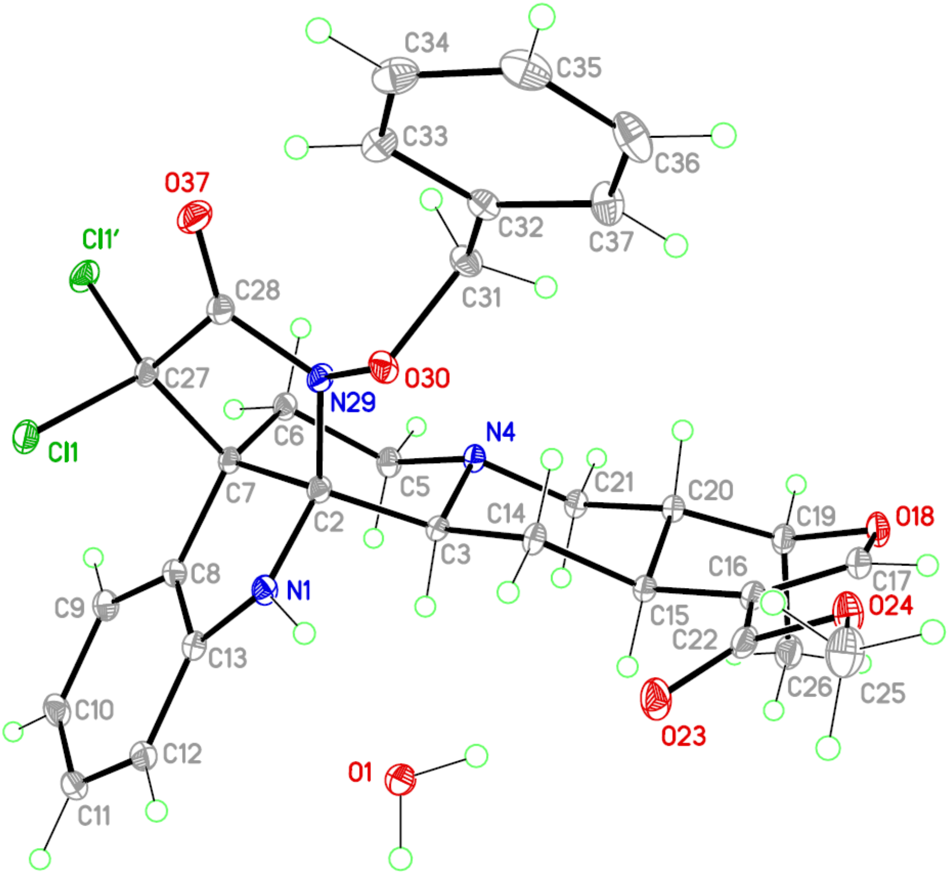

ORTEP of compound **12**.

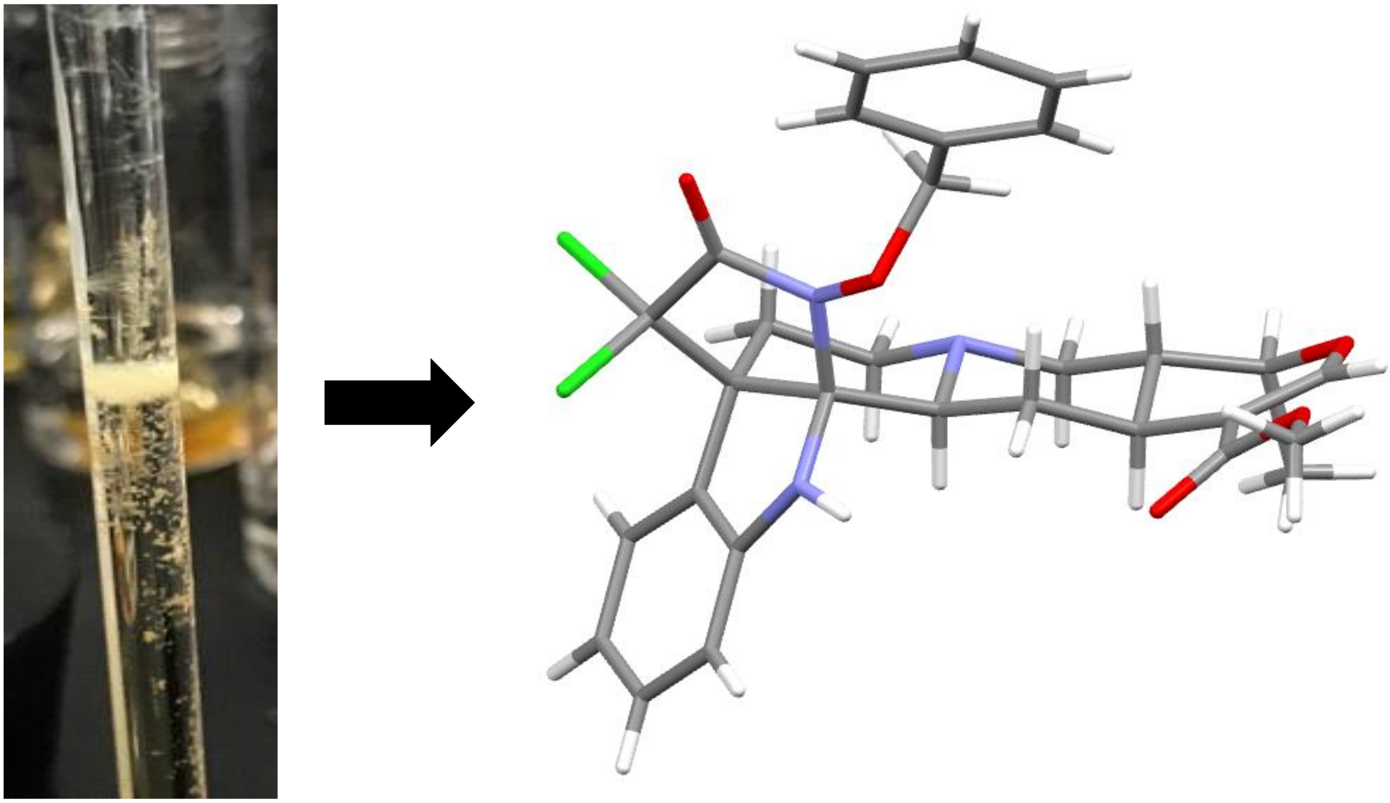

Note: Image of **12** crystals (crystal formation at liquid-liquid interface) was taken by Dr. Srinivasarao Tenneti.

Crystallographic data for **12** has been deposited with the Cambridge Crystallographic Data Centre (CCDC deposition number 2426868). Copies of the data can be obtained at http://www.ccdc.cam.ac.uk/.

Crystal data and structure refinement for compound **12**.

Identification code	st275b (compound **12**)	
Empirical formula	$C_{30}H_{33}Cl_2N_3O_6$ (includes H_2O within crystal)	
Formula weight	602.49	
Temperature	100(2) K	
Wavelength	0.71073 Å	
Crystal system	Orthorhombic	
Space group	$P2_12_12_1$	
Unit cell dimensions	a = 9.2614(3) Å	α = 90°.
	b = 13.3002(3) Å	β = 90°.
	c = 22.8643(7) Å	γ = 90°.
Volume	2816.39(14) $Å^3$	
Z	4	
Density (calculated)	1.421 Mg/m^3	
Absorption coefficient	0.281 mm^{-1}	
F(000)	1264	
Crystal size	0.541 x 0.328 x 0.232 mm^3	
Theta range for data collection	2.349 to 32.813°.	
Index ranges	$-13 \le h \le 13$, $-19 \le k \le 19$, $-34 \le l \le 33$	
Reflections collected	75148	
Independent reflections	9522 [R(int) = 0.1048]	
Completeness to theta = 25.242°	98.9 %	
Absorption correction	Semi-empirical from equivalents	
Max. and min. transmission	0.9475 and 0.9173	
Refinement method	Full-matrix least-squares on F^2	
Data / restraints / parameters	9522 / 0 / 379	
Goodness-of-fit on F^2	1.029	
Final R indices [I>2sigma(I)]	R1 = 0.0337, wR2 = 0.0844 [8960]	
R indices (all data)	R1 = 0.0361, wR2 = 0.0859	
Absolute structure parameter	0.001(16)	
Extinction coefficient	n/a	
Largest diff. peak and hole	0.364 and -0.428 e.$Å^{-3}$	

X-Ray Analysis of 13. X-Ray Intensity data were collected at 100 K on a Bruker Dual micro source D8 Venture diffractometer and PHOTON III detector running APEX3 software package of programs and using MoKα radiation (λ = 0.71073 Å). The data frames were integrated and multi-scan scaling was applied in APEX3. Intrinsic phasing structure solution provided all of the non-H atoms. The structure was refined using full-matrix least-squares refinement.[6] The non-H atoms were refined with anisotropic displacement parameters and all of the H atoms were calculated in idealized positions and refined riding on their parent atoms. In addition to the molecule, there is a disordered chloroform solvent molecule in the asymmetric unit. The chlorine atoms were disordered and refined in three sets of atoms. The absolute configuration is determined by anomalous dispersion. In the final cycle of refinement, 11435 reflections (of which 10606 are observed with $I > 2\sigma(I)$) were used to refine 460 parameters and the resulting R_1, wR_2 and S (goodness of fit) were 2.81%, 6.58% and 1.029, respectively. The refinement was carried out by minimizing the wR_2 function using F^2 rather than F values. R_1 is calculated to provide a reference to the conventional R value but its function is not minimized.

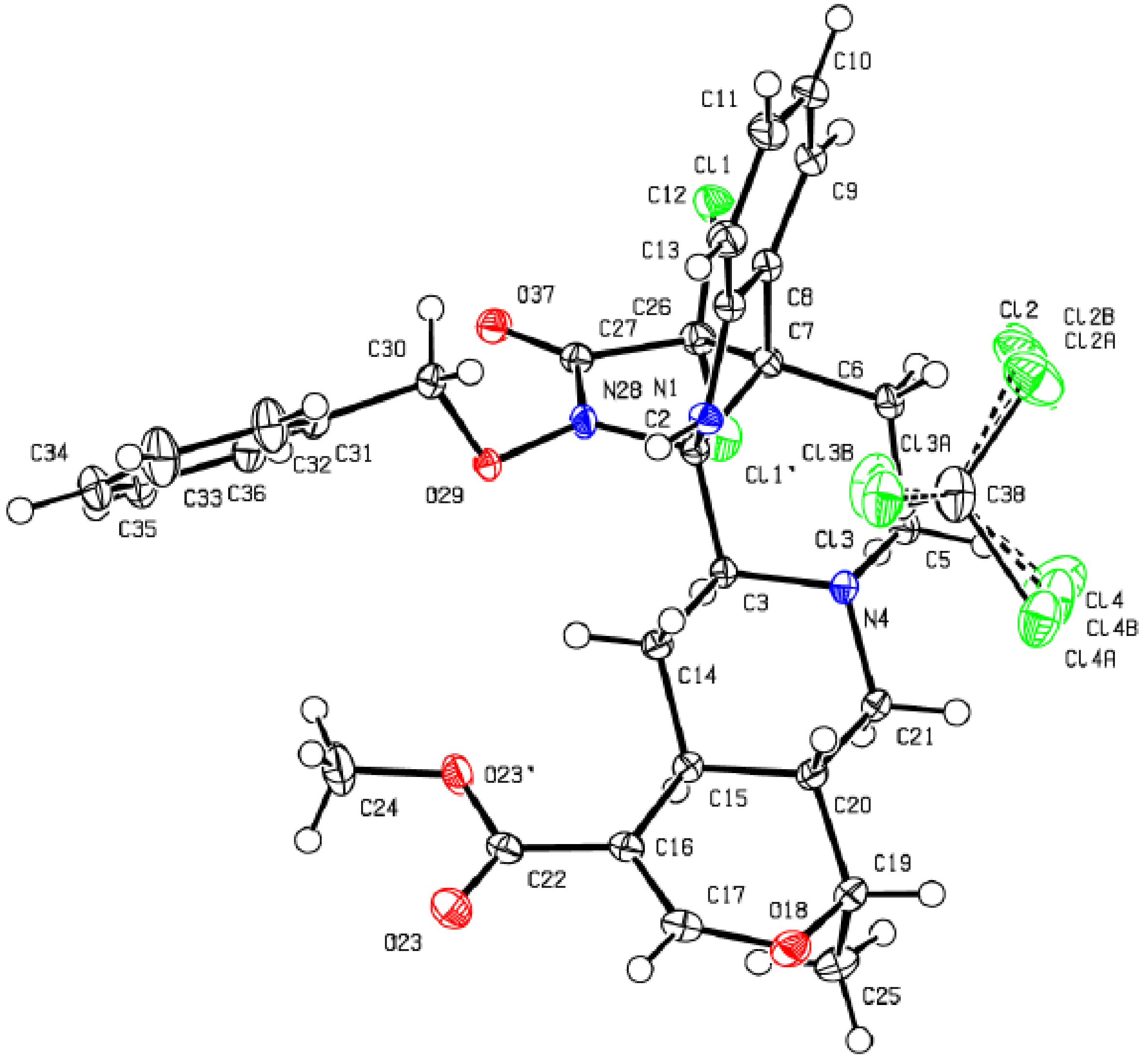

Raw crystal data and atomic numbering for **13**.

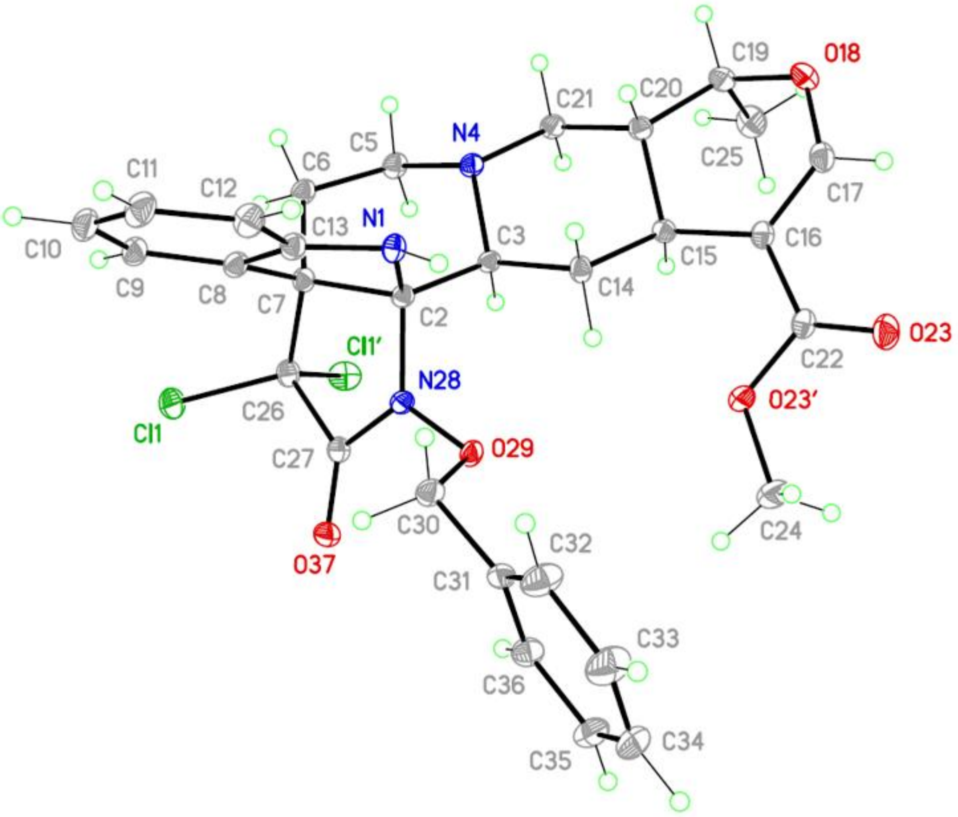

ORTEP of compound **13**.

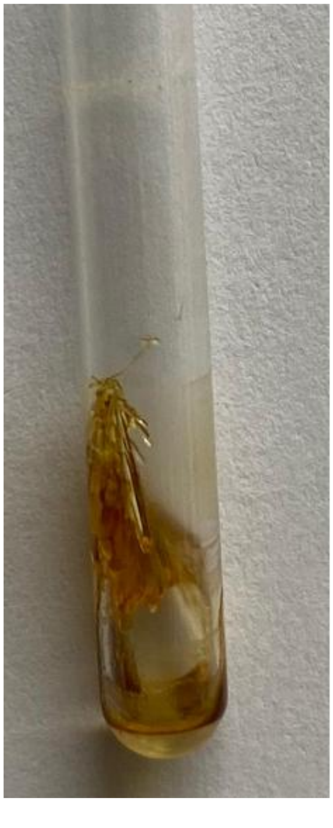

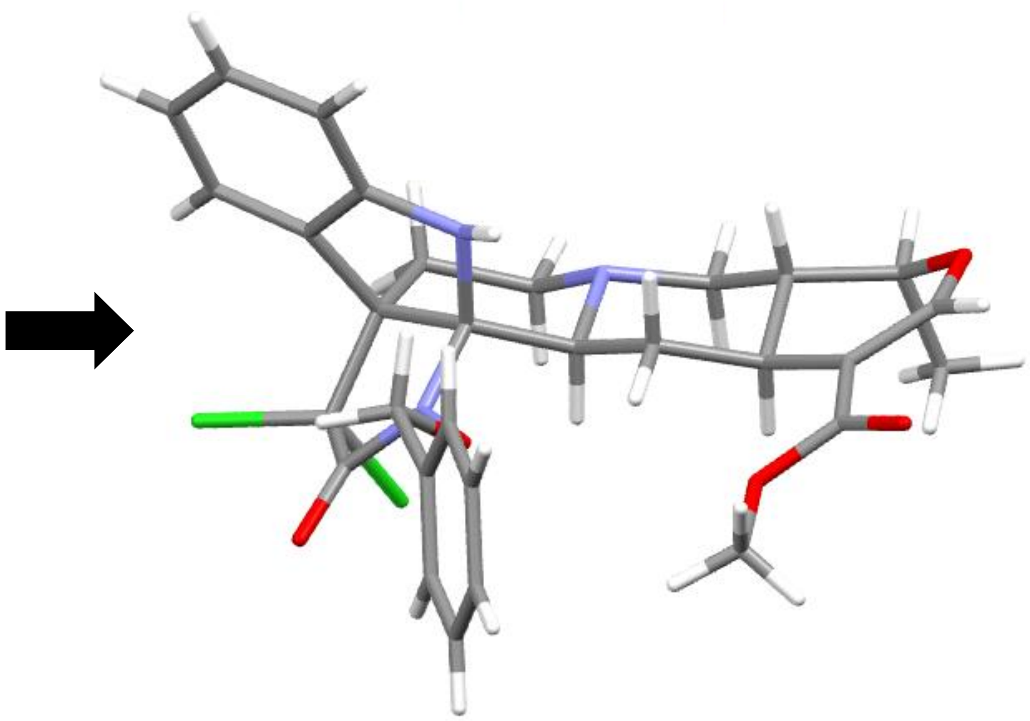

Note: Image of **13** crystals (crystal formation via slow evaporation) was taken by Dr. Srinivasarao Tenneti.

Crystallographic data for **13** has been deposited with the Cambridge Crystallographic Data Centre (CCDC deposition number 2426869). Copies of the data can be obtained at http://www.ccdc.cam.ac.uk/.

Crystal data and structure refinement for compound **13**.

Identification code	st275a (compound **13**)	
Empirical formula	$C_{31}H_{32}Cl_5N_3O_5$ (includes $CHCl_3$ within crystal)	
Formula weight	702.78	
Temperature	100(2) K	
Wavelength	0.71073 Å	
Crystal system	Orthorhombic	
Space group	P 21 21 21	
Unit cell dimensions	a = 9.1065(2) Å	α = 90°.
	b = 17.6230(5) Å	β = 90°.
	c = 20.4364(6) Å	γ = 90°.
Volume	3279.71(15) $Å^3$	
Z	4	
Density (calculated)	1.423 Mg/m^3	
Absorption coefficient	0.484 mm^{-1}	
F(000)	1454	
Crystal size	1.7x 1.1x0.9 mm^3	
Theta range for data collection	2.304 to 33.524°.	
Index ranges	$-13 \le h \le 14$, $-23 \le k \le 26$, $-31 \le l \le 29$	
Reflections collected	52048	
Independent reflections	11435 [R(int) = 0.0330]	
Completeness to theta = 25.242°	99.4 %	
Absorption correction	multi-scan	
Refinement method	Full-matrix least-squares on F^2	
Data / restraints / parameters	11435 / 1 / 460	
Goodness-of-fit on F^2	1.029	
Final R indices [I>2sigma(I)]	R1 = 0.0281, wR2 = 0.0658 [10606]	
R indices (all data)	R1 = 0.0322, wR2 = 0.0684	
Absolute structure parameter	-0.016(10)	
Extinction coefficient	n/a	
Largest diff. peak and hole	0.294 and -0.190 e.$Å^{-3}$	

X-Ray Analysis of 14. X-Ray Intensity data were collected at 100 K on a Bruker Dual micro source D8 Venture diffractometer and PHOTON III detector running APEX3 software package of programs and using MoKα radiation (λ = 0.71073 Å). The data frames were integrated and multi-scan scaling was applied in APEX3. Intrinsic phasing structure solution provided all of the non-H atoms. The structure was refined using full-matrix least-squares refinement.[6] The non-H atoms were refined with anisotropic displacement parameters and all of the H atoms were calculated in idealized positions and refined riding on their parent atoms. In addition to the molecule, there is a disordered chloroform solvent molecule in the asymmetric unit. The chlorine atoms were disordered and refined in three sets of atoms. The absolute configuration is determined by anomalous dispersion. In the final cycle of refinement, 8248 reflections (of which 7253 are observed with I > 2σ(I)) were used to refine 415 parameters and the resulting R_1, wR_2 and S (goodness of fit) were 3.43%, 9.63% and 0.765, respectively. The refinement was carried out by minimizing the wR_2 function using F^2 rather than F values. R_1 is calculated to provide a reference to the conventional R value but its function is not minimized.

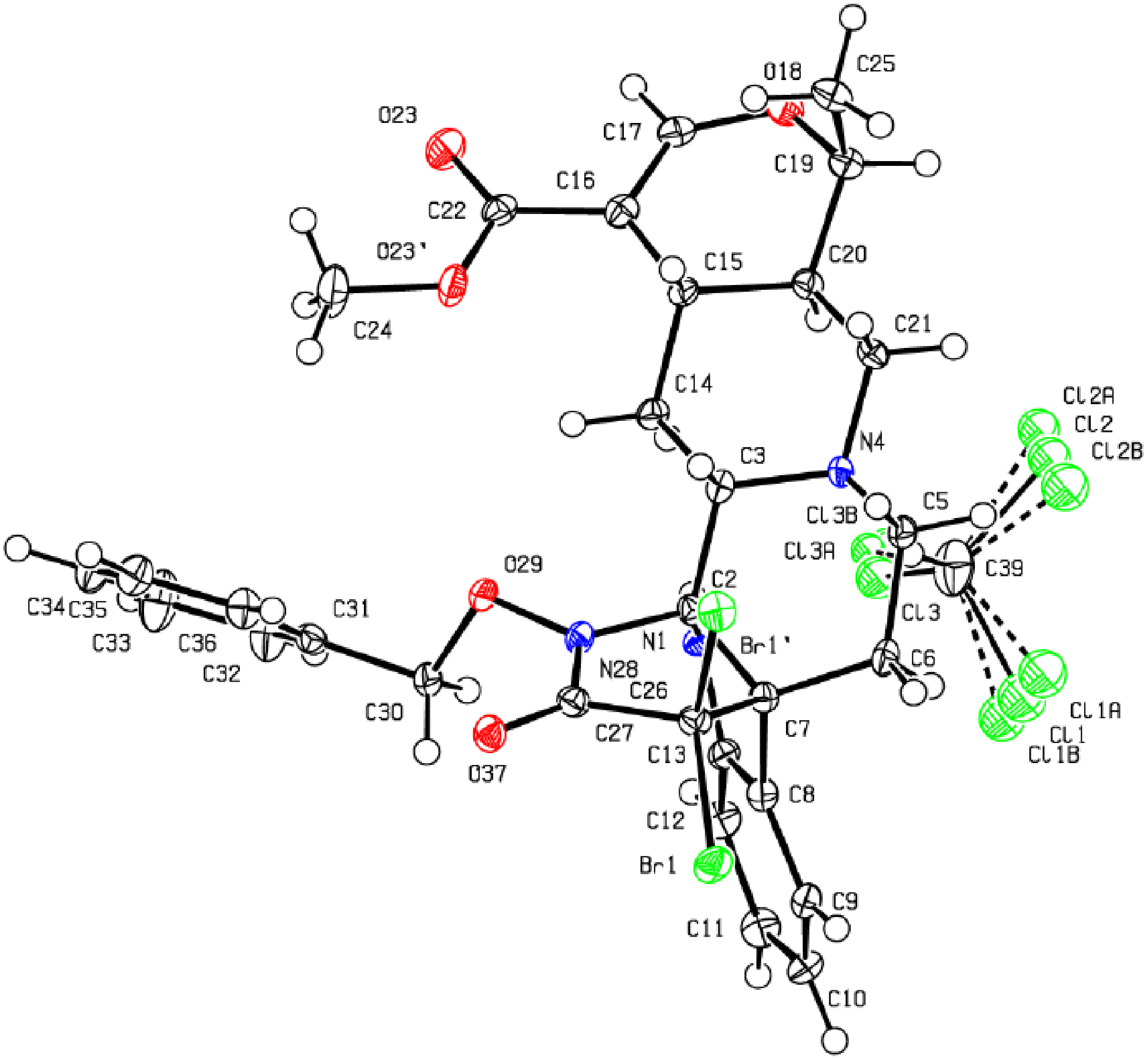

Raw crystal data and atomic numbering for **14**.

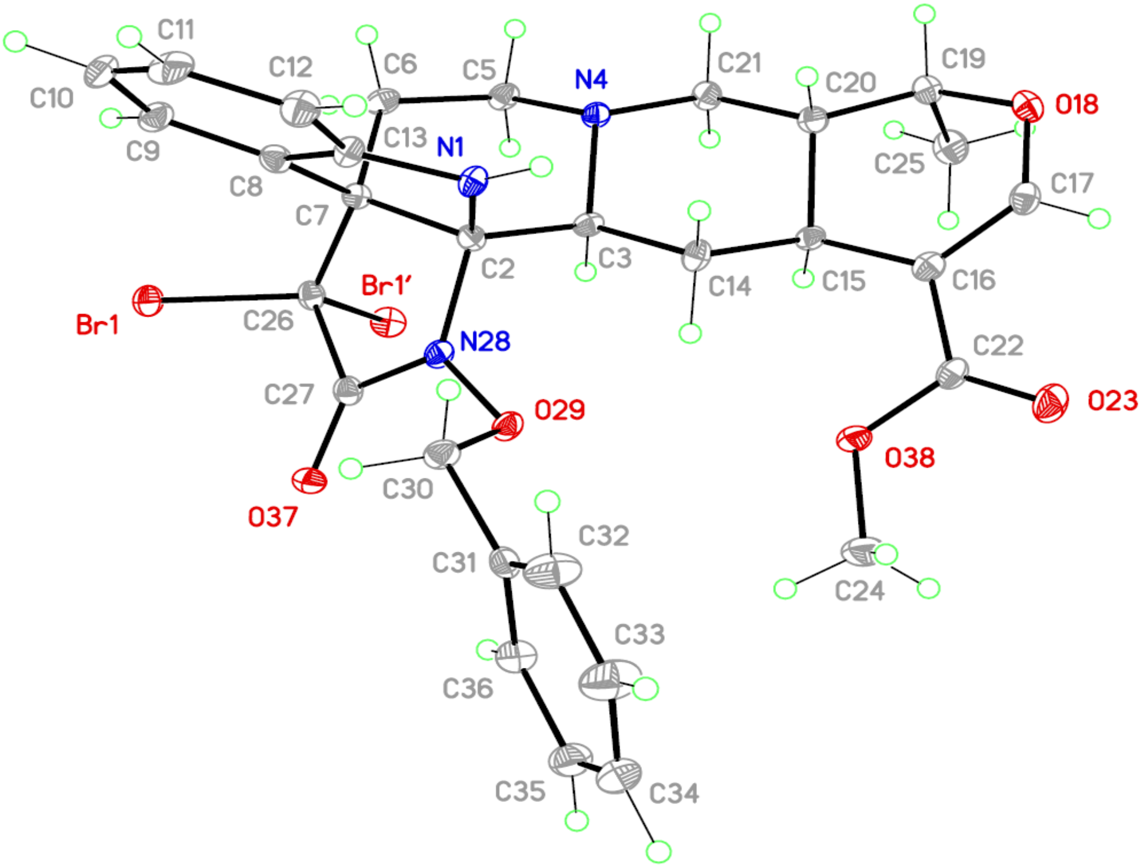

ORTEP of compound **14**.

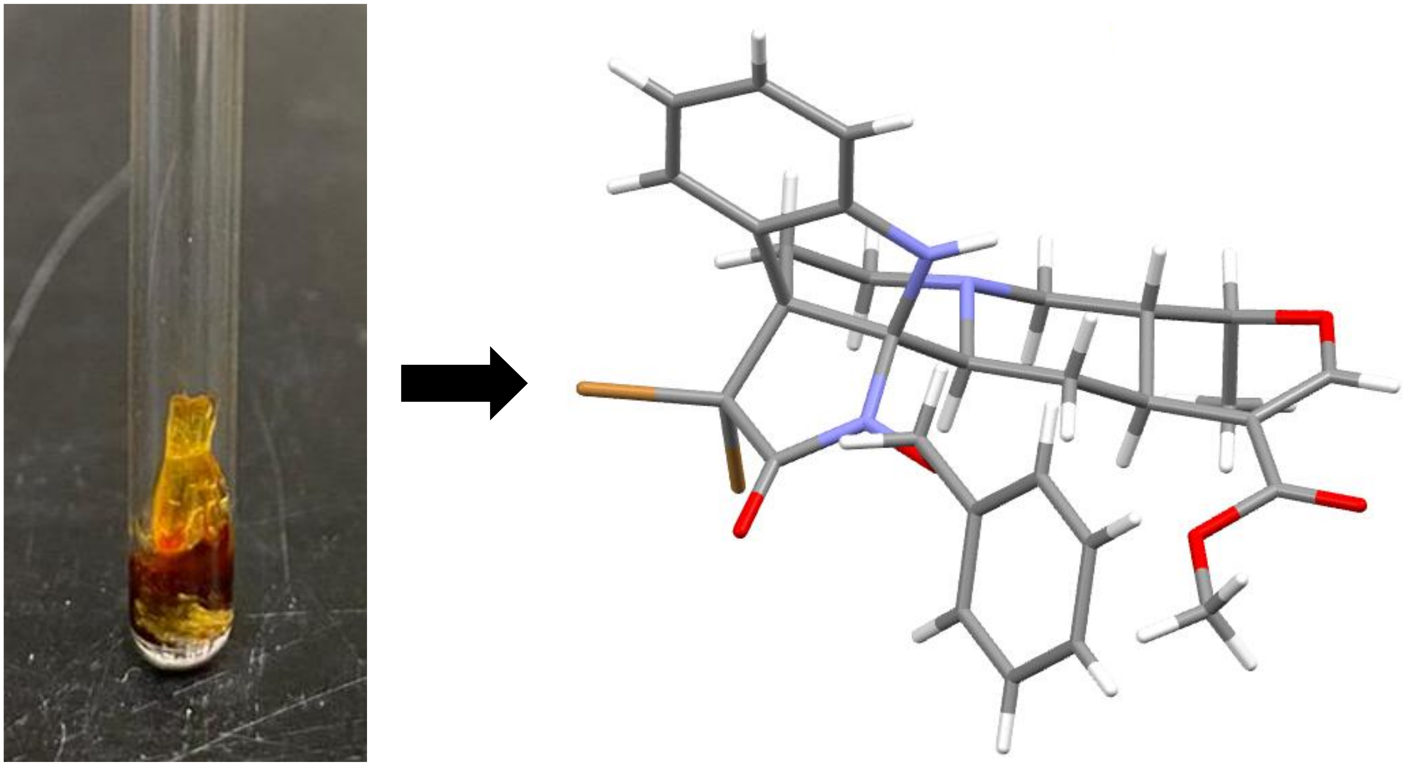

Note: Image of **14** crystals (crystal formation via slow evaporation) was taken by Dr. Srinivasarao Tenneti.

Crystallographic data for **14** has been deposited with the Cambridge Crystallographic Data Centre (CCDC deposition number 2426867). Copies of the data can be obtained at http://www.ccdc.cam.ac.uk/.

Crystal data and structure refinement for compound **14**.

Identification code	st2157a (compound **14**)	
Empirical formula	$C_{31}H_{32}Cl_3Br_2N_3O_5$ (includes $CHCl_3$ within crystal)	
Formula weight	792.59	
Temperature	100(2) K	
Wavelength	0.71073 Å	
Crystal system	Orthorhombic	
Space group	P 21 21 21	
Unit cell dimensions	a = 9.1202(3) Å	α = 90°.
	b = 17.8336(8) Å	β = 90°.
	c = 20.3905(8) Å	γ = 90°.
Volume	3316.4(2) Å^3	
Z	4	
Density (calculated)	1.587 Mg/m^3	
Absorption coefficient	2.729 mm^{-1}	
F(000)	1600	
Crystal size	1.000 x 0.800 x 0.600 mm^3	
Theta range for data collection	2.284 to 28.305°.	
Index ranges	$-12 \le h \le 12$, $-23 \le k \le 23$, $-27 \le l \le 27$	
Reflections collected	87185	
Independent reflections	8248 [R(int) = 0.1313]	
Completeness to theta = 25.242°	99.9 %	
Absorption correction	multi-scan	
Refinement method	Full-matrix least-squares on F^2	
Data / restraints / parameters	8248 / 1 / 415	
Goodness-of-fit on F^2	0.765	
Final R indices [I>2sigma(I)]	R1 = 0.0343, wR2 = 0.0963 [7253]	
R indices (all data)	R1 = 0.0429, wR2 = 0.1076	
Absolute structure parameter	-0.008(5)	
Extinction coefficient	n/a	
Largest diff. peak and hole	0.573 and -0.578 e.Å^{-3}	

X-Ray Analysis of 27. A specimen of $C_{21}H_{21}BrN_2O_2$ (**27**), approximate dimensions 0.030 mm x 0.280 mm x 0.350 mm in a pale-yellow crystal was used for the X-ray crystallographic analysis. The X-ray intensity data were measured at room temperature (299K) on a Bruker D8 Quest PHOTON 100 CMOS X-ray diffractometer system with Incoatec Microfocus Source (IμS) monochromated Mo Kα radiation (λ = 0.71073 Å, sealed tube) using phi and omega-scan technique.

The integration of the data using a triclinic unit cell yielded a total of 55431 reflections to a maximum θ angle of 27.00° (0.78 Å resolution), of which 3945 were independent (average redundancy 14.051, completeness = 99.9%, Rint = 11.82%, Rsig = 4.38%) and 3022 (76.60%) were greater than 2σ(F2). The final cell constants of a = 7.3848(4) Å, b = 10.8138(6) Å, c = 12.7670(6) Å, α = 105.211(3)°, β = 104.442(3)°, γ = 103.597(3)°, volume = 902.12(8) Å3, are based upon the refinement of the XYZ-centroids of 9615 reflections above 20 σ(I). The data were integrated with the manufacturer's SAINT software and corrected for absorption effects using the Multi-Scan method (SADABS). The calculated minimum and maximum transmission coefficients (based on crystal size) are 0.5218 and 0.7458.

The structure was solved and refined using the Bruker SHELXTL Software Package[6-8], using the space group P-1 (No. 2), with Z = 2 for the formula unit, $C_{21}H_{21}BrN_2O_2$. Non-hydrogen atoms were located from successive difference Fourier map calculations. In the final cycles of each refinement, all the non-hydrogen atoms were refined in anisotropic displacement parameters. All the hydrogen atom positions were calculated and allowed to ride on the carbon (or nitrogen) to which they are bonded assuming a C–H (or N-H) bond length of m Å (m = 0.930 for Ph-H group, m = 0.980 for CH groups, m = 0.970 for CH_2 groups, m = 0.860 for NH groups). Hydrogen atom temperature factors were fixed at n (n = 1.2 for Ph-H, CH, CH_2 and NH groups) times the isotropic temperature factors of the C (or N)-atoms to which they are bonded. The final anisotropic full-matrix least-squares refinement on F2 with 235 variables converged at R1 = 5.42%, for the observed data and wR2 = 15.46% for all data. The goodness-of-fit was 1.016. The largest peak in the final difference electron density synthesis was 0.617 e-/Å3 and the largest hole was -0.778 e-/Å3 with an RMS deviation of 0.084 e-/Å3. These largest residues are of no chemical significance. On the basis of the final model, the calculated density was 1.522 g/cm3 and F(000), 424 e-. The asymmetric unit contains one molecule with a formula of $C_{21}H_{21}BrN_2O_2$. There might be some inter-molecular H-bonding between the molecules. The efforts have been made to resolve as many alerts as possible generated by CheckCIF. The current highest alerts are at level C, which might be attributed to a little bit high thermal motion of some atoms in the molecule. Note: A suitable crystal of **27** was formed via vapor diffusion with hexanes (outer chamber) and chloroform (vial with compound, see next page for image).

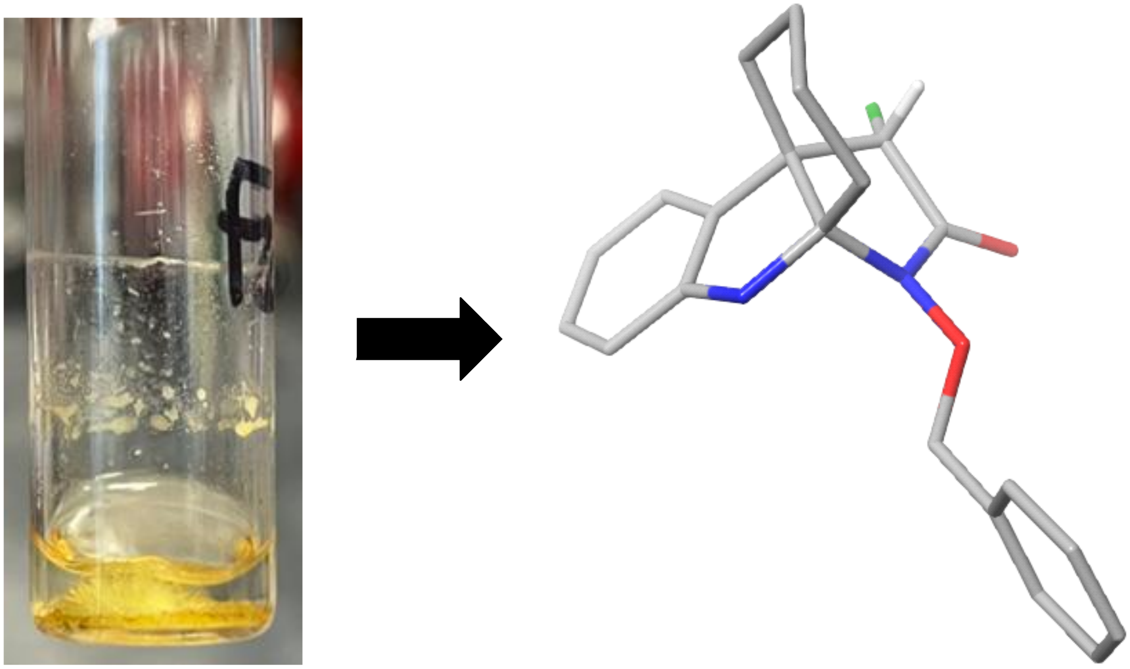

Note: Image of **27** crystals was taken by Dr. Qiwen Gao.

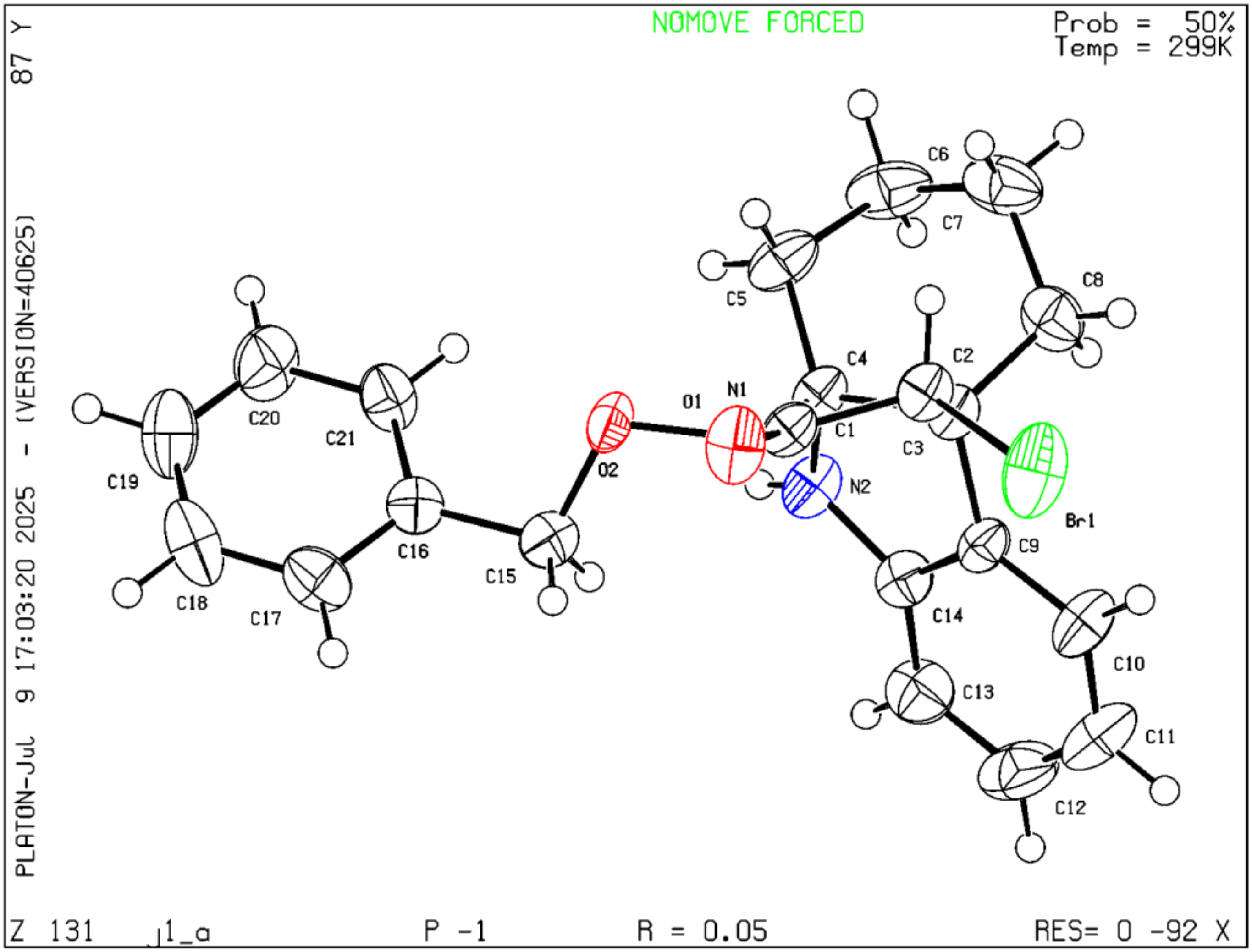

Ray crystal data and atomic numbering for **27**.

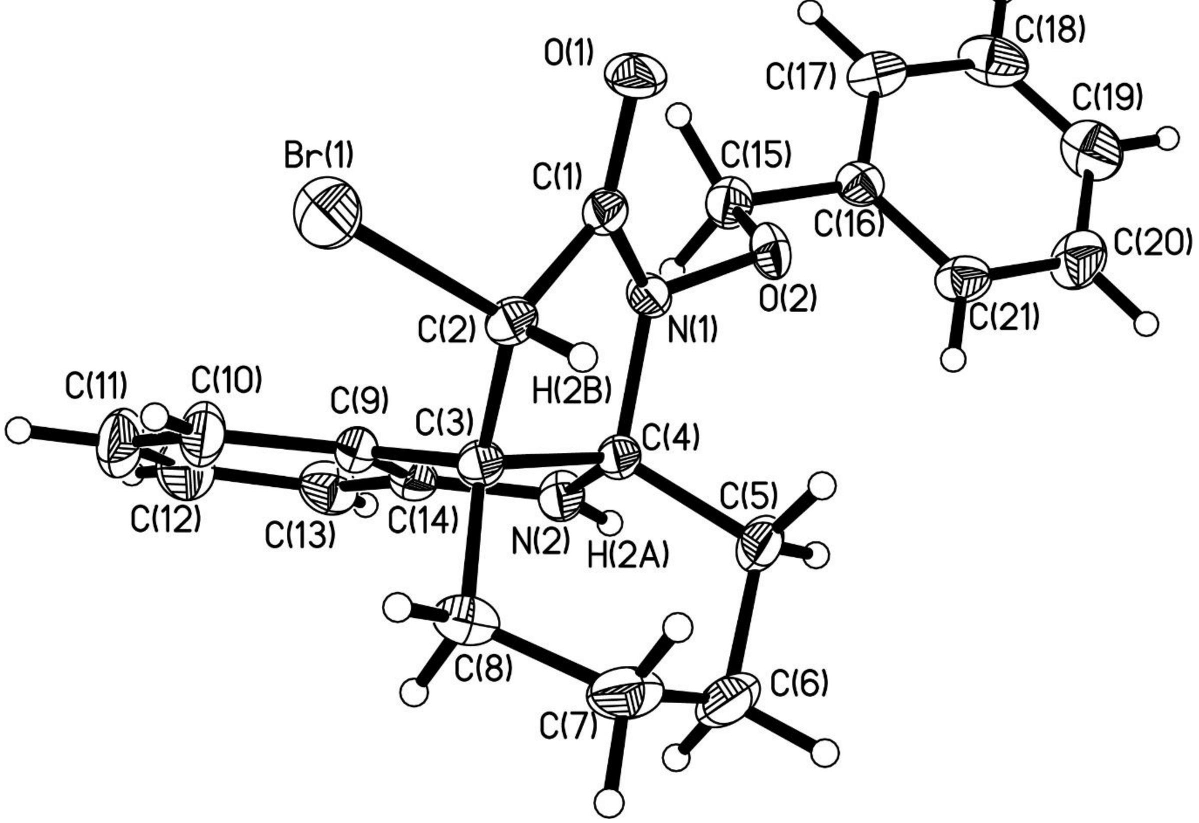

Ray crystal data and atomic numbering for **27**.

Crystallographic data for **27** has been deposited with the Cambridge Crystallographic Data Centre (CCDC deposition number 2472072). Copies of the data can be obtained at https://www.ccdc.cam.ac.uk/.

Crystal data and structure refinement for j1_a (compound **27**).

Identification code	j1_a (compound **27**)
Empirical formula	$C_{21}H_{21}BrN_2O_2$
Formula weight	413.31
Temperature	299(2) K
Wavelength	0.71073 A
Crystal system, space group	Triclinic, P-1
Unit cell dimensions	a = 7.3848(4) A alpha = 105.211(3) deg.
	b = 10.8138(6) A beta = 104.442(3) deg.
	c = 12.7670(6) A gamma = 103.597(3) deg.
Volume	902.12(8) A^3
Z, Calculated density	2, 1.522 Mg/m^3
Absorption coefficient	2.297 mm^-1
F(000)	424
Crystal size	0.350 x 0.280 x 0.030 mm
Theta range for data collection	2.204 to 26.999 deg.
Limiting indices	-9<=h<=9, -13<=k<=13, -16<=l<=16
Reflections collected / unique	55431 / 3945 [R(int) = 0.1182]
Completeness to theta	= 25.242 99.9 %
Absorption correction	Semi-empirical from equivalents
Max. and min. transmission	0.7458 and 0.5218
Refinement method	Full-matrix least-squares on F^2
Data / restraints / parameters	3945 / 0 / 235
Goodness-of-fit on F^2	1.016
Final R indices [I>2sigma(I)]	R1 = 0.0542, wR2 = 0.1370
R indices (all data)	R1 = 0.0737, wR2 = 0.1546
Extinction coefficient	n/a
Largest diff. peak and hole	0.617 and -0.778 e.A^-3

22.) References.

1.) Ji, W.; Yao, L.; Liao, X. Access to the pyrroloindoline core via [3 + 2] annulation as well as the application in the synthetic approach to (±)-minfiensine. *Org. Lett.* **2016**, *18*, 628-630.

2.) Ye, J.; Lin, Y.; Liu, Q.; Xu, D.; Wu, F.; Liu, B.; Gao, Y.; Chen, H. Biomimetic oxidative coupling cyclization enabling rapid construction of isochromanoindolenines. *Org. Lett.* **2018**, *20*, 5457-5460.

3.) Ali, M. H.; McDermott, M. Oxidation of thiols to disulfides with molecular bromine on hydrated silica gel support. *Tet. Lett.* **2002**, *43*, 6271-6273.

4.) Green, M.; Lown, E. M.; Strausz, O. P. Reactions of S atoms with dimethyl sulfide and thietane. *J. Am. Chem. Soc.* **1984**, *106*, 6938-6946.

5.) Cai, L.; Zeng, J.; Li, T.; Xiao, Y.; Ma, X.; Xiao, X.; Zhang, Q.; Meng, L.; Wan, Q. Dehydrative glycosylation enabled by a comproportionation reaction of 2-aryl-1,3-dithiane 1-oxide. *Chin. J. Chem.* **2020**, *38*, 43-49.

6.) Sheldrick, G. M. Crystal structure refinement with *SHELXL*. *Acta Cryst.* **2015**, *C71*, 3-8.

7.) Bruker AXS Inc. *APEX3 Crystallography Software Suite*. **2016**, Madison, Wisconsin, USA.

8.) Sheldrick, G.M. A short history of SHELXTL. *Acta Cryst*. **2008**, *A64*, 112.

23.) NMR Spectra.

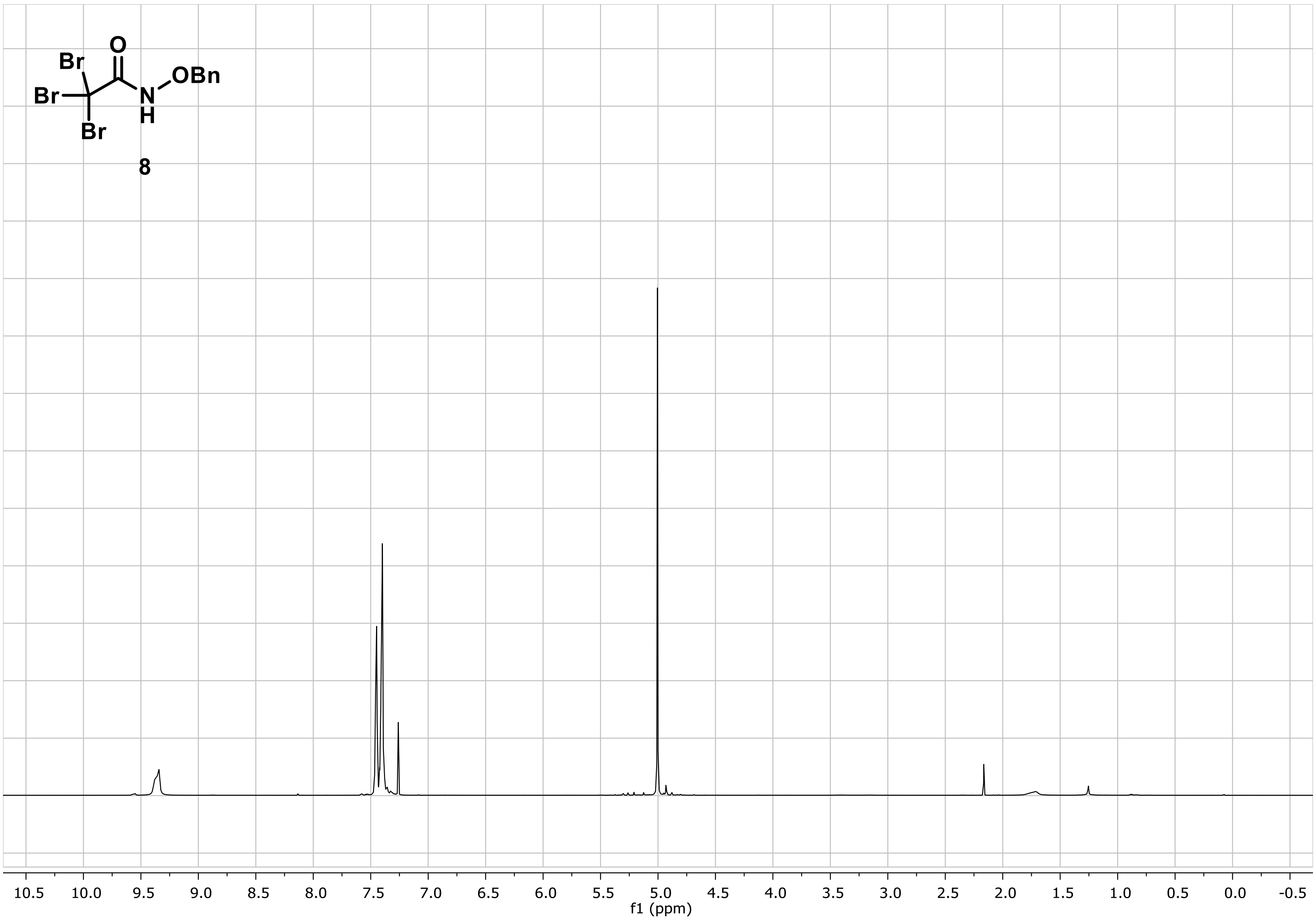
Br
O
OBn
Br
N
H
Br
8
10.5
10.0
9.5
9.0
8.5
8.0
7.5
7.0
6.5
6.0
5.5
5.0
4.5
4.0
3.5
3.0
2.5
2.0
1.5
1.0
0.5
0.0
-0.5
f1 (ppm)

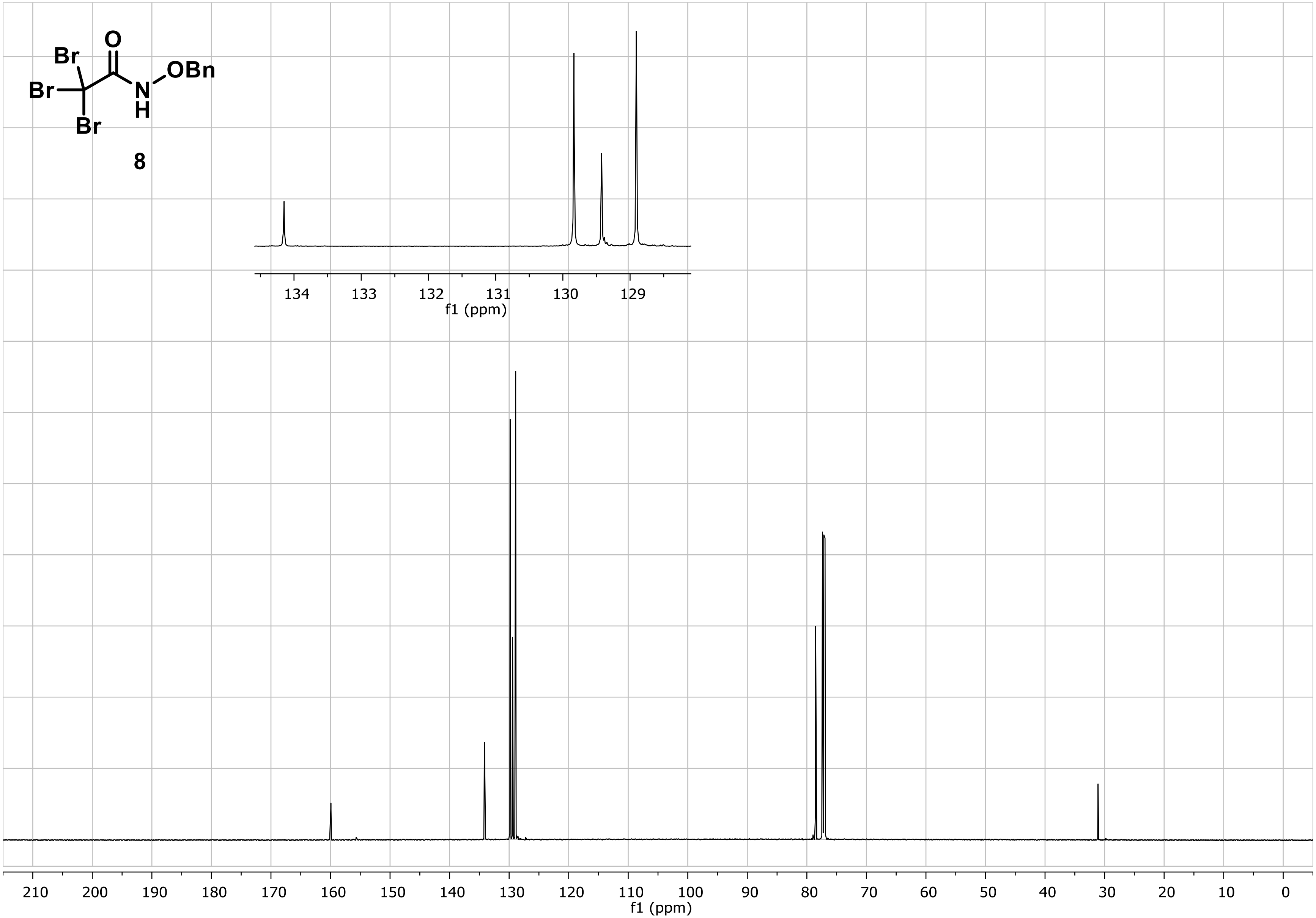

8
134
133
132
131
130
129
f1 (ppm)
210
200
190
180
170
160
150
140
130
120
110
100
90
80
70
60
50
40
30
20
10
0
f1 (ppm)

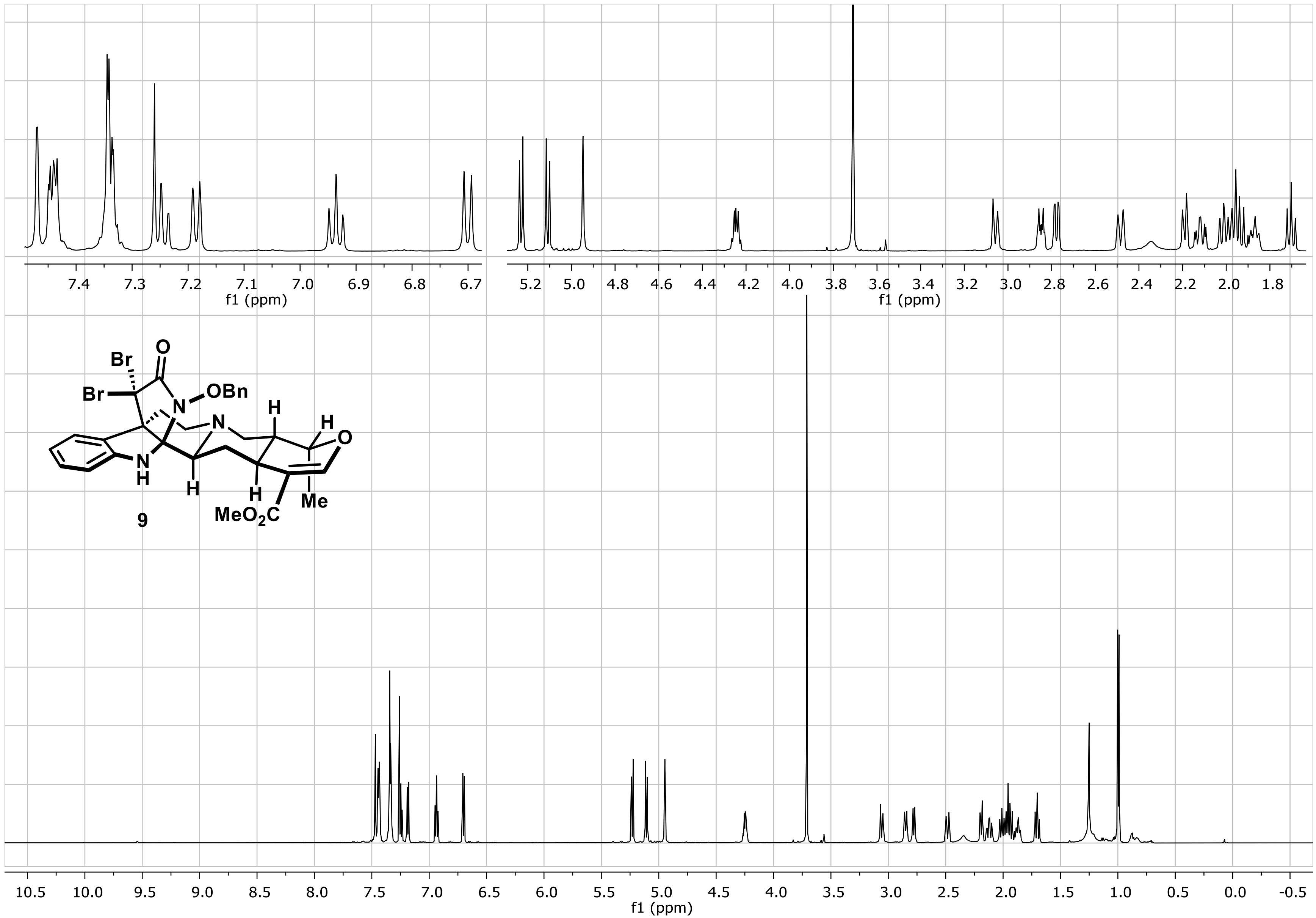
f1 (ppm)
Br
Br
O
N
OBn
N
H
H
O
NH
H
H
MeO2C
Me
9

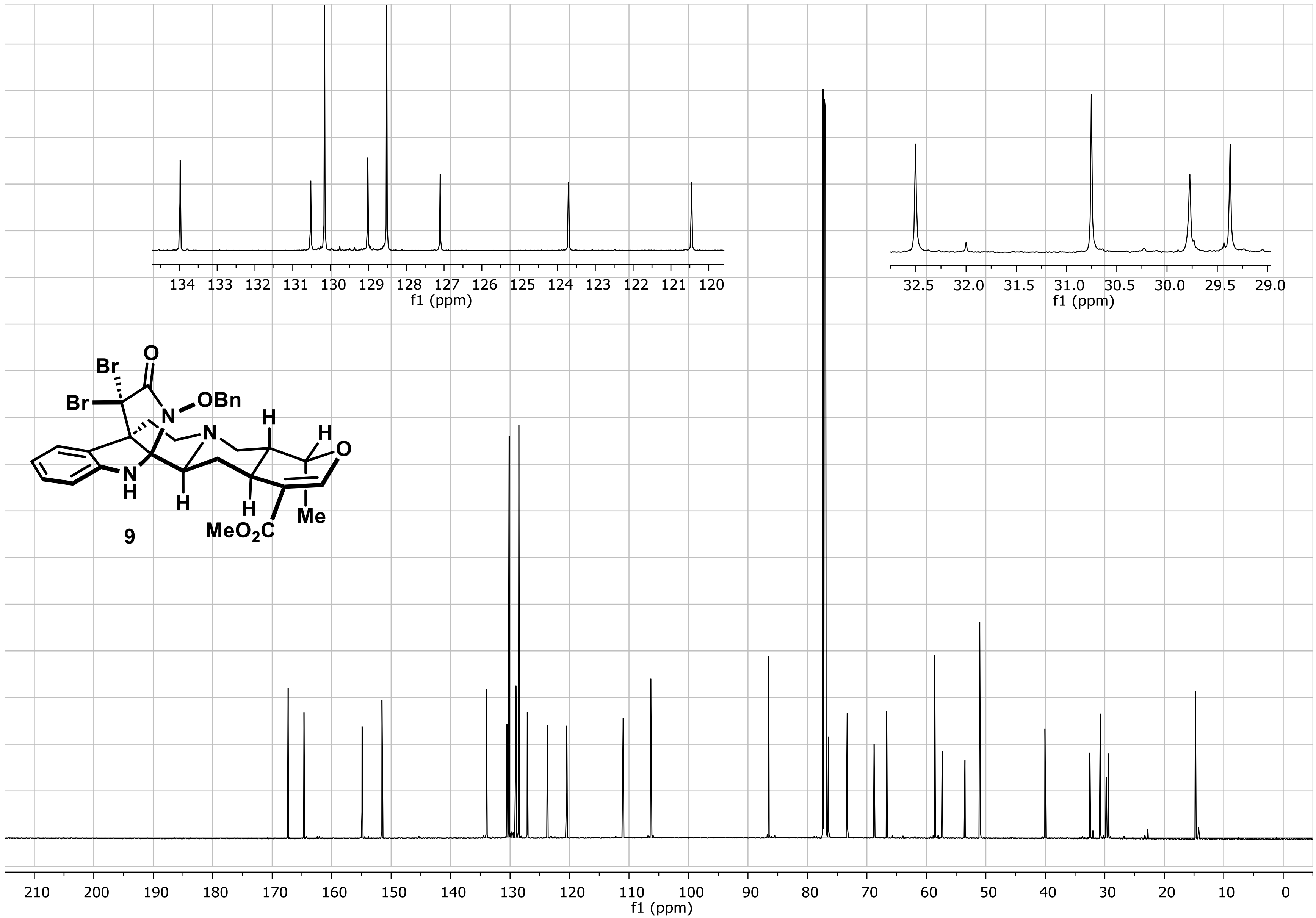
134 133 132 131 130 129 128 127 126 125 124 123 122 121 120
f1 (ppm)
32.5 32.0 31.5 31.0 30.5 30.0 29.5 29.0
f1 (ppm)
Br
Br
O
N
OBn
N
H
H
O
NH
H
H
H
Me
MeO$_2$C
9
210 200 190 180 170 160 150 140 130 120 110 100 90 80 70 60 50 40 30 20 10 0
f1 (ppm)

NOTE: *In JMOD quaternary (-C-) and methylene (-CH_2-) signals have opposite phase to those of methine (-CH-) and methyl (-CH_3) resonances.*

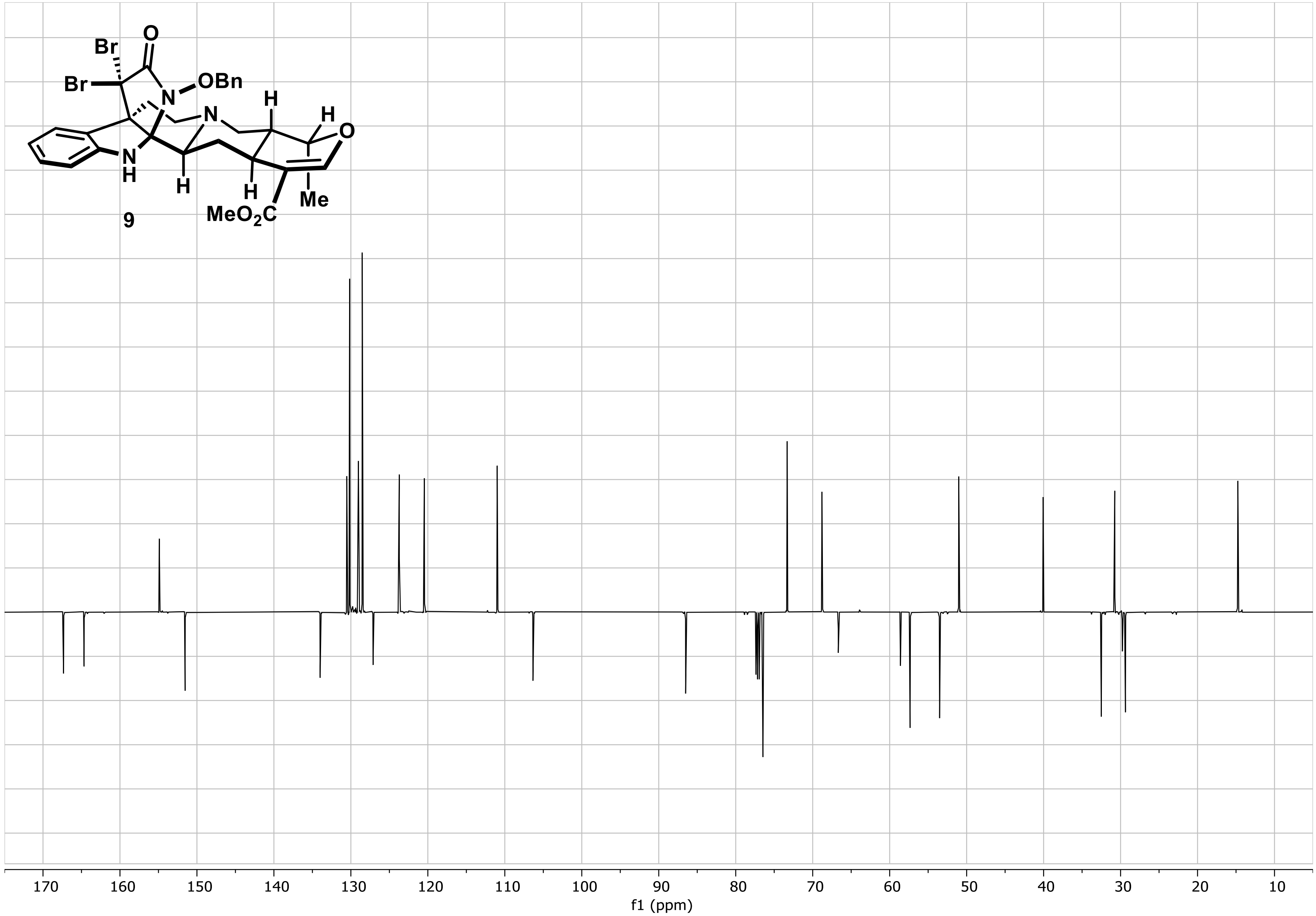

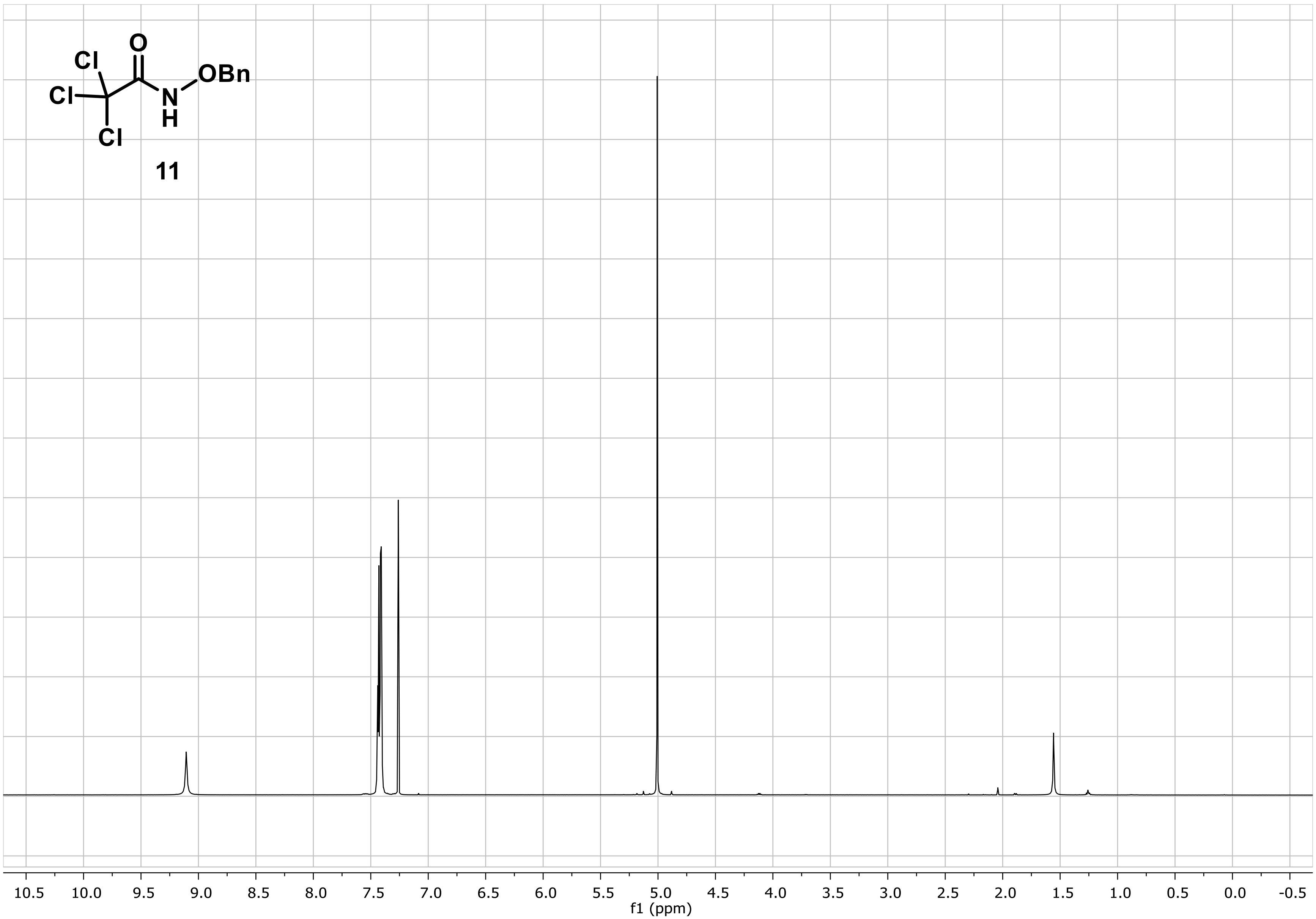

Cl
Cl
Cl
O
OBn
N
H
11
10.5
10.0
9.5
9.0
8.5
8.0
7.5
7.0
6.5
6.0
5.5
5.0
4.5
4.0
3.5
3.0
2.5
2.0
1.5
1.0
0.5
0.0
-0.5
f1 (ppm)

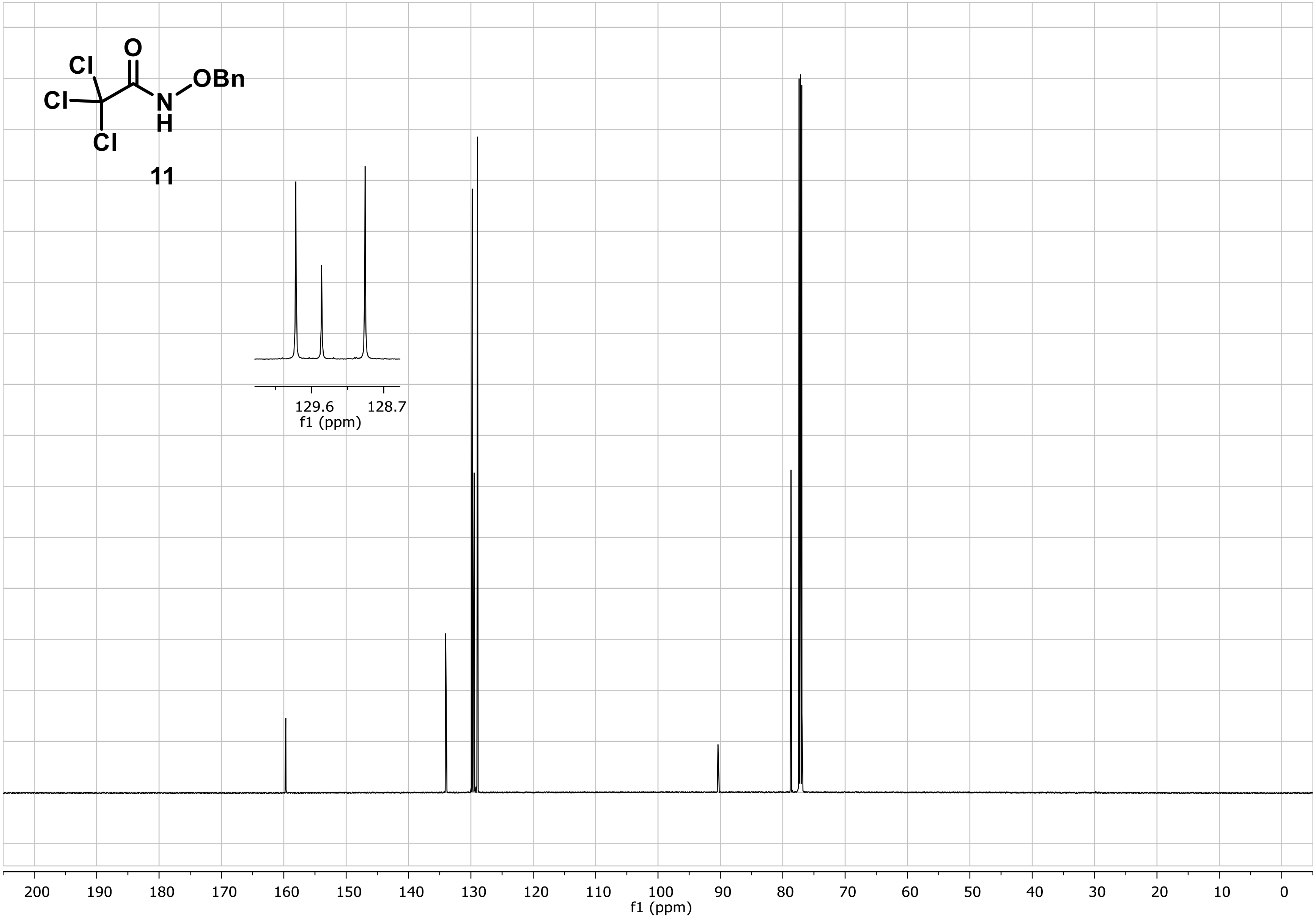

129.6
128.7
f1 (ppm)
200
190
180
170
160
150
140
130
120
110
100
90
80
70
60
50
40
30
20
10
0
f1 (ppm)

11

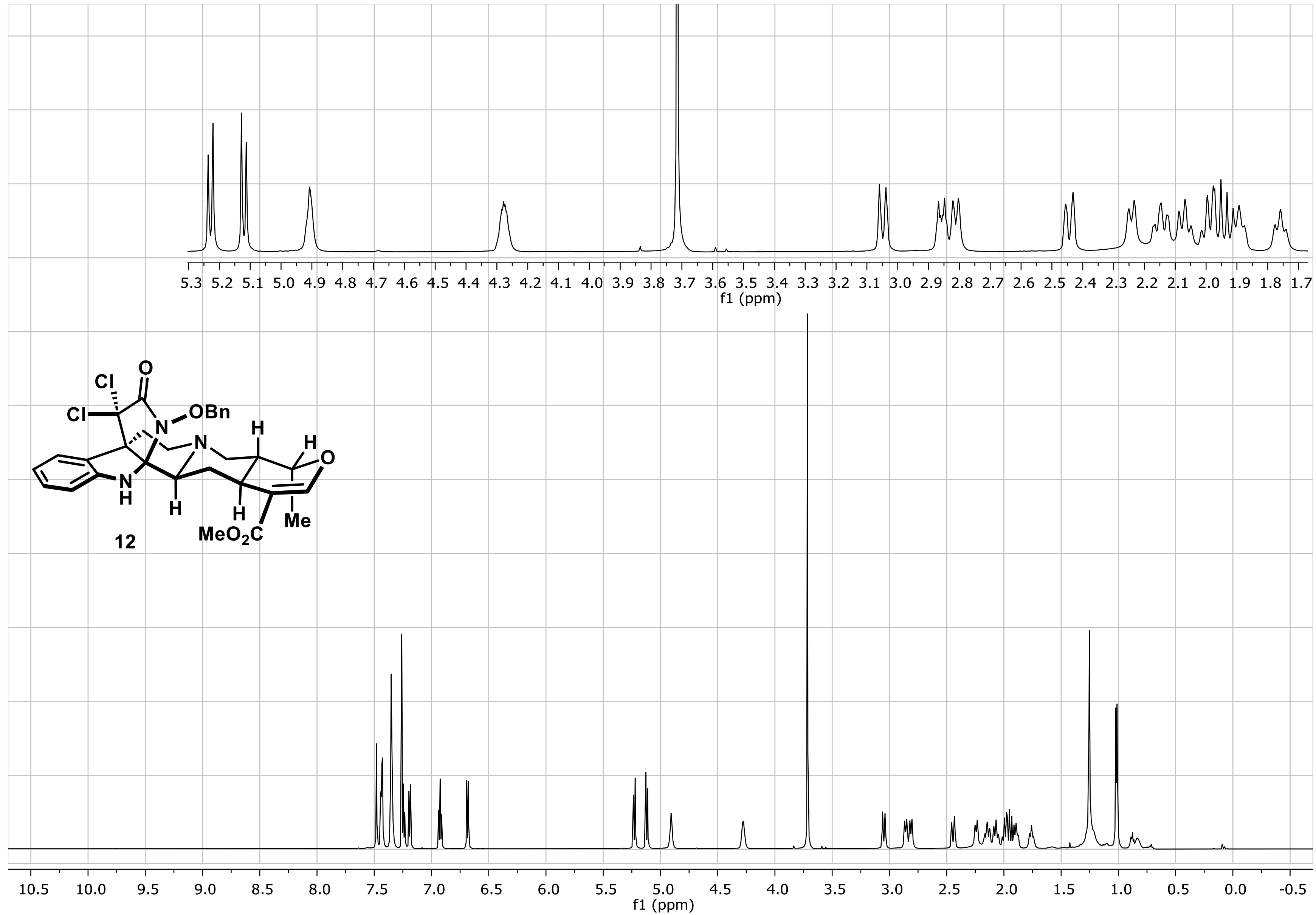
f1 (ppm)
f1 (ppm)
12

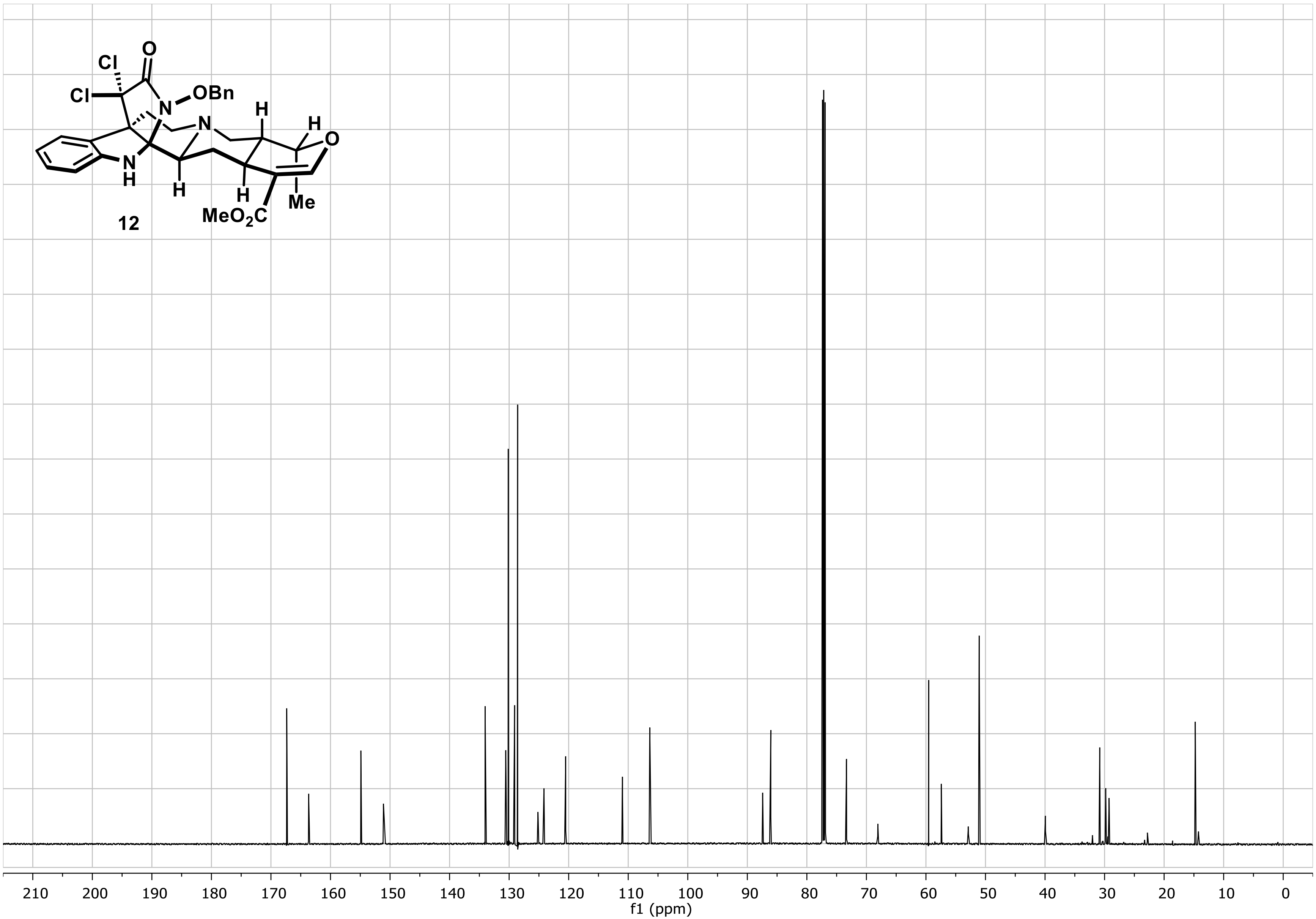
Cl
Cl
O
N
OBn
N
H
H
O
NH
H
H
H
MeO2C
Me
12
210 200 190 180 170 160 150 140 130 120 110 100 90 80 70 60 50 40 30 20 10 0
f1 (ppm)

NOTE: *In JMOD quaternary (-C-) and methylene (-CH_2-) signals have opposite phase to those of methine (-CH-) and methyl (-CH_3) resonances.*

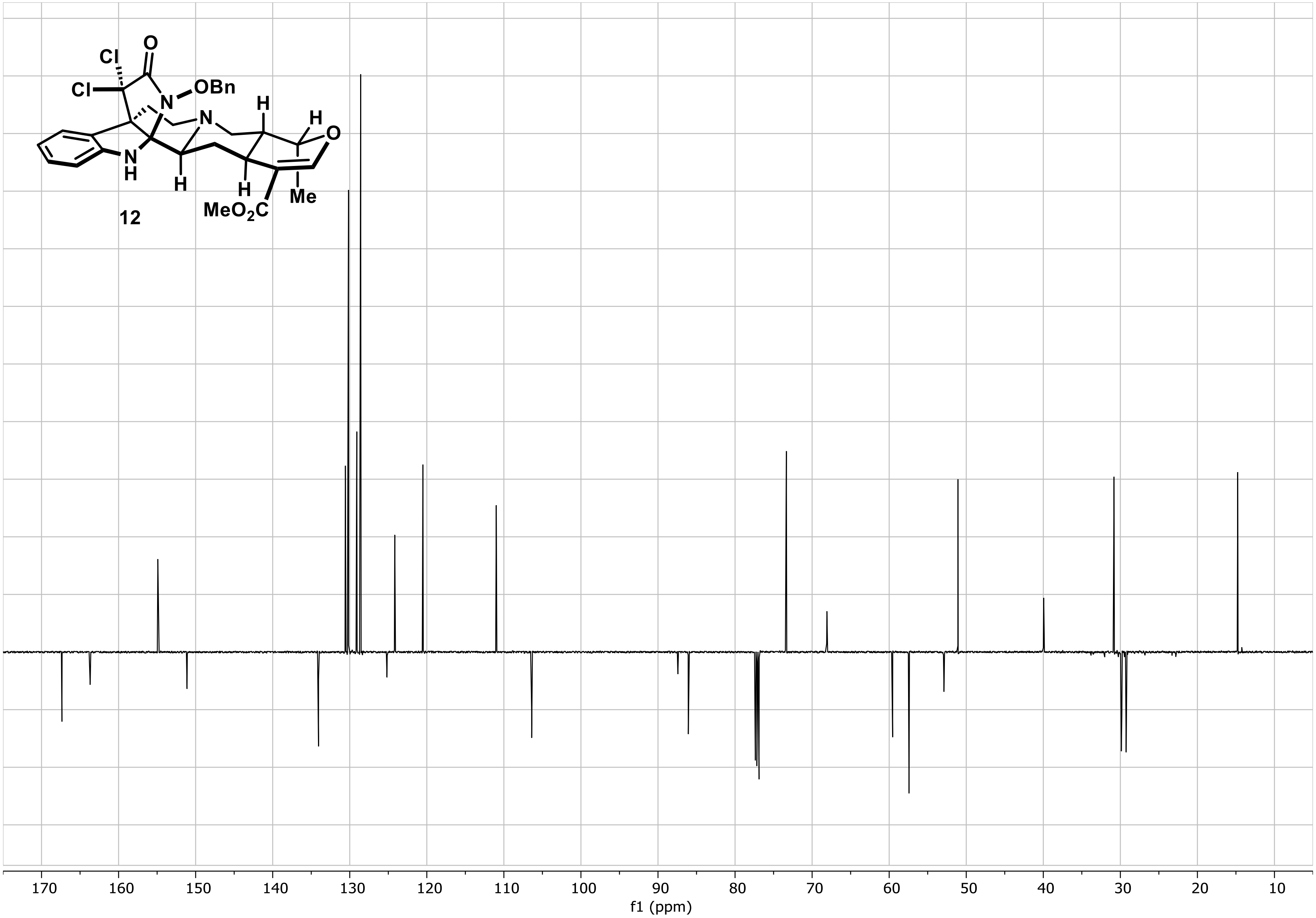

JMOD of 12

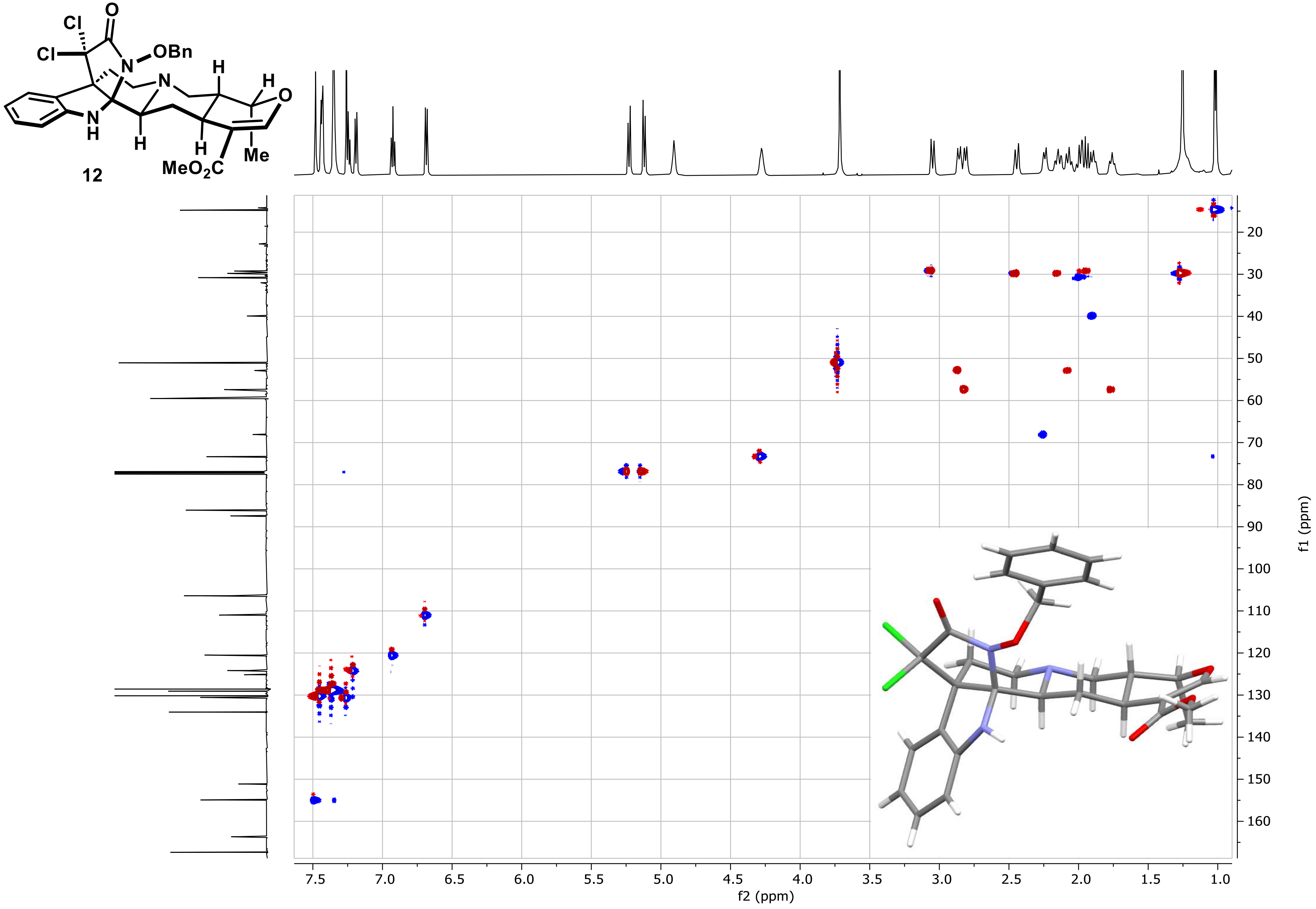

HSQC of 12 (with X-ray)

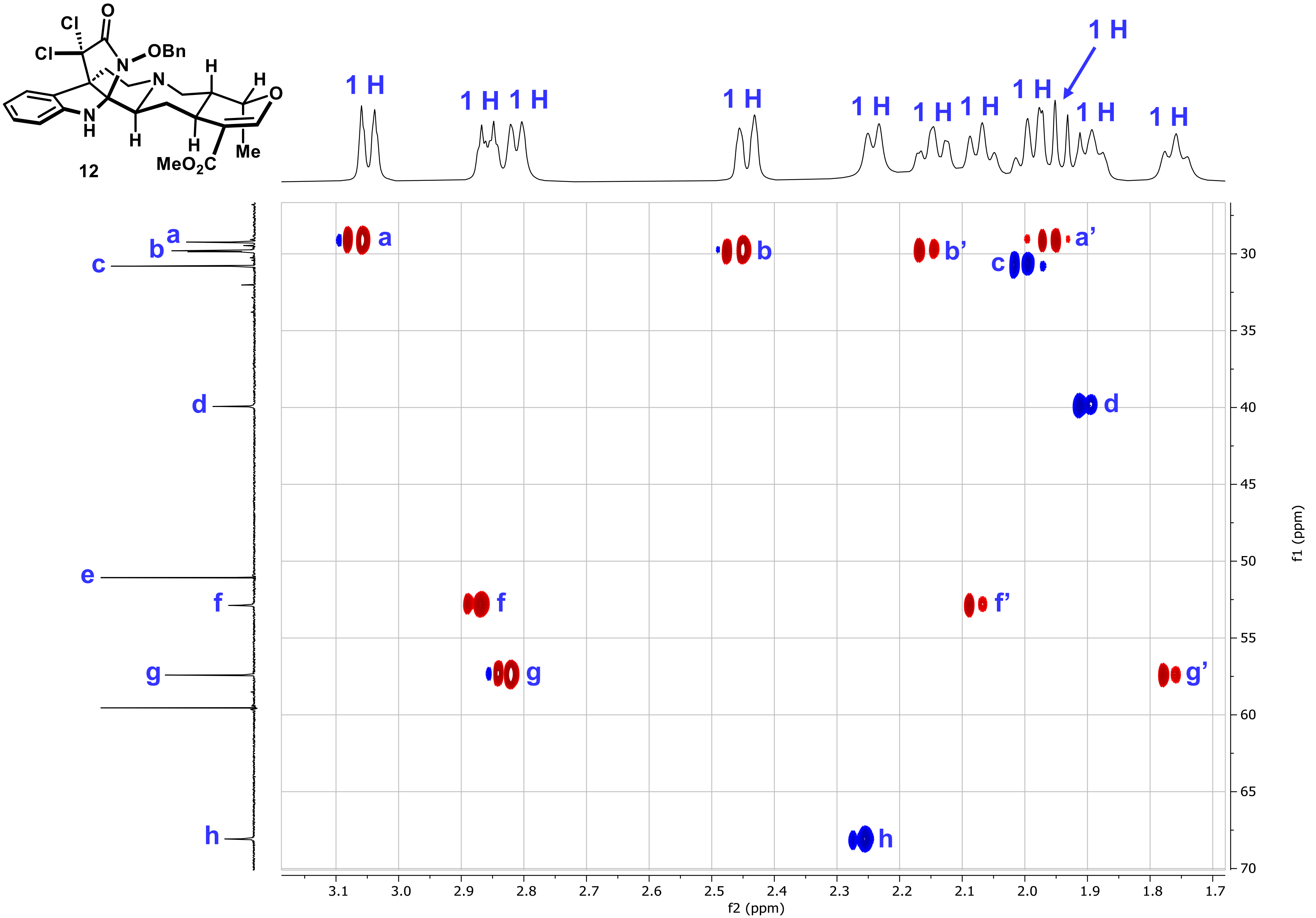

Zoomed In Version: HSQC of 12

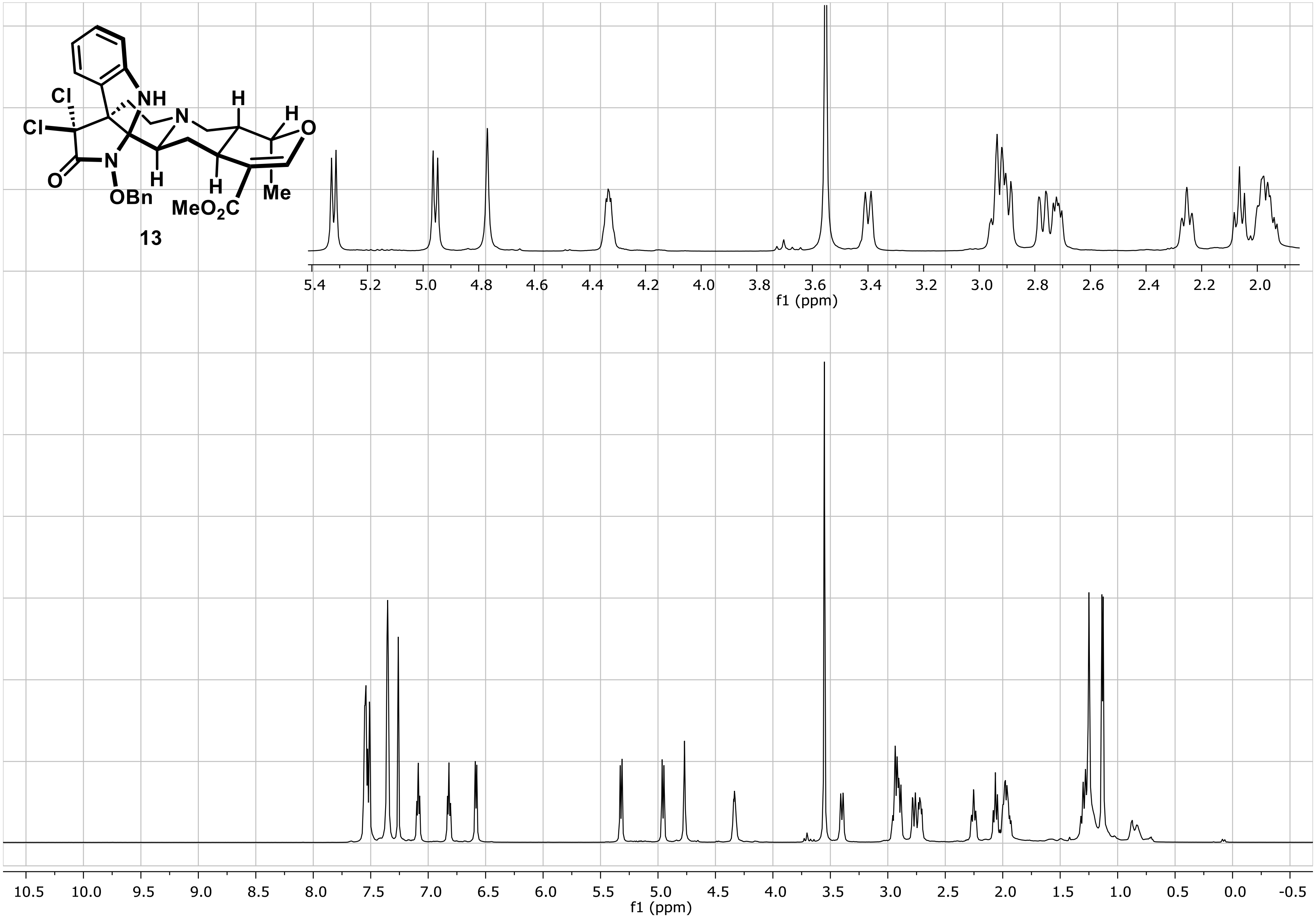
Cl
Cl
NH
N
H
H
O
N
O
H
H
OBn
MeO2C
Me
13
5.4
5.2
5.0
4.8
4.6
4.4
4.2
4.0
3.8
3.6
3.4
3.2
3.0
2.8
2.6
2.4
2.2
2.0
f1 (ppm)
10.5
10.0
9.5
9.0
8.5
8.0
7.5
7.0
6.5
6.0
5.5
5.0
4.5
4.0
3.5
3.0
2.5
2.0
1.5
1.0
0.5
0.0
-0.5
f1 (ppm)

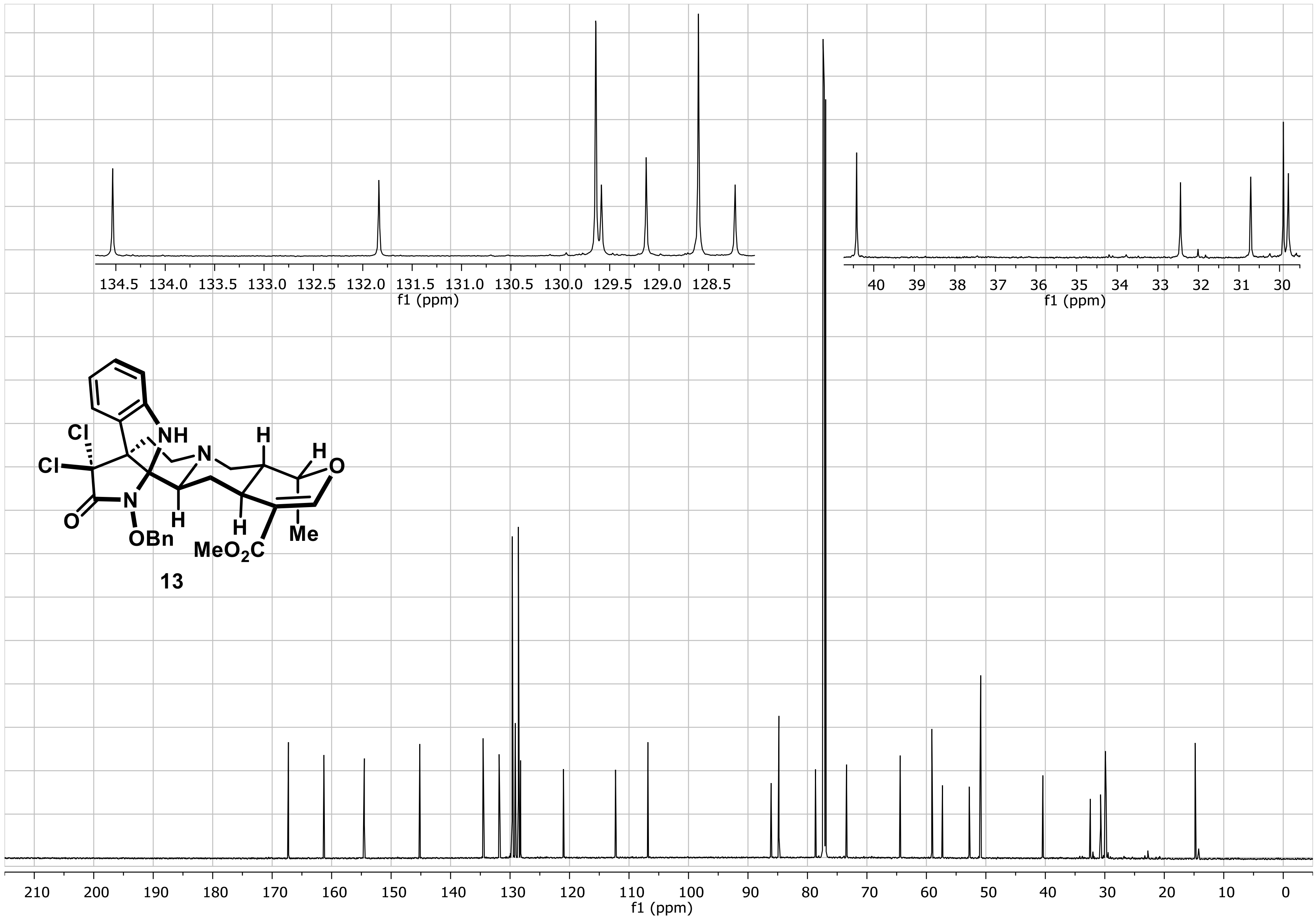

134.5 134.0 133.5 133.0 132.5 132.0 131.5 131.0 130.5 130.0 129.5 129.0 128.5
f1 (ppm)
40 39 38 37 36 35 34 33 32 31 30
f1 (ppm)
Cl
Cl
NH
N
H
H
O
O
N
OBn
H
H
MeO2C
Me
13
210 200 190 180 170 160 150 140 130 120 110 100 90 80 70 60 50 40 30 20 10 0
f1 (ppm)

NOTE: *In JMOD quaternary (-C-) and methylene (-CH_2-) signals have opposite phase to those of methine (-CH-) and methyl (-CH_3) resonances.*

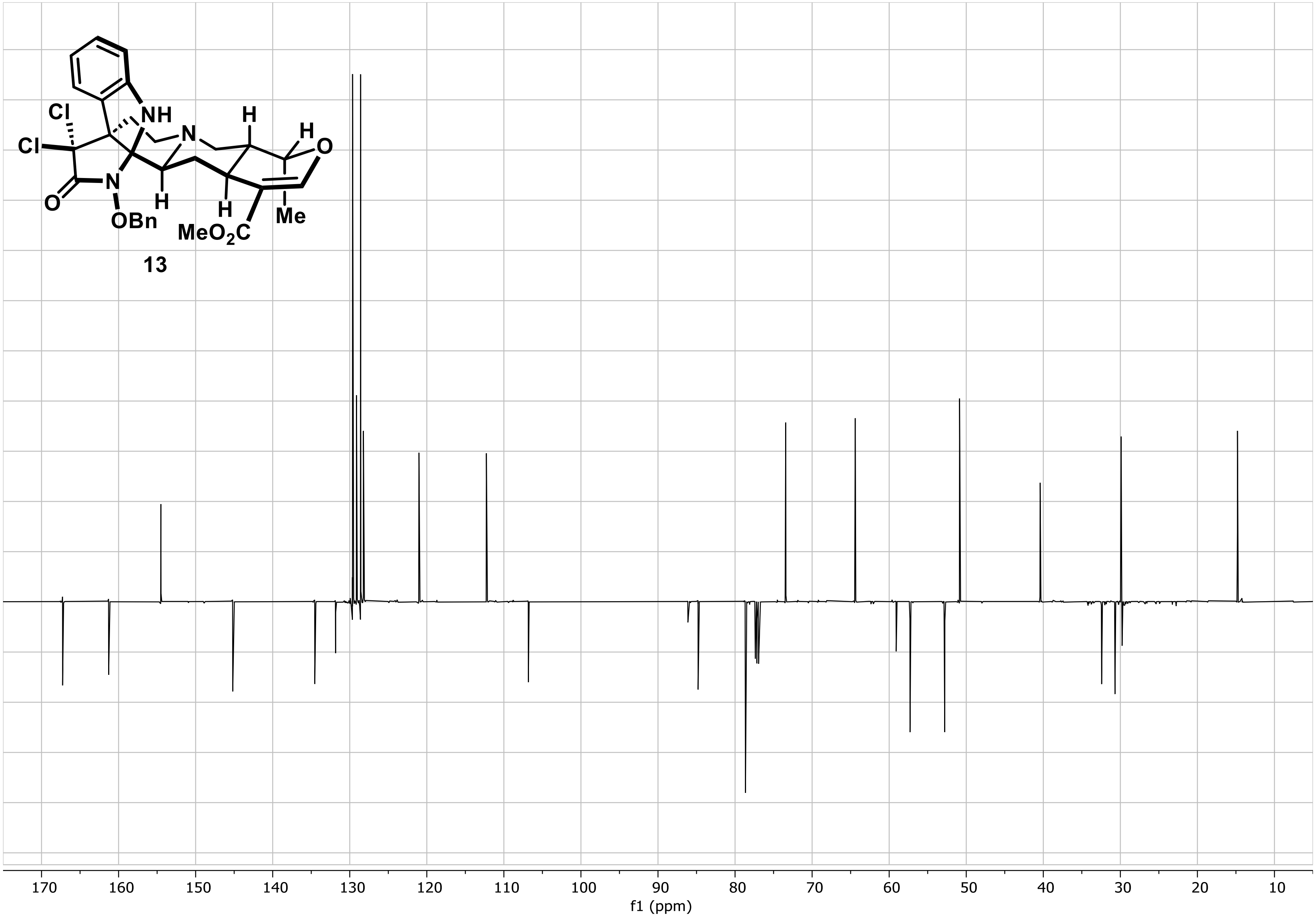

JMOD of 13

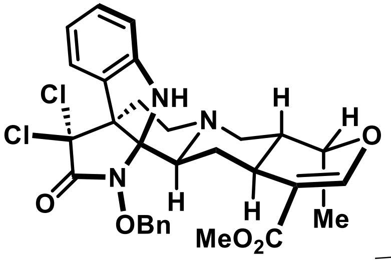

13

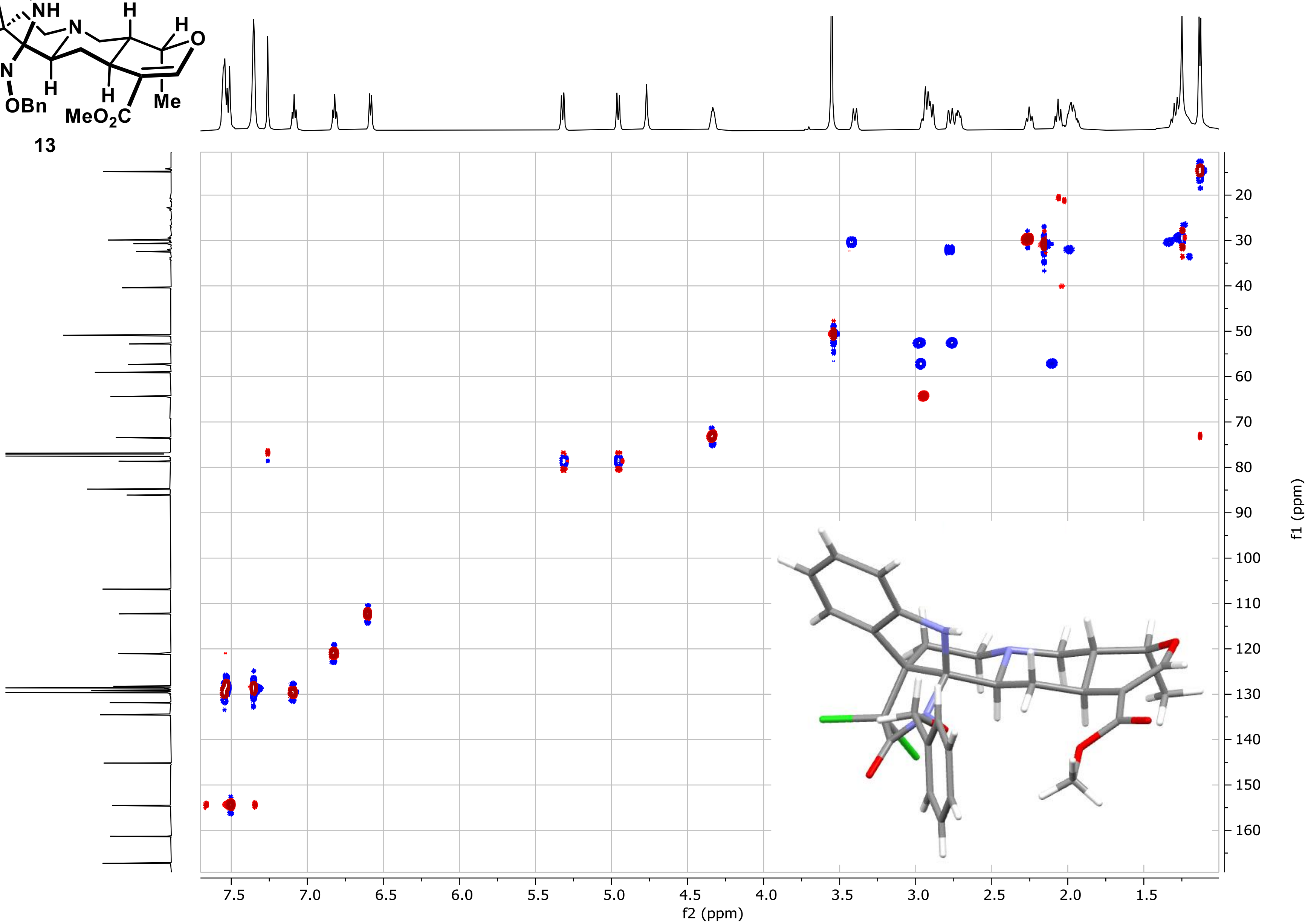

HSQC of 13 (with X-ray)

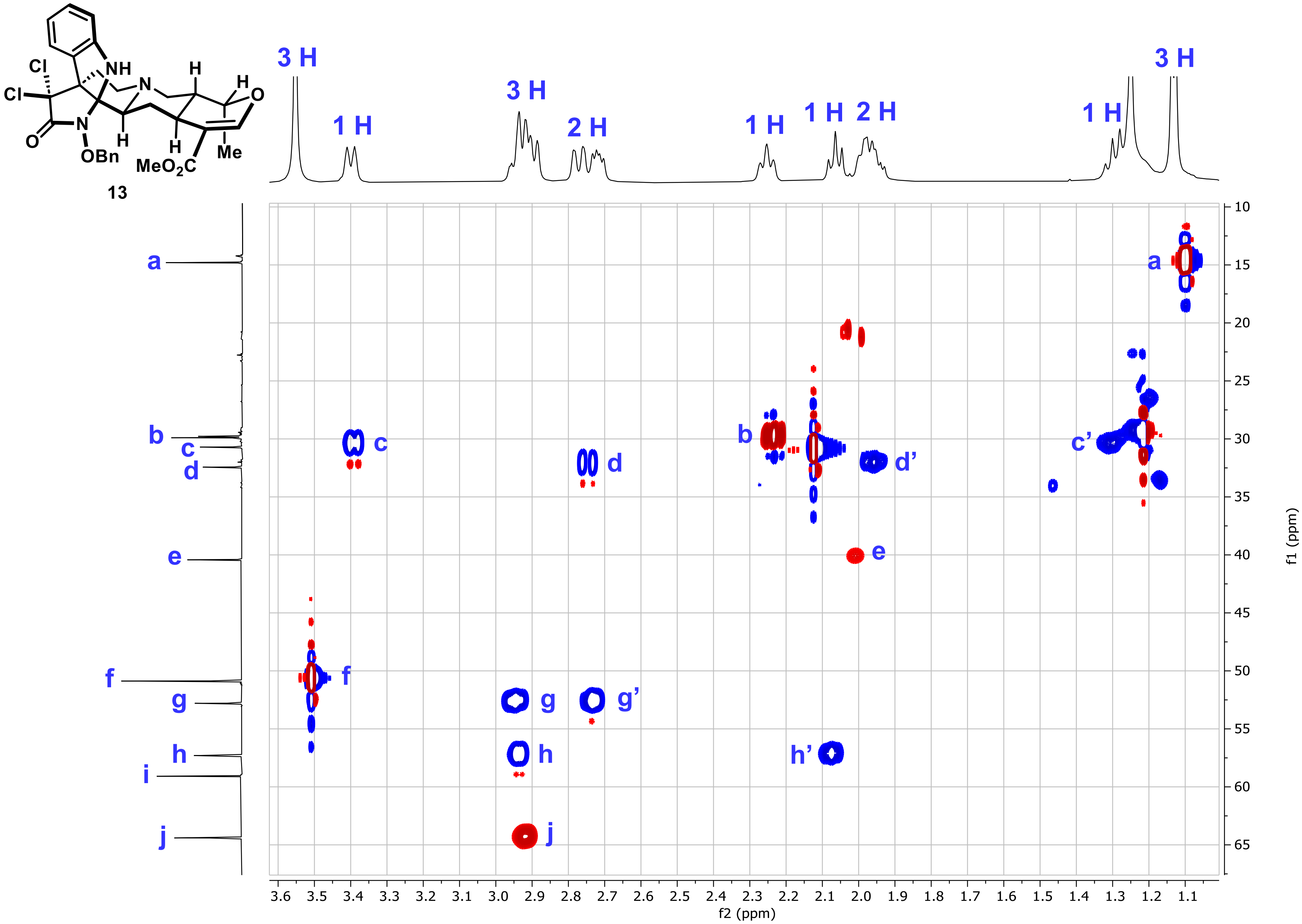

13

Zoomed In Version: HSQC of 13

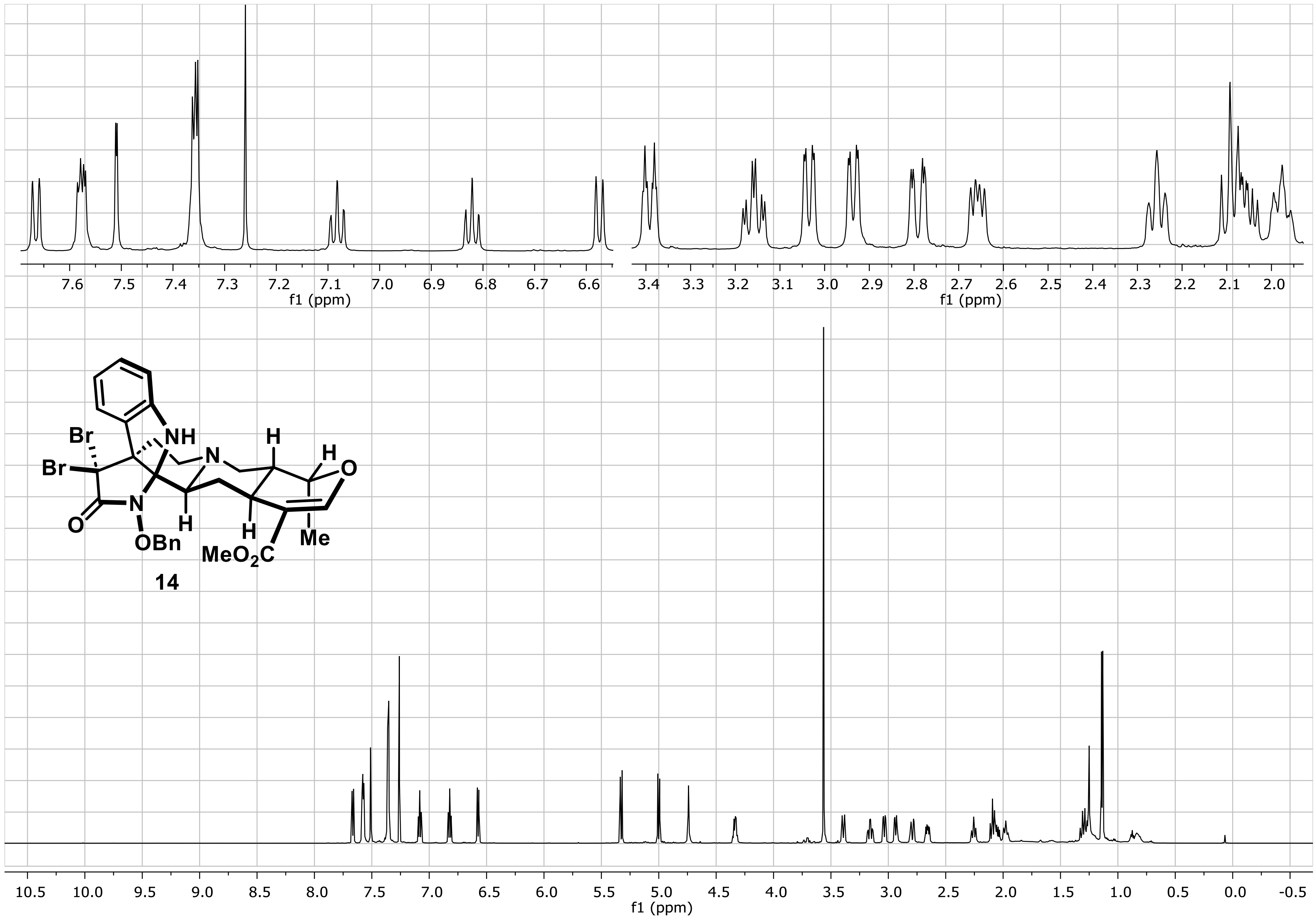
f1 (ppm)
f1 (ppm)
Br
Br
NH
N
H
H
O
O
N
OBn
H
H
MeO2C
Me
14
f1 (ppm)

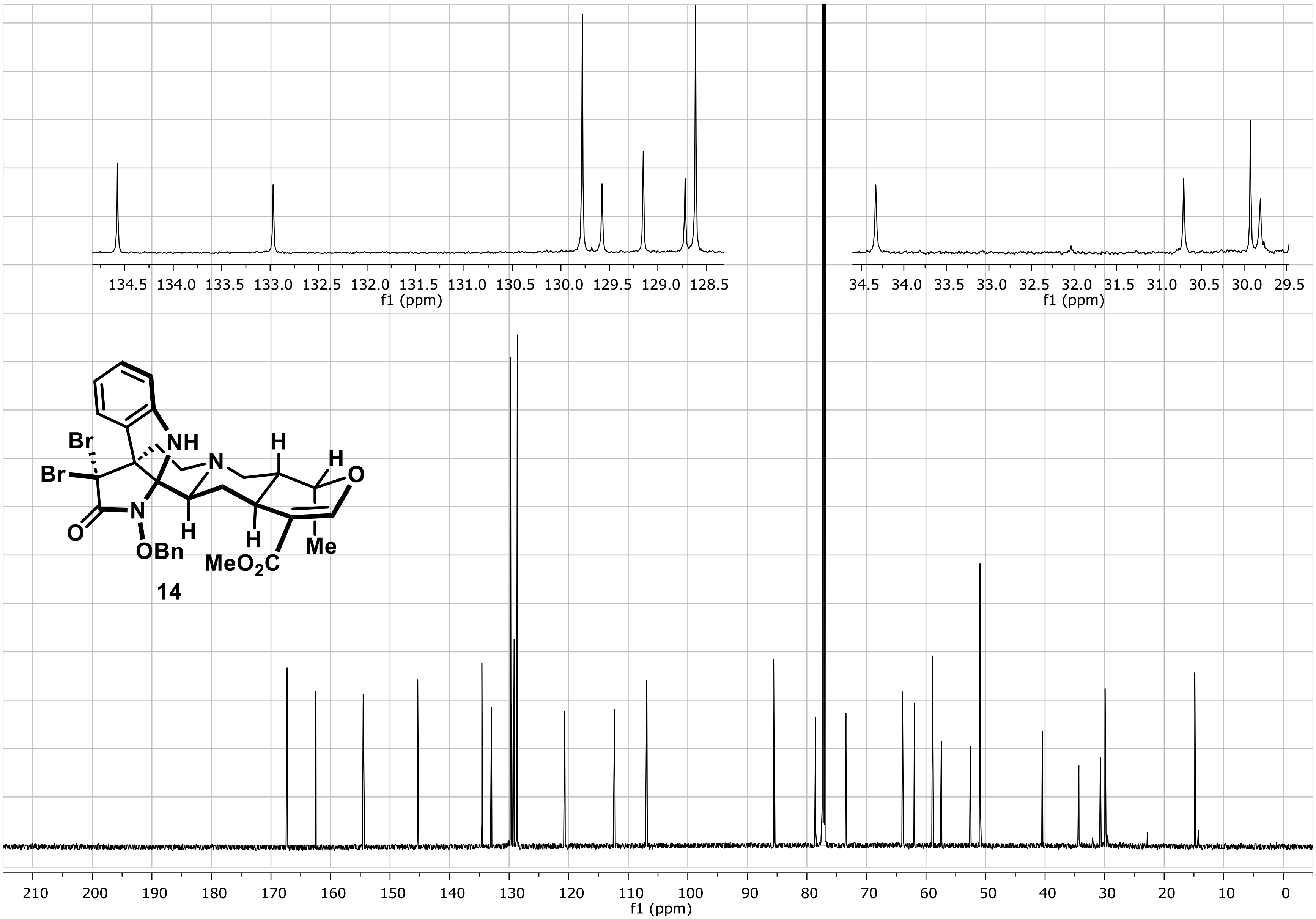
134.5 134.0 133.5 133.0 132.5 132.0 131.5 131.0 130.5 130.0 129.5 129.0 128.5
f1 (ppm)
34.5 34.0 33.5 33.0 32.5 32.0 31.5 31.0 30.5 30.0 29.5
f1 (ppm)
Br
Br
NH
N
H
H
O
O
N
OBn
H
H
MeO2C
Me
14
210 200 190 180 170 160 150 140 130 120 110 100 90 80 70 60 50 40 30 20 10 0
f1 (ppm)

NOTE: *In JMOD quaternary (-C-) and methylene (-CH$_2$-) signals have opposite phase to those of methine (-CH-) and methyl (-CH$_3$) resonances.*

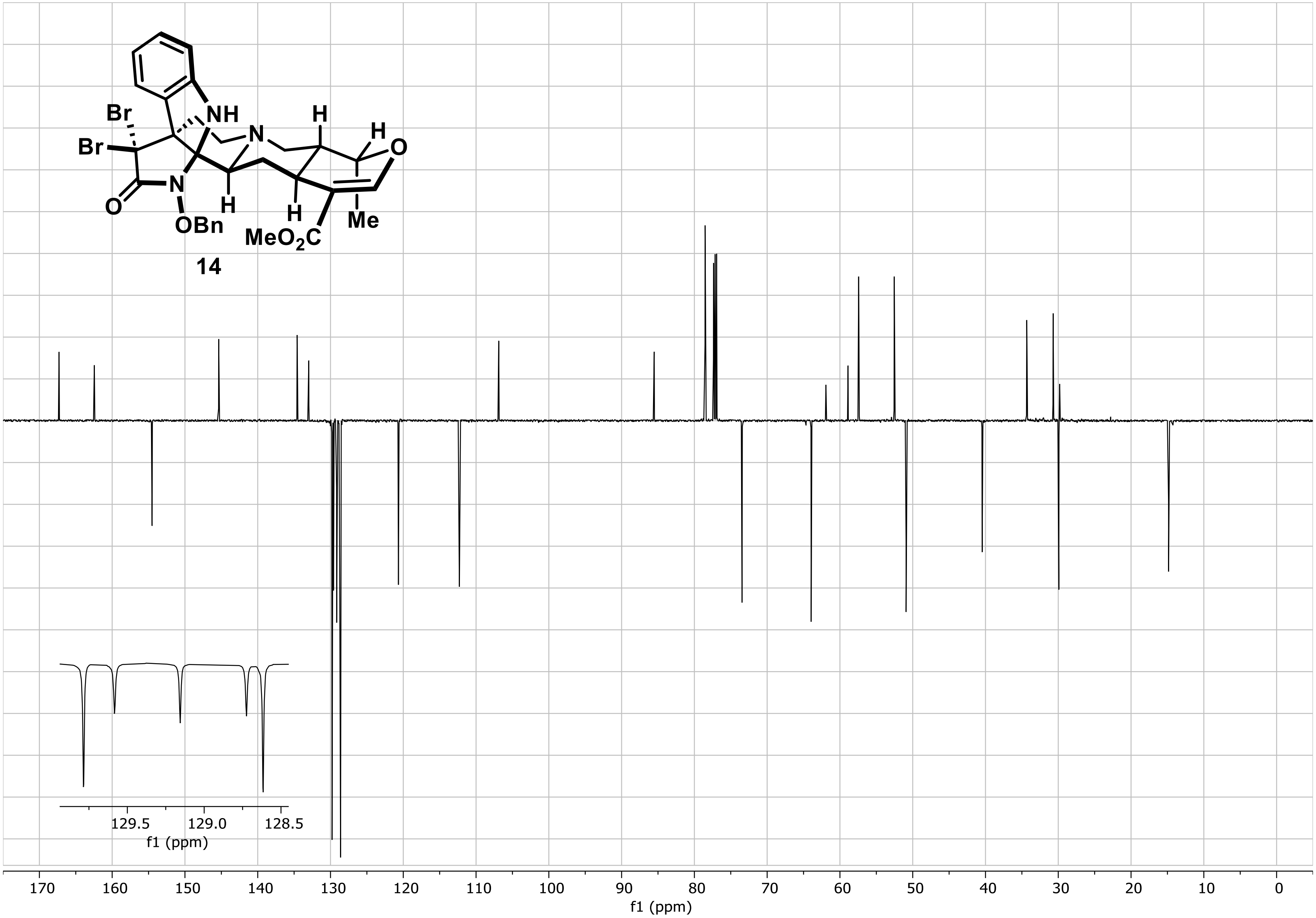

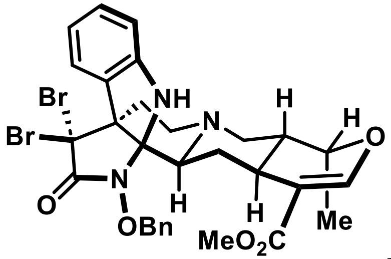

14

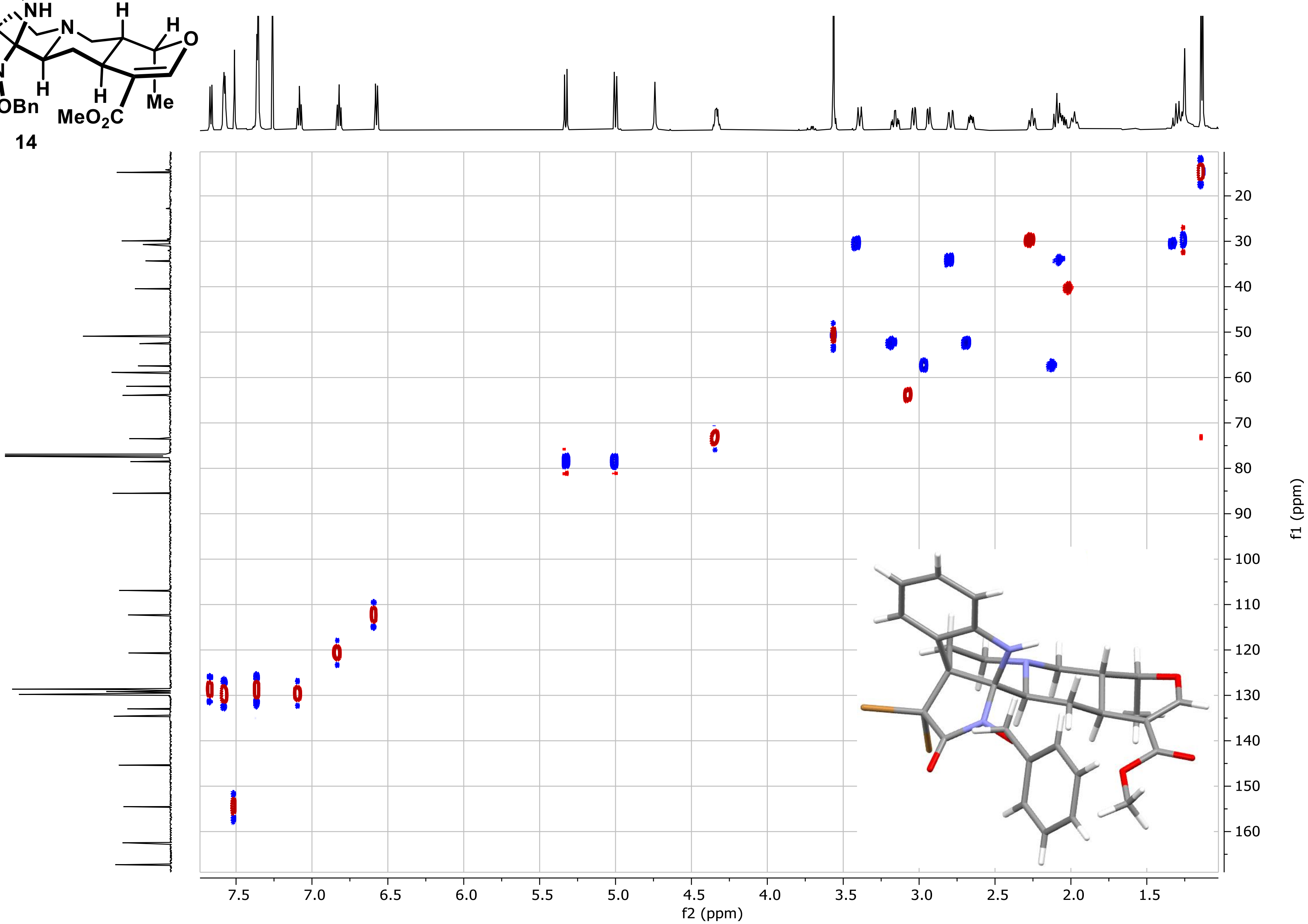

HSQC of 14 (with X-ray)

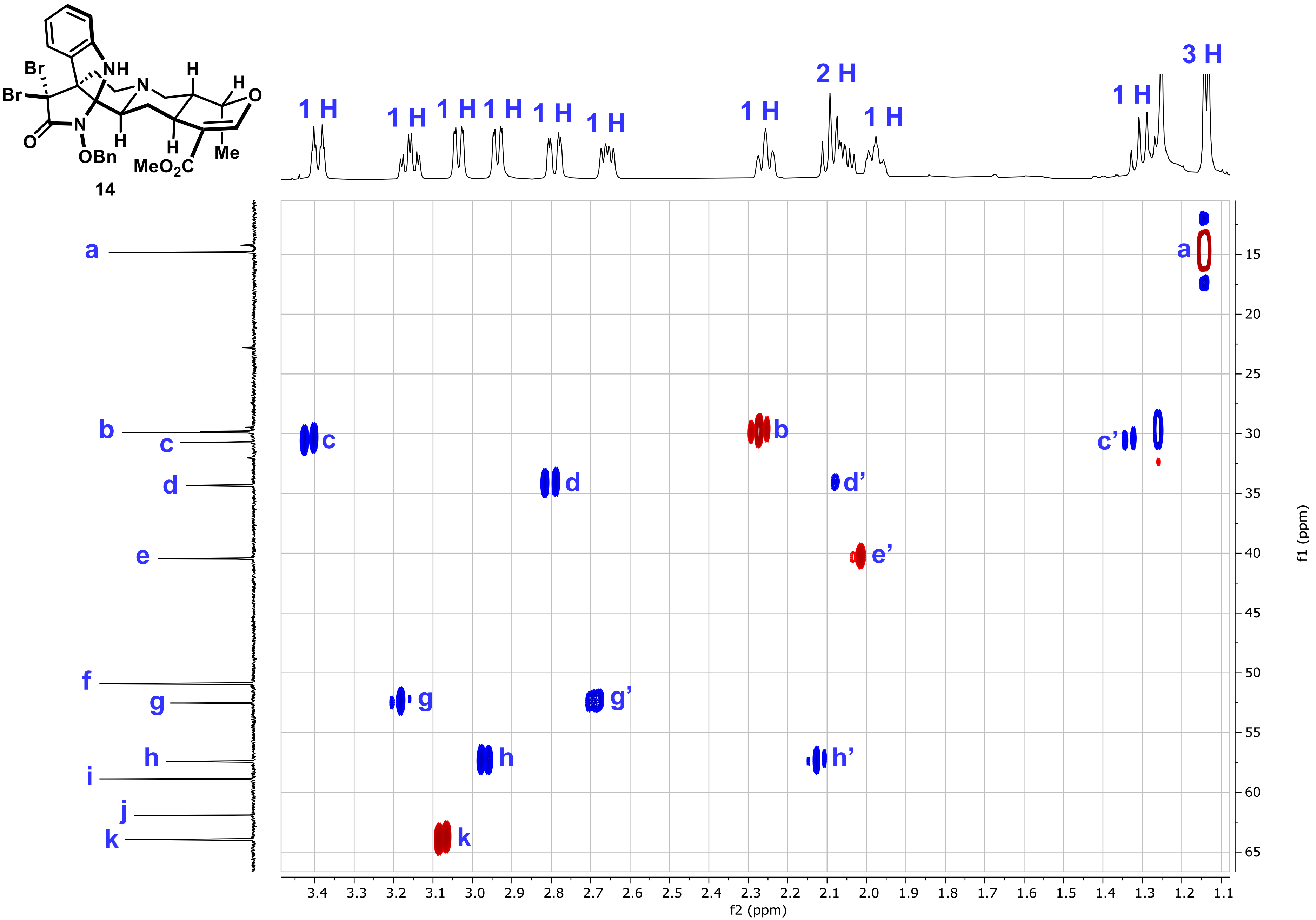

Zoomed In Version: HSQC of 14

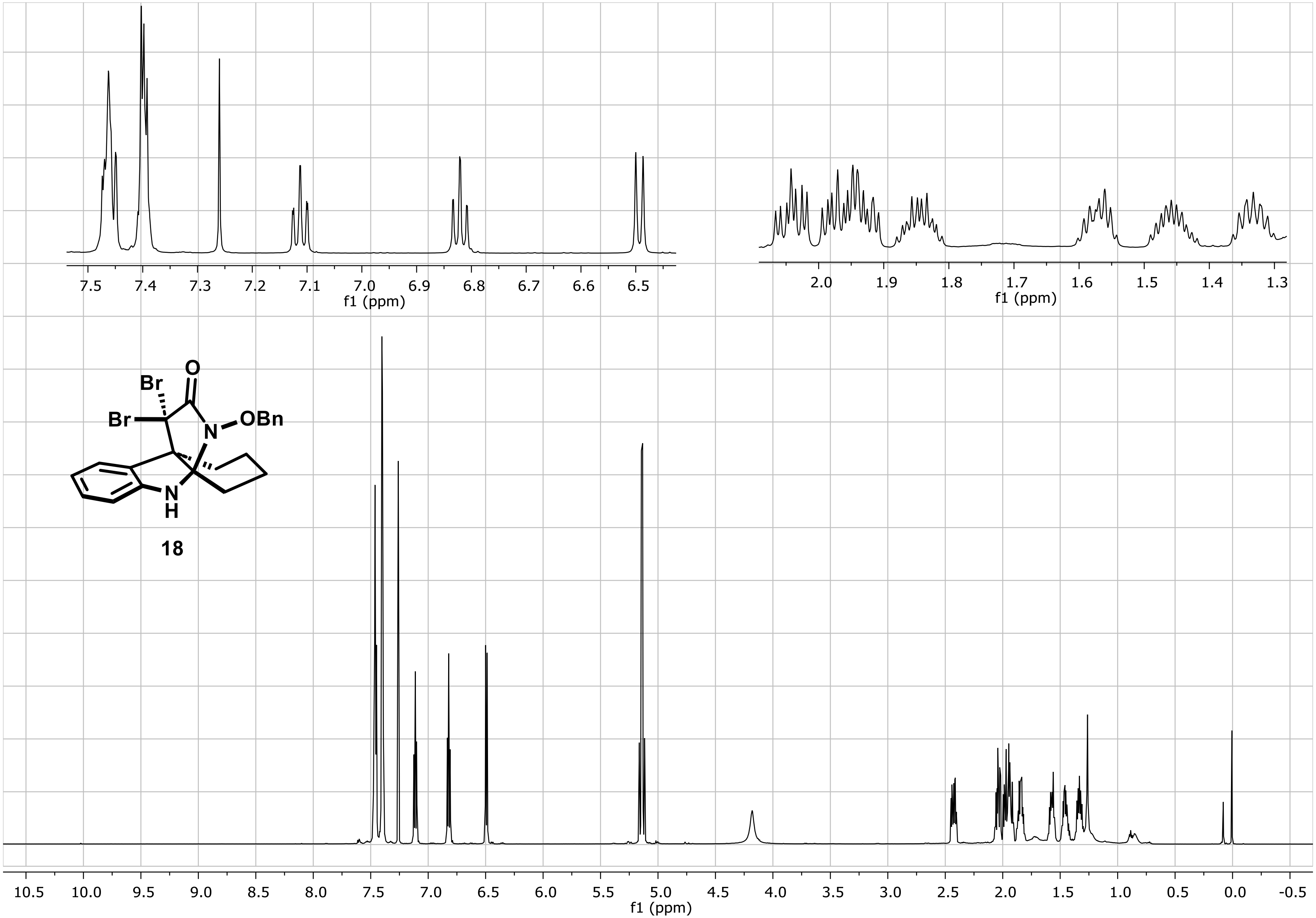
Br
Br
O
N
OBn
N
H
18
7.5 7.4 7.3 7.2 7.1 7.0 6.9 6.8 6.7 6.6 6.5
f1 (ppm)
2.0 1.9 1.8 1.7 1.6 1.5 1.4 1.3
f1 (ppm)
10.5 10.0 9.5 9.0 8.5 8.0 7.5 7.0 6.5 6.0 5.5 5.0 4.5 4.0 3.5 3.0 2.5 2.0 1.5 1.0 0.5 0.0 -0.5
f1 (ppm)

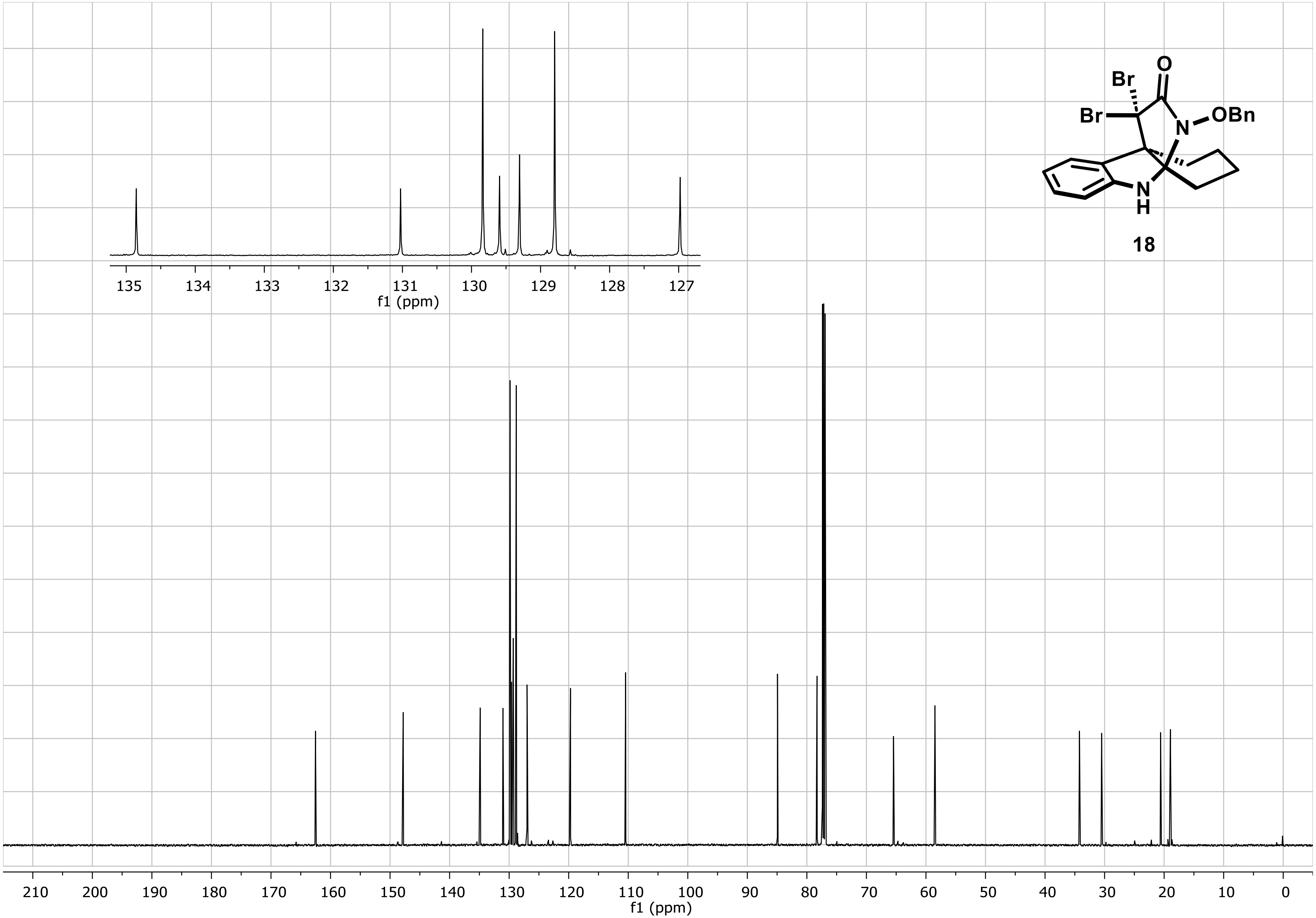

Br
Br
O
N
OBn
N
H
135 134 133 132 131 130 129 128 127
f1 (ppm)
210 200 190 180 170 160 150 140 130 120 110 100 90 80 70 60 50 40 30 20 10 0
f1 (ppm)

18

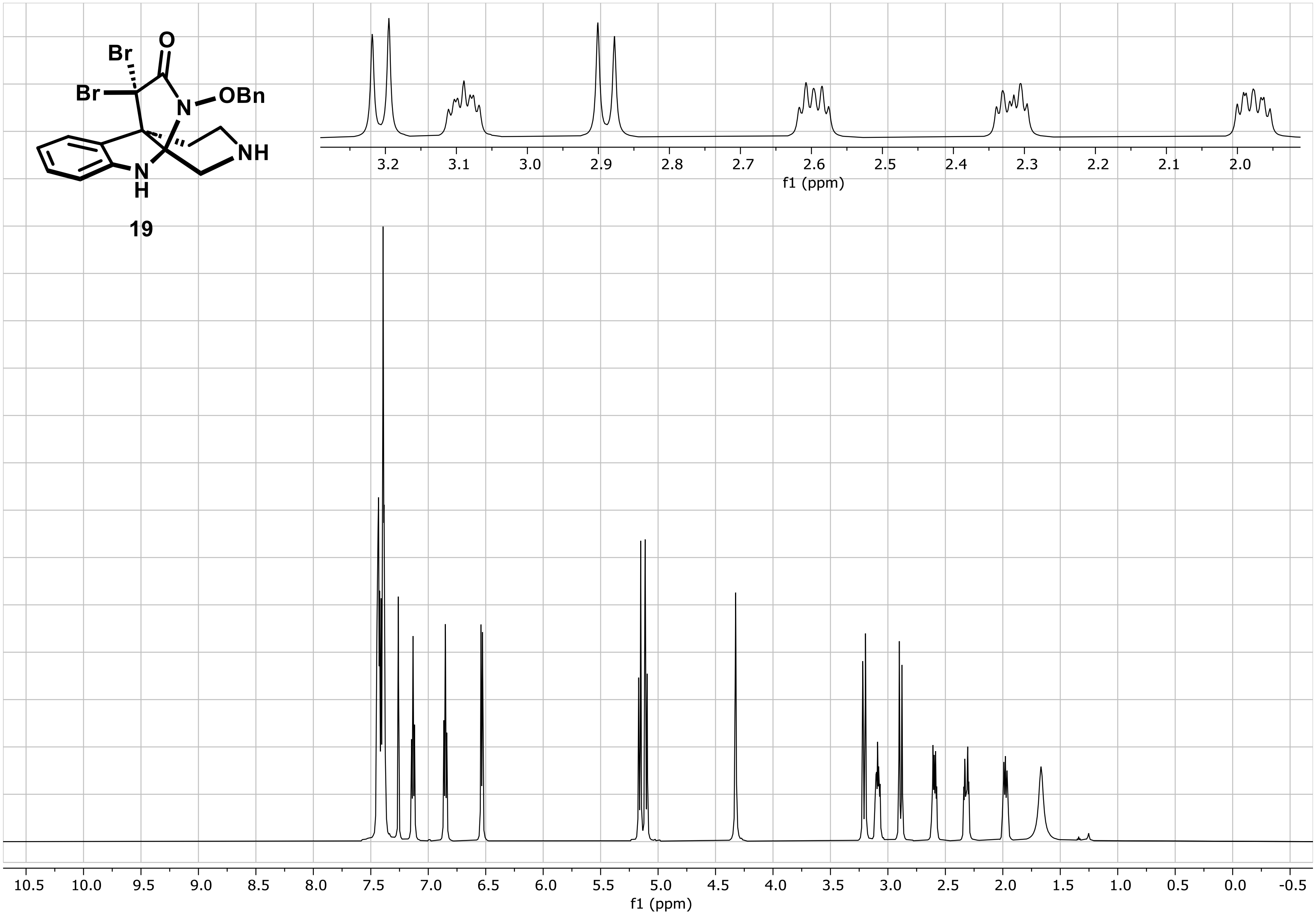
Br
Br
O
N
OBn
NH
N
H
19
3.2
3.1
3.0
2.9
2.8
2.7
2.6
2.5
2.4
2.3
2.2
2.1
2.0
f1 (ppm)
10.5
10.0
9.5
9.0
8.5
8.0
7.5
7.0
6.5
6.0
5.5
5.0
4.5
4.0
3.5
3.0
2.5
2.0
1.5
1.0
0.5
0.0
-0.5
f1 (ppm)

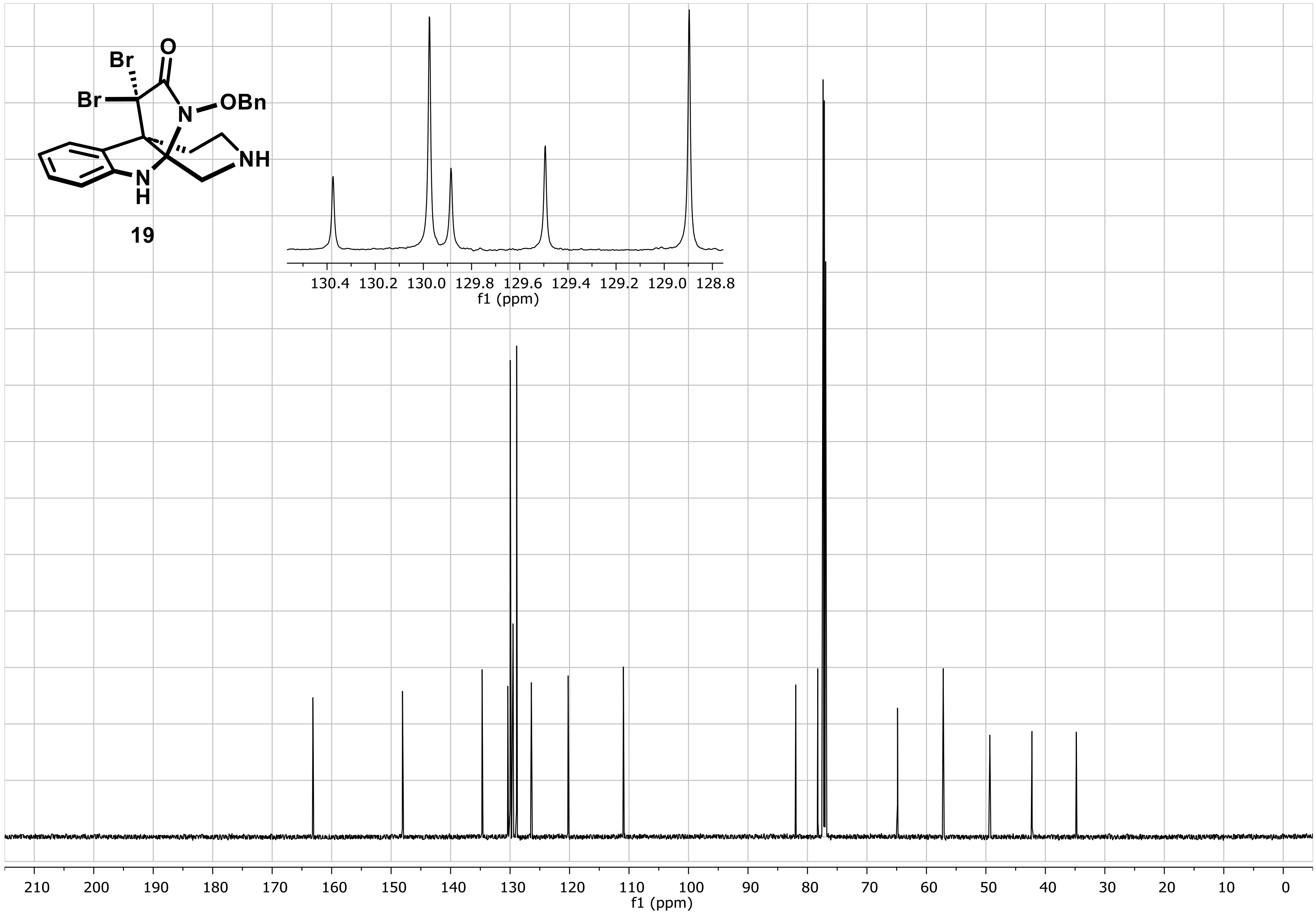
Br
O
Br
N
OBn
NH
N
H
19
130.4 130.2 130.0 129.8 129.6 129.4 129.2 129.0 128.8
f1 (ppm)
210 200 190 180 170 160 150 140 130 120 110 100 90 80 70 60 50 40 30 20 10 0
f1 (ppm)

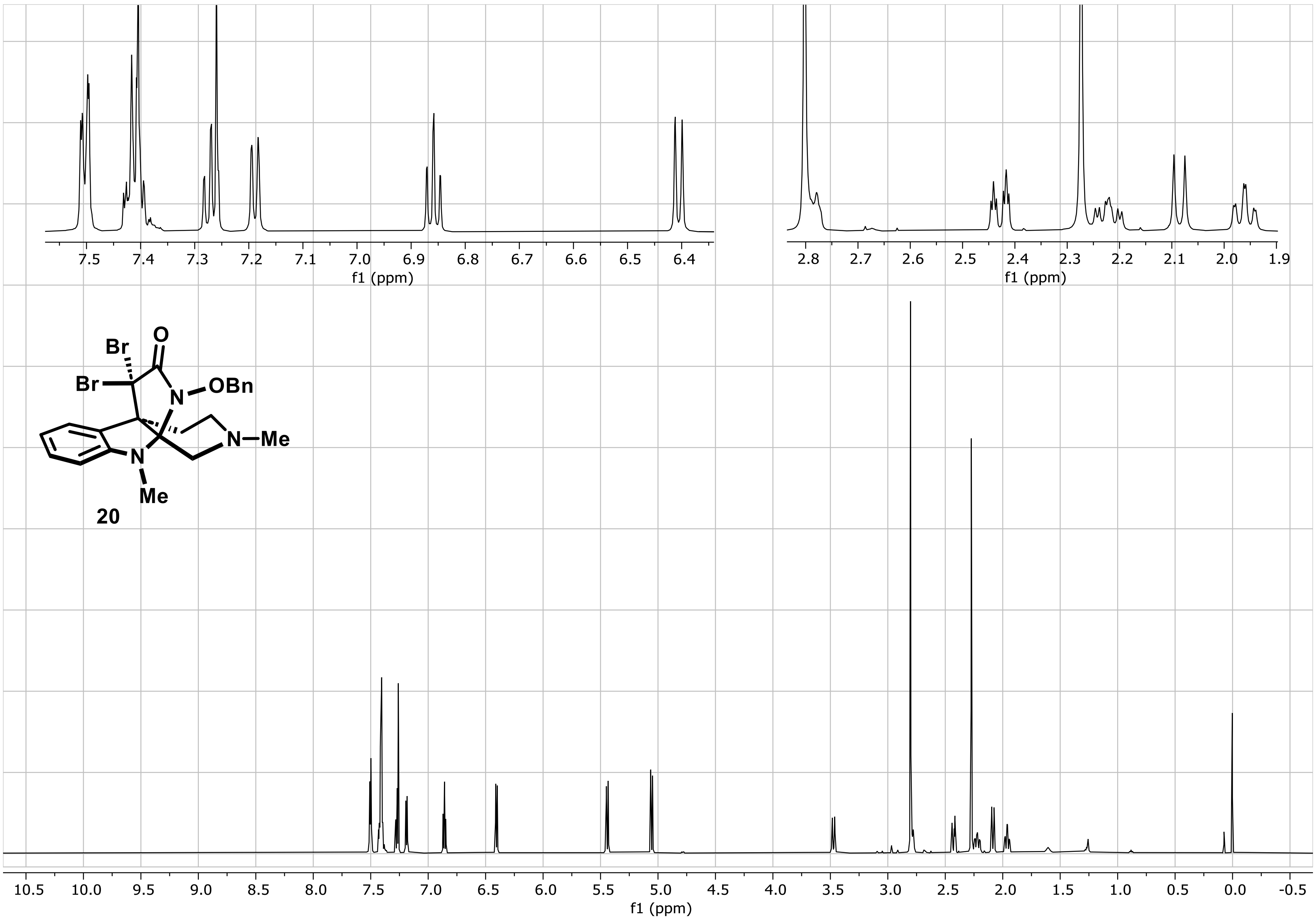
Br
O
Br
N
OBn
N
Me
N
Me
20
f1 (ppm)
f1 (ppm)
f1 (ppm)

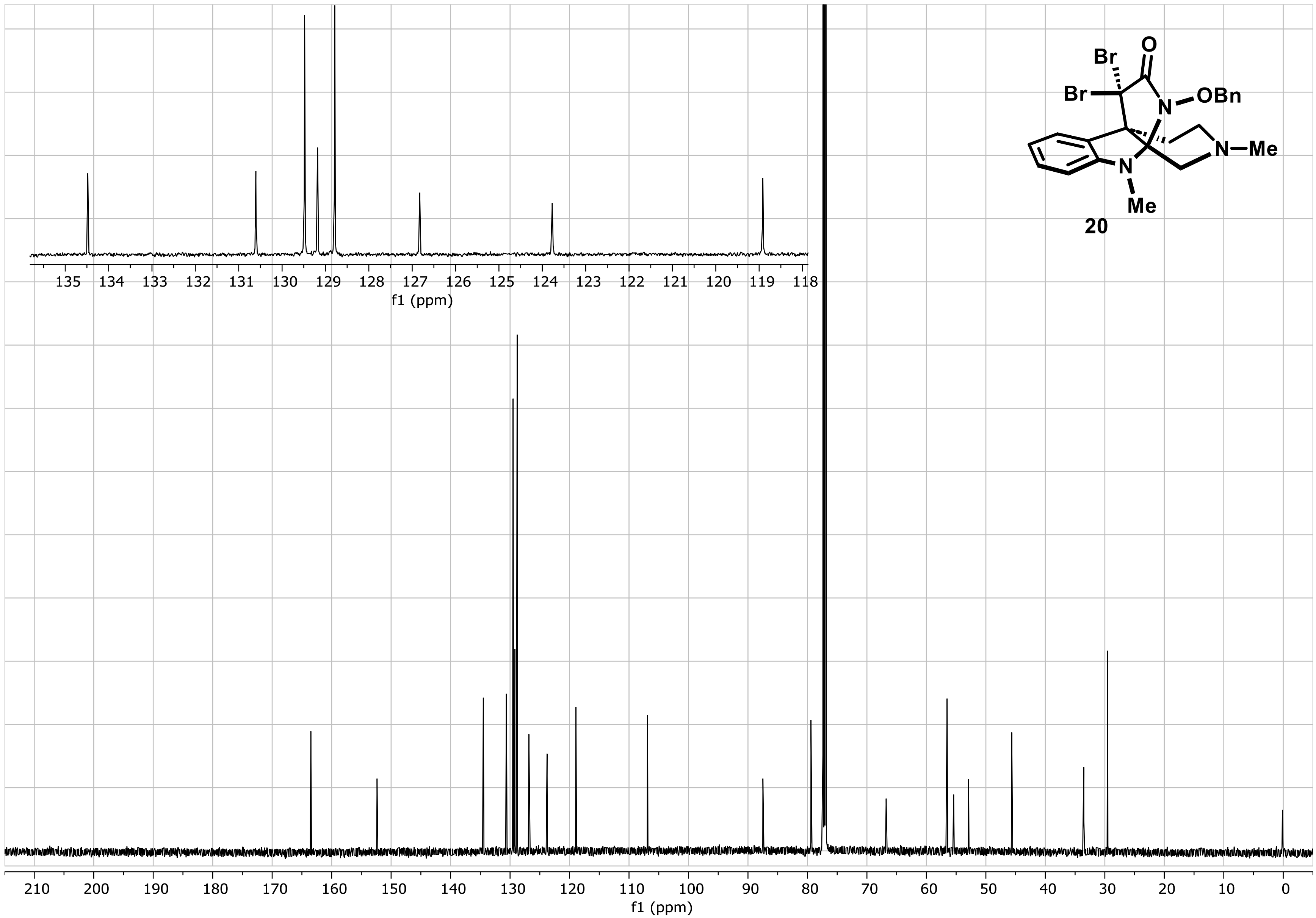
f1 (ppm)
f1 (ppm)
Br
Br
O
N
OBn
N
Me
N
Me
20

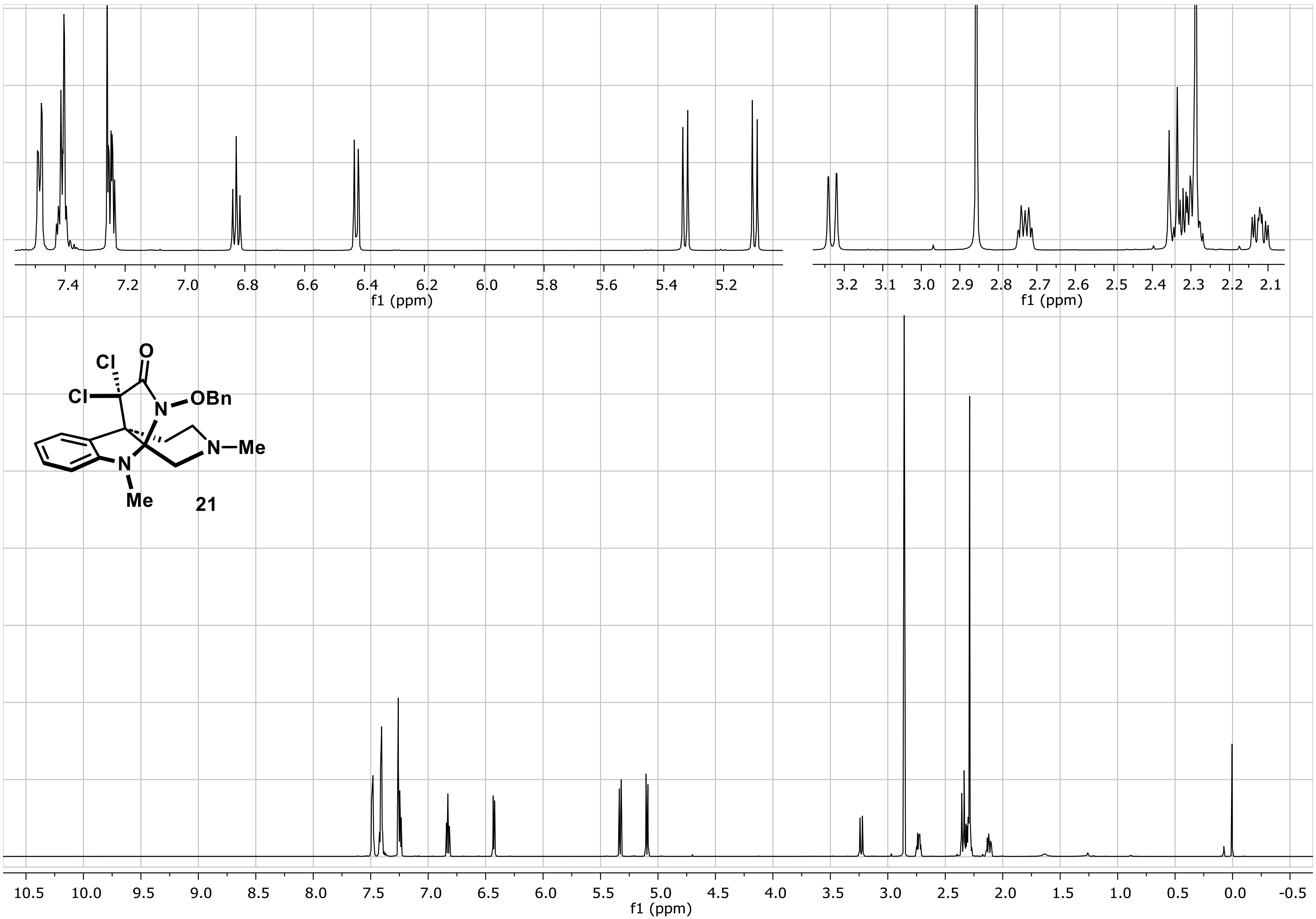
Cl
Cl
O
N
OBn
N
Me
N
Me
21
f1 (ppm)
f1 (ppm)
f1 (ppm)

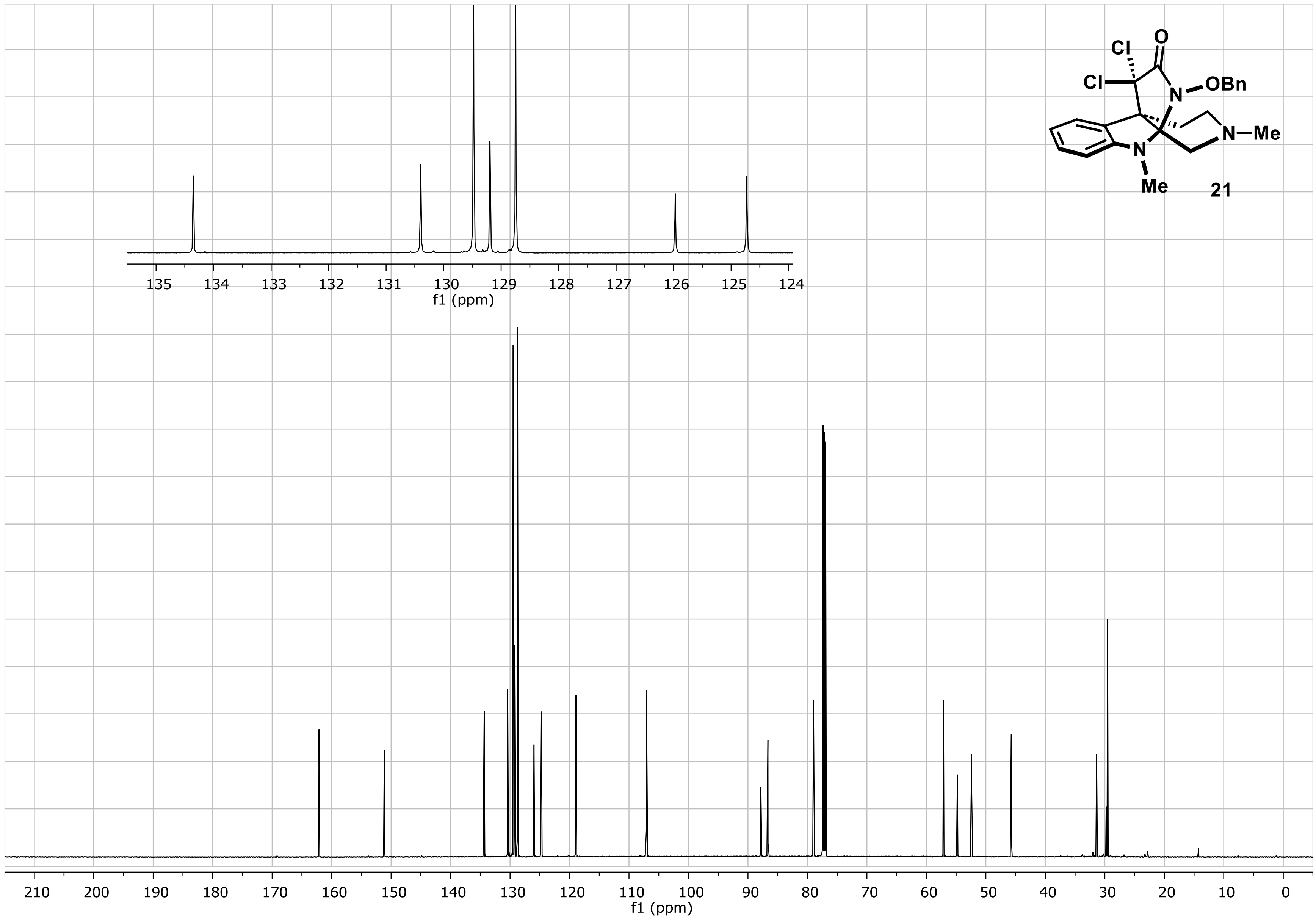
Cl
Cl
O
N
OBn
N
Me
N
Me
21
135 134 133 132 131 130 129 128 127 126 125 124
f1 (ppm)
210 200 190 180 170 160 150 140 130 120 110 100 90 80 70 60 50 40 30 20 10 0
f1 (ppm)

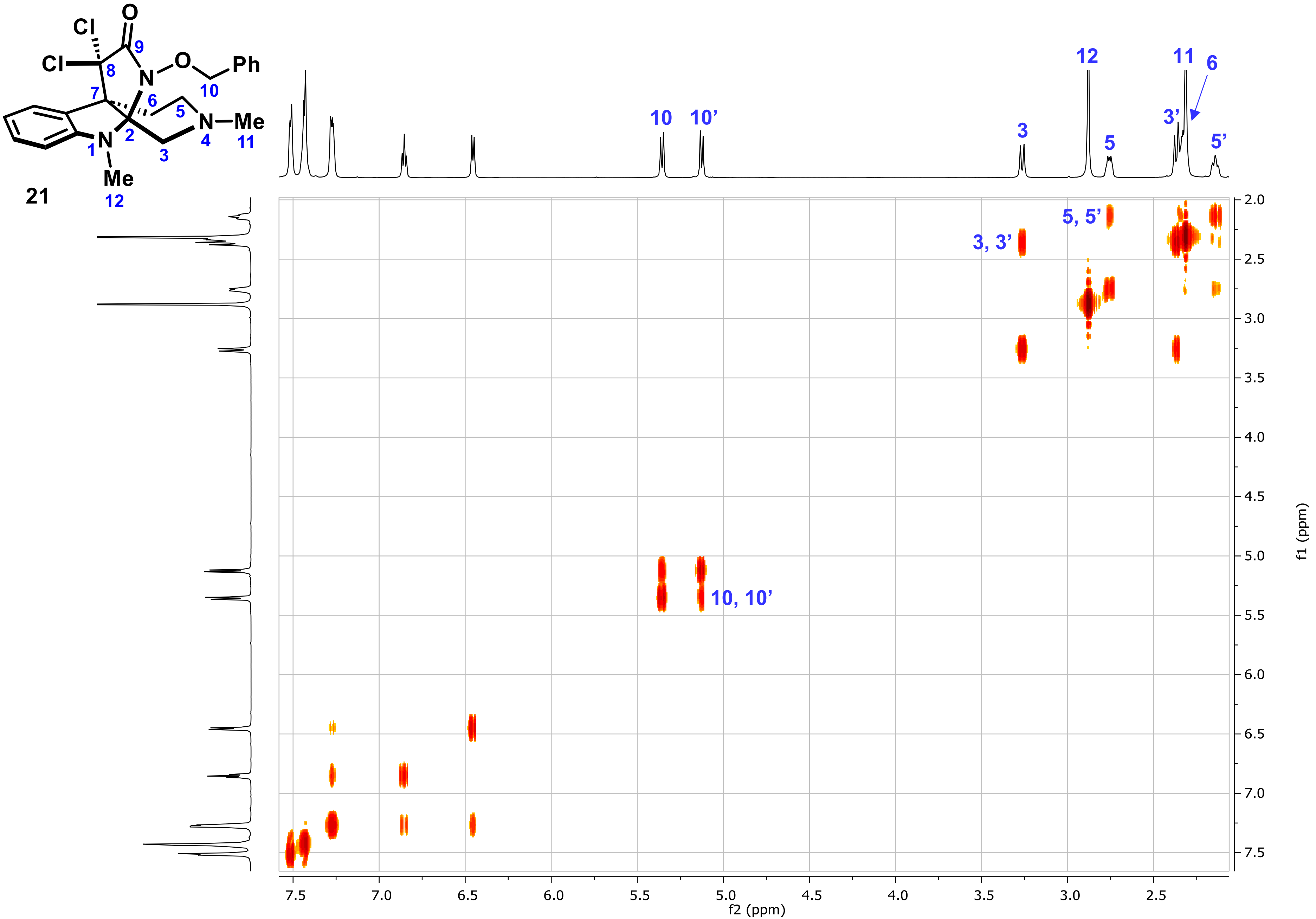
21
Cl
Cl
O
N
O
Ph
N
Me
Me
1
2
3
4
5
6
7
8
9
10
11
12
10
10’
3
12
5
3’
11
6
5’
5, 5’
3, 3’
10, 10’
f2 (ppm)
f1 (ppm)

COSY of 21

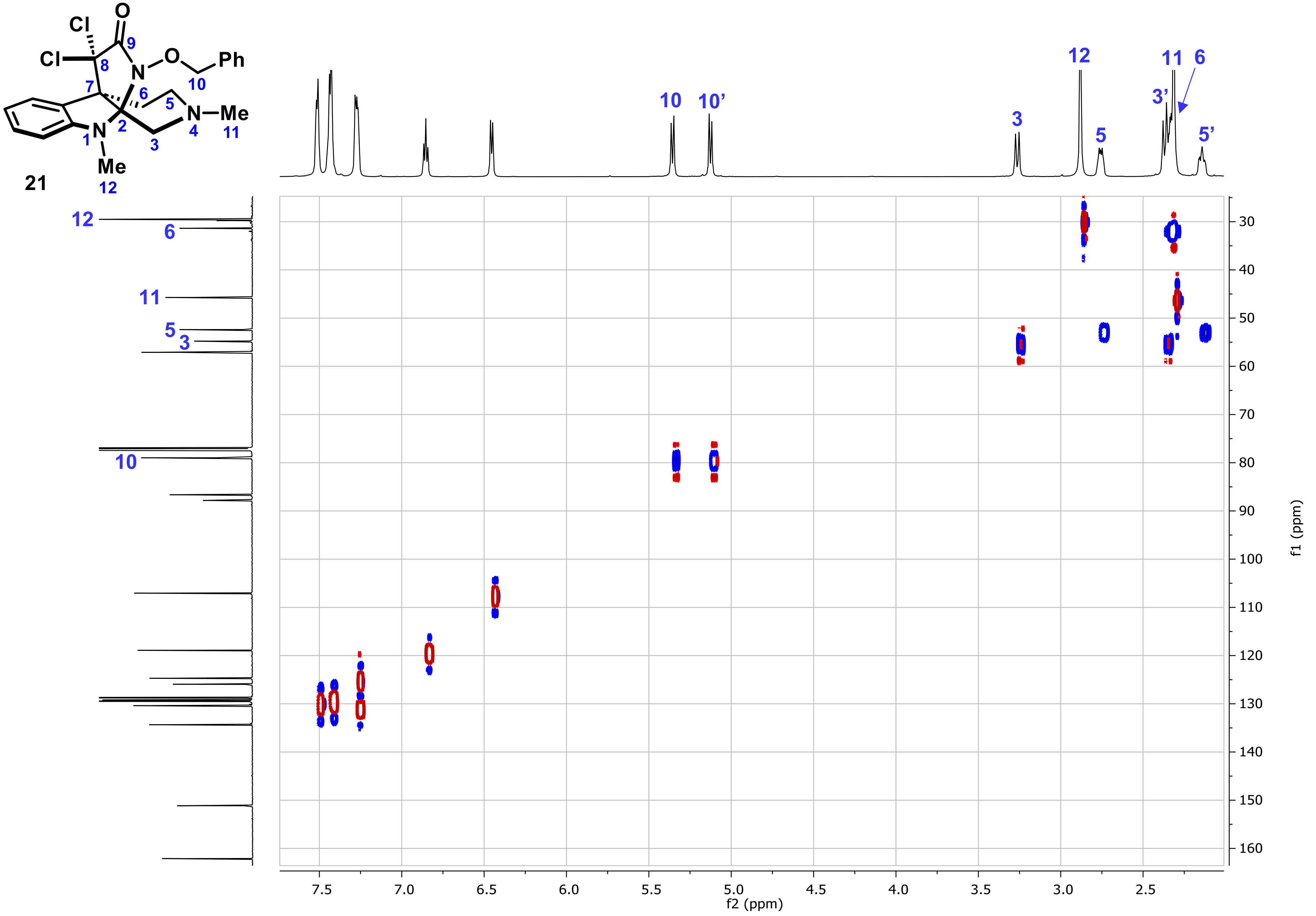

HSQC of 21

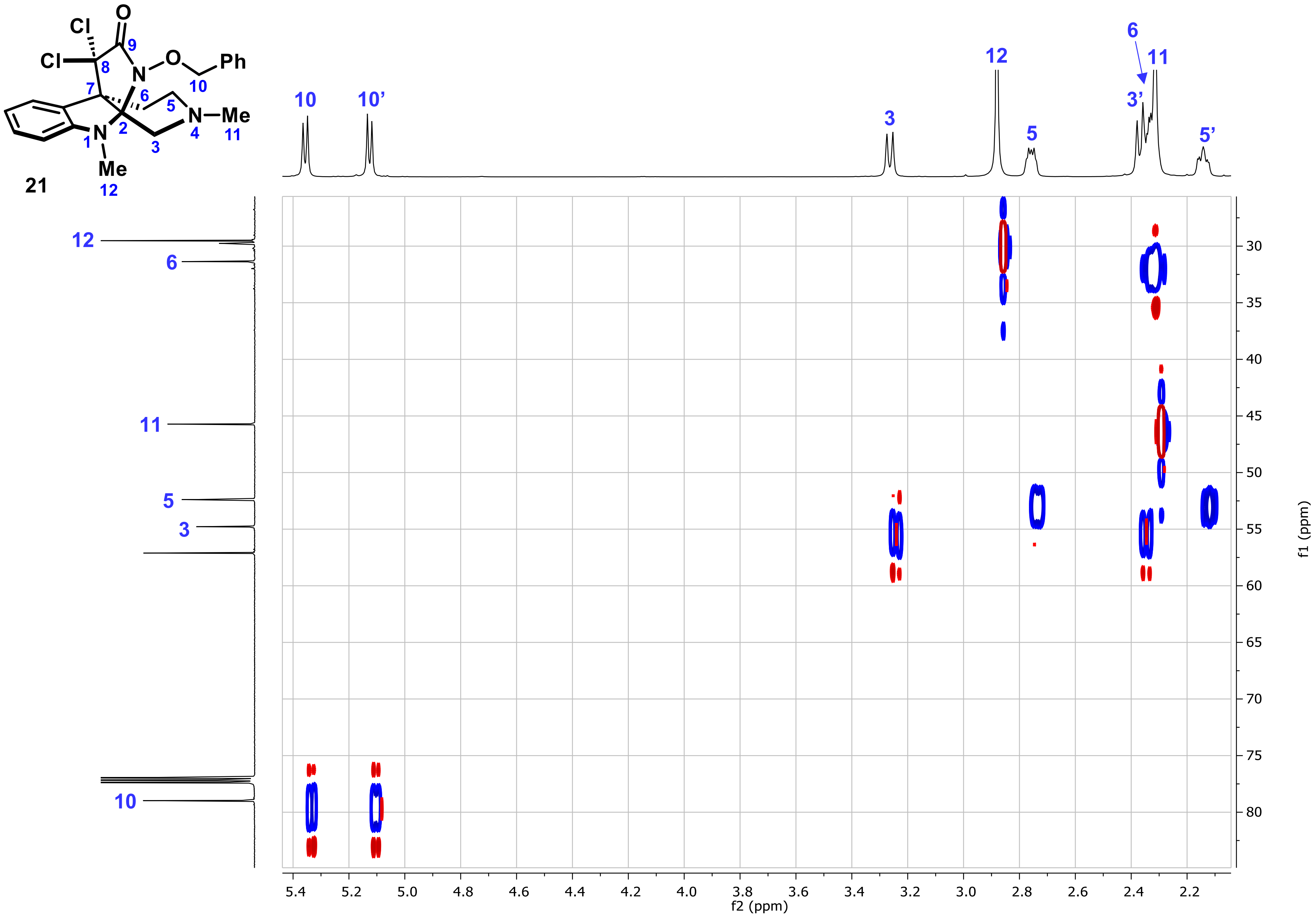

Zoomed In Version: HSQC of 21

NOTE: *In JMOD quaternary (-C-) and methylene (-CH_2-) signals have opposite phase to those of methine (-CH-) and methyl (-CH_3) resonances.*

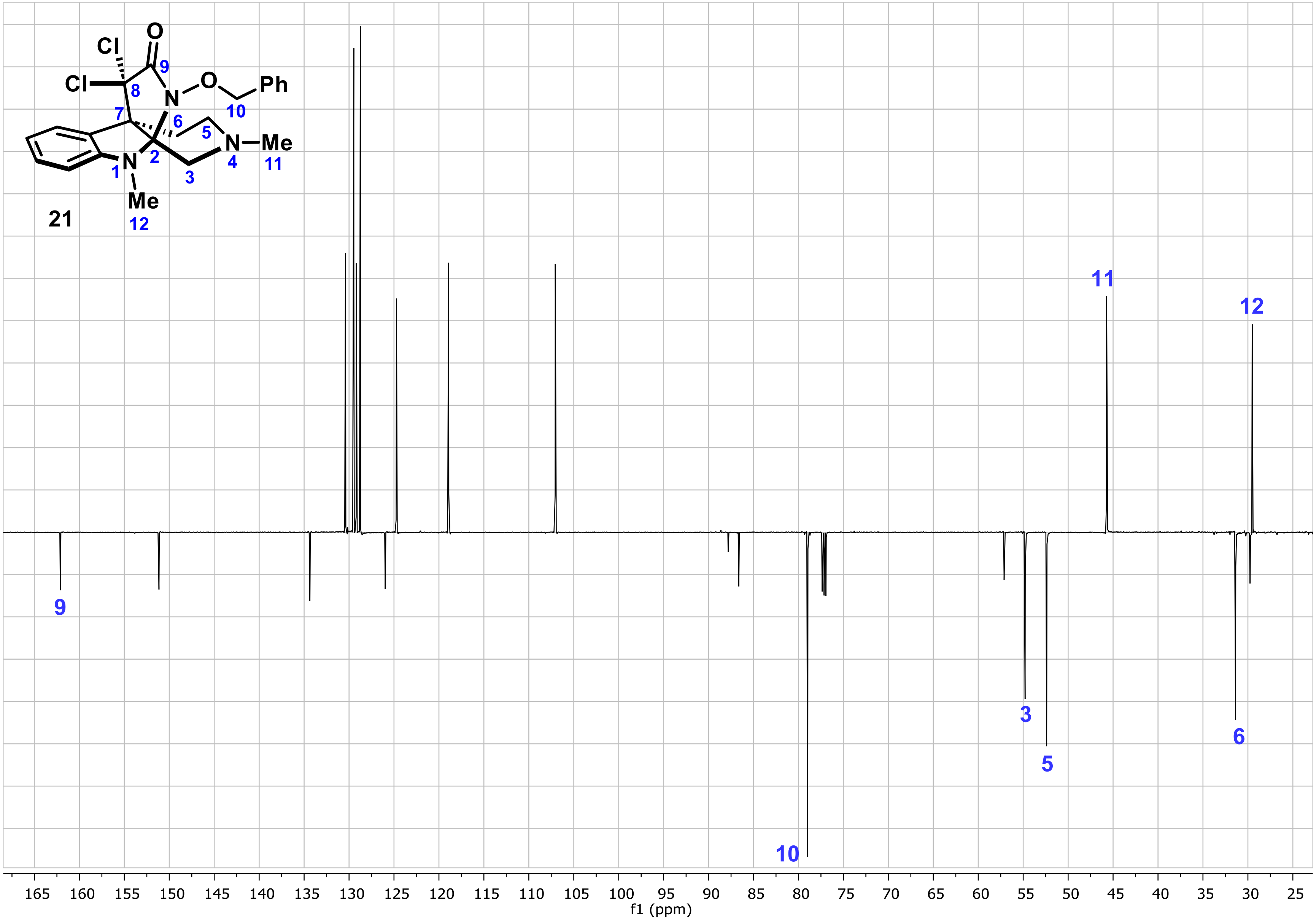

JMOD of 21

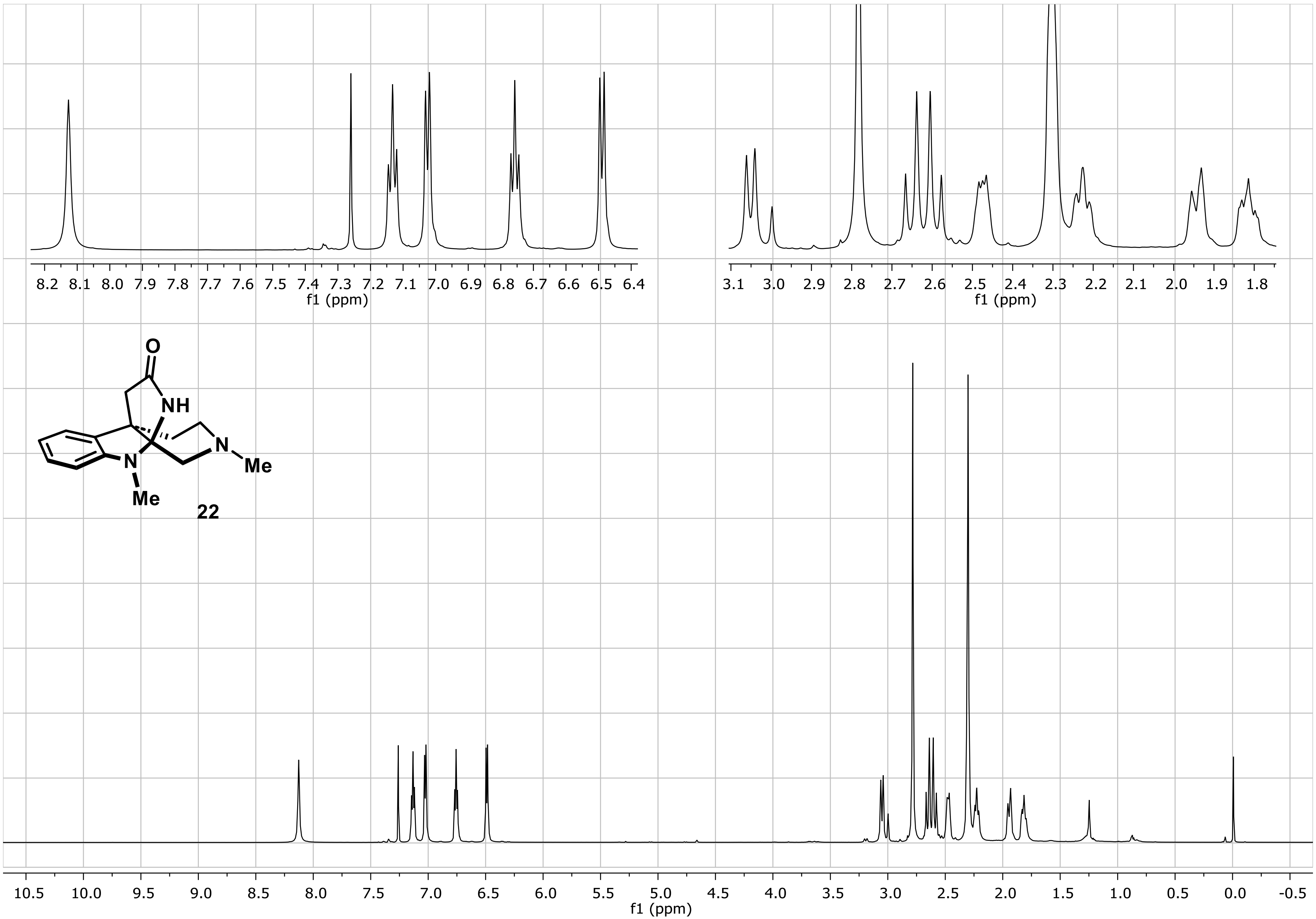
8.2 8.1 8.0 7.9 7.8 7.7 7.6 7.5 7.4 7.3 7.2 7.1 7.0 6.9 6.8 6.7 6.6 6.5 6.4
f1 (ppm)
3.1 3.0 2.9 2.8 2.7 2.6 2.5 2.4 2.3 2.2 2.1 2.0 1.9 1.8
f1 (ppm)
O
NH
N
Me
N
Me
22
10.5 10.0 9.5 9.0 8.5 8.0 7.5 7.0 6.5 6.0 5.5 5.0 4.5 4.0 3.5 3.0 2.5 2.0 1.5 1.0 0.5 0.0 -0.5
f1 (ppm)

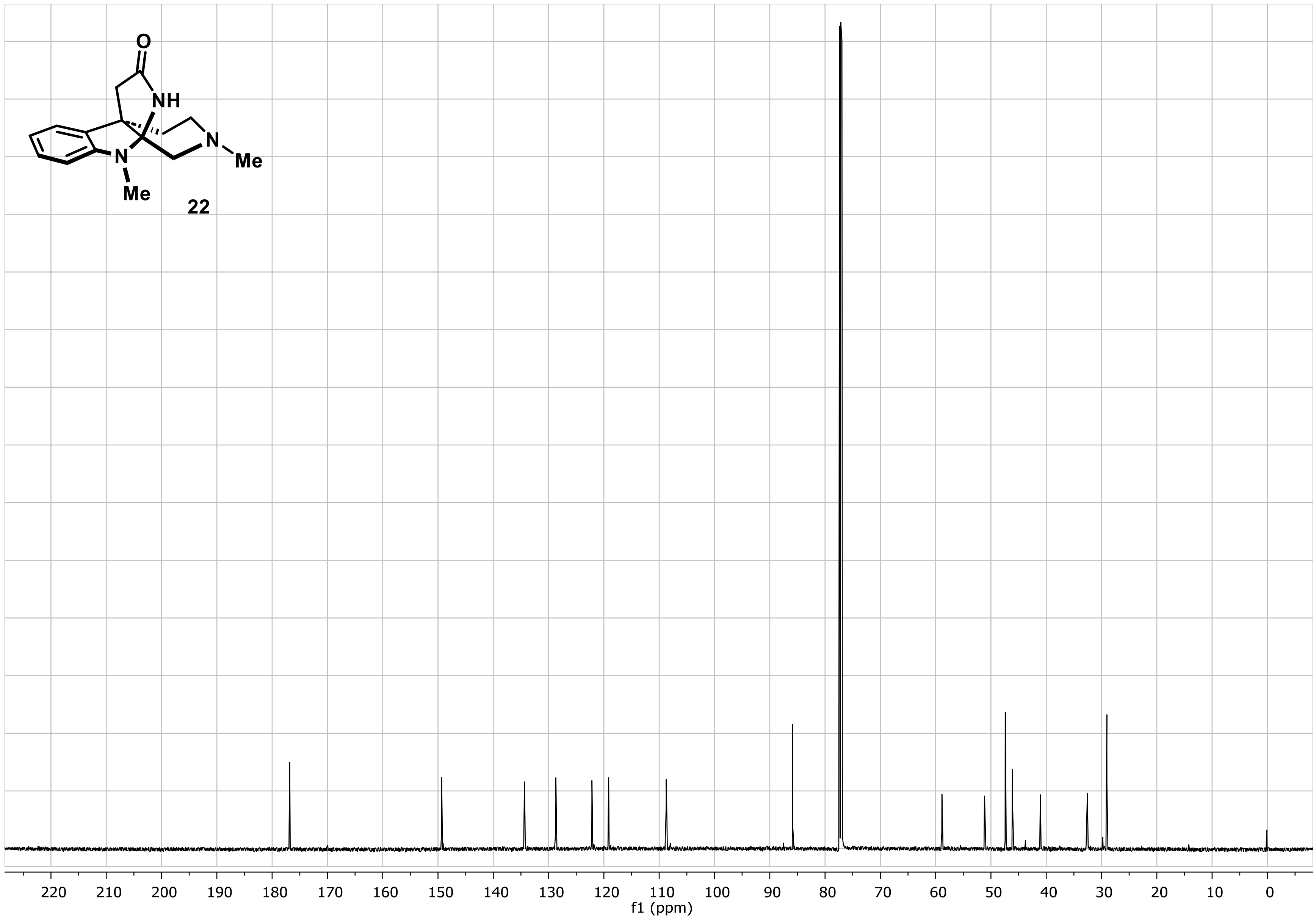

O
NH
N
Me
N
Me
22
220
210
200
190
180
170
160
150
140
130
120
110
100
90
80
70
60
50
40
30
20
10
0
f1 (ppm)

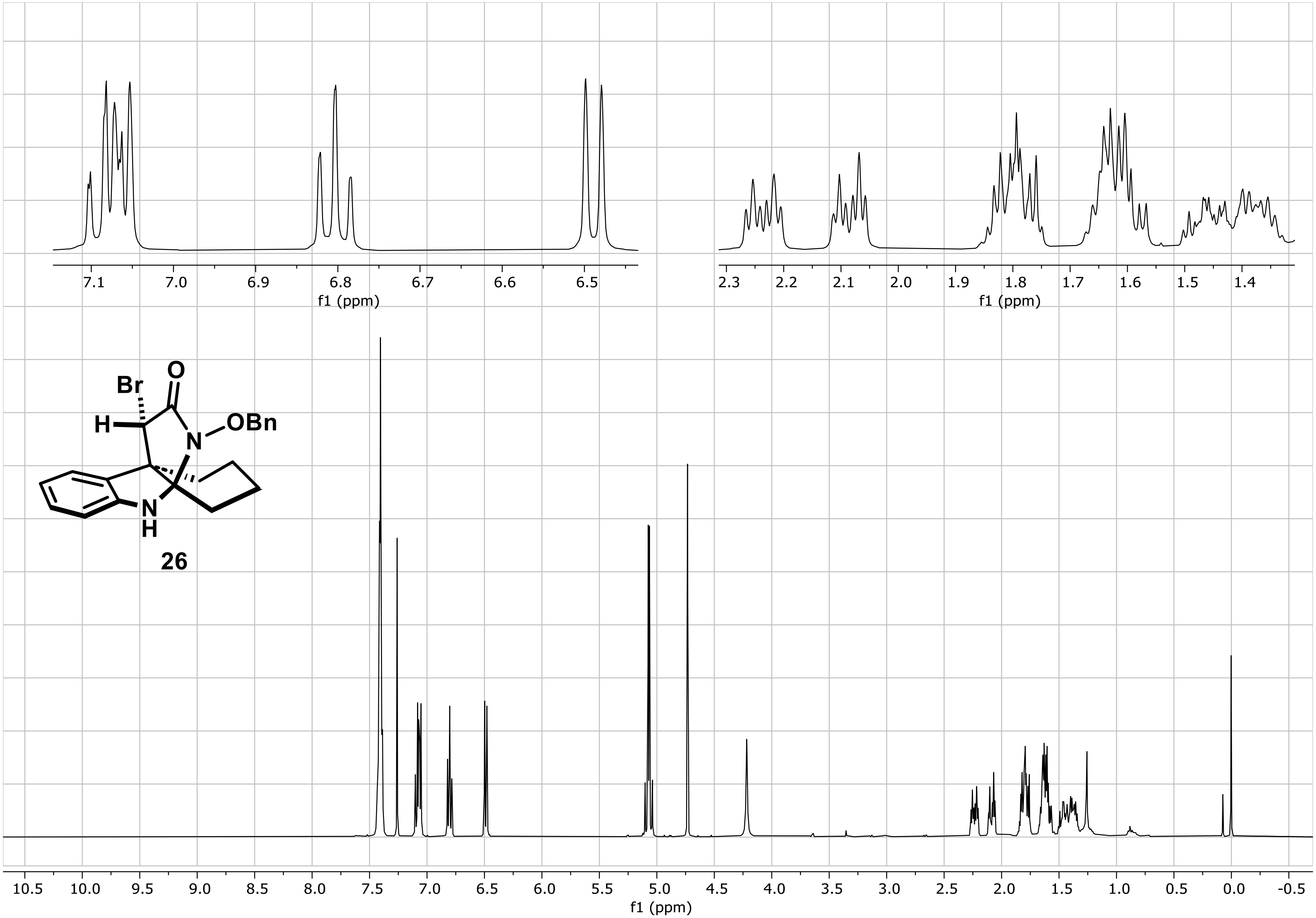
Br
O
H
N
OBn
N
H
26
f1 (ppm)
f1 (ppm)
f1 (ppm)

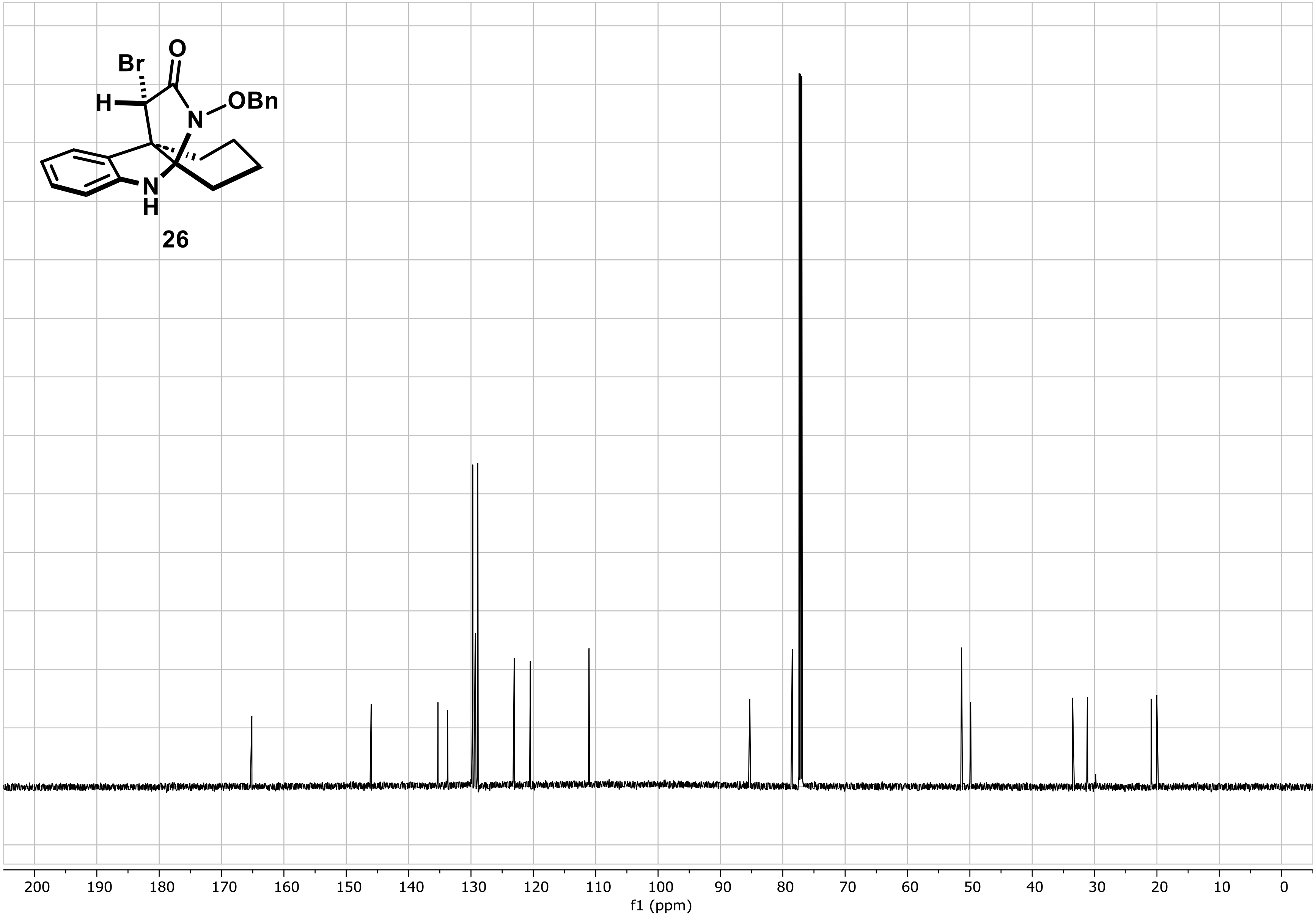
Br
O
H
N
OBn
N
H
26
200 190 180 170 160 150 140 130 120 110 100 90 80 70 60 50 40 30 20 10 0
f1 (ppm)

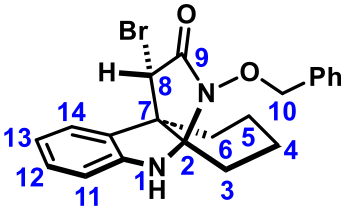

26

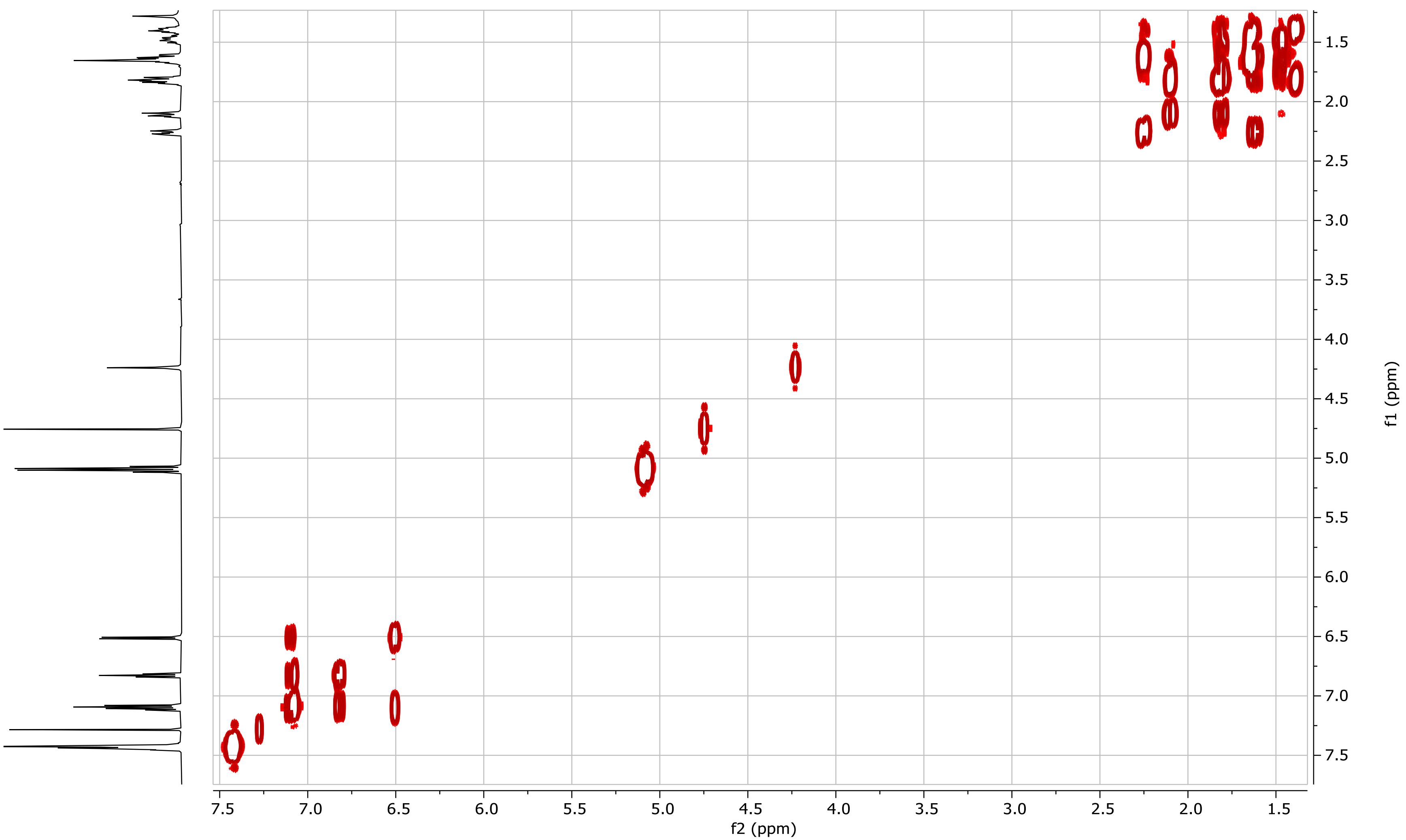

Full COSY of 26

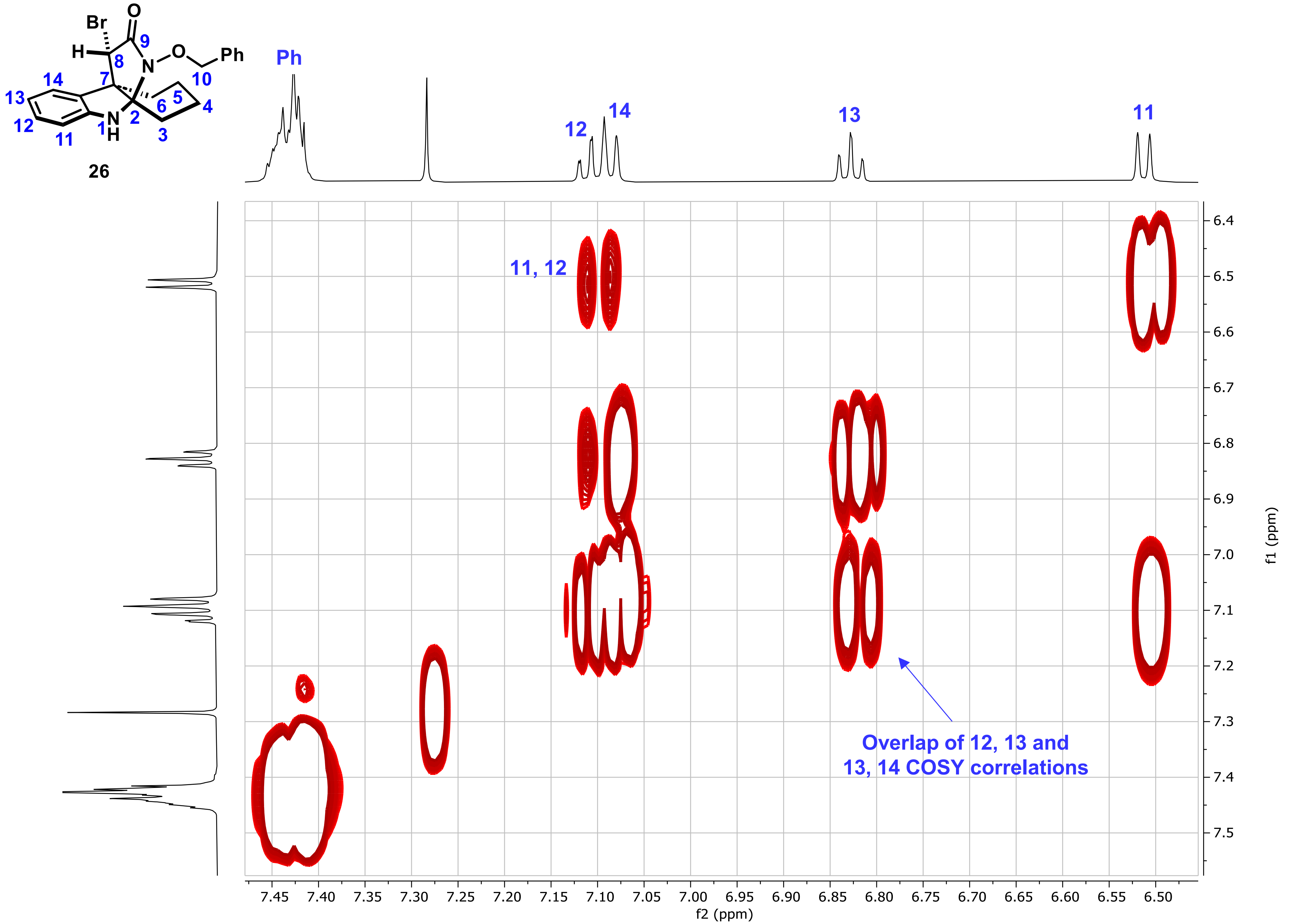

26

Zoomed In Version 1: COSY of 26

Unable to differentiate the 12, 13 & 13, 14 COSY correlations
Splitting patters were important in assignments

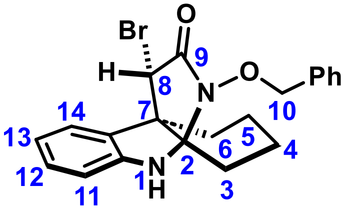

26

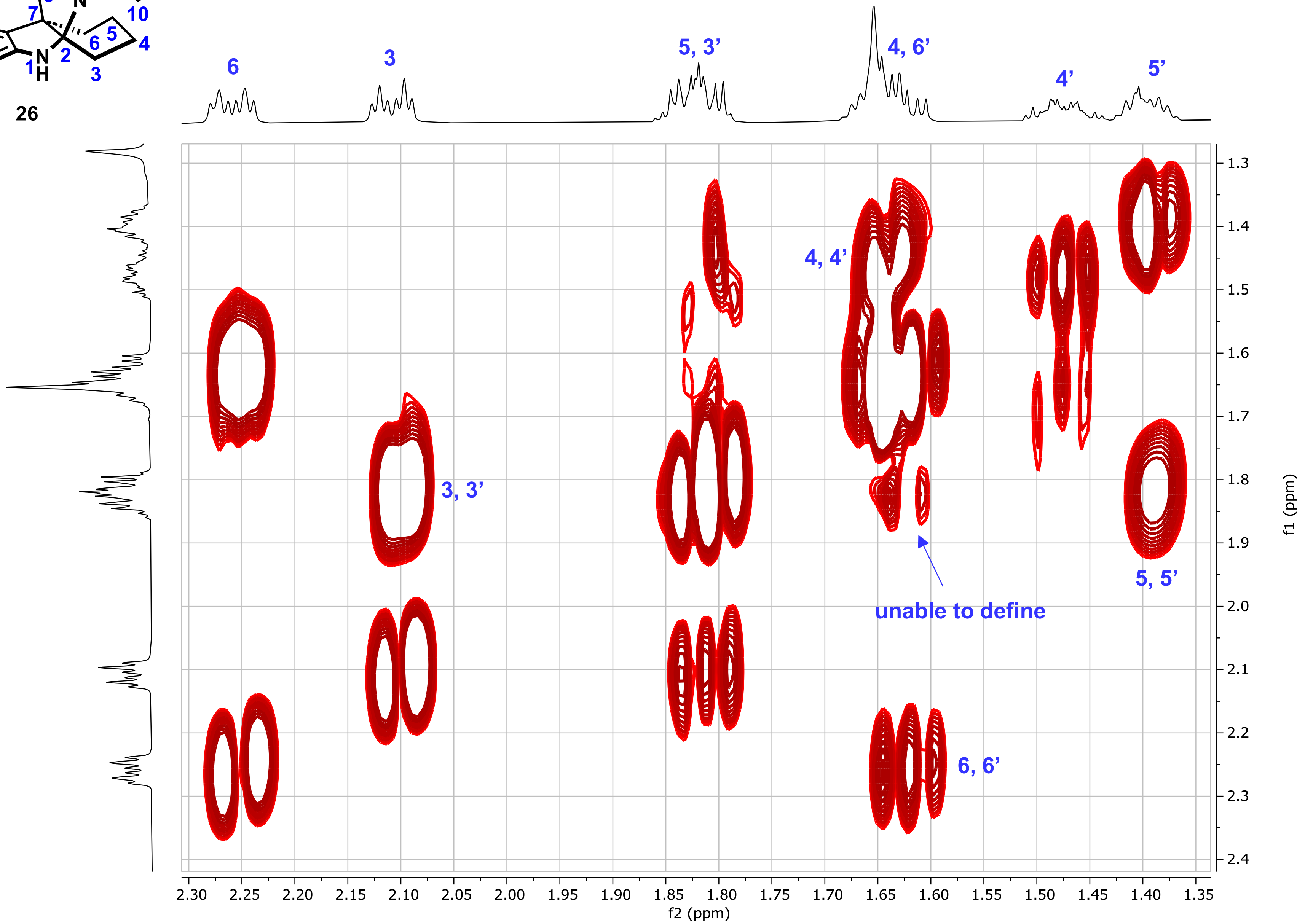

Zoomed In Version 2: COSY of 26

Positions 4 & 5: Determined by HMBC

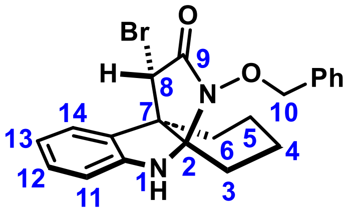
Br
O
H
9
8
N
O
Ph
7
10
14
13
6
5
4
12
N
1
H
2
11
3

26

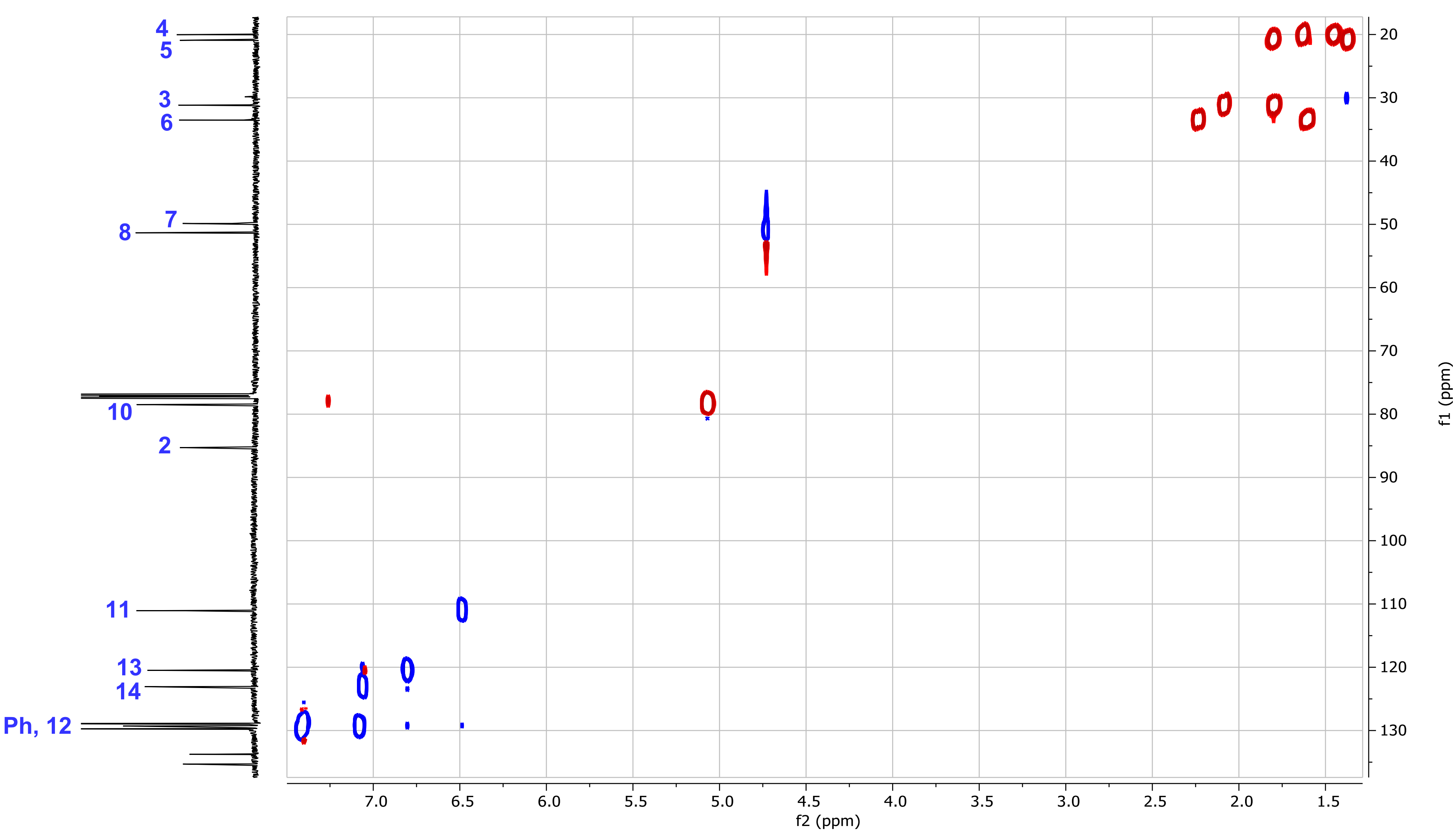
Ph
12, 14
13
11
10
8
N-H
6
3
5, 3’
4, 6’
4’
5’
4
5
3
6
7
8
10
2
11
13
14
Ph, 12
f2 (ppm)
f1 (ppm)

HSQC of 26

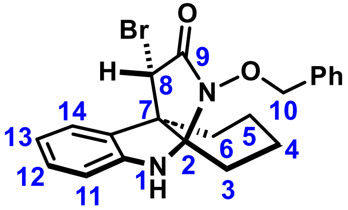

26

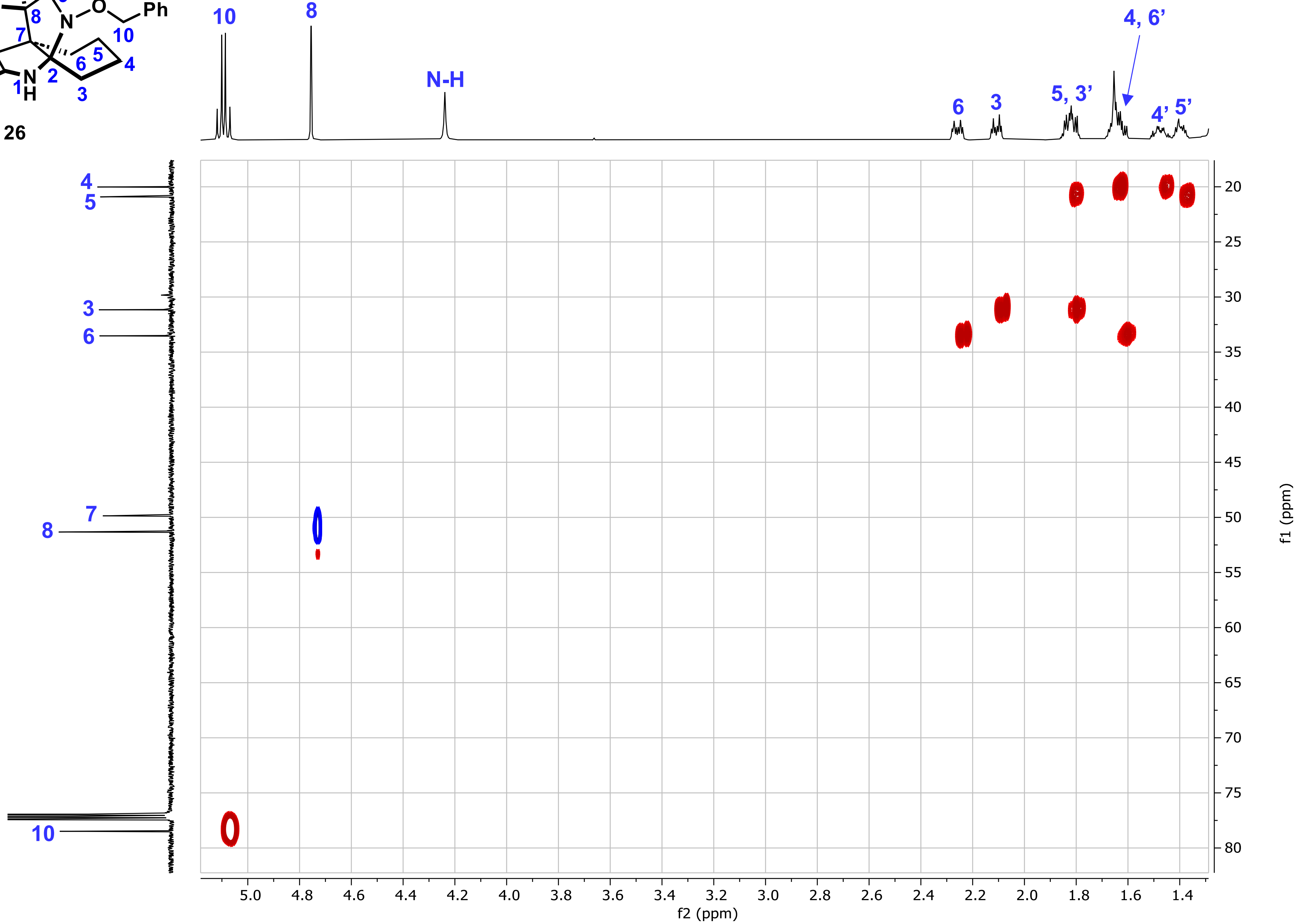

Zoomed In Version 1: HSQC of 26

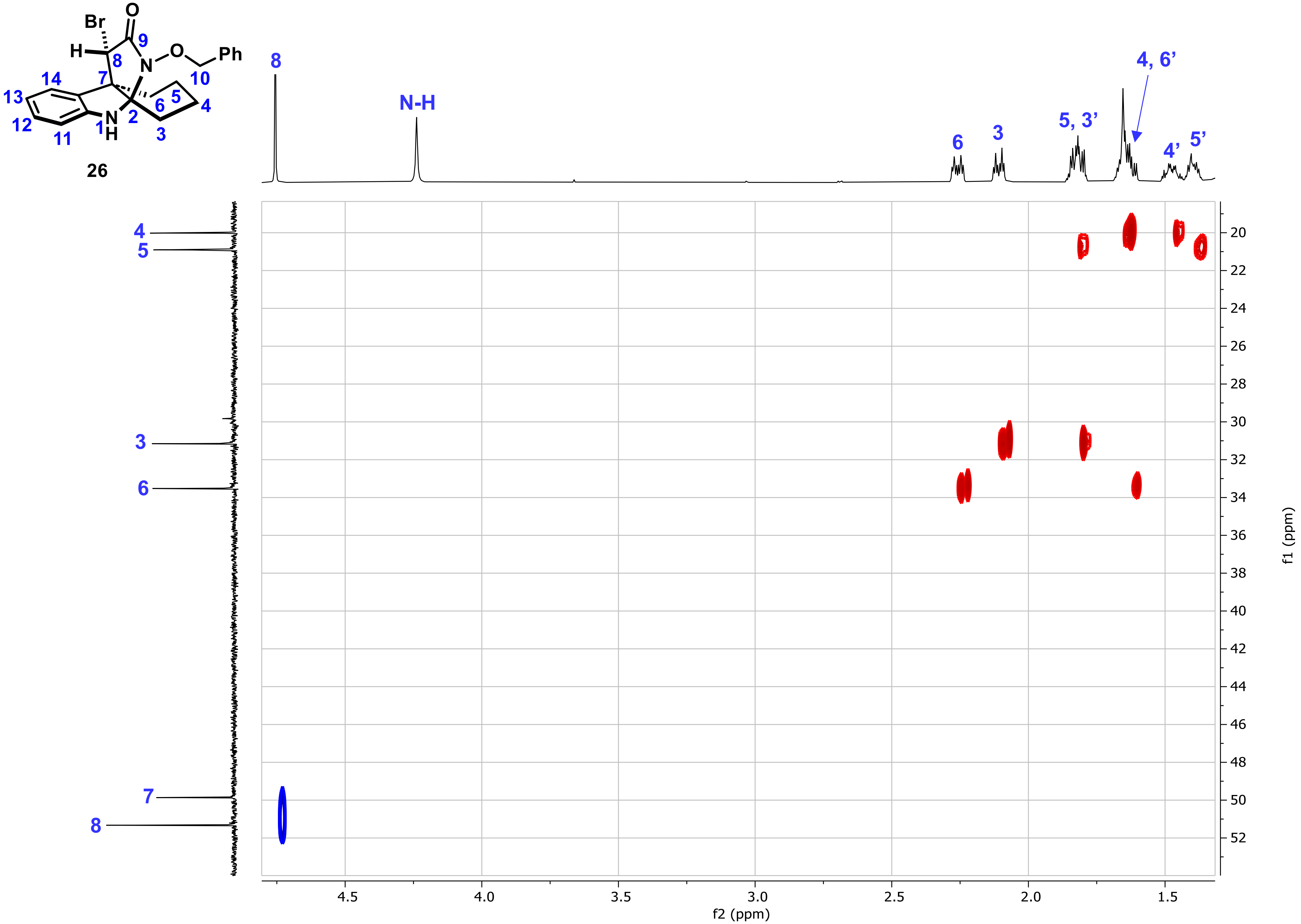

Zoomed In Version 2: HSQC of 26

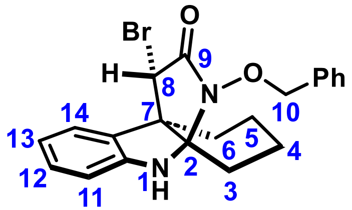

26

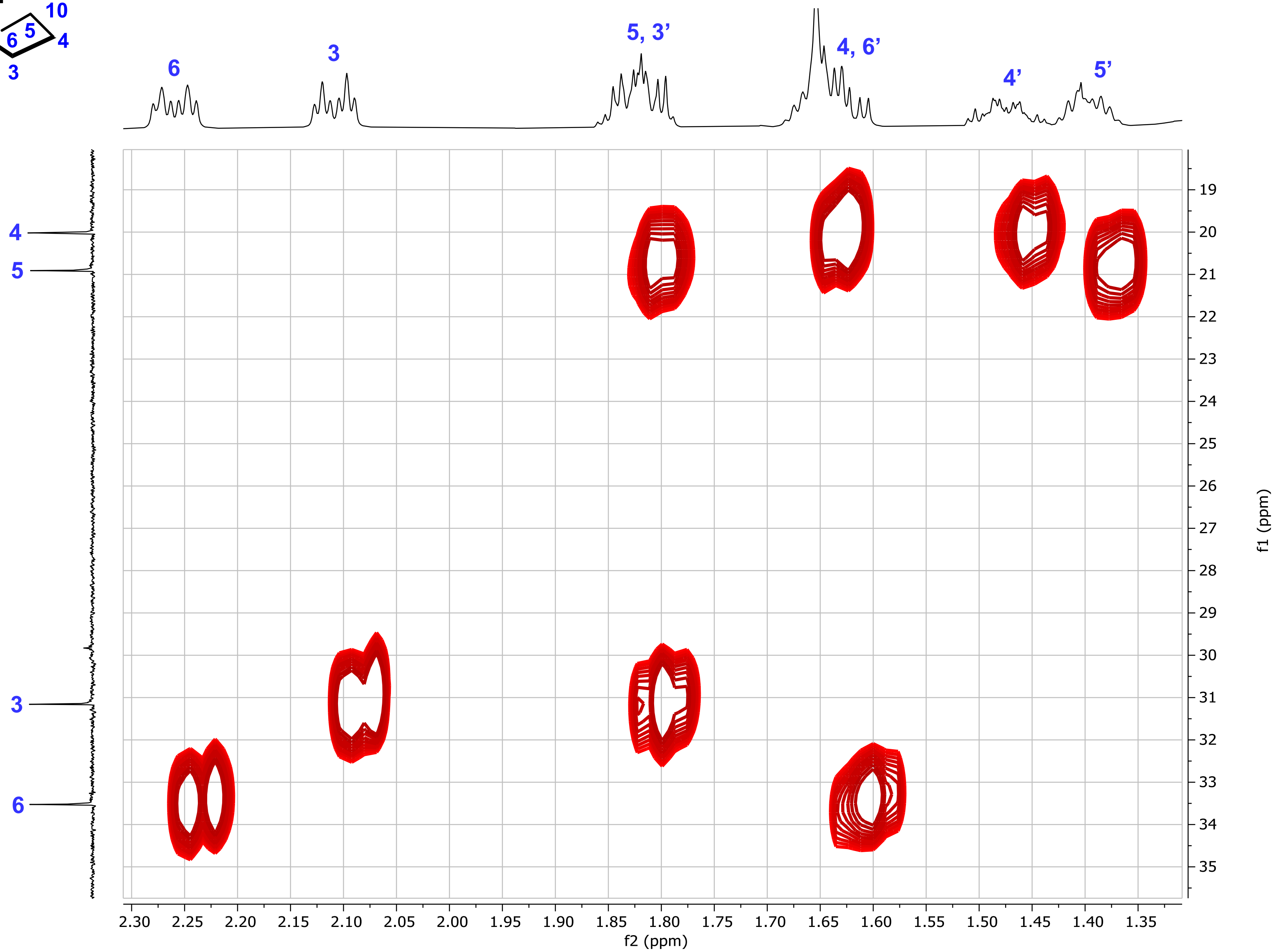

Zoomed In Version 3: HSQC of 26

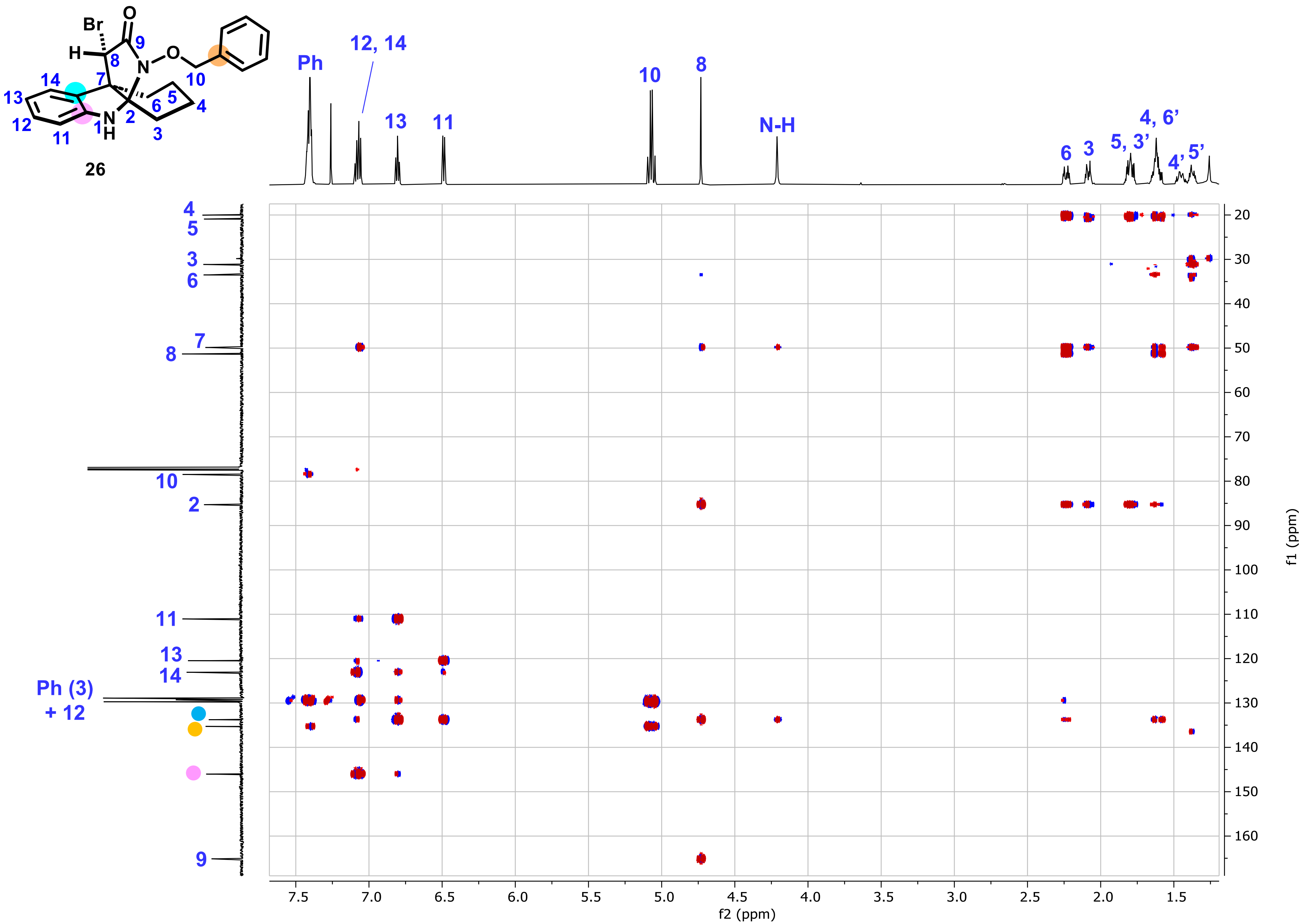

Br
O
H
8
9
N
O
10
14
7
13
5
6
4
12
N
2
11
1
H
3
26
Ph
12, 14
13
11
10
8
N-H
6
3
5, 3'
4, 6'
4', 5'
4
5
3
6
7
8
10
2
11
13
14
Ph (3)
+ 12
9
f1 (ppm)
f2 (ppm)

HMBC of 26

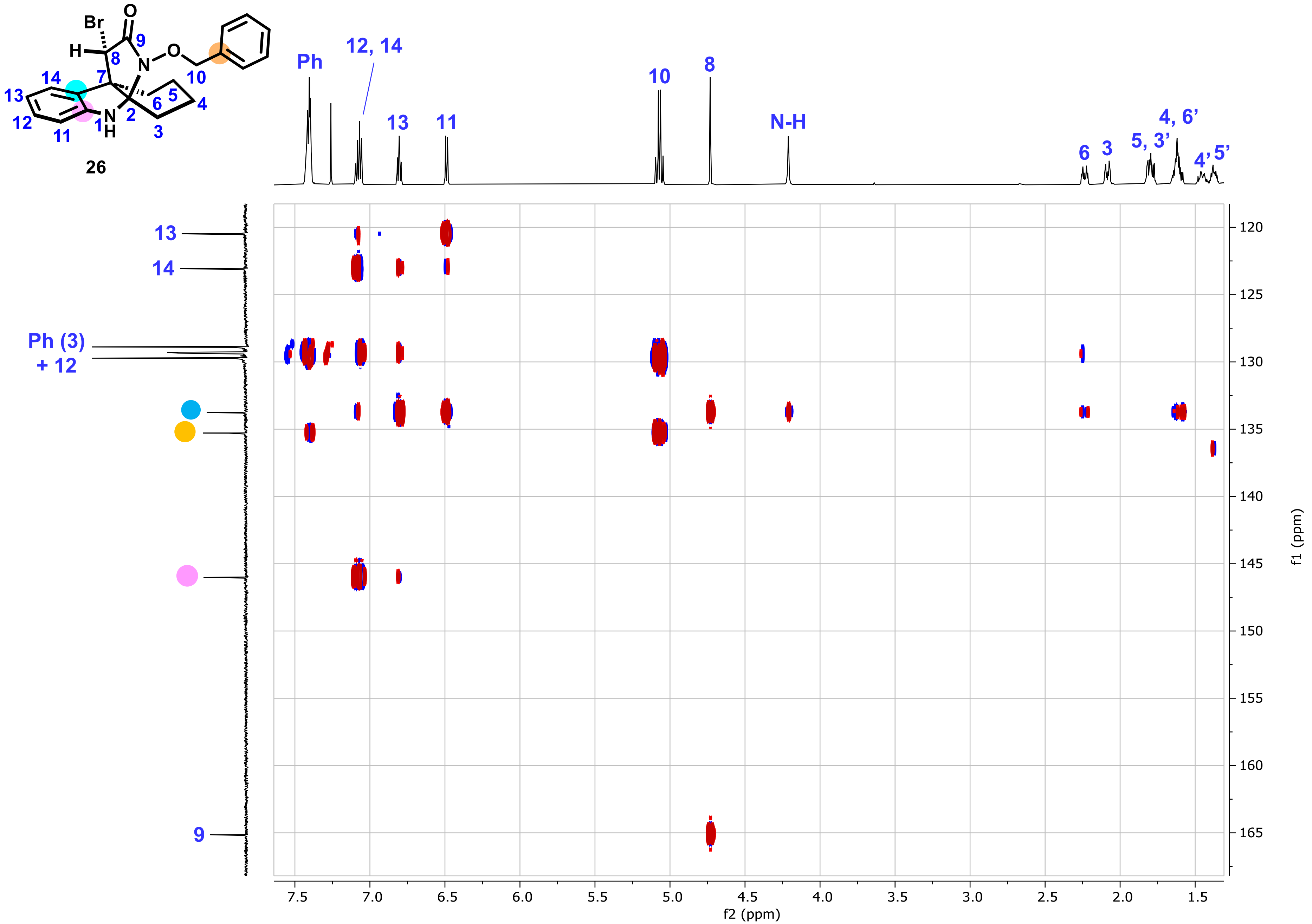

Zoomed In Version 1: HMBC of 26

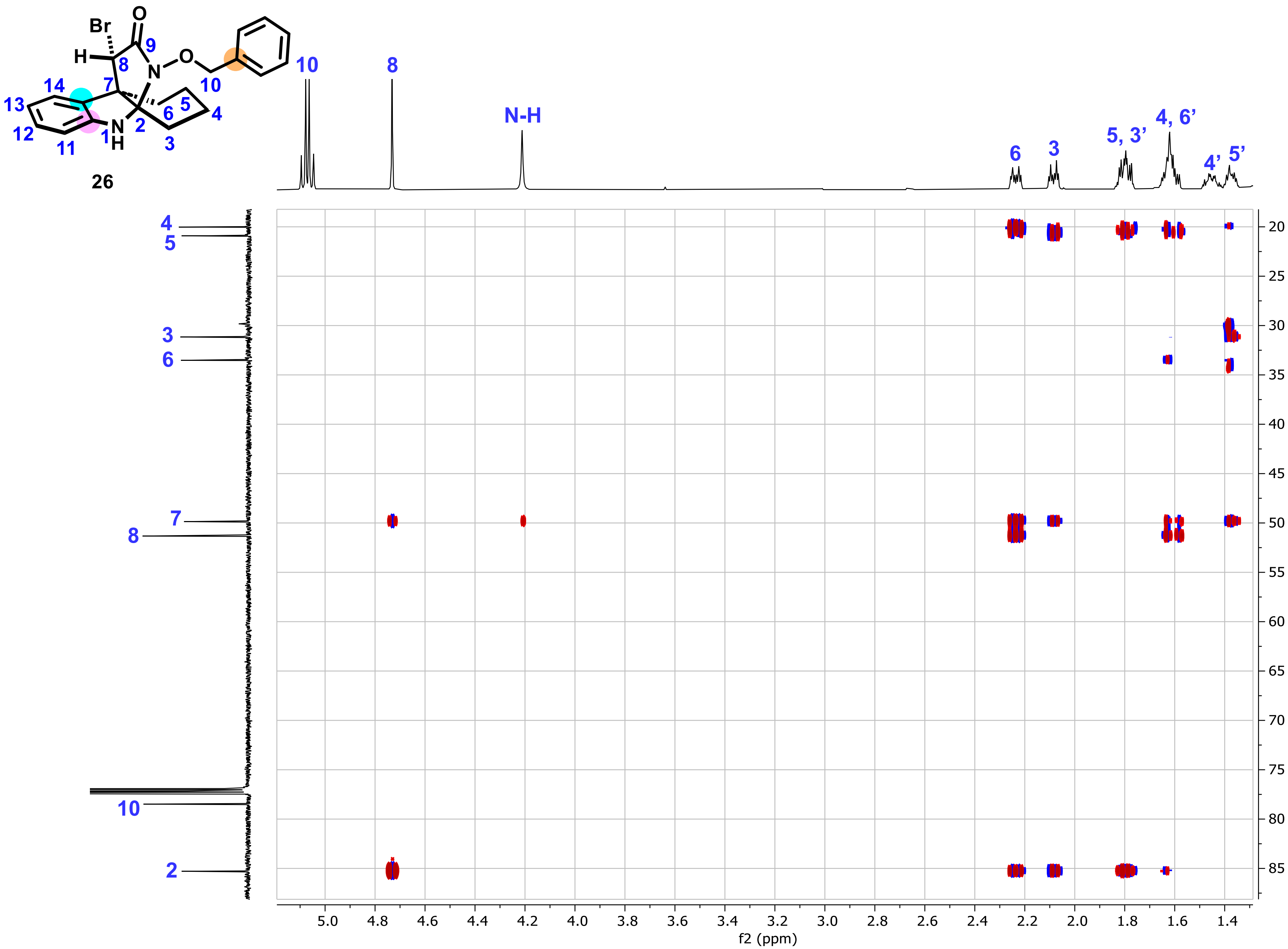

Zoomed In Version 2: HMBC of 26

clean HMBCs of protons at positions 1 & 8 to C7

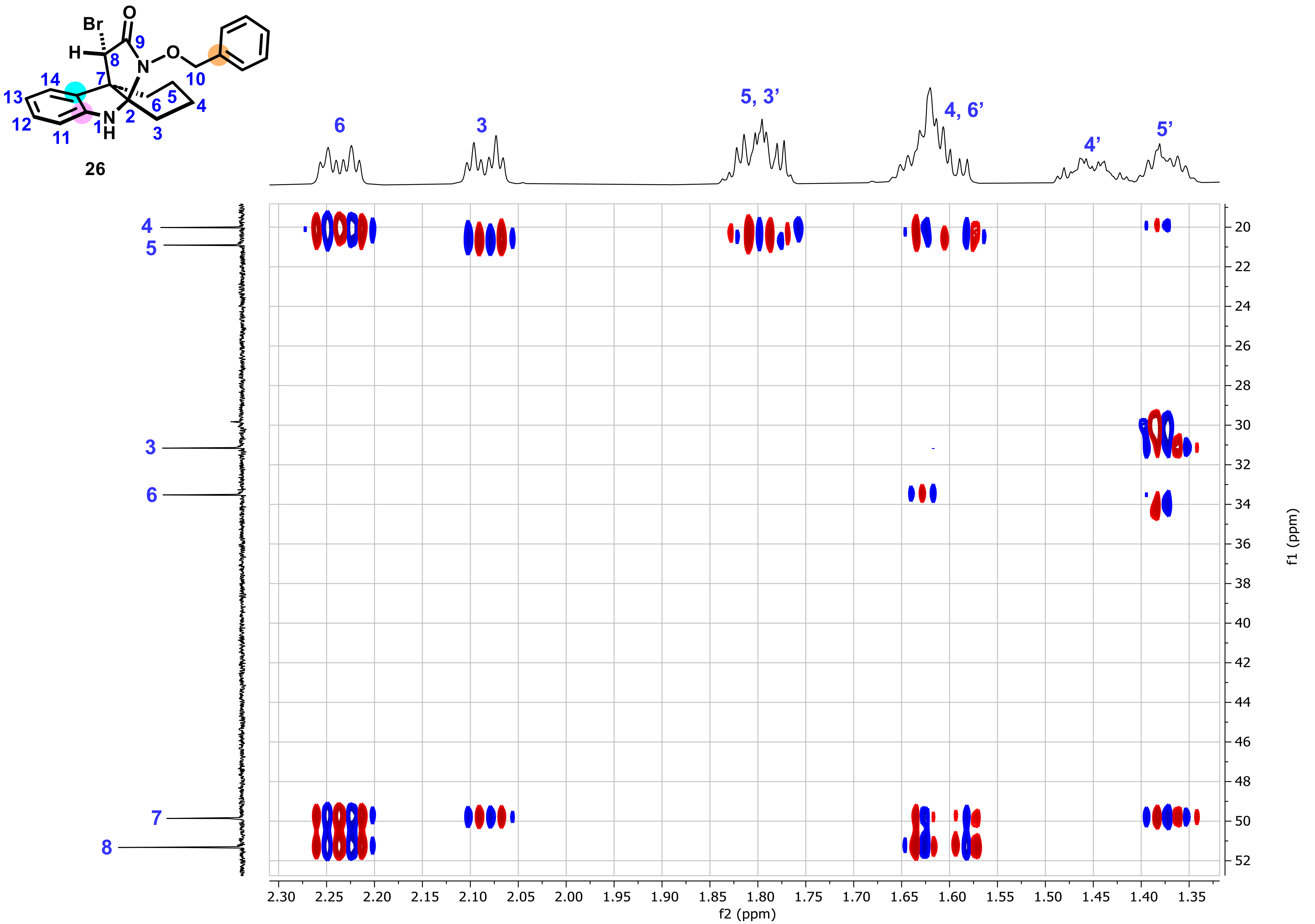

Zoomed In Version 3: HMBC of 26

Key HMBCs to Define Positions 4 & 5: H6 to C4, H3 to C5, H3 to C7, H4 to C6, H5' to C3, H5' to C7

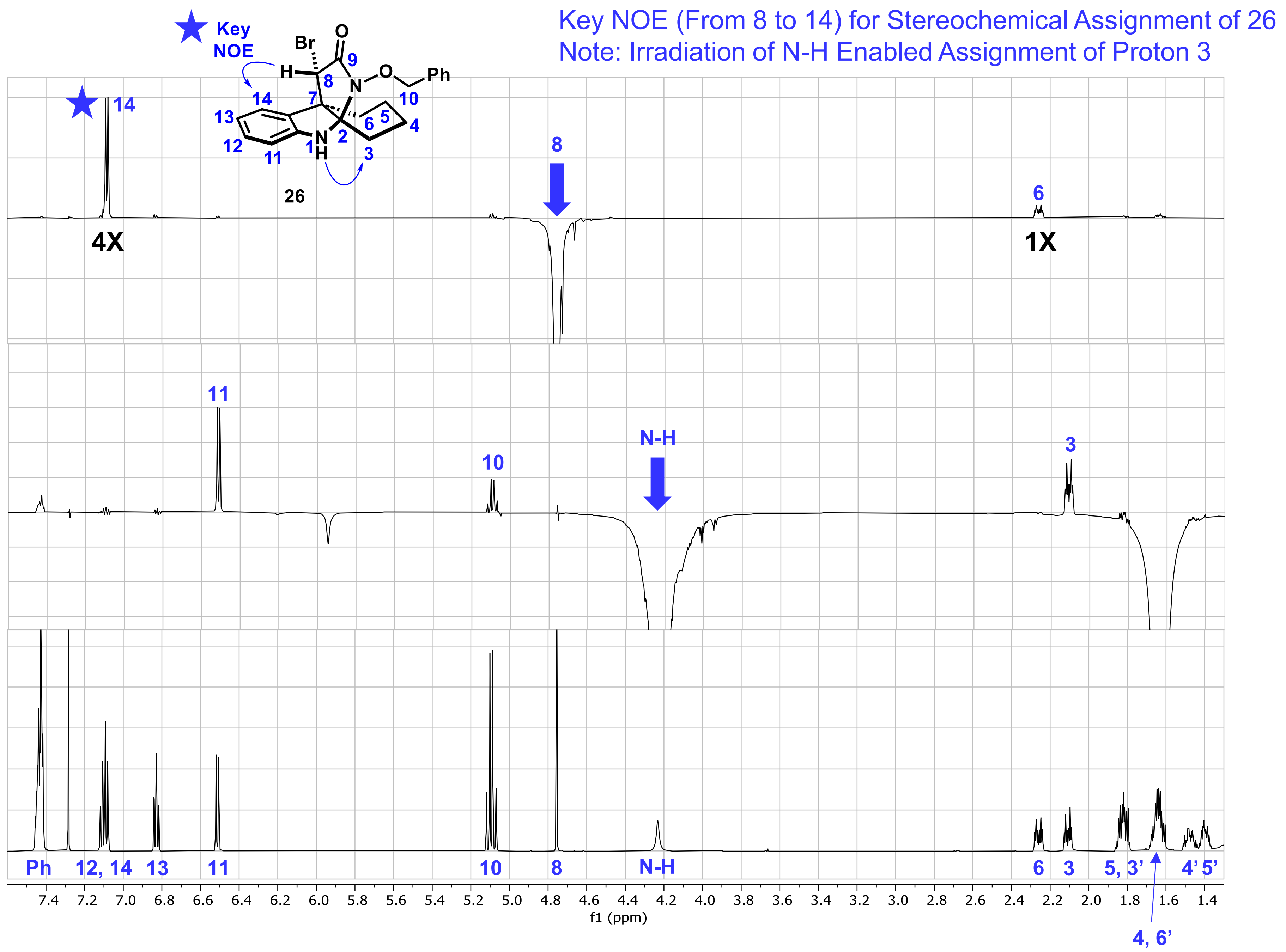
Key NOE (From 8 to 14) for Stereochemical Assignment of 26
Note: Irradiation of N-H Enabled Assignment of Proton 3
Key NOE
26
4X
1X
Ph
12, 14
13
11
10
8
N-H
6
3
5, 3'
4' 5'
4, 6'
f1 (ppm)

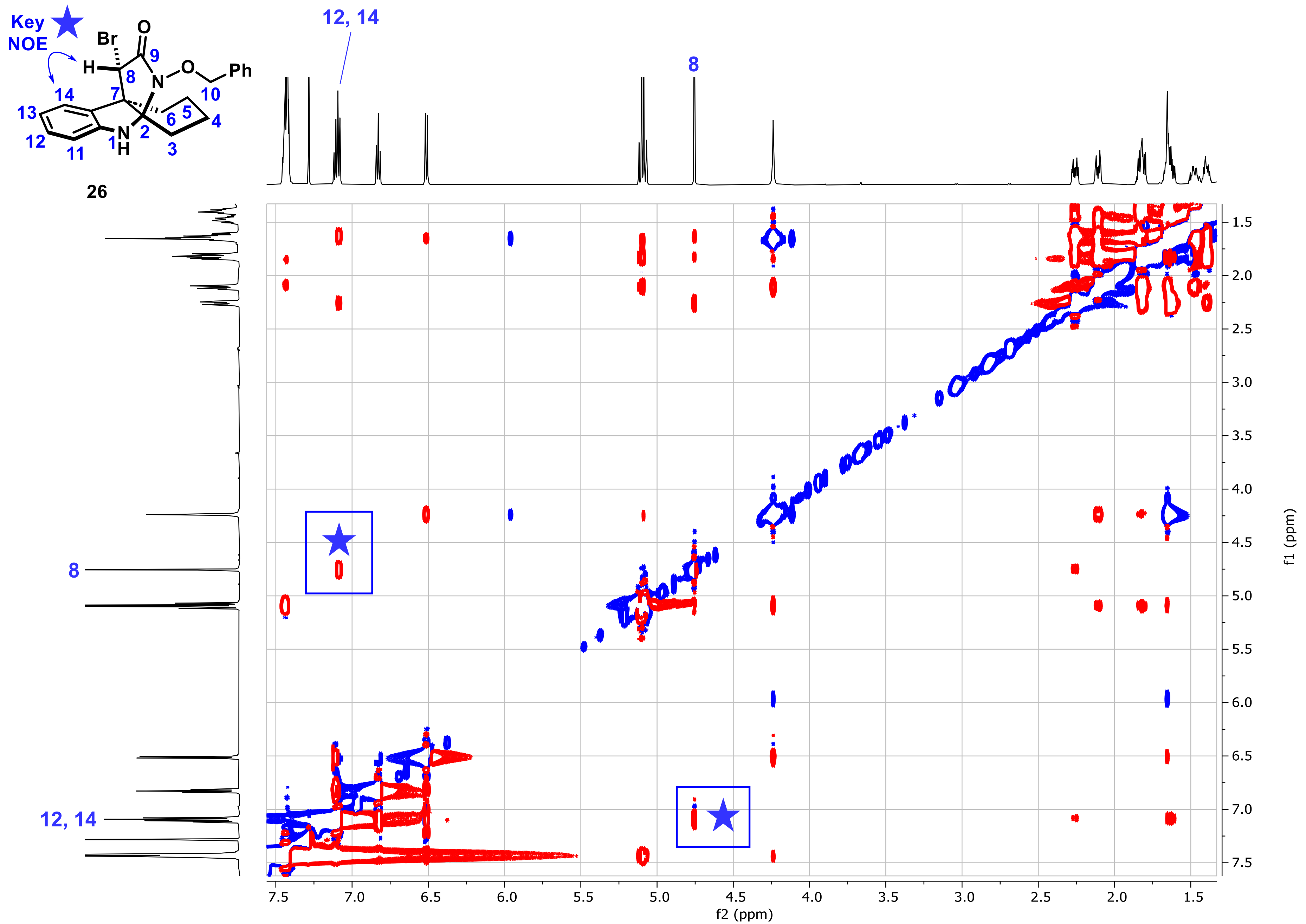

2D ROEs for Stereochemical Assignment of 26
(showing key 2D ROEs between 8 & 14)

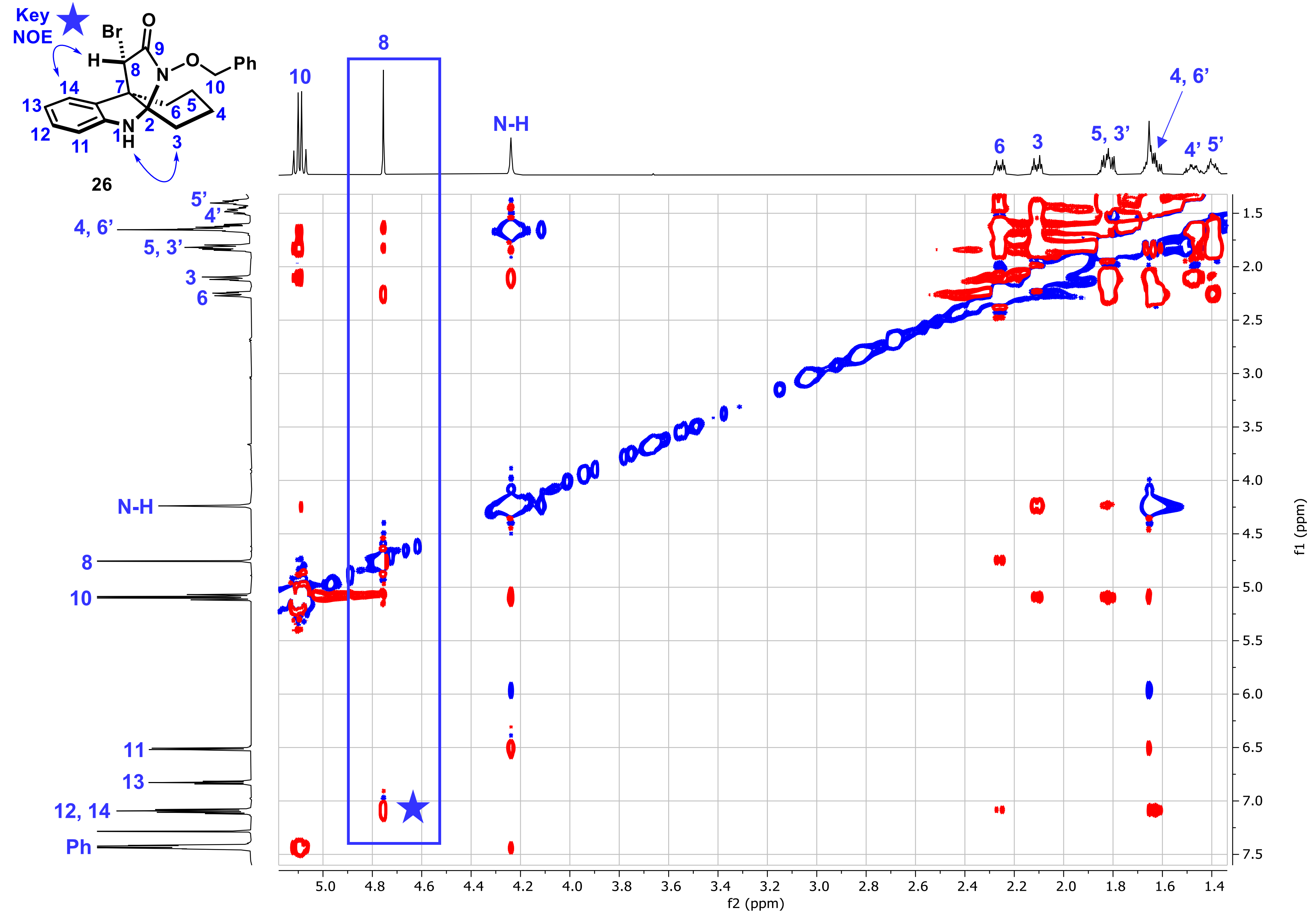

Zoomed In Version: 2D ROEs for Stereochemical Assignment of 26 (Strong ROE Between 8 & 14)

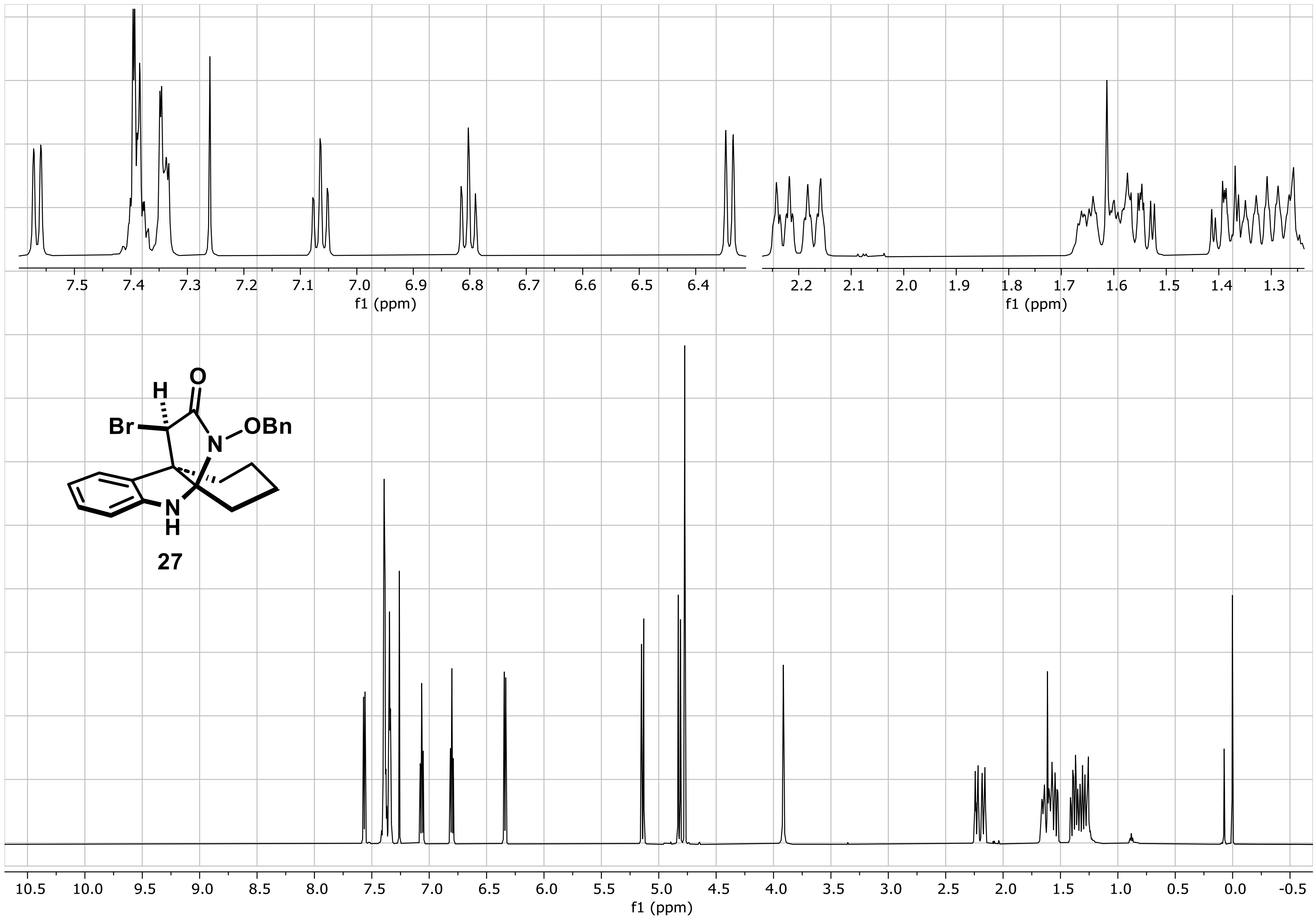

Br
H
O
N
OBn
N
H
27
f1 (ppm)
f1 (ppm)
f1 (ppm)

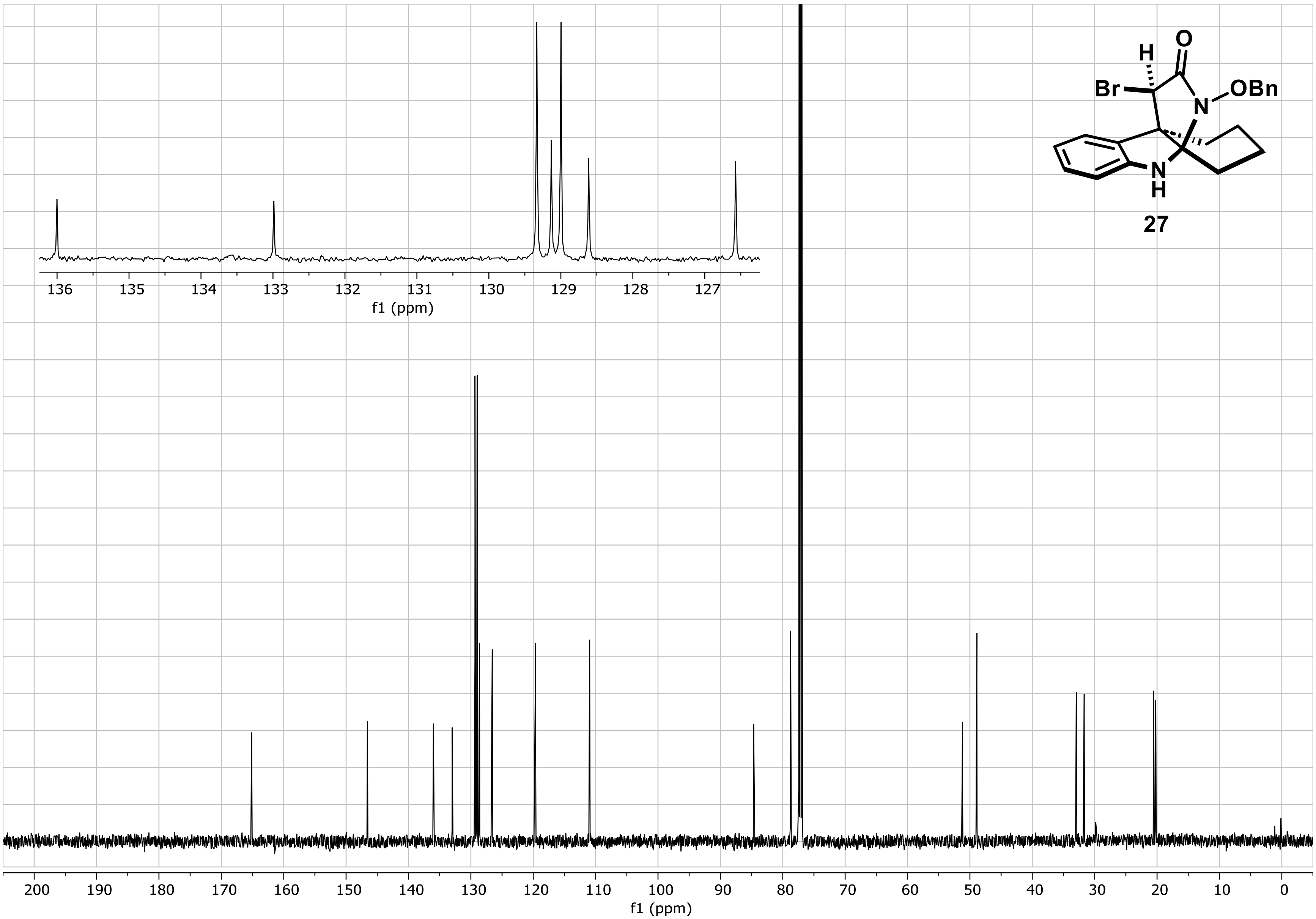
136 135 134 133 132 131 130 129 128 127
f1 (ppm)
H
O
Br
N
OBn
N
H
27
200 190 180 170 160 150 140 130 120 110 100 90 80 70 60 50 40 30 20 10 0
f1 (ppm)

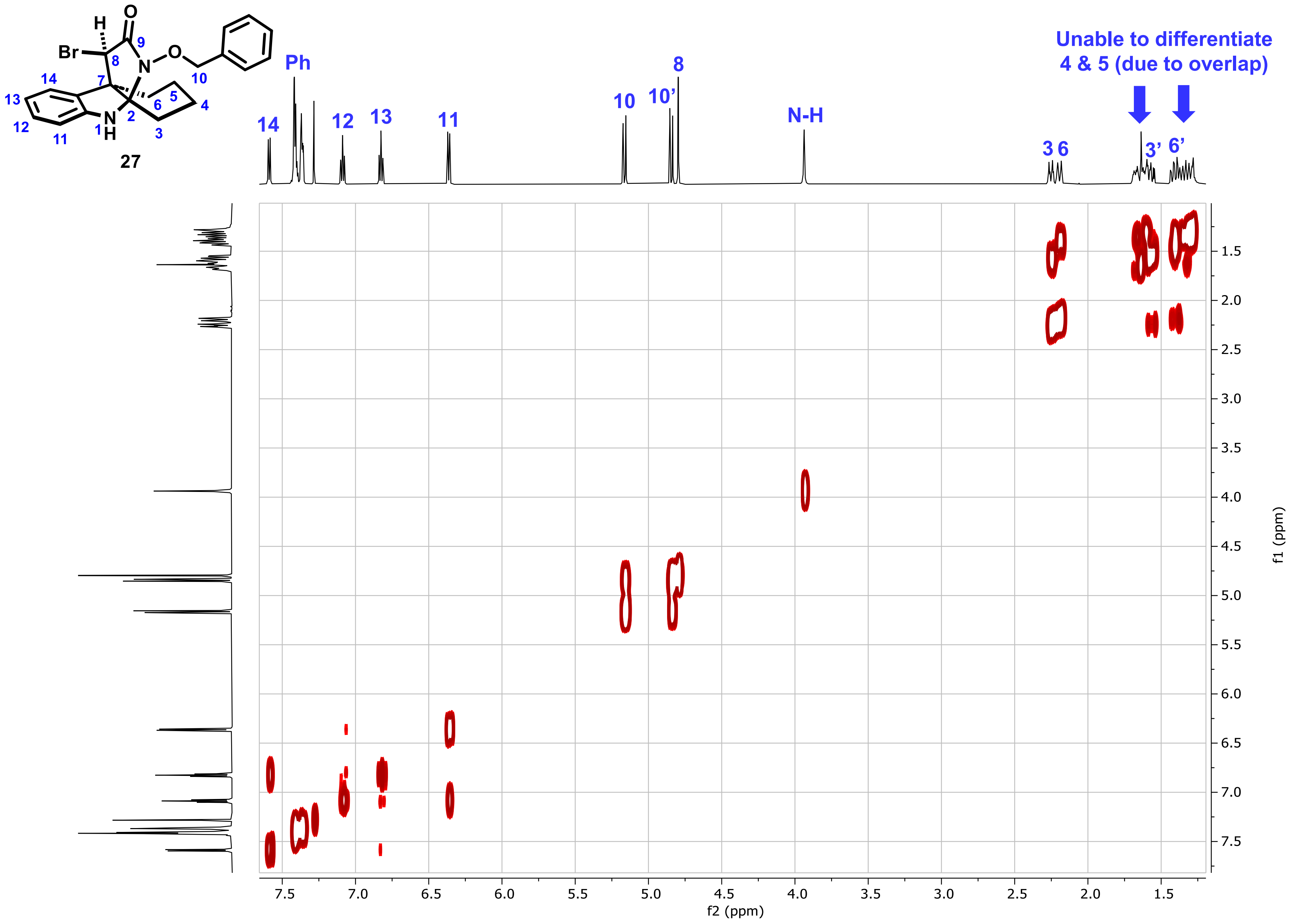
Br
H
O
N
O
NH
H
1
2
3
4
5
6
7
8
9
10
11
12
13
14
27
Ph
14
12
13
11
10
10'
8
N-H
3 6
3'
6'
Unable to differentiate
4 & 5 (due to overlap)
f2 (ppm)
f1 (ppm)

COSY of 27

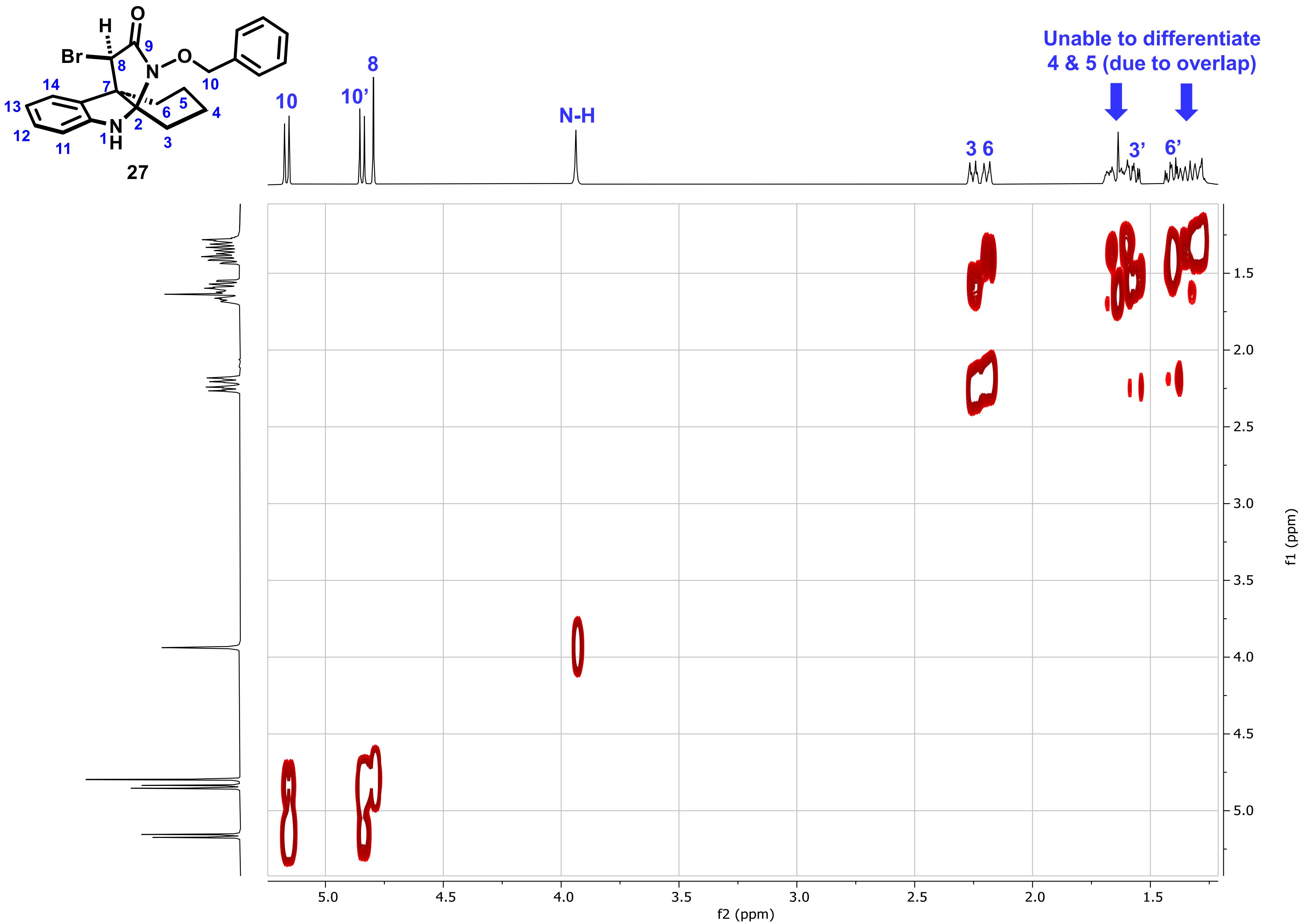

Zoomed In Version: COSY of 27

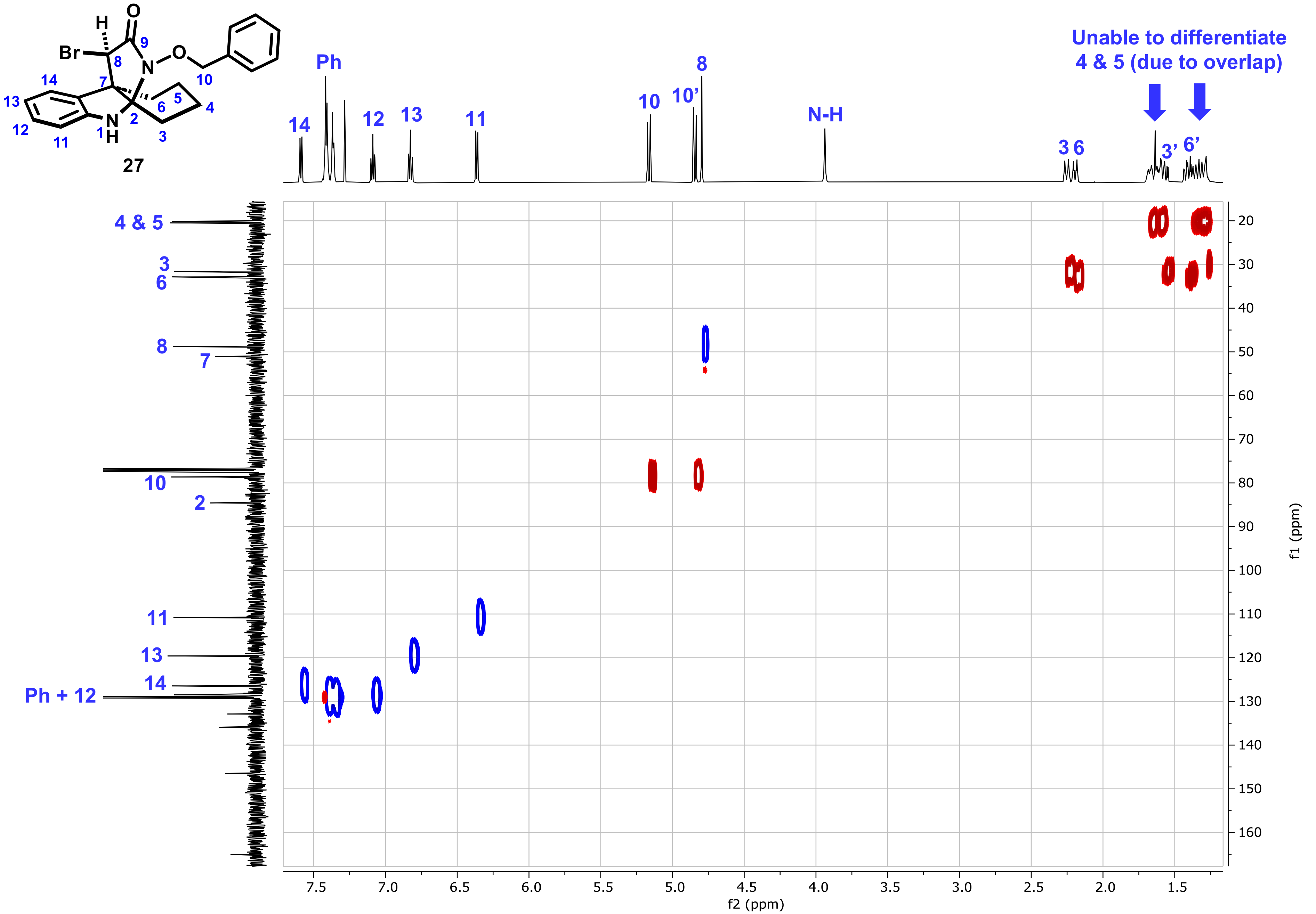

HSQC of 27
(unable to differentiate positions 4 & 5)

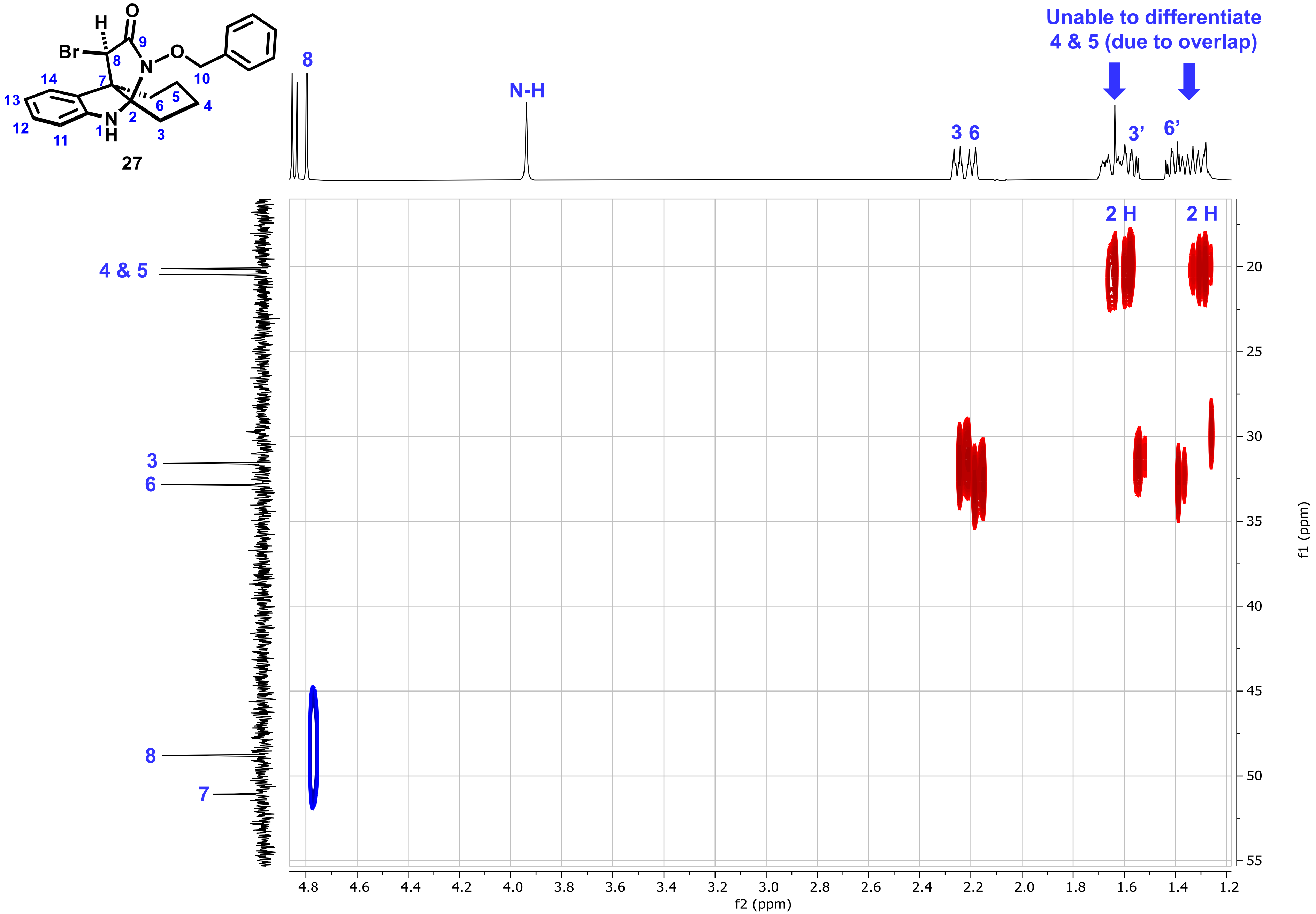

Zoomed In Version: HSQC of 27
(unable to differentiate positions 4 & 5)

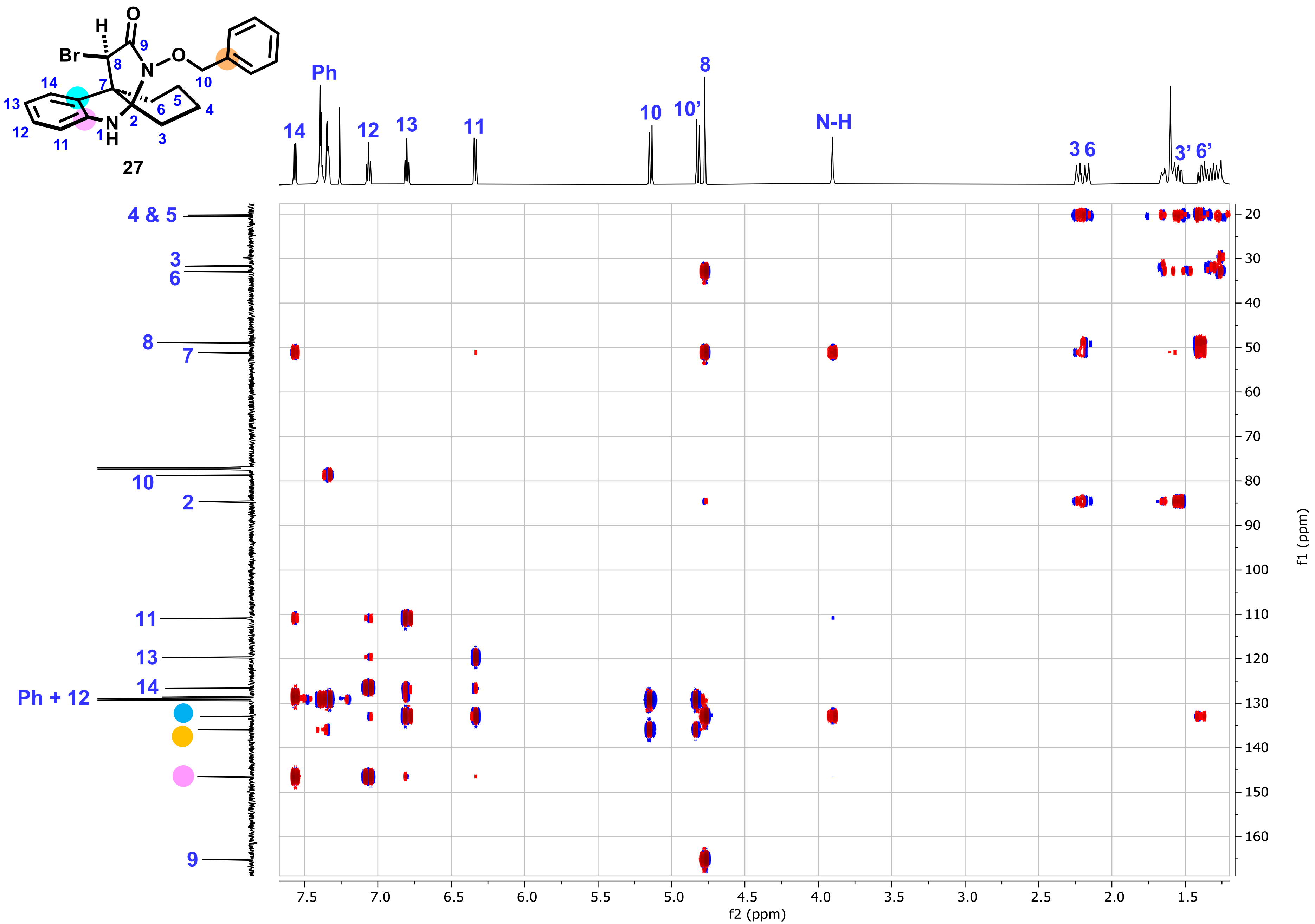

HMBC of 27
(unable to differentiate positions 4 & 5)

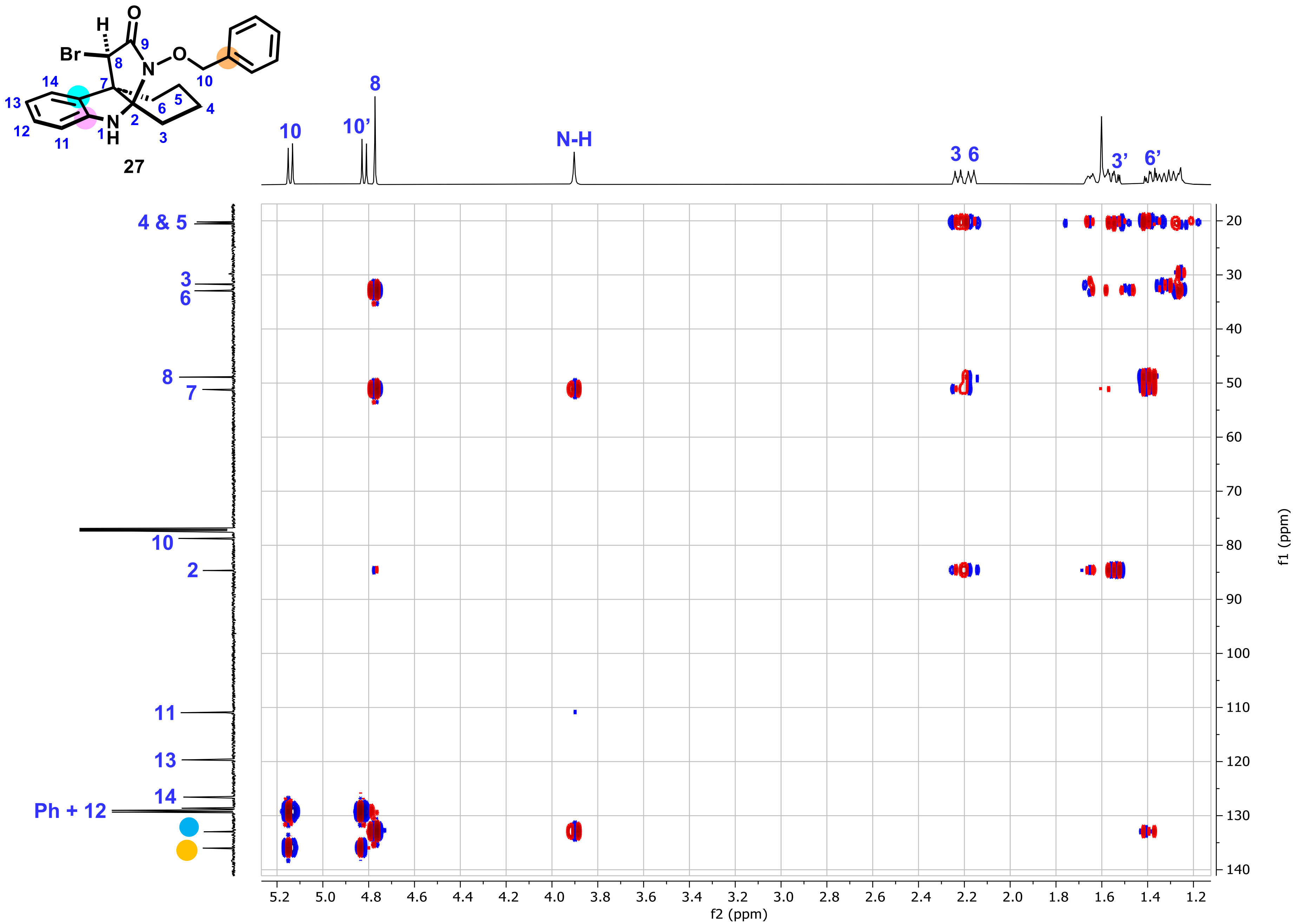

Zoomed In Version: HMBC of 27
(unable to differentiate positions 4 & 5)

Key NOE (From 8 to 6) for Stereochemical Assignment of 27
Note: Irradiation of N-H Enabled Assignment of Position 3

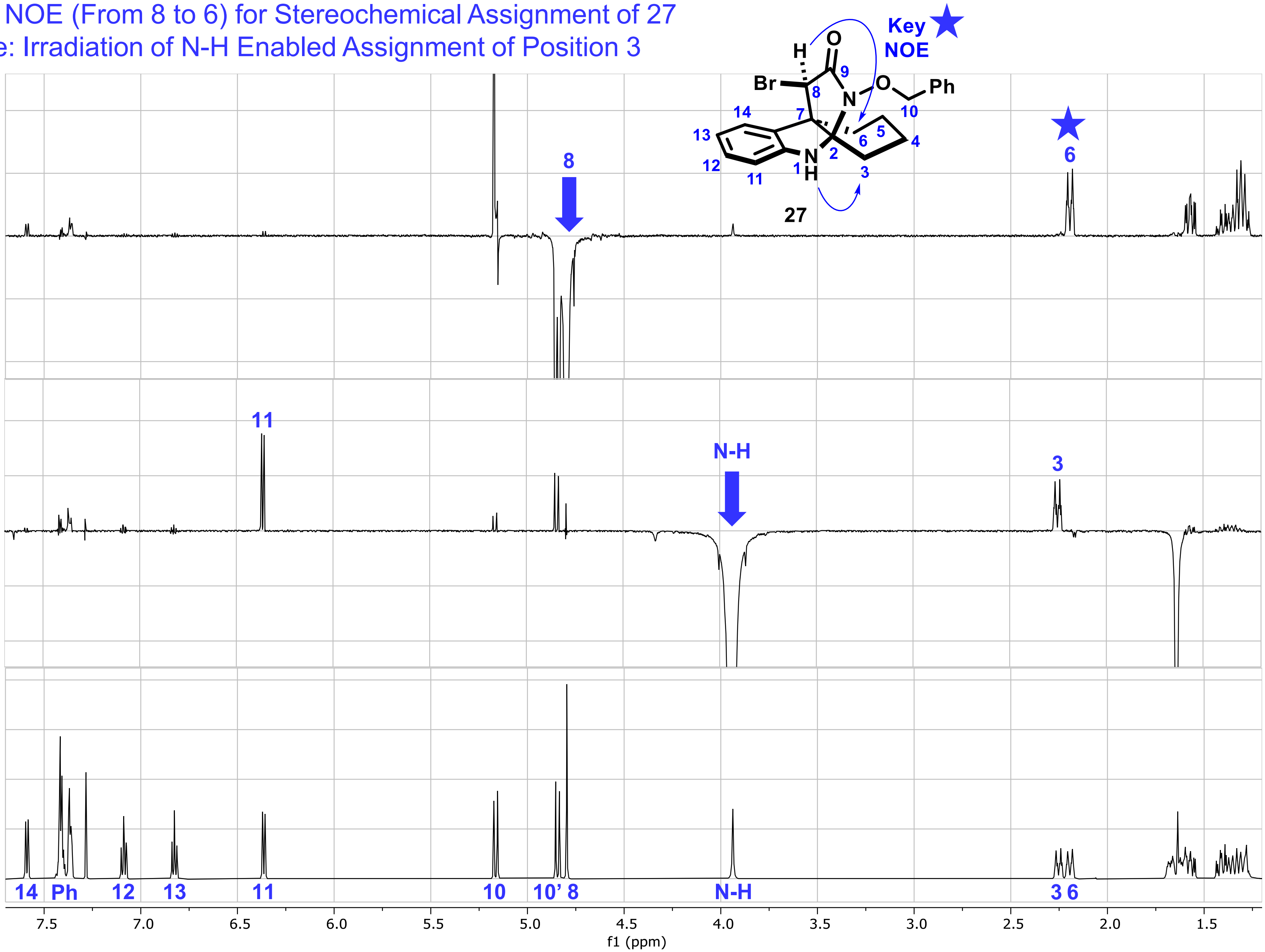

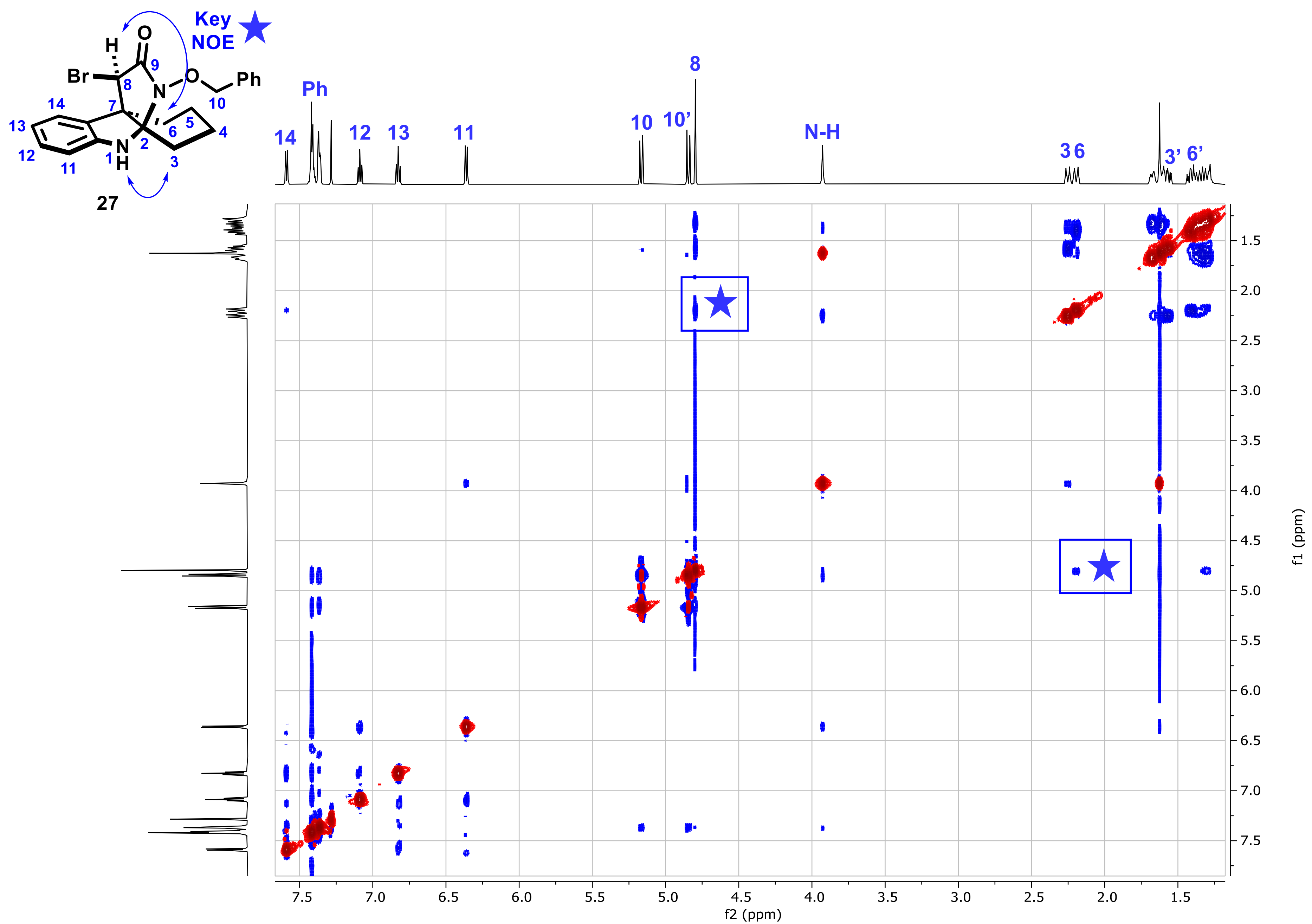

2D NOEs for Stereochemical Assignment of 27

Key NOEs Between Protons 8 and 6

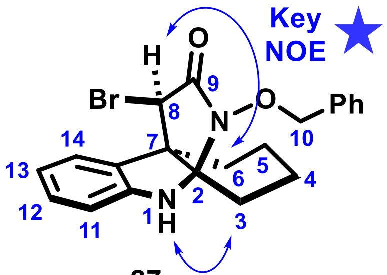

27

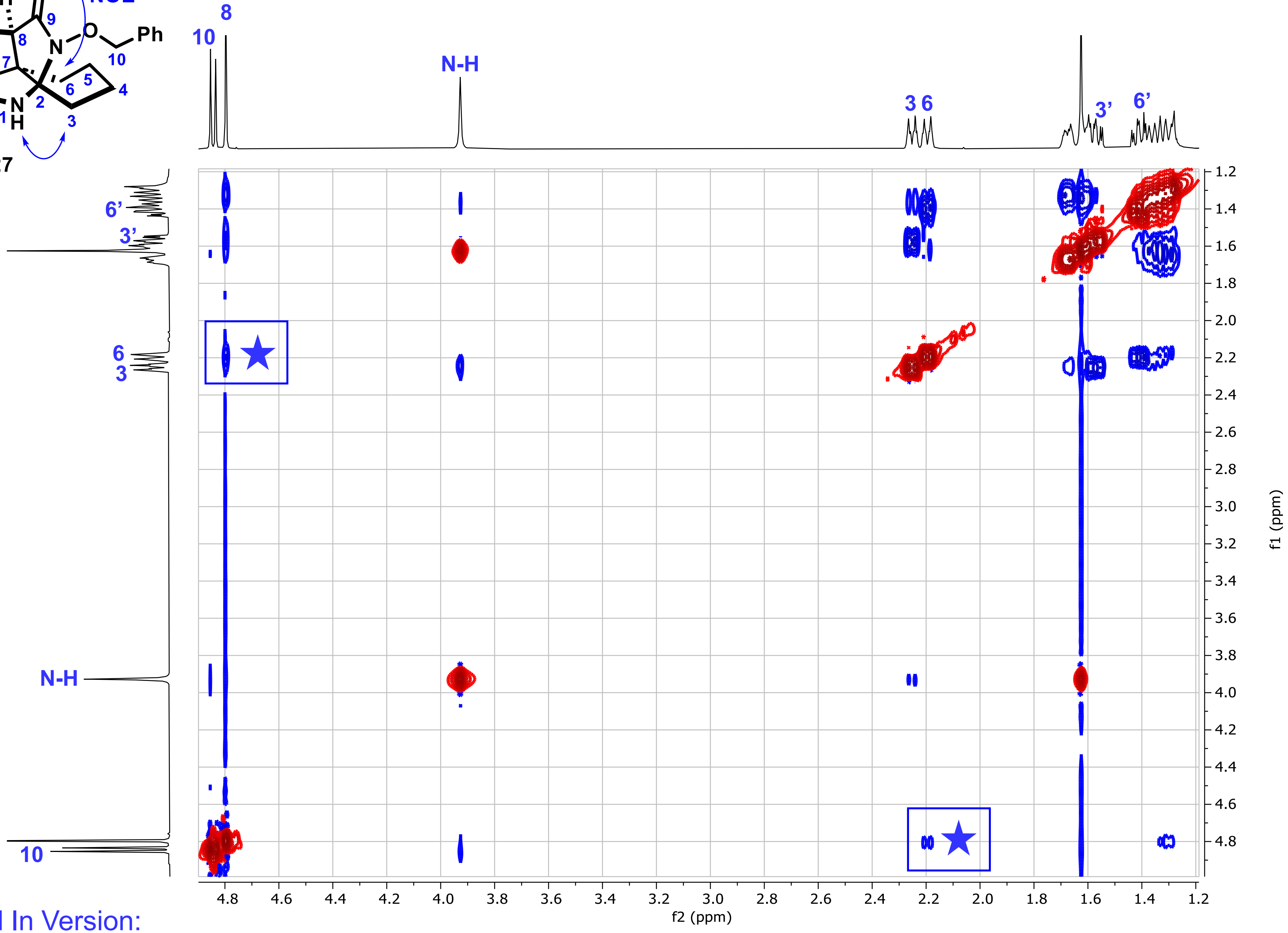

Zoomed In Version:
2D NOEs for Stereochemical Assignment of 27
Key NOEs Between Protons 8 and 6

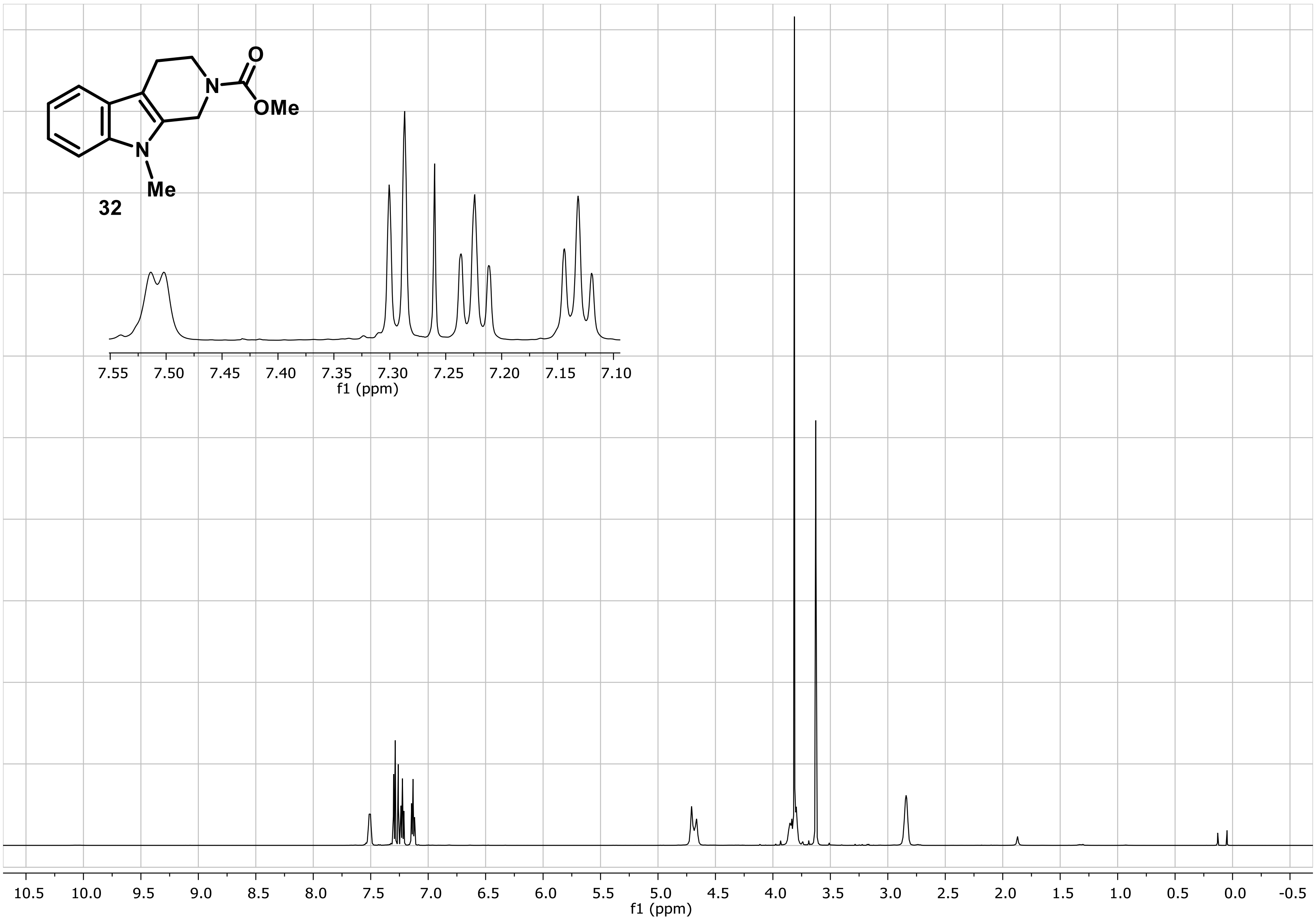
32
OMe
Me
f1 (ppm)
f1 (ppm)

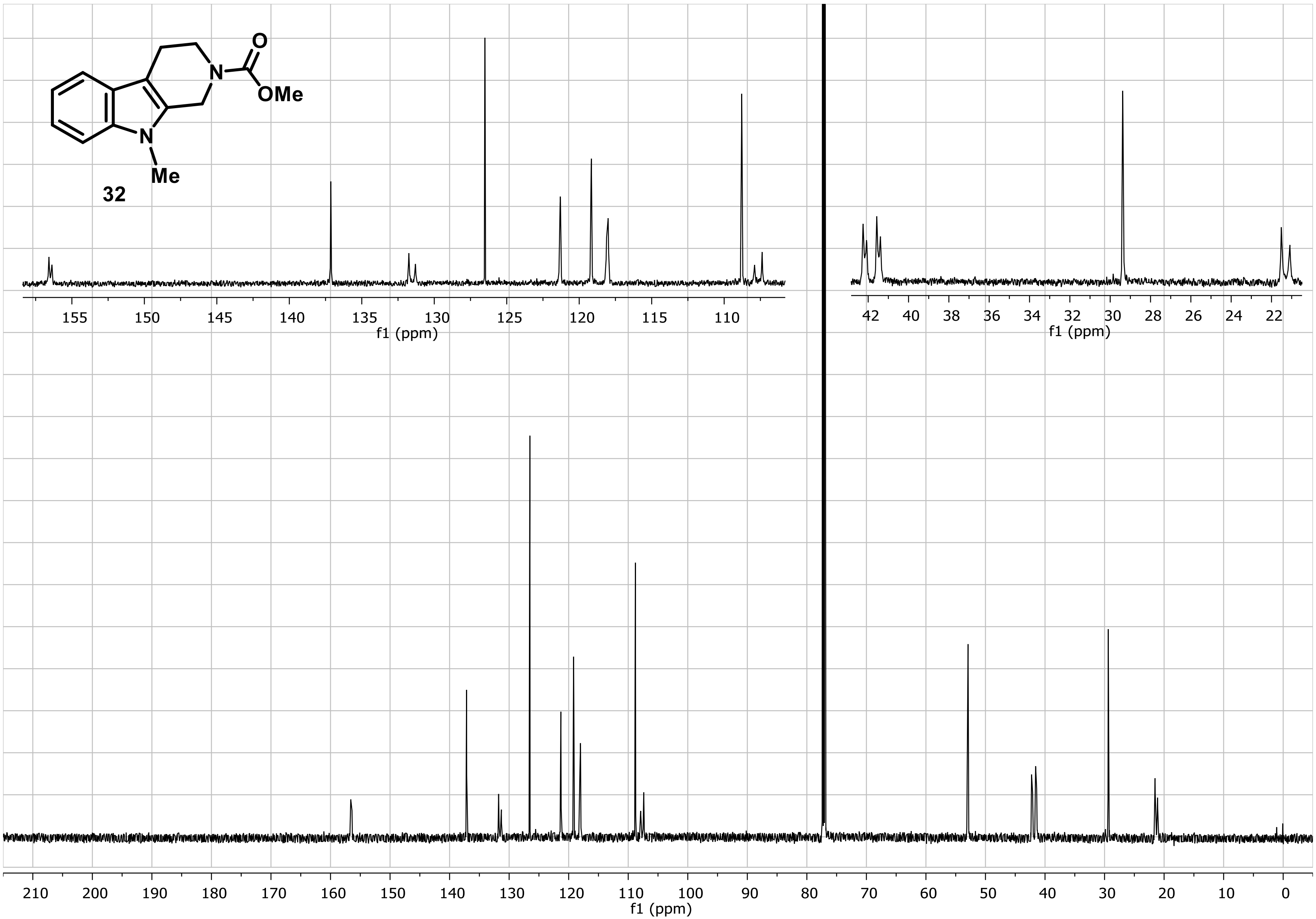
O
N
OMe
N
Me
32
155
150
145
140
135
130
125
120
115
110
f1 (ppm)
42
40
38
36
34
32
30
28
26
24
22
f1 (ppm)
210
200
190
180
170
160
150
140
130
120
110
100
90
80
70
60
50
40
30
20
10
0
f1 (ppm)

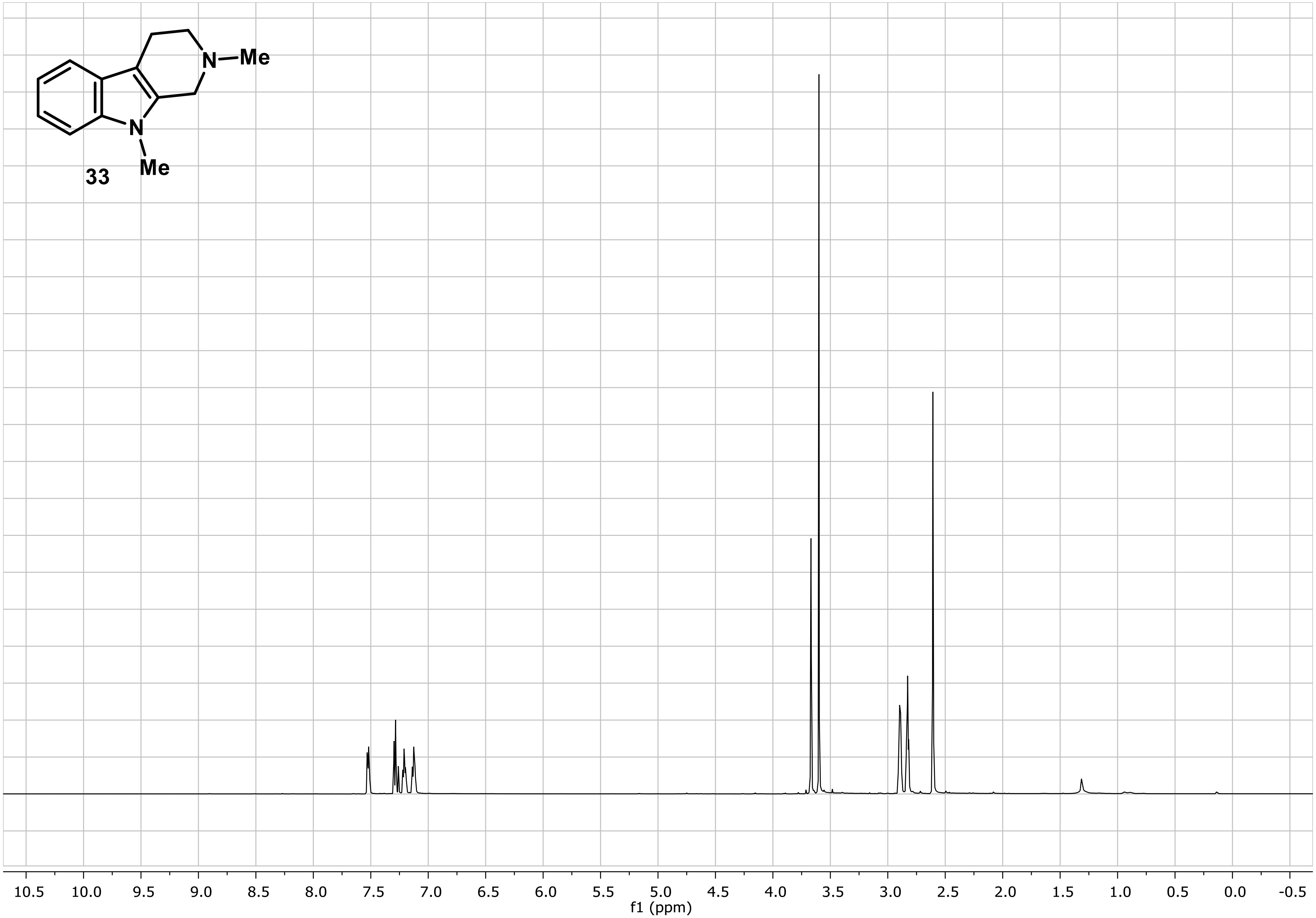
N—Me
N
Me
33
10.5 10.0 9.5 9.0 8.5 8.0 7.5 7.0 6.5 6.0 5.5 5.0 4.5 4.0 3.5 3.0 2.5 2.0 1.5 1.0 0.5 0.0 -0.5
f1 (ppm)

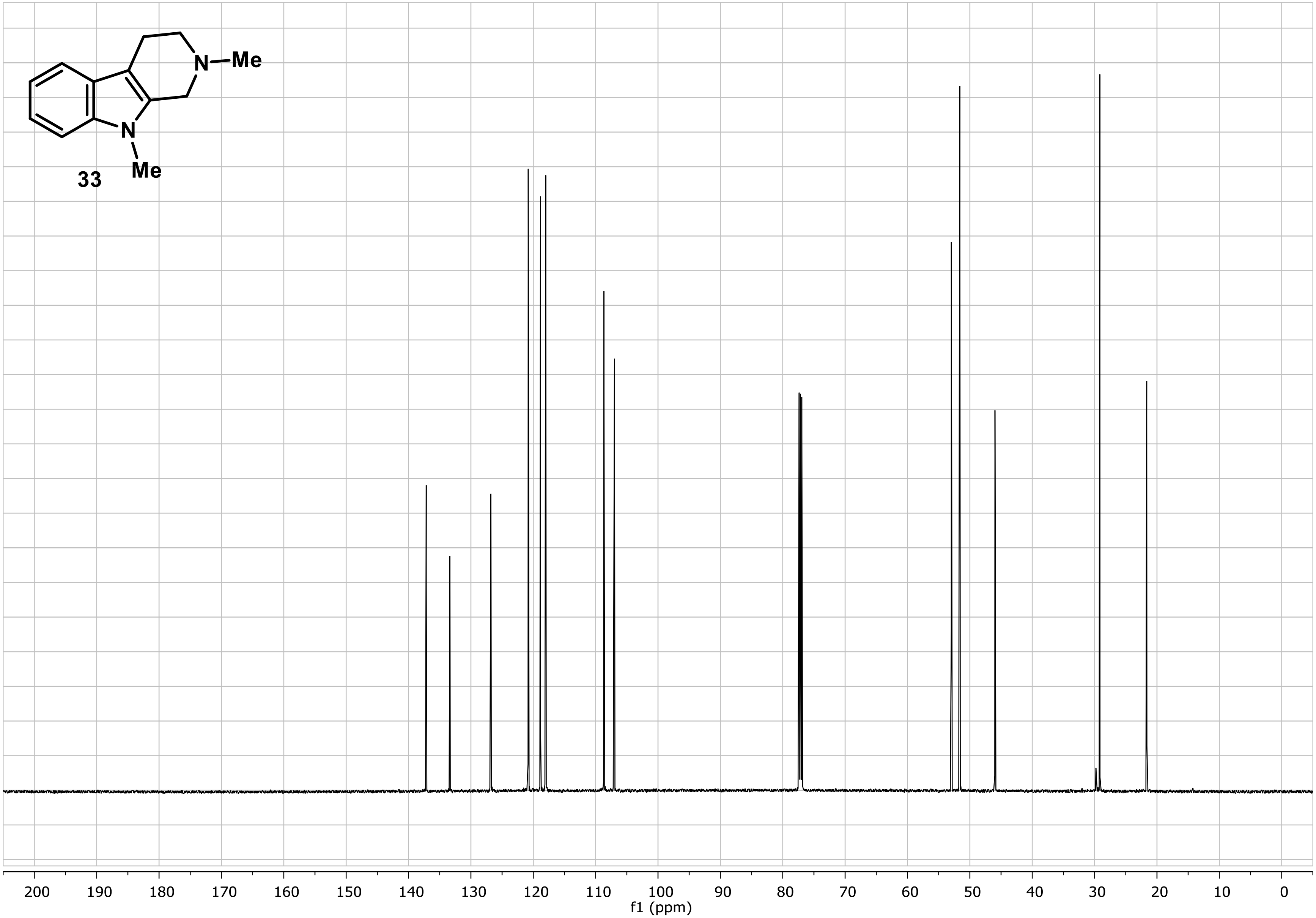

N—Me
N
Me
33
200 190 180 170 160 150 140 130 120 110 100 90 80 70 60 50 40 30 20 10 0
f1 (ppm)

Spectra On Following Slides

- ^{1}H NMR spectra of crude ring fusion reactions using ajmalicine to determine diastereomeric ratios (dr's; see integrations)

- ^{1}H NMR spectra of ajmalicine ring fusion products to demonstrate near identical NMR spectra profiles for analogues 9 & 12, and analogues 13 & 14

- NMR spectra of 1,2-dithiolane and analysis of a chemical reaction to confirm formation of 1,2-dithiolane from the bromine transfer reaction

Diastereomeric ratio for this reaction was determined by NMR analysis of the crude material following work-up.

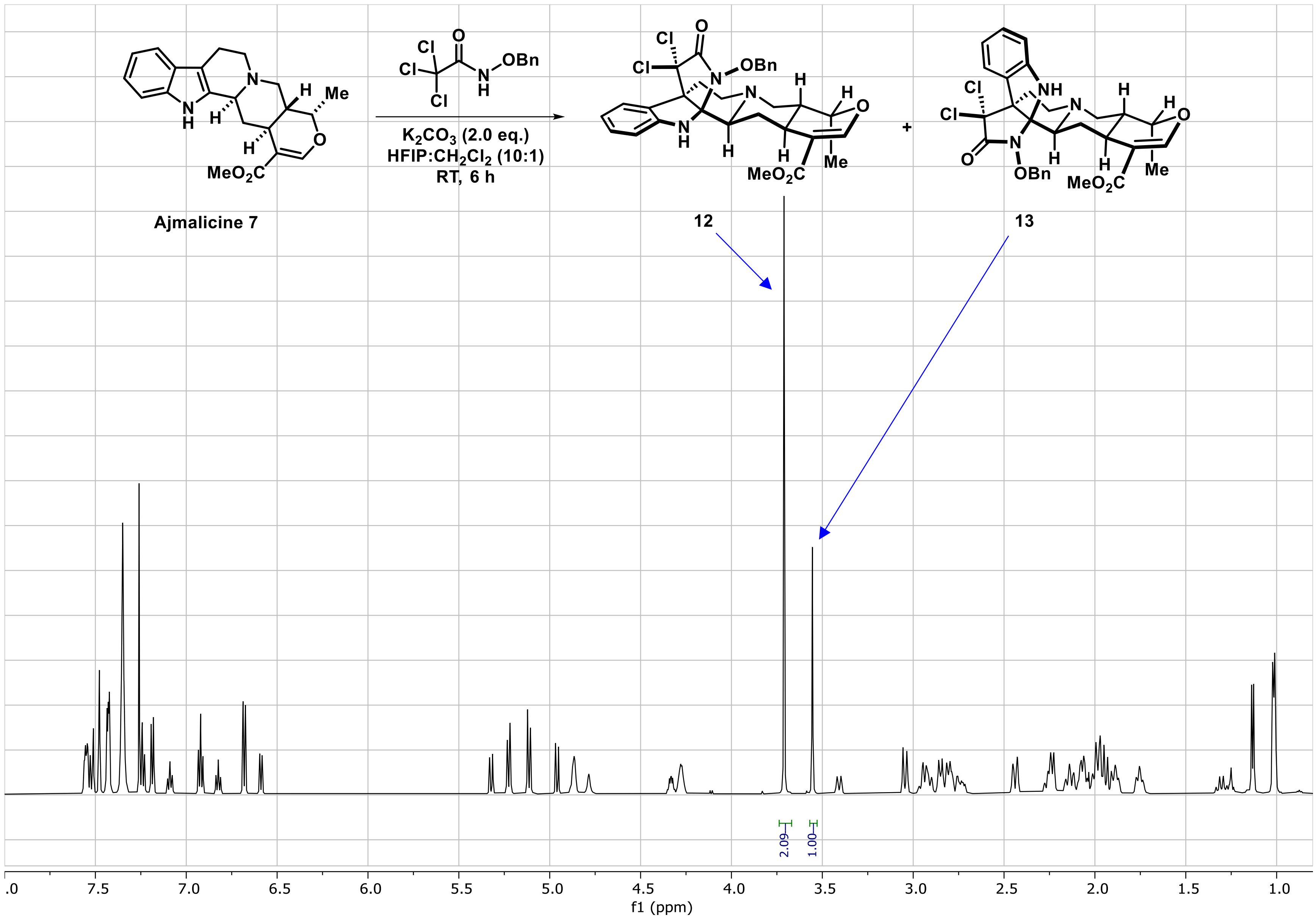

Methyl ester group ratios used to determine a 2.1:1 dr for this reaction

Diastereomeric ratio for this reaction was determined by NMR analysis of the crude material following work-up.

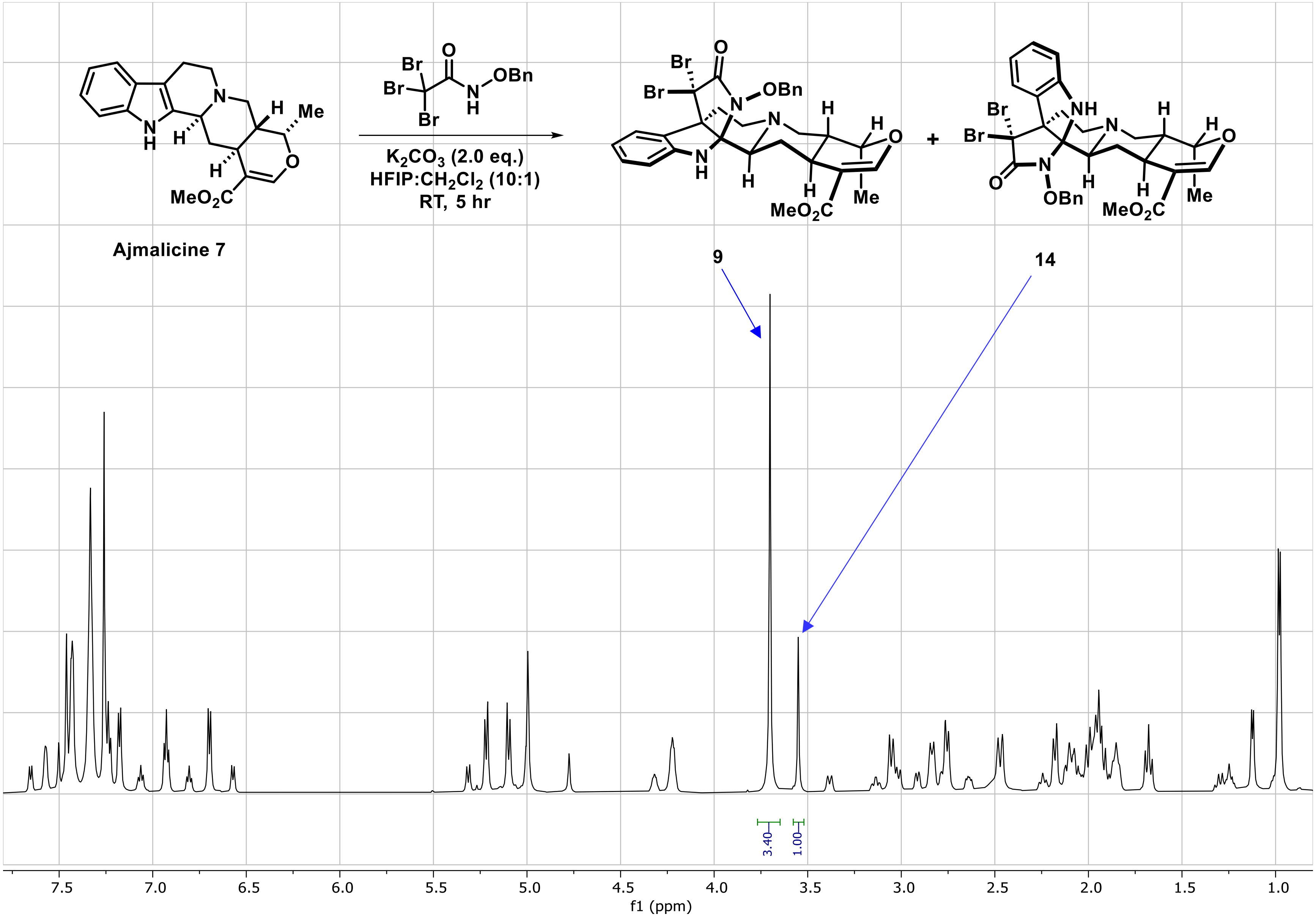

Methyl ester group ratios used to determine a 3.4:1 dr for this reaction

Diastereomeric ratio for this reaction was determined by NMR analysis of the crude material following work-up.

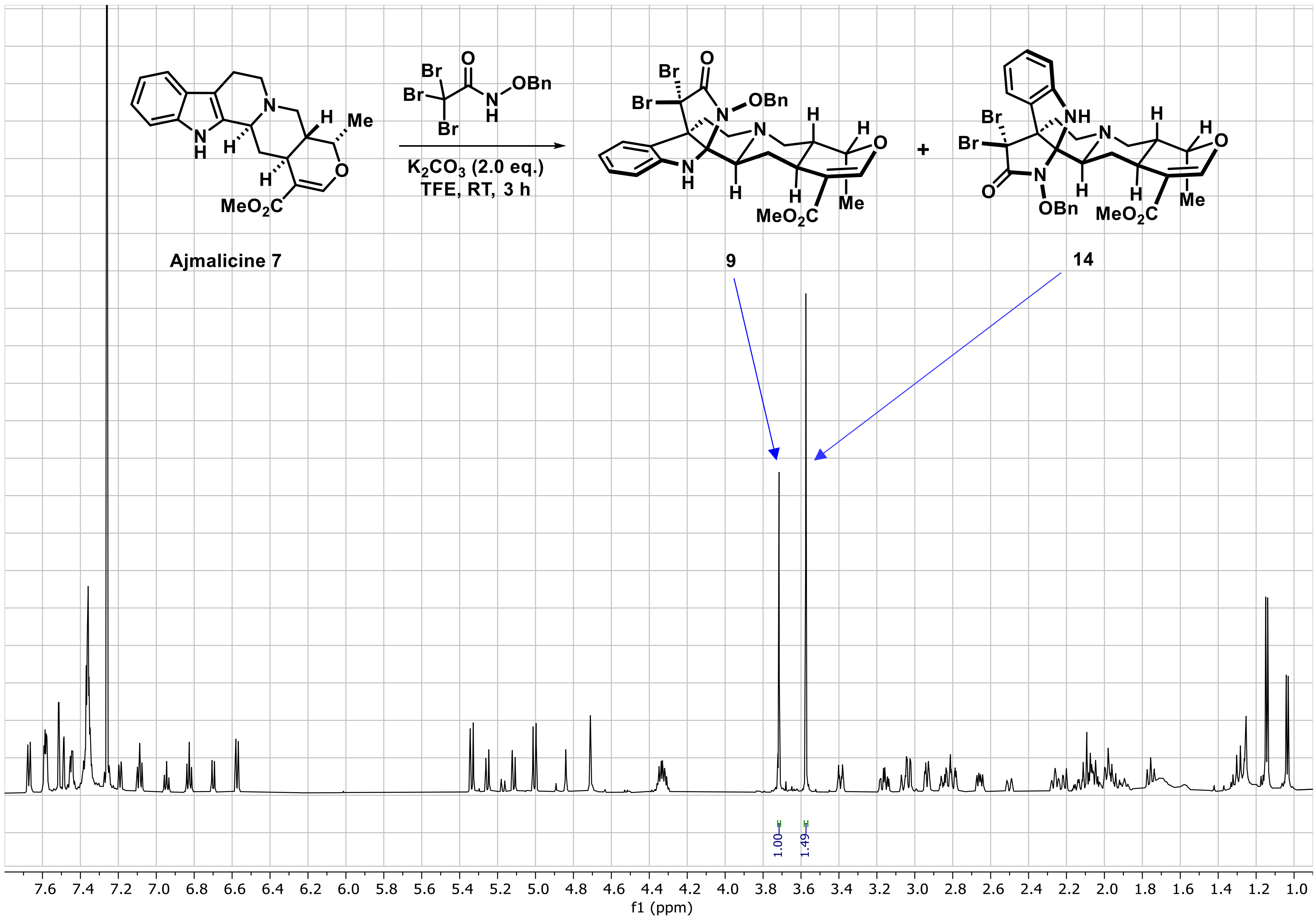

Methyl ester group ratios used to determine a 1:1.5 dr for this reaction

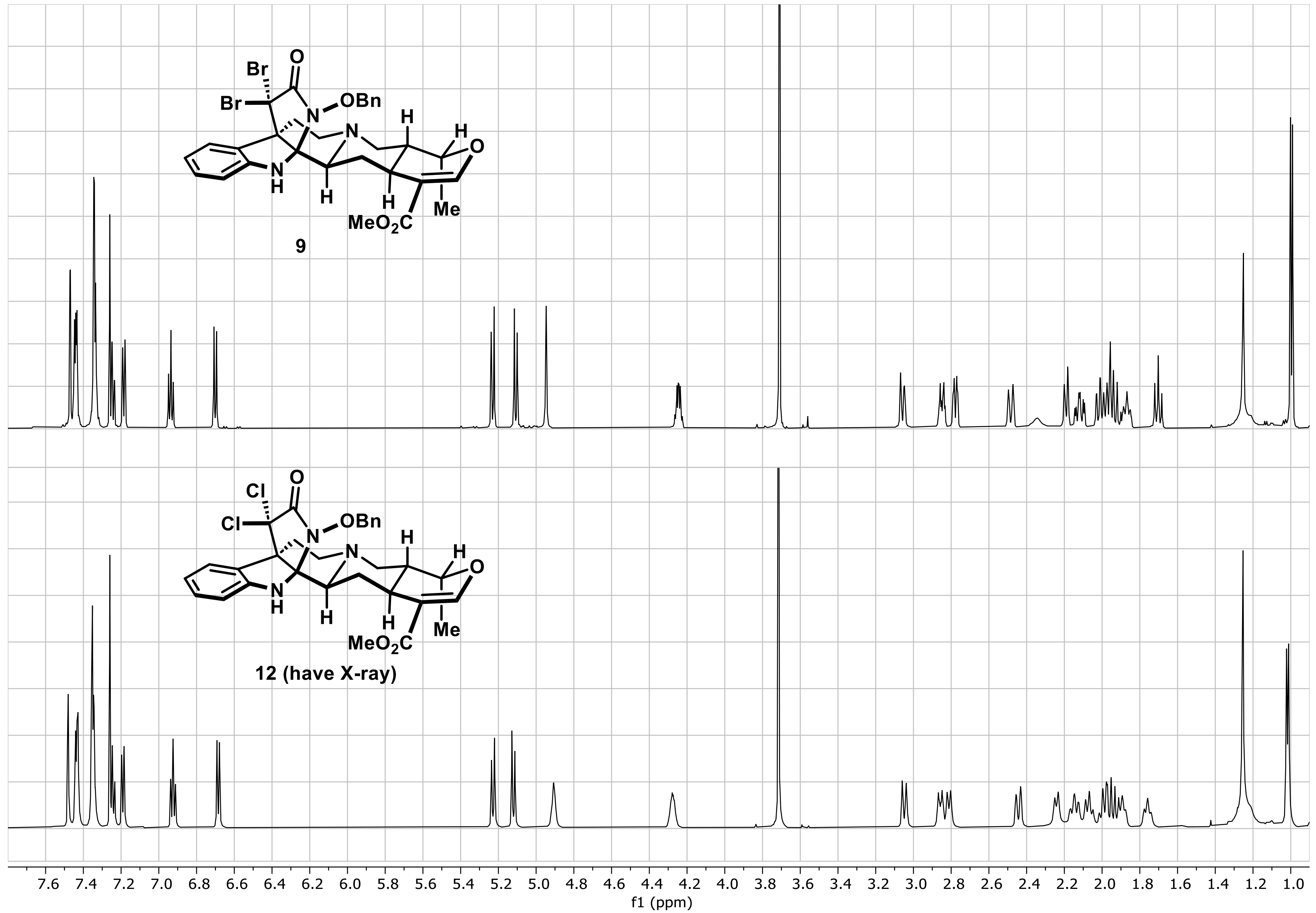

Note the similar proton NMR spectra profiles between 9 and 12

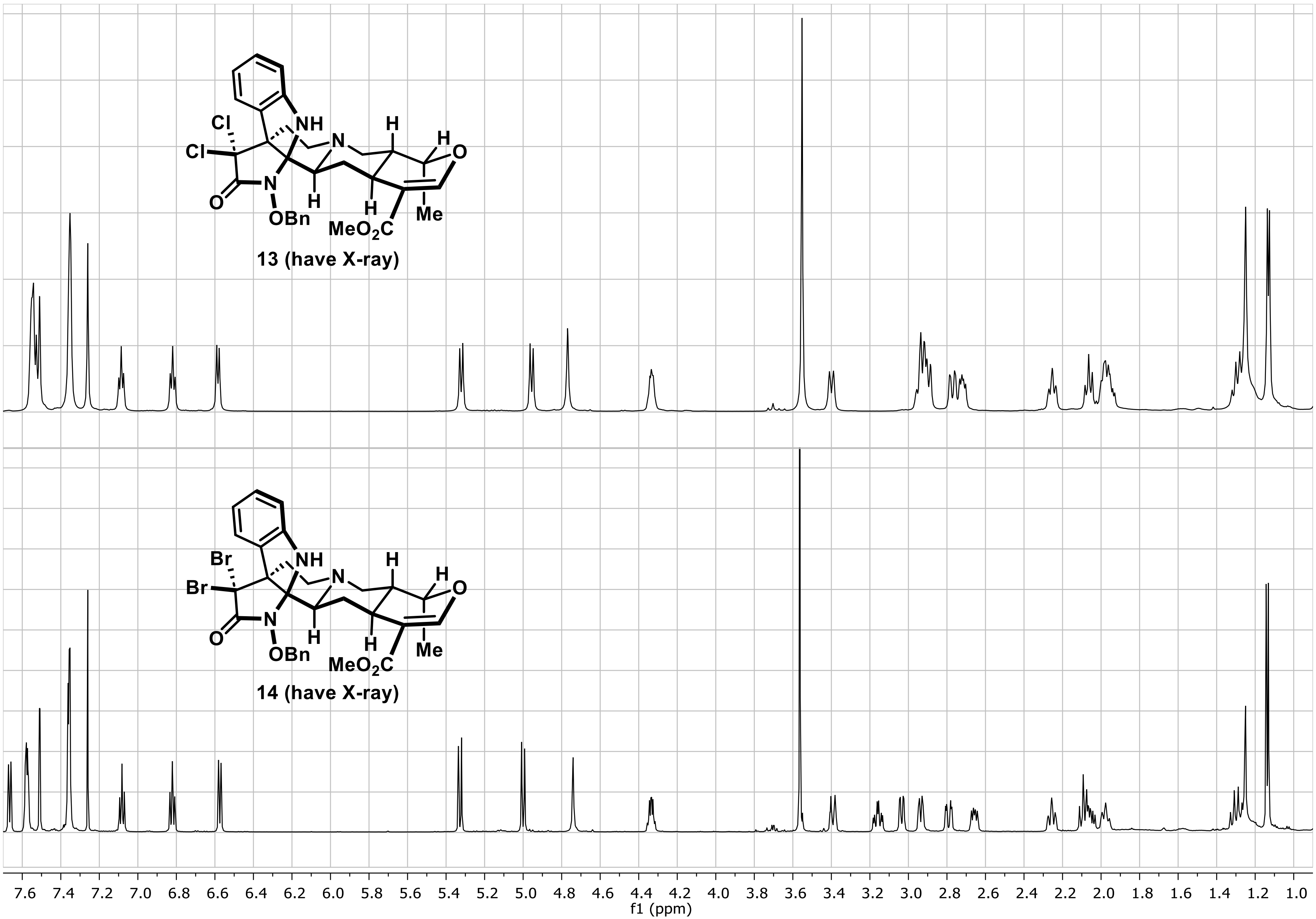

Note the similar proton NMR spectra profiles between 13 and 14

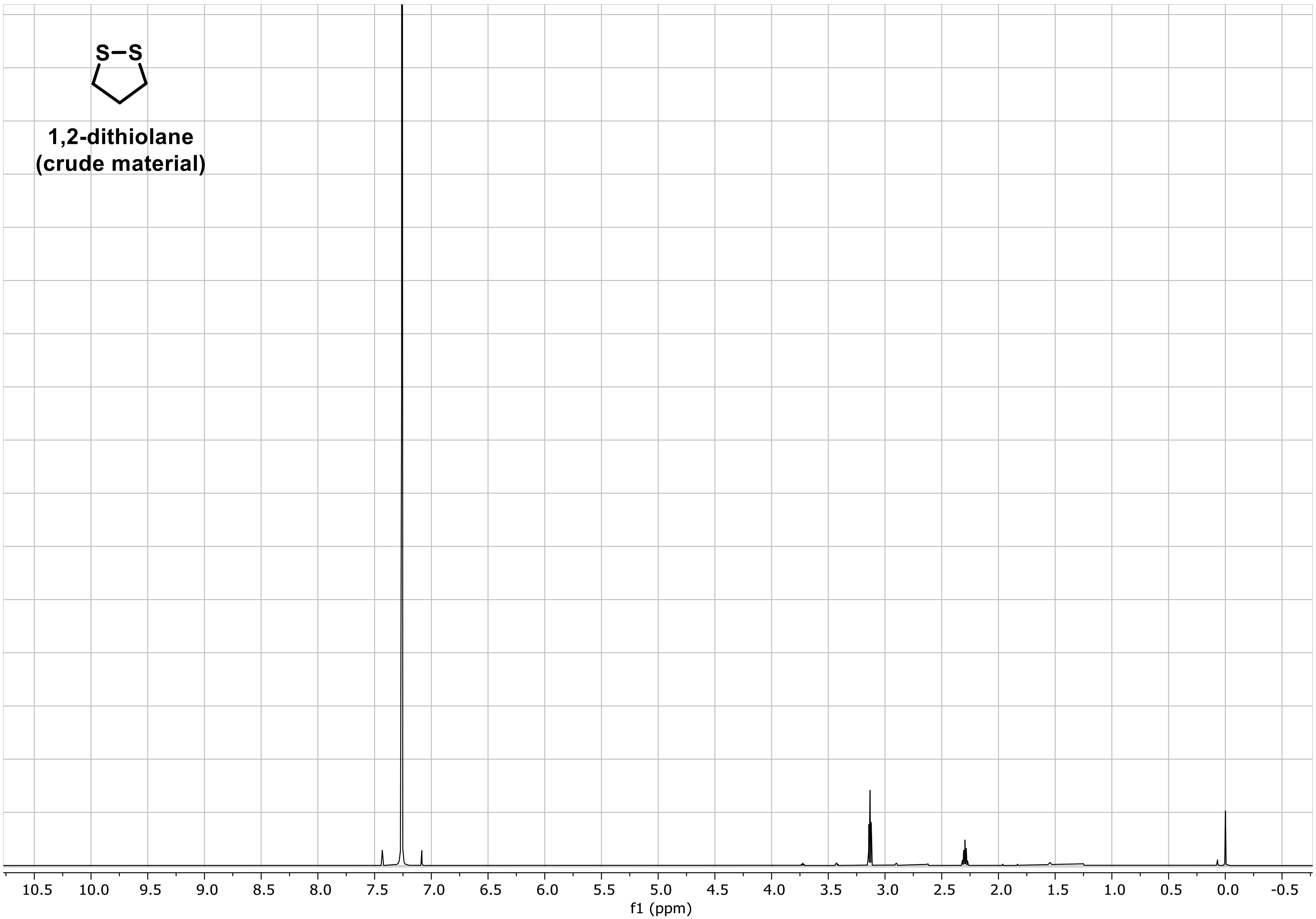

S
S
1,2-dithiolane
(crude material)
10.5
10.0
9.5
9.0
8.5
8.0
7.5
7.0
6.5
6.0
5.5
5.0
4.5
4.0
3.5
3.0
2.5
2.0
1.5
1.0
0.5
0.0
-0.5
f1 (ppm)

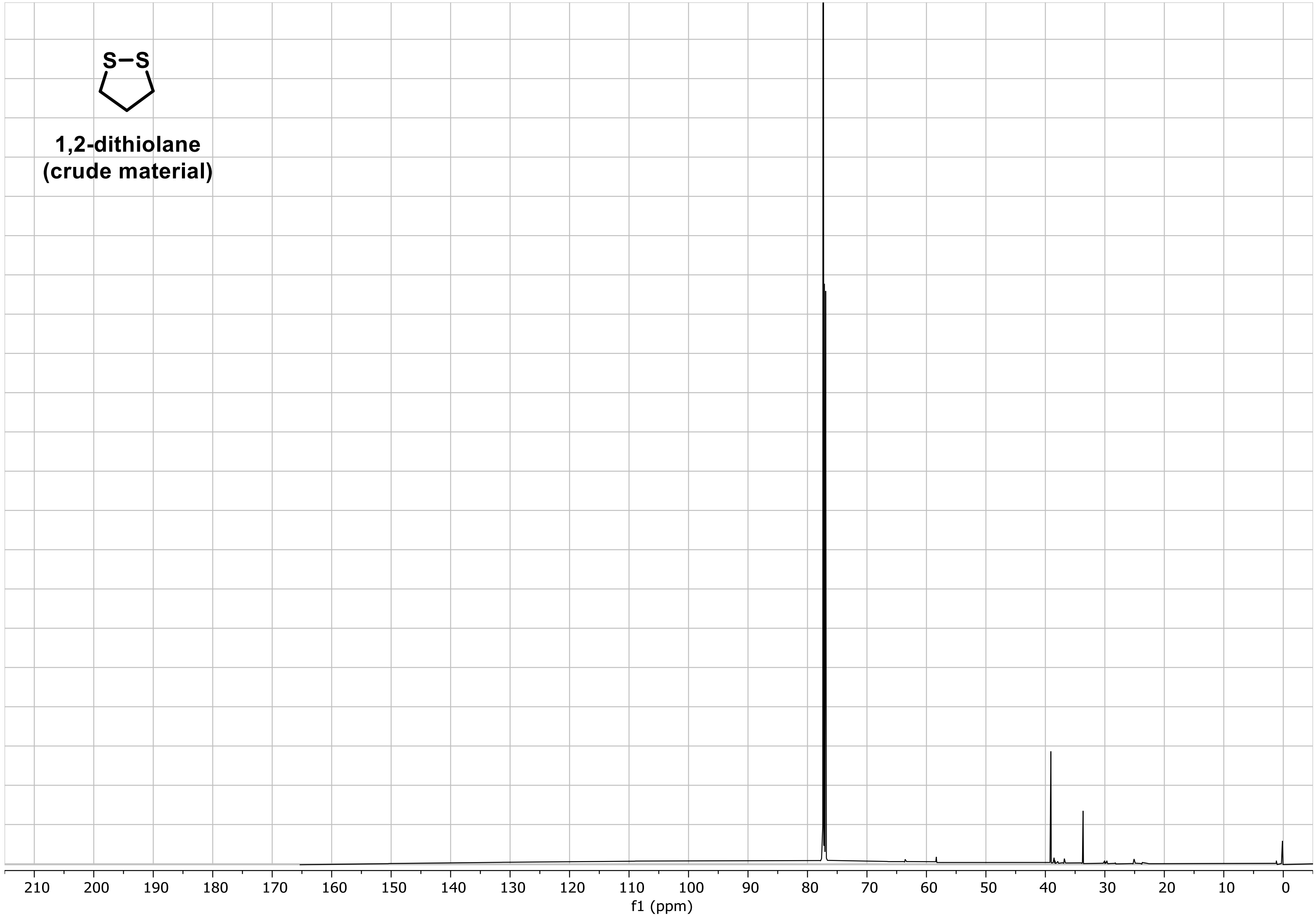
S–S
1,2-dithiolane
(crude material)
210 200 190 180 170 160 150 140 130 120 110 100 90 80 70 60 50 40 30 20 10 0
f1 (ppm)

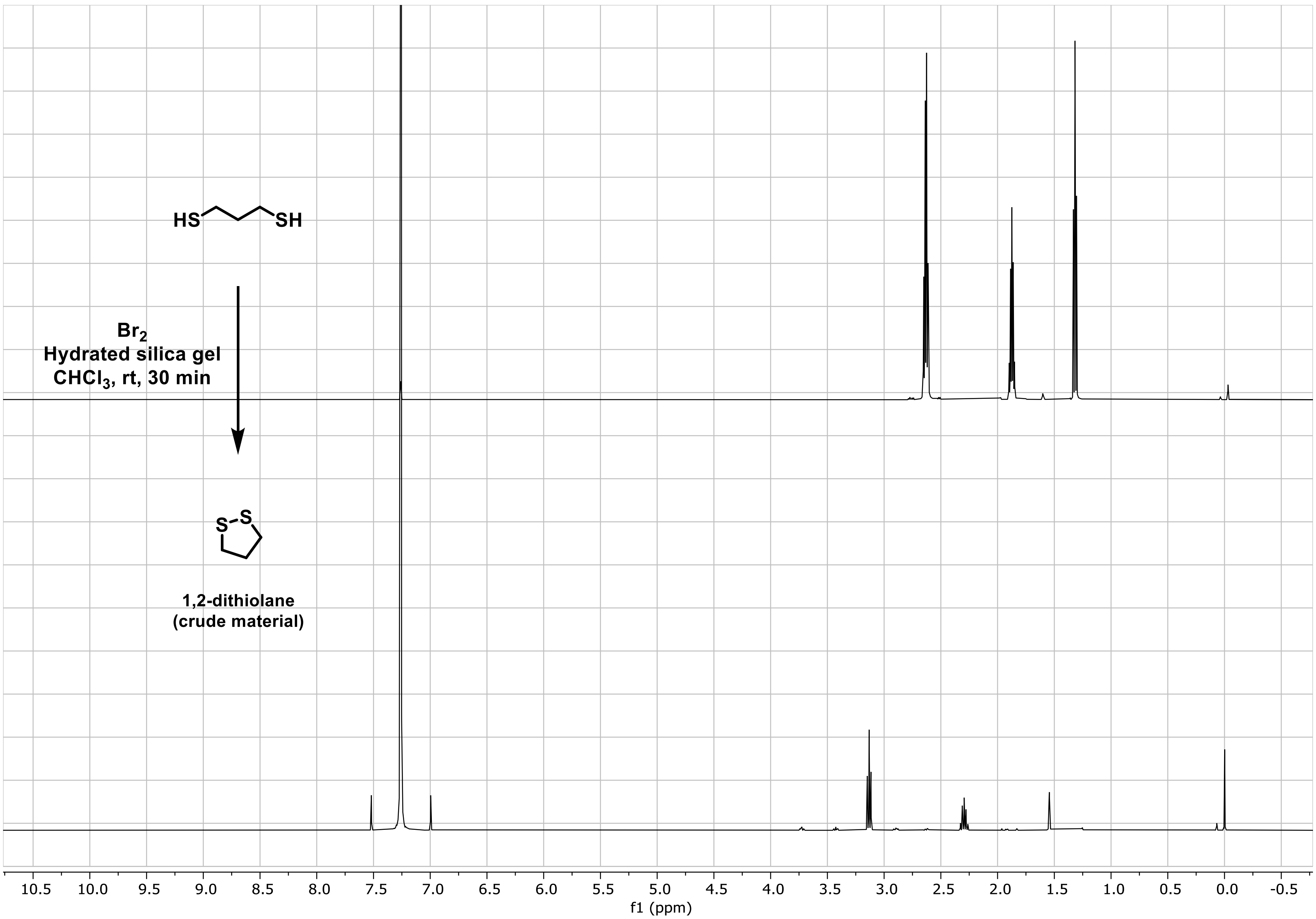

Crude reaction NMR analysis to confirm the formation of 1,2-dithiolane upon treatment of molecular bromine.

Full Version: NMR analysis (of crude reaction) to confirm 1,2-dithiolane formation upon reaction with the dibromo lactam warhead.

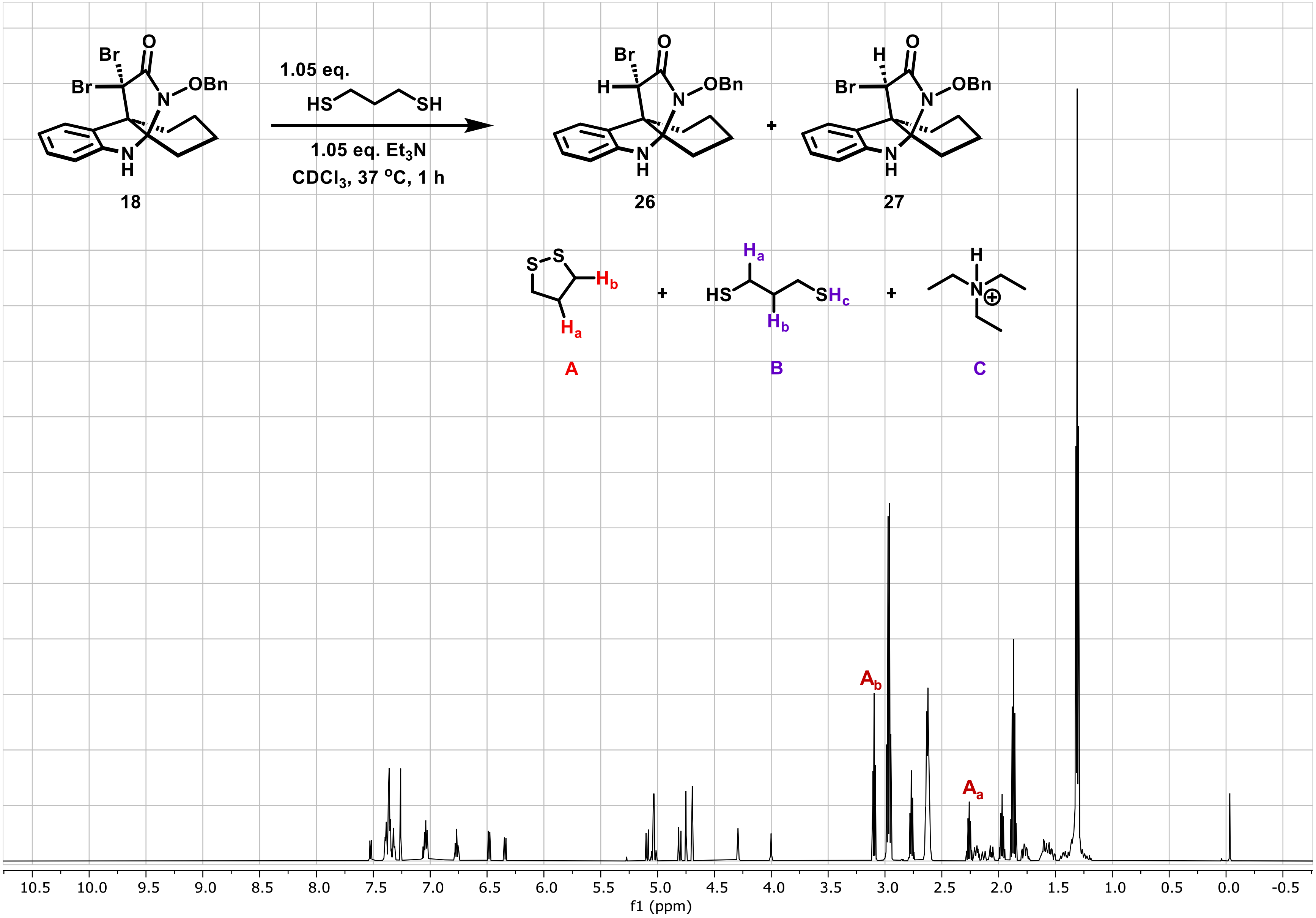

Zoomed In Version 1: NMR analysis (crude reaction) to confirm 1,2-dithiolane from reaction with the dibromo lactam warhead.

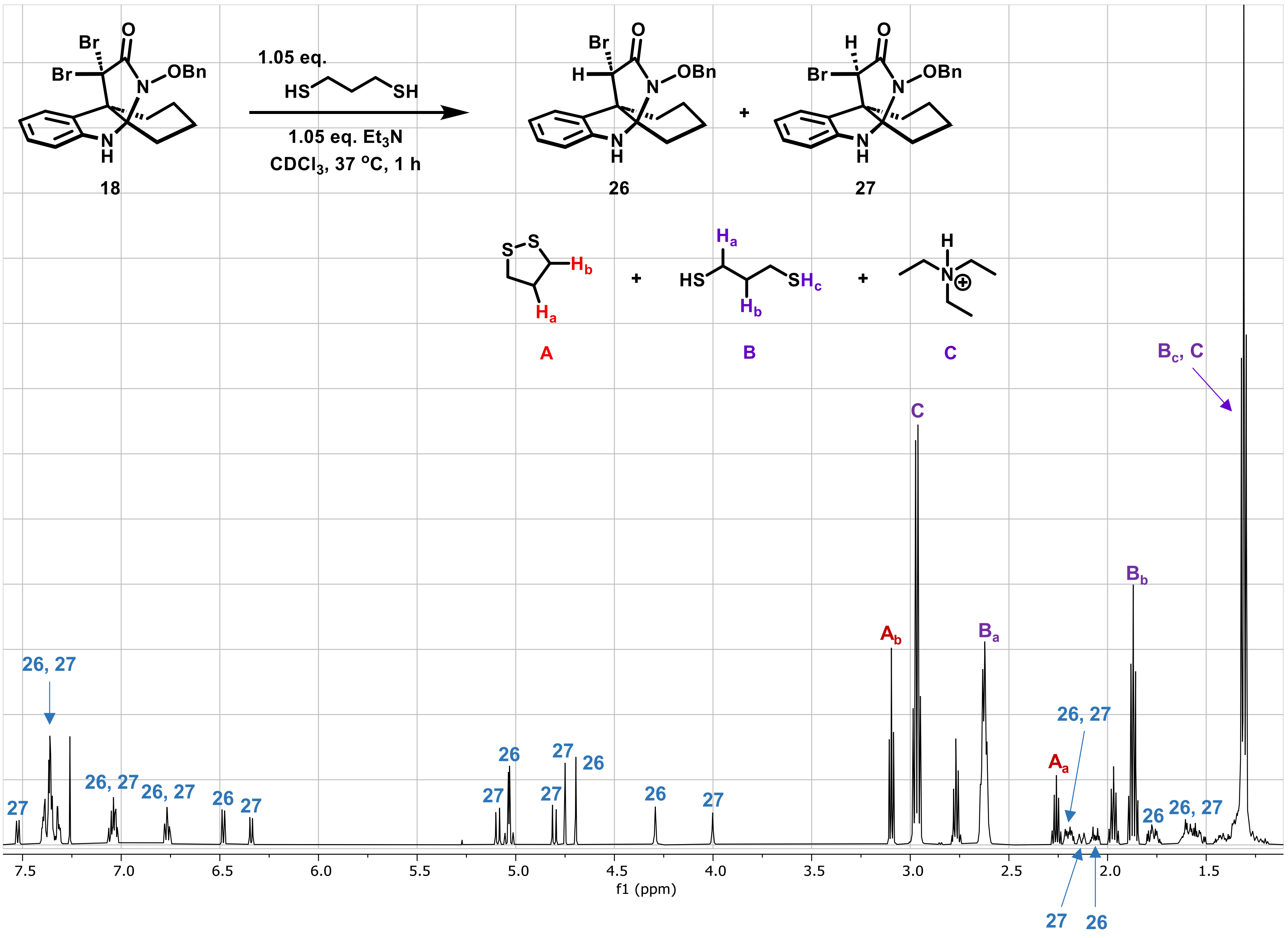

Zoomed In Version 2: NMR analysis (crude reaction) to confirm 1,2-dithiolane from reaction with the dibromo lactam warhead.

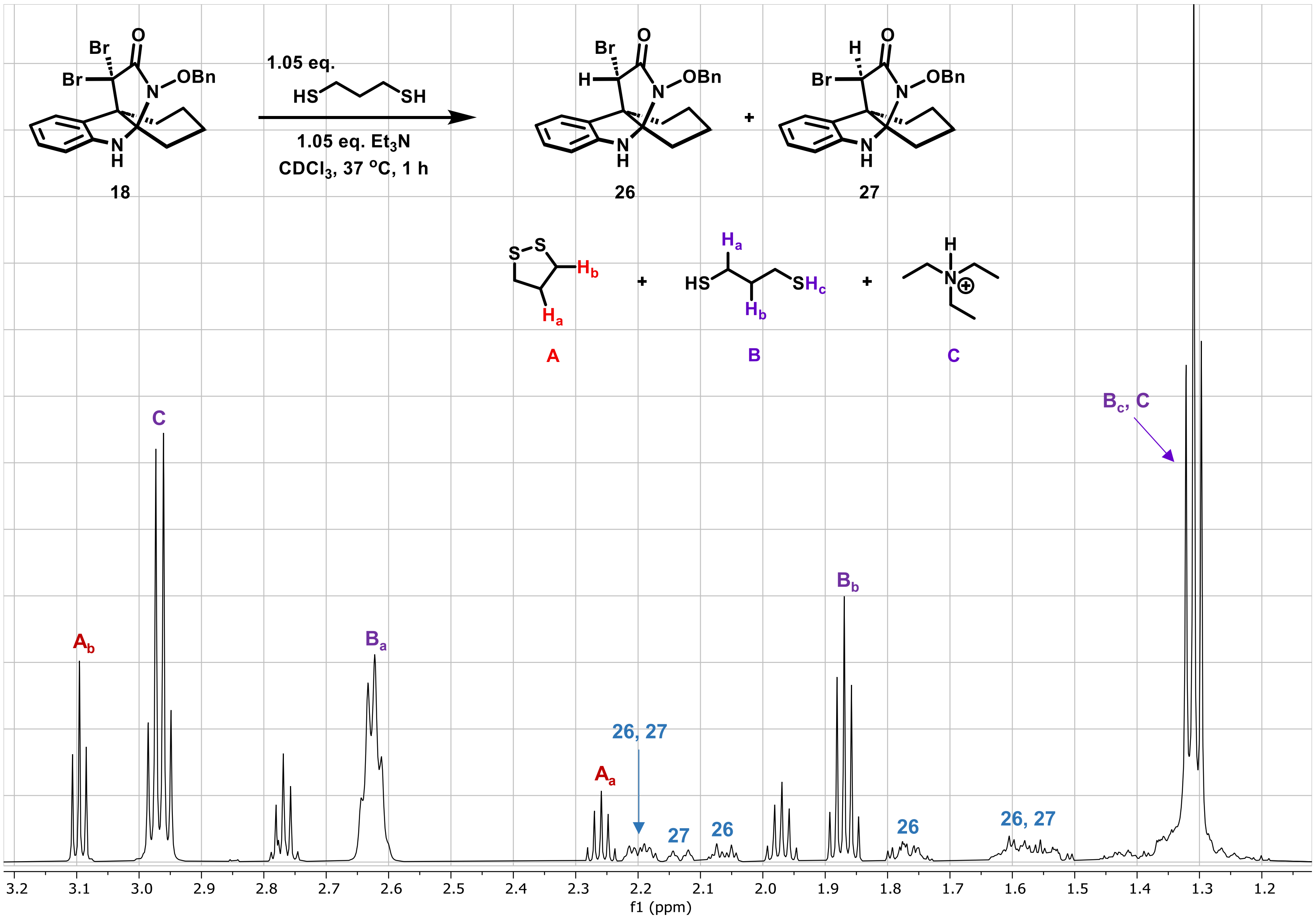

Supplement: Supplementary file 1 — Supporting File: advs75277‐sup‐0001‐SuppMat.pdf. [file ADVS-13-e12849-s001.pdf]
